# Stereodivergent, Diels–Alder-initiated organocascades employing α,β-unsaturated acylammonium salts: scope, mechanism, and application†
†Electronic supplementary information (ESI) available: Experimental procedures and characterization details for all new compounds including ^1^H and ^13^C NMR spectra, computational data, crystallographic data, chiral phase-HPLC traces. CCDC 972246. For ESI and crystallographic data in CIF or other electronic format see DOI: 10.1039/c6sc04273b



**DOI:** 10.1039/c6sc04273b

**Published:** 2016-10-21

**Authors:** Mikail E. Abbasov, Brandi M. Hudson, Dean J. Tantillo, Daniel Romo

**Affiliations:** a Department of Chemistry and Biochemistry , Baylor University , One Bear Place 97348 , Waco , Texas 76798 , USA; b Department of Chemistry , University of California-Davis , One Shields Avenue , Davis , California 95616 , USA . Email: djtantillo@ucdavis.edu

## Abstract

Chiral α,β-unsaturated acylammonium salts are novel dienophiles enabling enantioselective Diels–Alder-lactonization (DAL) organocascades leading to *cis*- and *trans*-fused, bicyclic γ- and δ-lactones from readily prepared dienes, commodity acid chlorides, and a chiral isothiourea organocatalyst under mild conditions. We describe extensions of stereodivergent DAL organocascades to other racemic dienes bearing pendant secondary and tertiary alcohols, and application to a formal synthesis of (+)-dihydrocompactin is described. A combined experimental and computational investigation of unsaturated acylammonium salt formation and the entire DAL organocascade pathway provide a rationalization of the effect of Brønsted base additives and enabled a controllable, diastereodivergent DAL process leading to a full complement of possible stereoisomeric products. Evaluation of free energy and enthalpy barriers in conjunction with experimentally observed temperature effects revealed that the DAL is a rare case of an entropy-controlled diastereoselective process. NMR analysis of diene alcohol–Brønsted base interactions and computational studies provide a plausible explanation of observed stabilization of *exo* transition-state structures through hydrogen-bonding effects.

## Abstract 2

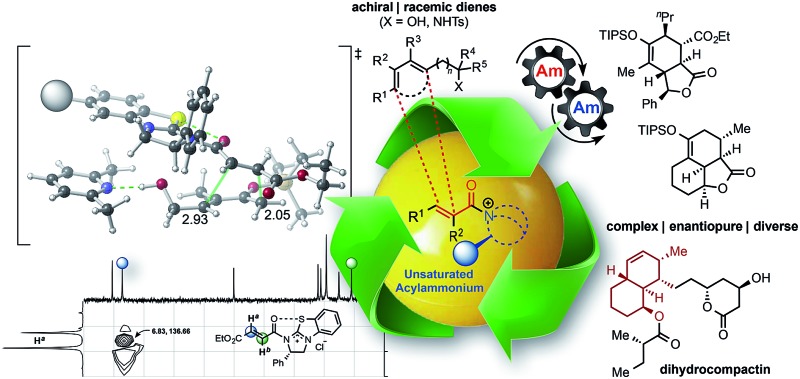
Chiral α,β-unsaturated acylammonium salts are novel dienophiles enabling enantioselective Diels–Alder-lactonization (DAL) organocascades leading to *cis*- and *trans*-fused, bicyclic γ- and δ-lactones.

## Introduction

**Fig. 1:**
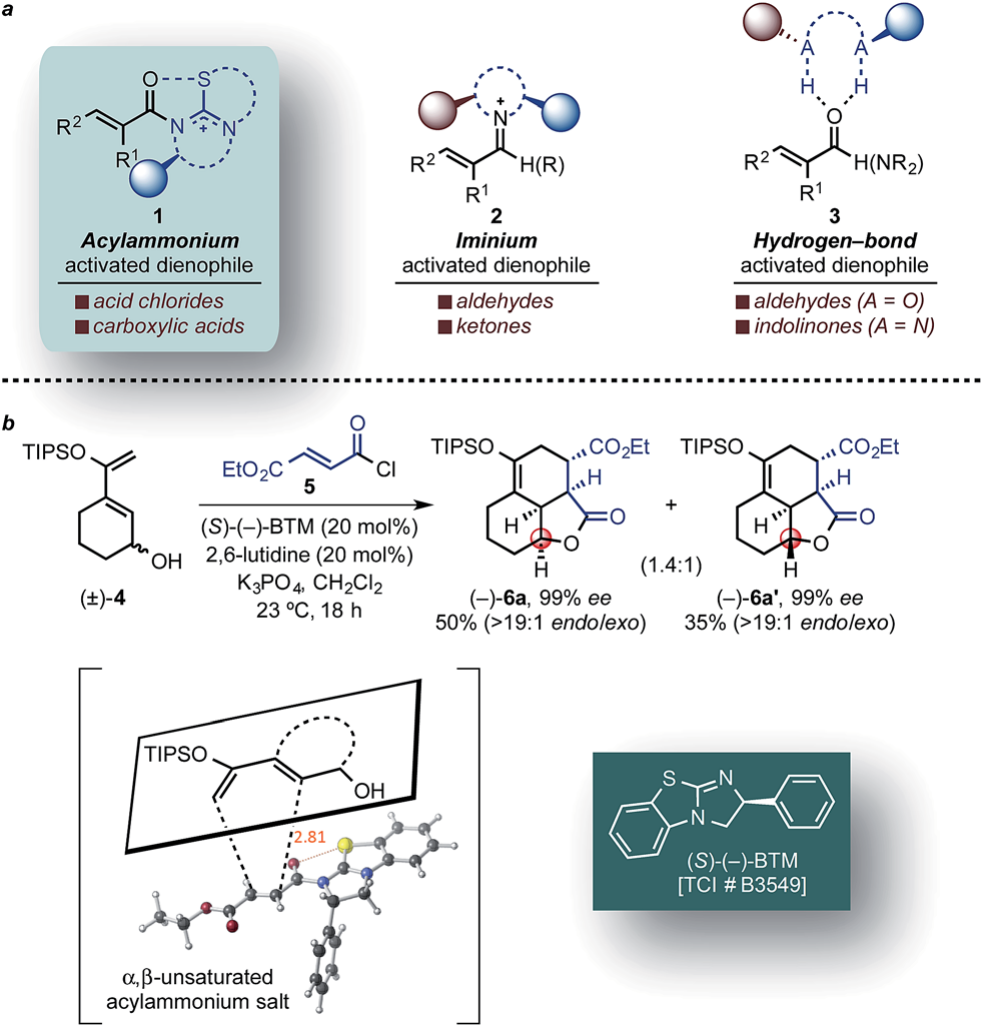
(a) Representative activation modes of α,β-unsaturated carbonyl compounds for organocatalytic asymmetric DA reactions: acylammonium salt- (this work), iminium-, and hydrogen bond-activated dienophiles. (b) Representative example of a DA-mediated, stereodivergent resolution of a racemic diene (*e.g.* (±)-**4**) employing *in situ* generated α,β-unsaturated acylammonium salt from acid chlorides (*e.g.* 5) and isothiourea catalysts (*e.g.* (*S*)-(–)-BTM) (inset, previously reported TS arrangement).

The Diels–Alder (DA) cycloaddition is arguably one of the most useful transformations in organic synthesis for the rapid introduction of complexity including stereochemical information.^1–3^ The rich history,^2^ utility, simplicity of operation, and continued evolution of strategies that broaden the scope and improve the stereoselectivity of the venerable DA reaction makes this cycloaddition arguably the most versatile and powerful transform in chemical synthesis.^3^ In particular, catalytic asymmetric DA reactions are unparalleled in their ability to rapidly and efficiently generate optically active, architecturally complex, and densely functionalized heterocycles and carbocycles from simple achiral substrates. Furthermore, enantioselective organocatalytic DA variants have recently been established using iminium,^4,5^ enamine,^6,7^ bifunctional acid–base,^8^ and hydrogen-bonding catalysis.^9,10^ MacMillan and co-workers employed both α,β-unsaturated aldehydes^4^ and ketones^5^ in DA cycloadditions through iminium-activated chiral dienophiles **2**, whereas unsaturated aldehydes^9^ and indolinones^10^ were activated through hydrogen-bonding catalysis (**3**) by Rawal and Barbas, respectively (Fig. 1a). Two extensions of the DA reaction that have received little attention are strategies for accessing the full complement of stereoisomers of a given cycloadduct and stereodivergent DA cycloadditions.^11–19^

The development of organic transformations that provide access to a full stereochemical array of a particular adduct bearing multiple stereocenters, *i.e.* diastereodivergent processes, remains a notable challenge in chemical synthesis.^20^ Such processes have impact beyond the realm of synthetic chemistry given that biological properties of organic molecules correlate to their three-dimensional architecture and thus the relative and absolute stereochemical configuration of each stereocenter in a molecule.^21^ In particular, the ability to access all stereoisomers of a target molecule allows evaluation of stereochemical structure–activity relationships. To date, this has been realized through a limited set of reactions including conjugate addition,^12^ Mannich reaction,^13,14^ intramolecular allylic substitution,^15^ deracemization,^16^ thio-Michael addition,^17^ hydrohydroxyalkylation,^18^ and α-allylation of aldehydes.^19^ The complex stereoselectivity issues inherent to the venerable DA cycloaddition leading to cycloadducts with up to four stereocenters makes this a challenging reaction to develop into a highly selective diastereodivergent process that would enable access to all possible diastereomers. While enantiomeric cycloadducts are readily obtained using the optical antipode of catalysts, altering diastereoselectivity through relative stereochemical control in DA cycloadditions is a much more challenging endeavour.

Synthetic methods that efficiently transform racemic mixtures into complex, enantioenriched products are important components of modern organic chemistry but remain scarce.^1–3^ These include underutilized stereodivergent processes, which convert racemic starting materials to non-enantiomeric products (*e.g.* diastereomers).^11^ Catalytic asymmetric variants of these reactions with racemic substrates represent a relatively unexploited strategy toward accessing a full complement of stereoisomers. Sarpong described an elegant example of a stereodivergent process applied to natural product synthesis;^22^ however, the majority of these reactions suffer from the crucial, practical issue of inseparable, diastereomeric products.^11^

We recently reported a novel class of dienophiles that utilize covalent,^23^ organocatalytic activation of α,β-unsaturated acid chlorides and carboxylic acid tosyl anhydrides enabling an asymmetric Diels–Alder-lactonization (DAL) organocascade.^24^ Importantly, this was the first example of a DA-initiated, stereodivergent organocascade (Fig. 1b) delivering complex and stereochemically diverse scaffolds found in bioactive compounds with excellent relative and absolute stereocontrol. Herein, we describe additional examples of stereodivergent DAL processes leading to complex, polycyclic adducts, new applications toward formal syntheses of natural products or core structures, and the potential of this reaction for diversity-oriented synthesis through diastereodivergent DAL processes. The latter is made possible by the discovery that diastereoselectivity of the DAL can be altered by judicious choice of Brønsted-base additive. Finally, we provide evidence that these organocascades may be rare examples of reactions in which diastereoselectivity is entropy-driven.

**Fig. 2:**
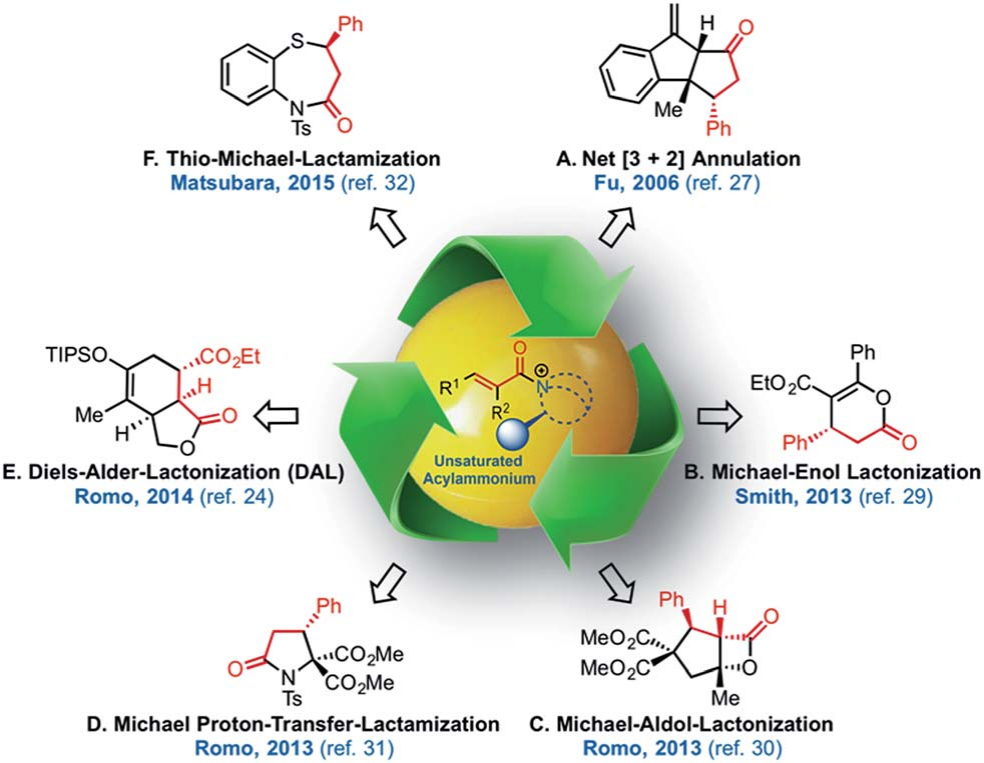
The expanding utility of α,β-unsaturated acylammonium salt-mediated, organocascade catalysis.

The potential of α,β-unsaturated acylammonium salts as catalytic intermediates (Fig. 2)^25,26^ was first demonstrated by Fu employing α,β-unsaturated acyl fluorides in a net [3 + 2] annulation promoted by a chiral 4-pyrrolidinopyridine catalyst.^27^ Building on this early work, the Lupton group reported an additional [3 + 2] cycloaddition,^28^ and the Smith group^29^ recently utilized α,β-unsaturated mixed anhydrides in an enantioselective tandem Michael-enol-lactonization. Furthermore, we demonstrated the full potential of chiral, triply reactive, α,β-unsaturated acylammonium salts derived from commodity acid chlorides for the rapid assembly of complex cyclopentanes through a nucleophile-catalyzed Michael-aldol-β-lactonization organocascade (NCMAL).^30^ Optically active γ-lactams and piperidones could also be prepared through a Michael-proton transfer-lactamization (NCMPL)^31^ process utilizing these intermediates. Most recently, Matsubara described the first example of a highly enantioselective net [4 + 3] cycloaddition to afford 1,5-benzothiazepines by utilizing α,β-unsaturated acylammonium intermediates generated by a chiral isothiourea catalyst.^32^

The continued interest in diversity-oriented synthesis (DOS) to access structurally complex and diverse small-molecule libraries^33^ is premised on its value for drug discovery,^34–36^ chemical genetics^37^ and identification of small-molecule modulators of challenging biological targets.^38,39^ In particular, synthetic methods that rapidly generate stereochemical complexity^40,41^ are important for drug lead discovery and a recent success for drug development is exemplified by the antimalarial agent, NITD609, currently in phase IIa clinical trials.^42,43^ Furthermore, natural product-inspired libraries are beginning to provide good success rates in identifying more potent and drug-like molecules.^44,45^ As described herein, the use of a chiral isothiourea catalyst capable of exercising high relative and absolute stereocontrol in DAL organocascade processes in combination with a Brønsted base, which was found to profoundly impact *endo*/*exo* selectivity, points to the potential of accessing all possible diastereomers through a DA cycloaddition which may find utility in DOS.^24^

Our interest in understanding the origins of Lewis base catalyst-Brønsted base synergy in DAL organocascades led us to undertake both experimental and computational studies. Most known asymmetric reactions possess temperature-dependent diastereodifferentiation and thus are performed at low temperatures to maximize the impact of ΔΔ*H* ^‡^ induced by steric repulsion, structural strain, or electronic interactions in the transition state. From a synthetic perspective, entropy-controlled asymmetric transformations with sufficient ΔΔ*S* ^‡^ are preferable due to their temperature independence, however entropy-driven, diastereoselective reactions are rare.^46–52^

## Results and discussion

### Stereodivergent Diels–Alder-lactonization organocascades

**Fig. 3:**
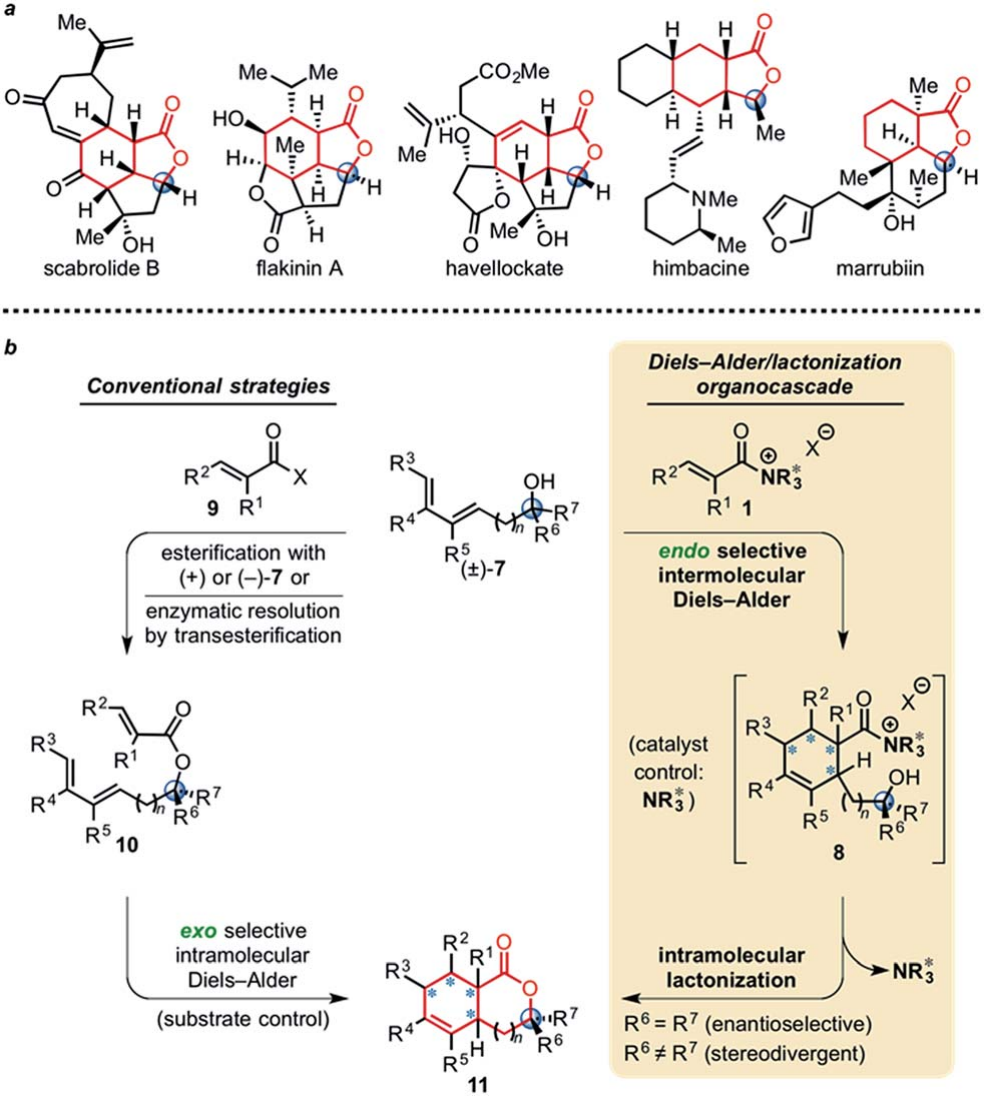
(a) Selected structures of naturally occurring and biologically active terpenoids containing γ-substituted, *cis*-fused bicyclic-γ-lactones. (b) Comparison of conventional strategies employing optically active dienyl alcohols **7** or enzymatic resolution and the described DAL organocascade to access bicyclic γ- and δ-lactones **11** (*n* = 0, 1). Use of racemic dienes (±)-**7** bearing a pendant carbinol stereocenter (blue circle) enables a stereodivergent organocascade that introduces up to four additional stereocenters (blue asterisk) through catalyst control independent of the resident carbinol stereocenter.

We previously reported a single example of a stereodivergent DAL organocascade with a cyclohexyl hydroxydiene (±)-**4** (see Fig. 1b). To further explore the scope of stereodivergent DAL processes, we targeted additional complex, bicyclic γ-lactones under conditions previously described.^24^ These are ubiquitous structural motifs found in bioactive natural products that could be accessed in a single operation through the DAL cascade (Fig. 3a). Previous strategies toward this class of optically active complex, bicyclic γ-lactones include *exo*-selective intramolecular DA cycloadditions with optically active dienyl esters **10**,^53^ for example those obtained by esterification with optically active dienyl alcohols **7** or through enzymatic resolution of racemic esters **10** by transesterification (Fig. 3b).^54^ We envisioned the use of racemic dienes bearing a pendant, stereogenic carbinol (*e.g* (±)-**7**, R_6_ ≠ R_7_, blue circles) for a terminating lactonization step for stereodivergent DAL cascades introducing up to four additional stereocenters through catalyst control independent of the existing carbinol center. The lactonization step would deliver diastereomeric cycloadducts **11** with distinct topologies that could facilitate separation (Fig. 3).

Building on our previous studies of stereodivergent DAL organocascades,^24^ we investigated additional racemic silyloxydienes (±)-**13a–c** and the previously described diene (±)-**4** (ref. 24) bearing pendant, secondary alcohols (Table 1). Ethyl fumaroyl chloride (**5**) was employed as dienophile given its higher reactivity and (*S*)-(–)-BTM was used as the chiral Lewis base. Use of silyloxydiene (±)-**13a** delivered a readily separable 1.5 : 1 diastereomeric mixture of bicyclic γ-lactones (–)-**14a** (99% ee) and (+)-**14a′** (98% ee) in 48% and 31% yield, respectively (Table 1, entry 1). Similarly, diene (±)-**13b** bearing a pendant, tertiary benzylic alcohol afforded a separable 1.8 : 1 diastereomeric mixture of cycloadducts (+)-**14b** (41% yield, 99% ee) and (+)-**14b′** (23% yield, 99% ee) bearing four contiguous stereocenters, including a quaternary carbon on gram-scale (entry 2).

**Table 1:** Diels–Alder-initiated, stereodivergent organocascades with racemic dienes (±)-**13a–d** providing bi- and tricyclic γ-lactones **14a–d** ^*a*^

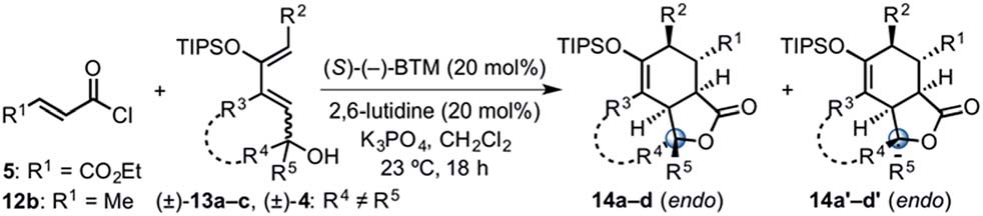			
Entry	Diene	Acid chloride	Cycloadducts % yield (*endo* : *exo*, % ee)
1	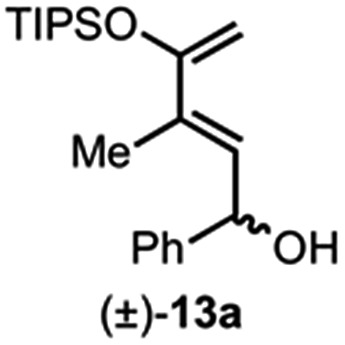	**12a**	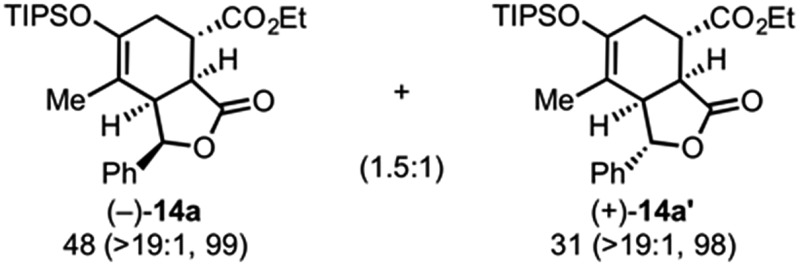
2	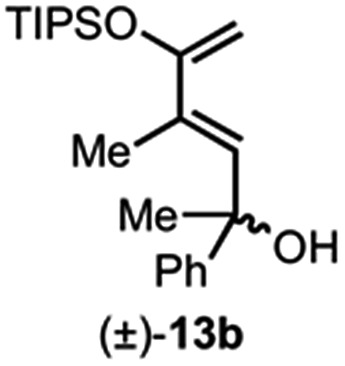	**12a**	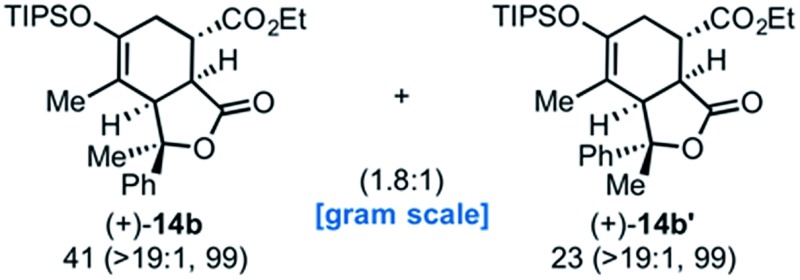
3	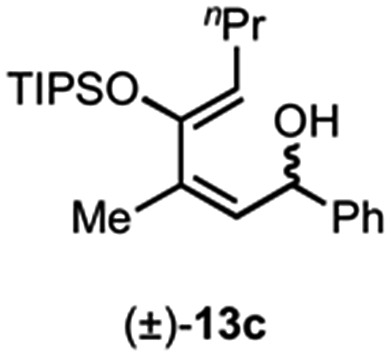	**12a**	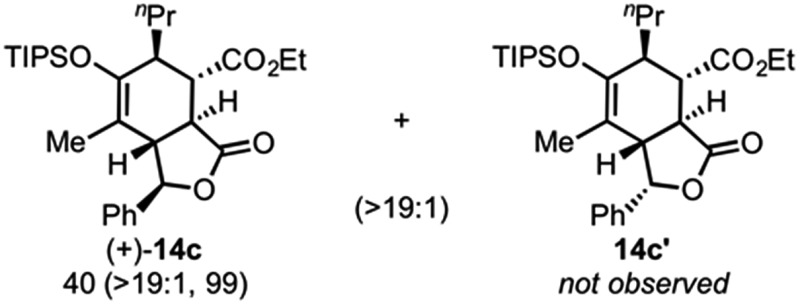
4	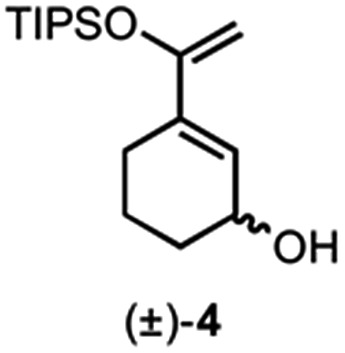	**12b**	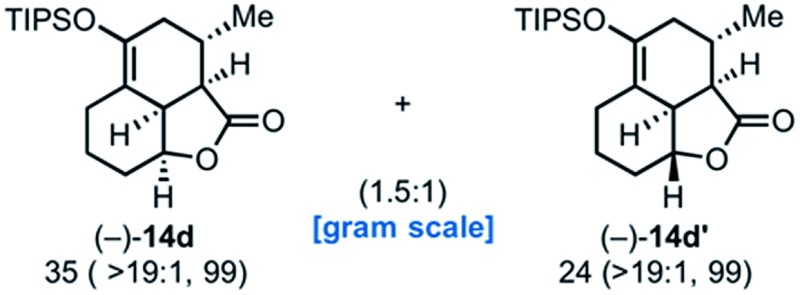

^*a*^Unless otherwise specified, all reactions were performed with diene (1.0 equiv.), acid chloride (1.5 equiv.), K_3_PO_4_ (3.0 equiv.), 2,6-lutidine (20 mol%), and (*S*)-(–)-BTM (20 mol%) at 23 °C for 18 h. Yields are based on isolated, purified cycloadducts. Enantiomeric excess was determined by chiral phase HPLC.

Use of racemic silyloxydiene (±)-**13c** possessing a (*Z*,*Z*)-configured diene provided *trans*-fused bicyclic γ-lactone (+)-**14c** as a single diastereomer with five contiguous stereocenters in 40% yield (99% ee) despite the *cis*-substituent that typically impedes effective cycloaddition (Table 1, entry 3).^55^ Presumably this diene leads to a kinetic resolution due to the presence of the *endo*-disposed phenyl substituent which may preclude terminating lactonization delivering the *exo* adduct **14c′**. To the best of our knowledge, this is the first report of a catalytic DA cycloaddition with a *cis*-substituted diene that occurs at ambient temperature (23 °C) with high enantioselectivity.^56^ We again studied the racemic cyclohexanol diene (±)-**4**, but with the less reactive commodity dienophile, crotonoyl chloride (**12b**). This DAL process gave fused, tricyclic 6,6,5-adducts on gram-scale as separable diastereomers (–)-**14d** and (–)-**14d′** in 35% (99% ee) and 24% yield (99% ee), respectively, from *trans*-β-methyl acryloyl chloride and the previously described diene (±)-**4** (ref. 24) (entry 4). The relative and absolute configuration of crystalline cycloadduct (–)-**14a** was previously determined by X-ray analysis^24^ while cycloadduct (–)-**14a′** required ring opening of γ-lactone with 4-bromobenzylamine (ESI, Fig. S2†). The relative and absolute configurations of cycloadducts **14a–d** and **14a′–d′** was assigned premised on these analogous systems in conjunction with detailed NMR analysis. In general, low yields in these stereodivergent DAL reactions were due to desilylation of dienes at ambient temperature (23 °C) upon prolonged reaction times and competitive, irreversible esterification of the tethered alcohol moiety. Importantly, subsequent intramolecular DA reactions of the resulting esters do not proceed at ambient temperature, therefore potential non-selective background processes are precluded. Sterically demanding α,β- and β,β-disubstituted acid chlorides and non-silyloxy-substituted dienes do not undergo DAL organocascades under comparable conditions (see ESI, Table S1†).

### Synthetic applications of the DAL organocascade process

**Fig. 4:**
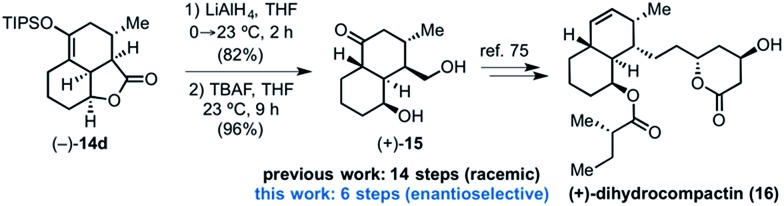
Conversion of the tricyclic γ-lactone (–)-**14d** to ketodiol (+)-**15** representing a formal synthesis of (+)-dihydrocompactin (**16**).

Toward demonstrating the utility of the DAL process, we targeted the core structure of (+)-dihydrocompactin (Fig. 4), a potent hypocholesterolemic agent first isolated by a group at Merck.^57^ This natural product, along with mevinolin, is historically significant in providing the original information for eventual development of the well-known statin drugs, lovastatin (Mevacor®) and simvastatin (Zocor®). In one approach to dihydrocompactin by Hagiwara,^58^ the bicyclic ketodiol **15** was employed in racemic form. We recognized that the tricyclic lactone (–)-**14d** could be fashioned into this precursor to dihydrocompactin with a few additional synthetic manipulations. The sequence entailed reduction of lactone (–)-**14d** with LiAlH_4_ to the corresponding diol followed by desilylation of the silyl enol ether with tetrabutylammonium fluoride (TBAF). Under the basic conditions of the deprotection, thermodynamic equilibration occurred to provide the desired *trans*-decalin of the targeted ketodiol (+)-**15** which previously served as a precursor to dihydrocompactin.^58^

### Effects of Brønsted base on acylammonium salt formation and initial Diels–Alder step

**Fig. 5:**
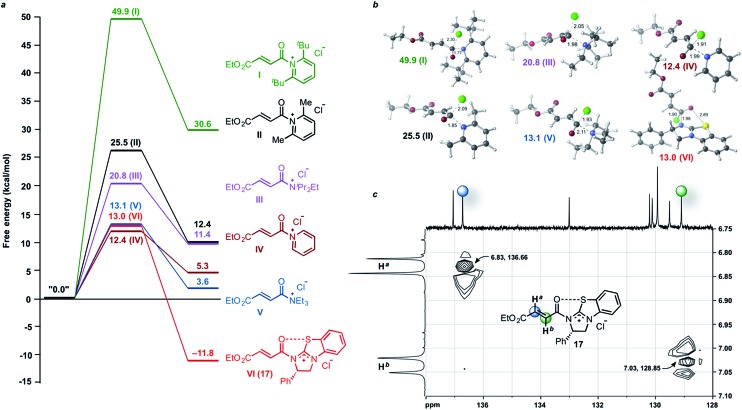
(a) Comparison of free energies for acylammonium salt formation (I, DTBP; II, 2,6-lutidine; III, Hünig's base; IV, Et_3_N; V, (*S*)-(–)-BTM; VI, pyridine) between ethyl fumaroyl chloride (**5**) and various achiral amines and (*S*)-(–)-BTM. Free energies of transition state structures (TSSs) and products (shown in kcal mol^–1^ relative to free energies of separated reactants) computed using SMD(DCM)-M06-2X/6-31G(d). (b) Calculated TSSs (I–VI) for the formation of acylammonium salts with various Brønsted bases optimized at the M06-2X/6-31G(d) level with an implicit solvent model [SMD (dichloromethane)]. Selected bond distances are shown (Å). (c) Section of the ^1^H–^13^C gHMQC NMR spectrum of the acylammonium salt **17** in CD_2_Cl_2_ formed from a 1 : 1 mixture of (*S*)-(–)-BTM and ethyl fumaroyl chloride (**5**).

During our previous screening of Brønsted bases, we determined that certain tertiary amines exert a profound effect on the *endo*/*exo* selectivities of the DAL process. We reasoned that the Brønsted base may be playing a dual role of deprotonation of the pendant alcohol during lactonization through initial hydrogen-bonding and also ensuring the free-base form of the catalyst. However, certain tertiary amine Brønsted bases can also act as Lewis base catalysts leading to racemic product.^59^ Thus, computational studies were pursued to determine the extent to which various Brønsted bases could compete effectively with the optically active Lewis base leading to achiral acylammonium dienophiles and nonselective DAL processes. Results from quantum chemical calculations (see ESI† for details) of acylammonium salt formation between ethyl fumaroyl chloride (**5**) and various tertiary amine Brønsted bases indicate that only pyridine and Et_3_N, with predicted free energy barriers of 12.4 and 13.1 kcal mol^–1^, respectively, would plausibly compete with BTM (barrier of 13.0 kcal mol^–1^) (Fig. 5a). However, both reactions are endergonic, with energy barriers for reversion to the corresponding acid chlorides of only 7.1–9.5 kcal mol^–1^, and thus are readily reversible.^24,60^

With these computational results in hand, we next sought to provide experimental support through a brief screen of selected Brønsted bases employing diene **13d** ^23^ and acid chloride **5** with (*S*)-(–)-BTM as catalyst (Table 2). Triethylamine, pyridine and even Hünig's base (^i^Pr_2_NEt) led to greatly reduced enantioselectivity (60–85% ee) compared to 2,6-lutidine (99% ee) and 2,6-di-*tert*-butylpyridine (DTBP, 99% ee). In that we predict that a nonselective background pathway involving achiral acylammonium salts would not compete effectively with the enantioselective DAL process, we suspect that the reduced enantioselectivity is due to the hydrogen-bonding effects discussed below. Interestingly, acylammonium salt formation through chloride ion exchange reactions with both (*S*)-(–)-BTM and tertiary-amine Brønsted bases were predicted to proceed by attack at the carbonyl carbon and displacement of chloride without formation of a tetrahedral intermediate as a minimum (Fig. 5b, I–VI). Instead, these reactions appear to proceed by a concerted S_N_2-type mechanism, consistent with previous computational studies indicating that some reaction coordinates for substitution of acyl derivatives lack minima corresponding to the tetrahedral intermediates expected for an addition–elimination process.^61–63^

**Table 2:** Brønsted base screen for DAL with diene **13d**, acid chloride **5**, and (*S*)-(–)-BTM as catalyst

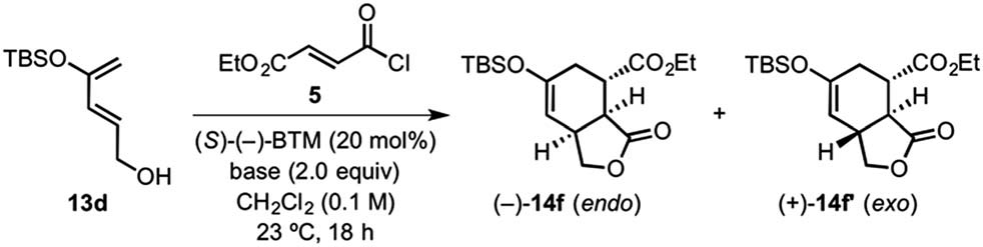				
Entry	Base	de^*a*^ (%)	ee^*b*^ ^,^ ^*c*^ (%)	Conversion^*d*^ (%)
1	Et_3_N	71	60	>95(60)
2	Pyridine	76	85	>95(46)
3	Hünig's base [EtN(^i^Pr)_2_]	71	65	>95(55)
4	2,6-Lutidine	60	99	>95(68)
5	2,6-Di-*tert*-butylpyridine [DTBP]	95	99	>95(43)

^*a*^Determined by ^1^H NMR (500 MHz) analysis of crude reaction mixtures.

^*b*^Determined by chiral HPLC analysis (see ESI for details).

^*c*^Enantiomeric excess of the major (–)-**14f** (*endo*) diastereomer (ee values for the *exo* diastereomer were similar).

^*d*^Yields in parentheses refer to isolated, purified yields of cycloadducts.

We postulated previously that activation of the DA reaction upon acylammonium salt formation would originate from inductive effects propagated through the σ-framework, which could ultimately be revealed through reduced electron density at the β-carbon.^64^ We therefore performed a ^1^H–^13^C gHMQC experiment and measured the ^13^C NMR chemical shifts in CD_2_Cl_2_ at 23 °C for the acylammonium salt **17** formed through chloride ion exchange reaction of the acid chloride **5** with the Lewis base, (*S*)-(–)-BTM (Fig. 5c). However, no significant change in the chemical shift of the β-carbon of acylammonium **17** (*δ* 136.7 ppm) was observed compared to the acid chloride **5** (*δ* 136.8 ppm). Comparison of the chemical shift of the carbonyl carbons revealed only a slight upfield shift in acylammonium salt **17** (*δ* 163.6 ppm) compared to the acid chloride **5** (*δ* 164.1 ppm) possibly due to the close proximity of the phenyl ring of (*S*)-(–)-BTM to the carbonyl carbon.

Thus, isothiourea-catalyzed acylammonium formation may not lead to dramatic LUMO-lowering activation, but rather a significant decrease in the rate of intermolecular, nucleophilic substitution at the carbonyl carbon by the pendant alcohol of the diene, enabling the DA-initiated organocascade. In addition, a comparison of energy barriers for the initial DA step between the BTM-derived dienophile and various achiral Lewis base-derived dienophiles (*e.g.* pyridine, Et_3_N, 2,6-lutidine) indicates a lower barrier for the enantioselective DA cycloaddition compared to the background nonselective DA (ΔΔ*G* ^‡^ ∼ 13–43 kcal mol^–1^) and is consistent with the generally observed excellent enantioselectivities in the DAL process (for computed energies see ESI (Comp studies), Table S3†).

### Effects of Brønsted base on the origins of the diastereoselectivity in the Diels–Alder-initiated cascades

**Fig. 6:**
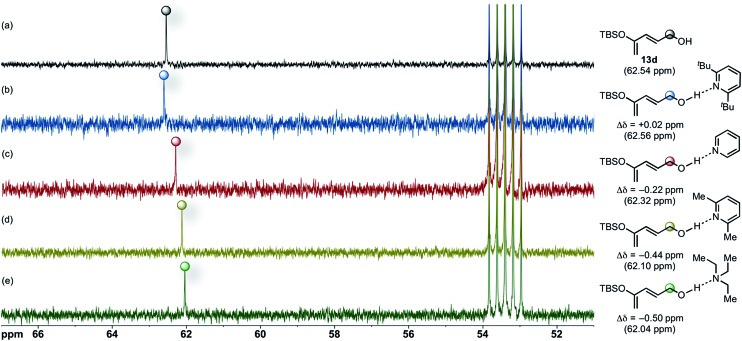
Partial ^13^C NMR (500 MHz, *δ* 51–67 ppm) spectra in CD_2_Cl_2_ at 23 °C of an equimolar mixture of diene **13d** (a) and DTBP (b), pyridine (c), 2,6-lutidine (d), and Et_3_N (e).

Based on the aforementioned experimental and computational results suggesting that bulky Brønsted bases could not effectively compete with BTM as Lewis base promoter during the initial DA cycloaddition, we considered other roles that the Brønsted base could be serving during the cycloaddition. On the basis of previous studies of alcohol–amine complexes,^65^ we envisaged a hydrogen-bonded complex between these amino Brønsted bases and the alcohol moiety of the dienes employed. We postulated that evidence for such an interaction might be detected by ^13^C NMR through increased electron density at the carbinol-carbon of diene due to inductive effects propagated through the σ-framework. We performed standard ^13^C NMR (500 MHz) experiments in CD_2_Cl_2_ (0.1 M) at 23 °C and measured the changes in chemical shifts of the carbinol carbon of diene **13d** (Fig. 6a) upon addition of an equimolar amount of Brønsted bases. Addition of 1.0 equiv. of DTBP to diene **13d** leads to negligible change in the ^13^C NMR spectrum (Fig. 6b) indicating that under these conditions a hydrogen-bonded complex does not form or is highly reversible favouring a non-hydrogen bonded complex.

Furthermore, the chemical shift (62.56 ppm) of the carbinol-carbon (Δ*δ* = +0.02) of diene **13d** (62.54 ppm) is relatively unchanged upon addition of DTBP (Fig. 6a *vs.* b). The inability of the nitrogen atom in DTBP to participate in hydrogen-bonding is anticipated due to steric hindrance^66^ imposed by the adjacent *tert*-butyl substituents, and this is proposed to be largely responsible for its low relative basicity.^67^

In contrast, the ^13^C NMR spectrum of an equimolar mixture of diene **13d** and pyridine shows a pronounced upfield chemical shift (Δ*δ* = –0.22 ppm) for the carbinol carbon, supporting formation of a hydrogen-bonded complex (Fig. 6c). The extent of complexation was particularly evident in the ^13^C NMR spectrum of diene **13d** with added 2,6-lutidine resulting in a significant upfield chemical shift (Δ*δ* = –0.44 ppm) of the carbinol carbon (Fig. 6d) and likewise with Et_3_N (Δ*δ* = –0.50 ppm). These upfield shifts qualitatively correlate with hydrogen-bond strength and Brønsted basicity. In particular, p*K*_a_ values (in DMSO) and Δ*δ* differences follow the order: DTBP (0.9,^68–70^ +0.02) < pyridine (3.4,^70^ –0.22) < 2,6-lutidine (4.46,^69^ –0.44) < Et_3_N (9.0,^68^ –0.50).

**Fig. 7:**
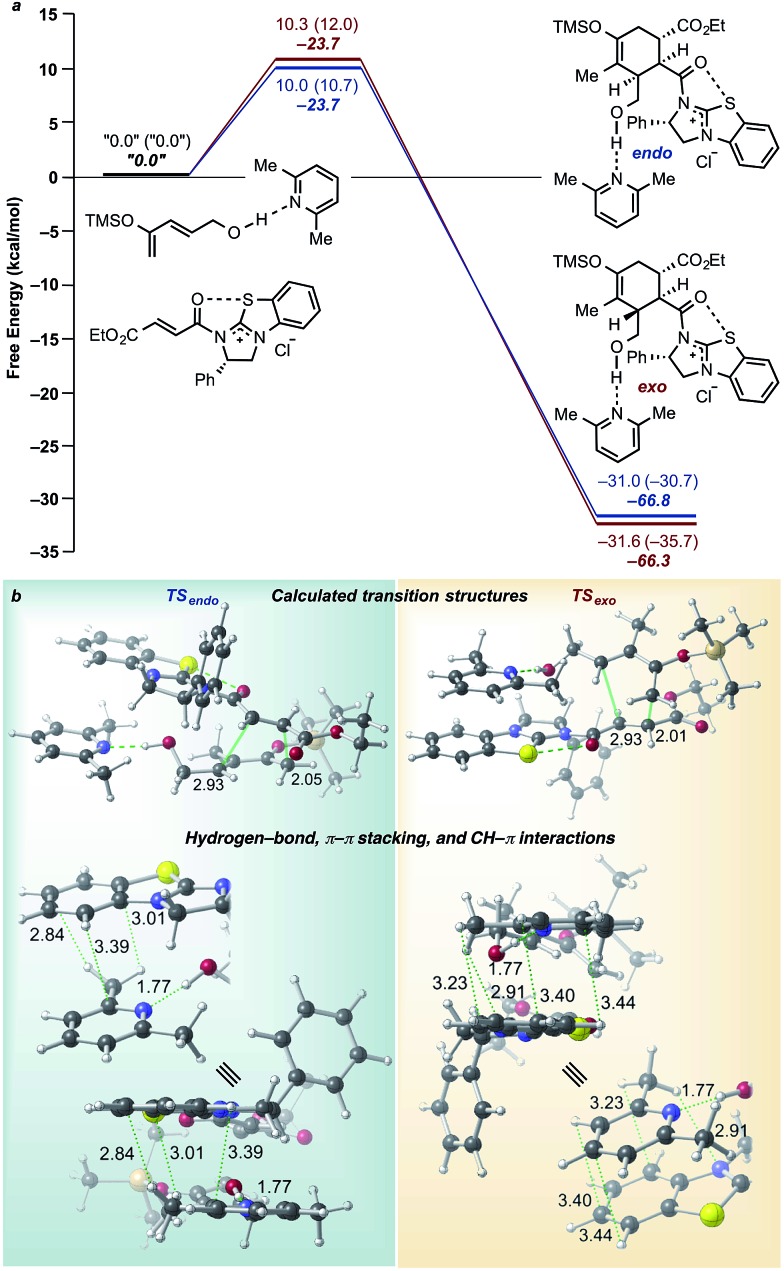
(a) Free energies (normal text) and enthalpies (bold, italic) calculated at SMD(DCM)-M06-2X/6-31G(d) are in kcal mol^–1^ relative to separated reactant species shown. Values inside parentheses represent free energies without explicit base. An explicit base (2,6-lutidine) was modelled to study stereoelectronic effects on TSSs involved in the initial DA cycloaddition. (b) Optimized TSSs leading to *endo* and *exo* cycloadducts showing π–π stacking and CH–π interactions between the BTM-bound acylammonium salt and the hydrogen-bonded Brønsted base–diene complex. Selected bond distances are shown (Å).

Based on the potential that hydrogen-bonded complexes may participate in this organocascade process, TSSs for the DA cycloaddition were recalculated with 2,6-lutidine complexed to silyloxydiene **13d** (Fig. 7). A manual conformational search sampling numerous possible orientations of 2,6-lutidine resulted in the lowest energy *endo* and *exo* TSS conformations as shown in Fig. 7b. As indicated, 2,6-lutidine can indeed participate in hydrogen bonding with the terminal alcohol of the diene and simultaneously engage in CH–π and π–π stacking interactions with the benzotetramisole moiety of the BTM-bound acylammonium salt. These interactions lower the free energy barrier for the *exo* cycloaddition (12.0–10.3 kcal mol^–1^)^71,72^ to a greater extent than the *endo* cycloaddition (10.7–10.0 kcal mol^–1^), thereby leading to the prediction that such complexation would reduce diastereoselectivity as observed (95 *vs.* 60% de, DTBP *vs.* 2,6-lutidine, Table 2). Differences in relative stabilization of the DA TSSs by 2,6-lutidine are associated with the respective orientations of the BTM-bound acylammonium salt, 2,6-lutidine, and the terminal alcohol of the diene. In the *exo* TSS, 2,6-lutidine appears to be unable to maximize hydrogen-bonding, CH–π, and π–π stacking interactions simultaneously. The BTM-bound acylammonium salt and 2,6-lutidine are involved in a displaced π–π stacking interaction, whereas, in the *exo* TSS, π–π stacking of the BTM-bound acylammonium salt and 2,6-lutidine occurs over a larger area while allowing the base to maintain a hydrogen-bonding interaction with the terminal alcohol of the diene. While these complexation geometries bear on future design, the extremely similar predicted free energy barriers for *endo* and *exo* cycloadducts is not consistent with the magnitude of the experimentally observed diastereoselectivity (60% de, *cf.* Table 2), which prompted further investigations of the origins of this apparent discrepancy.

### Entropy-controlled diastereodifferentiation in Diels–Alder-initiated cascades

**Fig. 8:**
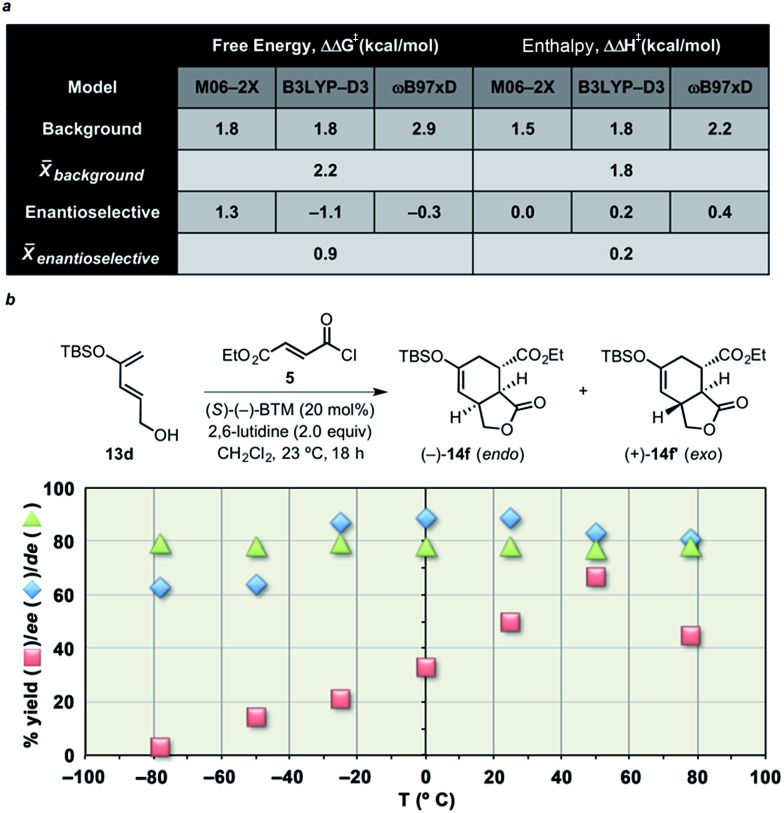
(a) Differences in free energies and enthalpies of TSSs from the non-selective background and enantioselective DA cycloadditions computed using multiple model chemistries and the 6-31G(d) basis set (see ESI† (Comp studies) for details). Energies shown in kcal mol^–1^ relative to separated reactants. (b) Plots of yield and enantiomeric and diastereomeric excess as a function of temperature for DAL reactions employing diene **13d** and acid chloride **5**. Enantiomeric excess was determined by chiral-phase HPLC and is only shown for the major (*endo*) diastereomer (ee values for the *exo* diastereomer were similar).

While the predicted diastereoselectivity for the Brønsted base-free DA cycloaddition was found not to depend greatly on Δ*S* corrections, the BTM-promoted cycloaddition was found, based on calculations, to change significantly upon consideration of Δ*H versus* Δ*G* (Fig. 8a). Unfortunately, the direction and magnitude of this change varied with the level of theory used, likely a result of known difficulties in accurately computing entropy corrections^73^ and dispersion interactions.^74^ Nevertheless, these results led us to consider the possibility that the diastereoselectivity was not controlled by enthalpy (*i.e.* predicted ΔΔ*H* ^‡^s are insignificant), but rather by entropy.^46–52^

We therefore investigated the enantio- and diastereoselectivity of the DAL organocascade of silyloxydiene **13d** with ethyl fumaroyl chloride (**5**) over a wide range of temperatures (Fig. 8b). Plots of yield and enantio- and diastereomeric excess as a function of temperature are depicted in Fig. 8b. The data indicates the dominance of the background racemic reaction at the extremities of temperature, likely due to inefficient acylammonium formation at temperatures below –20 °C and adequately competent background reaction above +50 °C. In the case of diastereoselectivity in the temperature range –78 to +80 °C, the data clearly shows an invariance in diastereoselectivity. Reactions were analyzed after 18 h, and the relative *endo*/*exo* ratios and enantioselectivity were concurrently determined by chiral-phase HPLC of the crude reaction mixtures. The chemical yields were determined after flash chromatography on silica gel. The “flat” temperature dependency observed in this temperature range (Δ*T* ≈ 160 °C) provides evidence that the diastereoselectivity of the enantioselective DA cycloaddition (with or without modelled Brønsted base) is predominantly controlled by the differential activation entropy ΔΔ*S* ^‡^ rather than ΔΔ*H* ^‡^.

### Computed potential energy surface for the lactonization step of the DAL

**Fig. 9:**
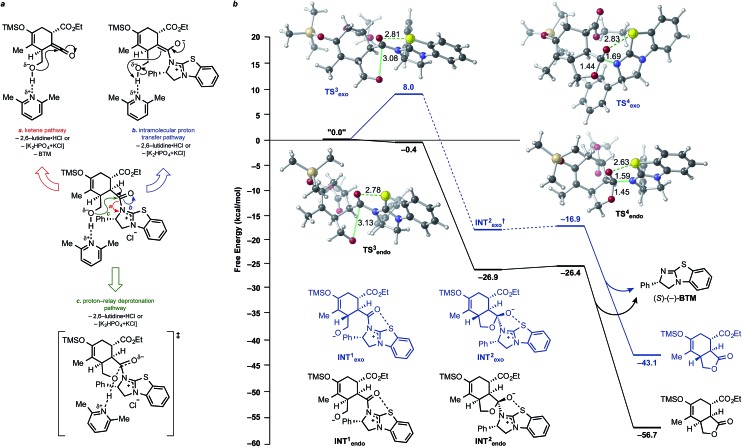
(a) Possible lactonization pathways for the DAL organocascade process. (b) Computed potential energy surface for the lactonization step of the DAL. Free energies (kcal mol^–1^) were calculated employing SMD(DCM)-M06-2X/6-31G(d). Intermediates and optimized TSSs for both *endo* (black) and *exo* (blue) pathways are shown. Selected bond distances are shown (Å).

Plausible lactonization pathways are depicted in Fig. 9a. The results of our computations show that initial deprotonation of the alcohol leads, not surprisingly to attack on the acylammonium carbonyl with a low barrier (no barrier for the *endo* case; Fig. 9b). Again, well-defined tetrahedral intermediates are not found (a minimum for the *endo* case was found but it has a negligible barrier for fragmentation; Fig. 9b), consistent with a concerted but highly asynchronous lactonization reaction.^75–77^ The lactonization pathway involving attack of the neutral alcohol on the acylammonium salt intermediate showed this path is unlikely given the computed free energy barrier of ∼26 kcal mol^–1^; attempts to model this stepwise nucleophilic addition of the alcohol and subsequent deprotonation by 2,6-lutidine proved unsuccessful. Due to the difficulty of accurately modelling deprotonation/protonation steps,^73^ especially considering the presence of base, shuttle base, and solvent in solution, full pathways proposed in Fig. 9a were not computed; instead, ketene and enolate intermediates resulting from proposed pathways were optimized and their relative energies compared. The ketene intermediate (+ BTM) was found to be ∼17 kcal mol^–1^ higher in energy than the initial DA adduct (pathway a), making this pathway energetically feasible, with deprotonation and ketene formation with concomitant catalyst regeneration likely occurring in a concerted process (see ESI, Scheme S3†). The enolate intermediate was found to be ∼7 kcal mol^–1^ lower in energy than the deprotonated alcohol of the DA adduct (INT1, Fig. 9b, pathway b), making enolate formation also energetically feasible. However, both the ketene pathway and intramolecular proton transfer pathway would result in scrambling of the stereochemistry, which was not experimentally observed; therefore, it is likely that deprotonation and lactone formation occur in a coordinated process (pathway c).

### Switching diastereoselection: toward achieving the full complement of possible stereoisomeric products in a Diels–Alder cycloaddition

**Fig. 10:**
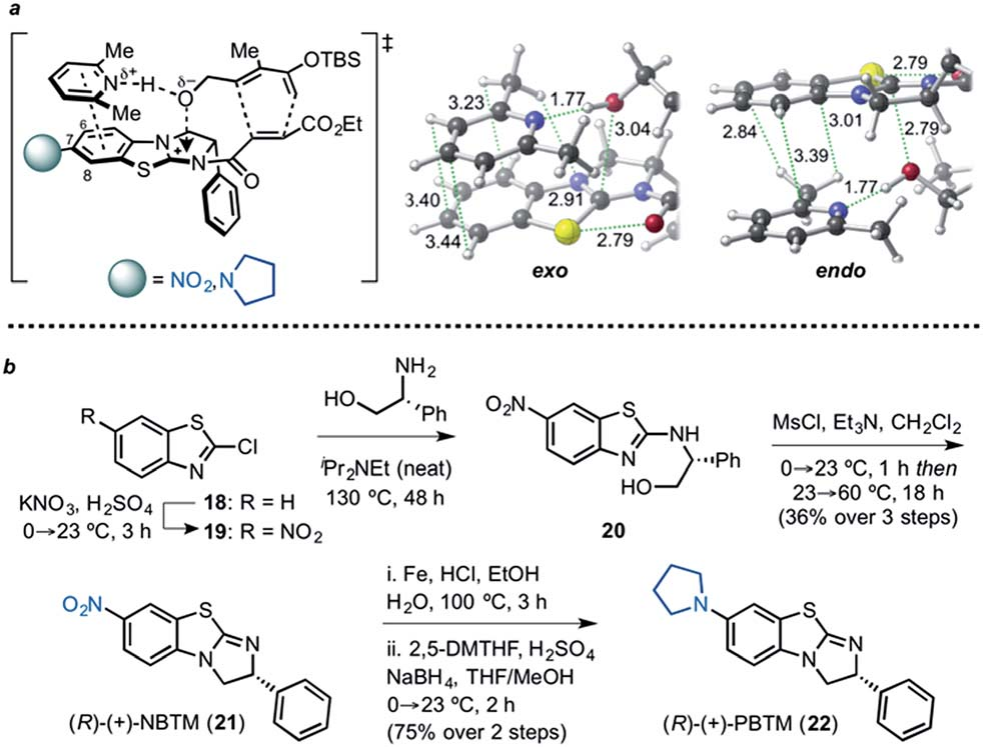
(a) Catalysts designed to potentially alter diastereoselection in DAL organocascades, and TSSs depicting potential stabilization by n → π* interaction optimized with SMD(DCM)-M06-2X/6-31G(d), modelled with an explicit Brønsted base (2,6-lutidine). Selected bond distances are shown (Å). (b) Preparative synthesis of electronically tuned BTM-based catalysts.

Based upon aforementioned computations suggesting selective stabilization of the *exo* TSS by CH–π and π–π stacking interactions between the Brønsted and Lewis bases employed, we reasoned that installation of an electron-withdrawing substituent at the C7 position of the benzothiazole moiety of BTM might enhance these interactions.^78,79^ Conversely, an electron-donating substituent also would be expected to impact the interaction, thus altering the *endo*/*exo* selectivity. In addition, a closer look at the optimized TSSs revealed the potential for an n → π* interaction^80–83^ between the hydroxyl group and imidazolium cation in both *exo* (3.04 Å) and *endo* (2.79 Å) TSSs (Fig. 10a).^84–86^

We chose to examine a highly electron-withdrawing nitro group and an electron-donating pyrrolidinyl group, reminiscent of 4-pyrrolidinopyridine (4-PPY) (Fig. 10). The synthesis of these catalysts commenced with nitration of 2-chlorobenzothiazole (**18**) with a mixture of concentrated sulfuric acid and fuming nitric acid to provide the nitrothiazole **19**,^87^ which was used directly in the next step without purification. Employing Smith's recently improved, scalable two-step protocol,^88^ nitrothiazole **20** was subjected to condensation with (*R*)-phenylglycinol in neat diisopropylethylamine to furnish alcohol **20**, which was directly treated with methanesulfonyl chloride. Refluxing a dichloromethane solution of the resultant mesylate in the presence of triethylamine and methanol overnight provided 7-nitrobenzotetramisole, (*R*)-(+)-NBTM in 36% yield (over 3 steps). The nitro group was reduced with iron powder in ethanol to afford the corresponding amino benzene derivative, which upon treatment with 2,5-dimethoxytetrahydrofuran (DMTHF) and sodium borohydride underwent reductive amination to provide the desired pyrrolidinylbenzotetramisole, (*R*)-(+)-PBTM in 75% yield (over 2 steps).

Given the ability to access both *endo* and *exo* transition states using particular Brønsted bases, we studied the possibility of a fully stereodivergent version of the DAL with a racemic diene to access all possible stereoisomers of a particular family of cycloadducts. Employing racemic silyloxydiene (±)-**13e** bearing a pendant secondary benzylic alcohol, ethyl fumaroyl chloride (**5**), and (*S*)-(–)-BTM (20 mol%) and 2,6-lutidine (2.0 equiv.), four separable diastereomers (–)-**14** (27% yield, 98% ee), (+)-**14′** (22% yield, 99% ee), (+)-**14′′** (25% yield, 99% ee) and (–)-**14′′′** (18% yield, 97% ee) in 92% combined yield were produced (entry 1, Table 3). This reaction could be performed on a preparative scale with only 10 mol% (*S*)-(–)-BTM providing 76% yield of these diastereomers.

**Table 3:** Tunable diastereoselectivity in accessing a stereoisomeric family of bicyclic-γ-lactones through chiral Lewis base catalyst and Brønsted base permutations in the DAL organocascade

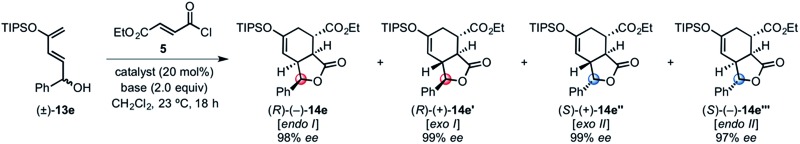						
Entry	Catalyst	Base	(*R*)-(–)-**14e** ^*a*^ (%)	(*R*)-(+)-**14e′** ^*a*^ (%)	(*S*)-(+)-**14e′′** ^*a*^ (%)	(*S*)-(–)-**14e′′′** ^*a*^ (%)
1	**A**	**I**	27	22	25	18
2	**A**	**II**	24	—	23	15
3	**A**	**III**	27	—	—	21
4	**A**	**IV**	29	—	20	—
5	**B**	**V**	—	—	26	19
6	**B**	**IV**	—	—	18	—
7	**C**	**IV**	22	—	—	—
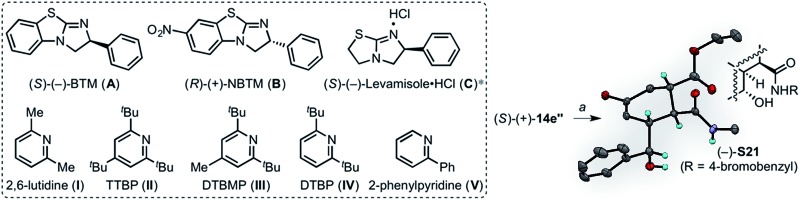						

^*a*^Yields and diastereomeric ratios are based on isolated, purified cycloadducts. Enantiomeric excess was determined by chiral phase-HPLC. *Employed in free-base form. Inset is a single crystal X-ray structure in ORTEP format of adduct (*R*)-(+)-**14′′** following ring opening with 4-bromobenzylamine (50% probability; TIPS and 4-bromobenzyl groups are removed for clarity, see ESI Fig. S1). ^*a*^4-BrC_6_H_4_CH_2_NH_2_, THF, 23 °C, 36 h (46%).

The use of commercially available pyridines as Brønsted bases bearing electron-withdrawing substituents, such as 2- and 3-bromopyridine, and 2,6-dibromopyridine, were ineffective in delivering cycloadducts, presumably due to their reduced basicity. In contrast, pyridines with electron-donating groups, such as 3- and 4-methoxypyridine delivered cycloadducts, but led to negligible changes in diastereoselectivity. On the other hand, 2,4,6-tri-*tert*-butylpyridine (TTBP) selectively suppressed formation of the *exo* I diastereomer, (+)-**14′** (entry 2, Table 3), whereas 2,6-di-*tert*-butyl-4-methylpyridine (DTBMP) deterred formation of both *exo* I and II diastereomers, (+)-**14′** and (+)-**14′′** (entry 3, Table 3). In addition, 2,6-di-*tert*-butylpyridine (DTBP) induced preferential formation of both *endo* I and *exo* II diastereomers, (–)-**14** and (+)-**14′′**, in 29% and 20% yields, respectively (entry 4, Table 3).

We next studied the BTM-based catalysts in conjunction with various substituted pyridine bases. Accordingly, the highly nucleophilic, electron-rich (*R*)-(+)-PBTM catalyst accelerated the formation of the corresponding cycloadducts, likely due to faster formation of the resultant acylammonium salt, however without noticeable deviations in diastereoselection. In contrast, use of the (*R*)-(+)-NBTM catalyst and 2-phenylpyridine selectively impeded the reactivity of the (*R*)-enantiomer of (±)-**14**, consequently resulting in formation of both *exo* II and *endo* II, diastereomers, (+)-**14′′** and (–)-**14′′′**, in 26% and 19% yields, respectively (entry 5, Table 3).

A single *exo* II diastereomer, (+)-**14′′**, was obtained in 18% yield (99% ee) through use of (*R*)-(+)-NBTM and 2,6-di-*tert*-butylpyridine (entry 6, Table 3). In addition, a single *endo* I diastereomer, (–)-**14**, was obtained in 22% yield (99% ee) by a combination of 2,6-di-*tert*-butylpyridine with the free-base form of levamisole hydrochloride (entry 7, Table 3). The relative and absolute configuration of a ring-opened derivative of cycloadduct (+)-**14′′** was verified by X-ray analysis (see ESI, Fig. S1†) enabling assignment of all cycloadducts (–)-**14**, (+)-**14′** and (–)-**14′′′** through comparative 2-D NMR analysis. It should be noted that use of (*Z*)-(±)-**13e** and the optical antipode of the catalyst would theoretically enable access to the remaining diastereomeric and enantiomeric members (16 total) of this family of cycloadducts. While varying the chiral Lewis base catalyst and Brønsted base enabled control of DA diastereomers produced, the yields are not yet serviceable when single diastereomers are obtained (entries 6 and 7; Table 3). Indeed, variations in diastereoselectivity could be a reflection of the rate of the subsequent lactonization rather than inherent diastereoselectivity of the DA cycloaddition. However, these preliminary results point to the possibility of developing a fully stereodivergent DAL organocascade that can access all possible stereoisomers of a given family through variations of Lewis and Brønsted bases employed.

## Conclusion

Further examples, applications, and mechanistic studies of the Diels–Alder-lactonization organocascade employing α,β-unsaturated acylammonium salts are presented. Factors affecting the selectivity of stereodivergent, Diels–Alder-initiated organocascades were investigated systematically with a view to understanding, predicting, and tuning the stereochemical outcome. An evaluation of various experimental parameters, guided by the results of computational studies of the DAL process, was undertaken in order to derive a more detailed understanding of the origins of selectivity. In addition, the substrate scope of the stereodivergent organocascade was extended to tethered secondary and tertiary racemic alcohols leading to the corresponding optically active γ-substituted *cis*- and *trans*-fused bicyclic γ-lactones in good yields and with excellent enantiocontrol. The combined experimental and theoretical results described herein allow a detailed picture of the full catalytic cycle of the DAL process to emerge. While the described organocascade demonstrates reasonable scope, it currently has limitations. *e.g.* the type of dienes that participate in cycloaddition, typically requiring more electron rich dienes.

The utility of this methodology was showcased through the formal synthesis of a member of the fungus-derived and widely marketed statin drugs, (+)-dihydrocompactin.

Computational studies indicate that benzotetramisole-derived acylammonium formation proceeds by an exergonic, concerted S_N_2-type mechanism and provided insights into the role of Brønsted base in these cycloadditions. These studies revealed the potential of n → π*, CH–π and π–π stacking inter-actions in controlling selectivity, and point to the possibility of a rare entropy-controlled stereoselectivity. The experimentally observed temperature independence in the studied range of –78 to +80 °C supports the idea that the diastereoselectivity of these cycloadditions is indeed predominantly controlled by the differential activation entropy, ΔΔ*S* ^‡^.

Lastly, we documented the potential of developing a fully stereodivergent DA organocascade that could enable access to all stereoisomeric members of a given family of cycloadducts through judicious choice of Lewis and Brønsted bases. To date, this goal has only been reached for a small set of reactions and not for the DA process. Continued studies directed toward understanding the subtleties of the DAL organocascade process and applications of this process toward bioactive natural product synthesis are ongoing in our laboratories.
