# Supplementary material for: Stereodivergent, Diels–Alder-initiated organocascades employing α,β-unsaturated acylammonium salts: scope, mechanism, and application
Source: Chem Sci. 2016 Oct 21;8(2):1511–24. doi: 10.1039/c6sc04273b (PMC5460596; doi:10.1039/c6sc04273b)
Supplement: Supplementary file 1 [file SC-008-C6SC04273B-s001.pdf]

Electronic Supplementary Material (ESI) for Chemical Science.
This journal is © The Royal Society of Chemistry 2016

Stereodivergent, Diels-Alder-Initiated Organocascades Employing α,β–Unsaturated Acylammonium Salts: Scope, Mechanism, and Application

Mikail E. Abbasov,† Brandi M. Hudson,‡ Dean J. Tantillo‡,* and Daniel Romo†,*

†Department of Chemistry and Biochemistry, Baylor University, One Bear Place 97348, Waco, Texas 76798, United States

‡Department of Chemistry, University of California–Davis, One Shields Avenue, Davis, California 95616, United States

<u>**Table of Contents**</u>

• General procedures and abbreviation list S2–S3
• Experimental procedures S4–S28
  • Preparation of compounds **S3**, **S4**, **S6**, **S9**, **S10**, (±)-**13a**, (±)-**13b**, (±)-**13c**, (±)-**13e**
  • Representative procedure for the stereodivergent DAL process as described for bicyclic •γ-lactones (–)-**14a** and (+)-**14a'**
  • Representative examples of unsuccessful DAL processes (**Table S1**)
  • Synthetic applications of γ-lactone (–)-**14d**
  • Synthesis of the isothiourea catalysts (*R*)-(+)-**NBTM** and (*R*)-(+)-**PBTM**
  • Representative procedure for the enantioselective and stereodivergent DAL process leading to a full stereoisomeric complement as described for bicyclic γ-lactones (–)-**14e**, (+)-**14e'**, (+)-**14e''** and (–)-**14e'''**
• Single crystal X-ray structures and selected crystallographic data for amide (–)-**S20** (**Figures S1**) S29
• Chiral HPLC traces for compounds **14a-d**, **14a'-d'**, (–)-**14e**, (+)-**14'e**, (+)-**14e''** and (–)-**14e'''** (**Figures S2 and S3**) S33
• References S44

General Procedures

All non-aqueous reactions were performed under a nitrogen atmosphere in oven-dried glassware. Dichloromethane (CH_2Cl_2), tetrahydrofuran (THF), diethyl ether (Et_2O), acetonitrile (CH_3CN) and toluene (PhMe) were dried by passing through activated alumina (solvent purification system). Diisopropylethylamine ($EtN(^iPr)_2$) and triethylamine (Et_3N) were distilled from calcium hydride prior to use. Other solvents and reagents were used as received from commercially available sources. Deuterated solvents were purchased from Cambridge Isotopes and used as received. ^1H NMR spectra were measured at 500 MHz and referenced relative to residual chloroform (7.26 ppm) or benzene (7.16 ppm) and were reported in parts per million. Coupling constants (J) were reported in Hertz (Hz), with multiplicity reported following usual convention: s, singlet; d, doublet; t, triplet; q, quartet; dd, doublet of doublets; dt, doublet of triplets; dq, doublet of quartets; qd, quartet of doublets; td, triplet of doublets; tt, triplet of triplets; ddd, doublet of doublet of doublets; ddt, doublet of doublet of triplets; ddq, doublet of doublet of quartets; dddd, doublet of doublet of doublet of doublets; ddddt, doublet of doublet of doublet of doublet of triplets; ddquint, doublet of doublet of quintets; m, multiplet; br s, broad singlet. ^{13}C NMR spectra were measured at 125 MHz and referenced relative to residual chloroform (77.23 ppm) or benzene (128.06 ppm) and were reported in parts per million (ppm). Flash column chromatography was performed with 60Å Silica Gel (230-400 mesh) as stationary phase on an automated flash chromatography system (EtOAc/hexanes as eluent unless indicated otherwise). High-resolution mass spectra (ESI) were obtained through the Laboratory for Biological Mass Spectrometry (Texas A&M University). Thin Layer Chromatography (TLC) was performed using glass-backed silica gel F254 (Silicycle, 250 μm thickness). Visualization of developed plates was performed by fluorescence quenching or by treating with Seebach's[1] staining solution. *Fourier* Transform Infrared (FTIR) spectra were recorded as thin films on NaCl plates. Optical rotations were recorded on a polarimeter at 589 nm employing a 25 mm cell. High Performance Liquid Chromatography (HPLC) was performed on a chromatographic system using various

chiral columns (25 cm) as noted. X-ray diffraction was obtained by the X-ray Diffraction Laboratory at Texas A&M University. (*S*)-(–)-BTM, (*R*)-(+)-BTM and (*S*)-(–)-TM×HCl were purchased from TCI chemicals and used as received. All unsaturated acid chlorides were purchased from Sigma-Aldrich and used as received without further purification.

Abbreviation List

$EtN(^{i}Pr)_2$	=	*N*,*N*-diisopropylethylamine
Et_3N	=	triethylamine
DTBP	=	2,6-di-*tert*-butylpyridine
$NaBH_4$	=	sodium borohydride
MnO_2	=	manganese(II) oxide
K_3PO_4	=	potassium phosphate tribasic
DIBAl-H	=	diisobutylaluminum hydride
TIPSOTf	=	triisopropylsilyl trifluoromethanesulfonate
(*S*)-(–)-BTM	=	(*S*)-(–)-benzotetramisole
(*R*)-(+)-BTM	=	(*R*)-(+)-benzotetramisole
(*S*)-(–)-TM•HCl	=	(*S*)-(–)-tetramisole (levamisole) hydrochloride

Preparation of compounds S3, S4, S5, S8, S9, (±)-13a, (±)-13b, (±)-13c, (±)-13e:

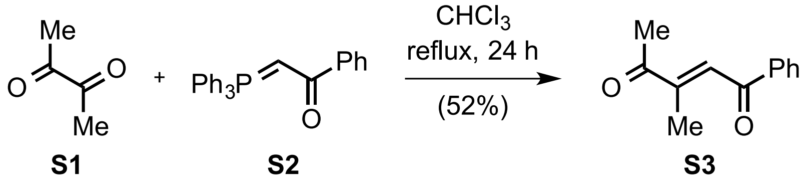

(*E*)-3-methyl-1-phenylpent-2-ene-1,4-dione (S3): To a solution of 2,3-butanedione **S1** (2.0 mL, 23.2 mmol, 1.0 equiv) in anhydrous $CHCl_3$ (77 mL) was added (benzoylmethylene)triphenylphosphorane **S2** (8.84 g, 23.2 mmol, 1.0 equiv) and refluxed (65-70 °C) for 24 h. After cooling to ambient temperature, the mixture was filtered through a short pad of celite and the filtrate was concentrated using rotary evaporation. The residue was then diluted with cold Et_2O (50 mL), filtered through a plug of celite and washed with additional Et_2O (25 mL). The filtrate was concentrated by rotary evaporation and purified by an automated flash chromatography system (5→15% EtOAc/hexanes) providing 2.24 g (52% yield) of diketone **S3** as a yellow oil: TLC (EtOAc:hexanes, 1:9 *v/v*): $R_f = 0.42$; ^{1}H NMR (500 MHz, $CDCl_3$): δ 7.95-7.93 (m, 2H), 7.62-7.58 (m, 1H), 7.51-7.48 (m, 2H), 7.44-7.42 (m, 1H), 2.47 (s, 3H), 2.07 (d, J = 1.4 Hz, 3H); ^{13}C NMR (125 MHz; $CDCl_3$): δ 200.1, 193.3, 147.0, 137.3, 133.9, 131.8, 129.0 (2), 128.7 (2), 26.4, 14.0; IR (thin film): 1681, 1668, 1597 cm^{-1}; HRMS (ESI+) *m/z* calcd for $C_{12}H_{12}LiO_2$ $[M+Li]^+$: 195.0997, found: 195.0988.

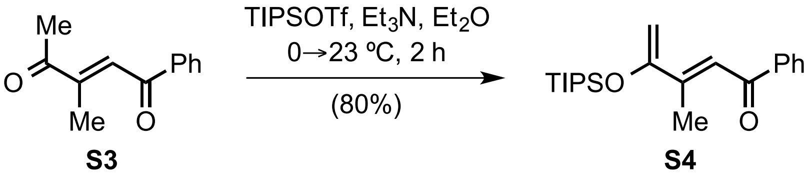

(*E*)-3-methyl-1-phenyl-4-((triisopropylsilyl)oxy)penta-2,4-dien-1-one (S4): To a solution of diketone **S3** (2.24 g, 11.9 mmol, 1.0 equiv) in anhydrous Et_2O (40 mL) at 0 ºC was added Et_3N (2.5 mL, 17.9 mmol, 1.5 equiv) dropwise. After stirring for 10 min, TIPSOTf (3.8 mL, 14.3 mmol, 1.2 equiv) was added over a period of 30 min. The reaction was stirred for 30 min at 0 ºC then allowed to warm to ambient temperature (23

ºC) and stirred for 1 h. The reaction mixture was then quenched with a saturated aqueous solution of $NaHCO_3$ (20 mL). The aqueous layer was extracted with Et_2O (2 x 50 mL) and the combined organic extracts were then washed with brine (50 mL). The organic layer was then dried over anhydrous $MgSO_4$, filtered, and concentrated by rotary evaporation. The residue was purified by an automated flash chromatography system (0.5→10% EtOAc/hexanes) providing 3.28 g (80% yield) of diene **S4** as a yellow oil: TLC (EtOAc:hexanes, 1:9 *v/v*): $R_f = 0.87$; ^1H NMR (500 MHz, $CDCl_3$): δ 7.97-7.94 (m, 2H), 7.54 (tt, $J = 7.4, 1.7$ Hz, 1H), 7.48-7.44 (m, 3H), 4.90 (d, $J = 2.0$ Hz, 1H), 4.66 (d, $J = 1.9$ Hz, 1H), 2.28 (d, $J = 1.1$ Hz, 3H), 1.33-1.26 (m, 3H), 1.14 (d, $J = 7.3$ Hz, 18H); ^{13}C NMR (125 MHz; $CDCl_3$): δ 193.1, 156.9, 148.3, 139.4, 132.7, 128.6 (2), 128.4 (2), 121.0, 96.8, 18.2 (6), 15.3, 12.9 (3); IR (thin film): 2946, 2869, 1660, 1594 cm^{-1}; HRMS (ESI+) *m/z* calcd for $C_{21}H_{33}O_2Si$ $[M+H]^+$: 345.2250, found: 345.2255.

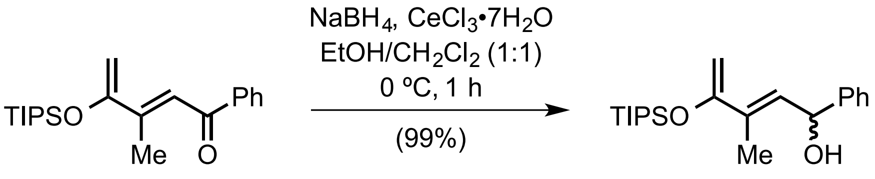

S4 (±)-**13a**

(*E*)-3-methyl-1-phenyl-4-((triisopropylsilyl)oxy)penta-2,4-dien-1-ol ((±)-13a): To a solution of diene **S4** (100 mg, 0.29 mmol, 1.0 equiv) in absolute EtOH (1.9 mL) and anhydrous CH_2Cl_2 (1.9 mL) at 0 ºC was added $CeCl_3$•$7H_2O$ (120 mg, 0.32 mmol, 1.1 equiv) in one portion. After stirring for 15 min, $NaBH_4$ (27 mg, 0.73 mmol, 2.5 equiv) was added portionwise over a period of 1 min. The reaction was stirred for 45 min at 0 ºC and then quenched with a saturated aqueous solution of $NaHCO_3$ (2.0 mL) and allowed to warm to ambient temperature. The aqueous layer was extracted with CH_2Cl_2 (2 x 5.0 mL) and the combined organic extracts were then washed with brine (2.0 mL). The organic layer was then dried over anhydrous $MgSO_4$, filtered, and concentrated by rotary evaporation. The residue was purified by an automated flash chromatography system (5 → 20% EtOAc/hexanes) providing 101 mg (99% yield) of silyloxydiene alcohol (±)-**13a** as a pale yellow oil: TLC (EtOAc:hexanes, 1:9 *v/v*): $R_f = 0.42$; ^1H NMR

(500 MHz, $CDCl_3$): δ 7.42-7.27 (m, 5H), 6.35 (d, J = 8.8 Hz, 1H), 5.58 (d, J = 8.8 Hz, 1H), 4.52 (d, J = 1.4 Hz, 1H), 4.38 (s, 1H), 1.93 (d, J = 1.1 Hz, 3H), 1.26-1.21 (m, 3H), 1.10 (dd, J = 7.4, 2.2 Hz, 18H); ^{13}C NMR (125 MHz; $CDCl_3$): δ 157.1, 143.6, 133.0, 129.8, 128.6 (2), 127.6, 126.3 (2), 92.1, 71.2, 18.2 (6), 14.0, 12.9 (3); IR (thin film): 3384, 2945, 2867, 1595 cm^{-1}; HRMS (ESI+) *m/z* calcd for $C_{21}H_{34}O_2Si$ $[M+H]^+$: 347.2406, found: 347.2390.

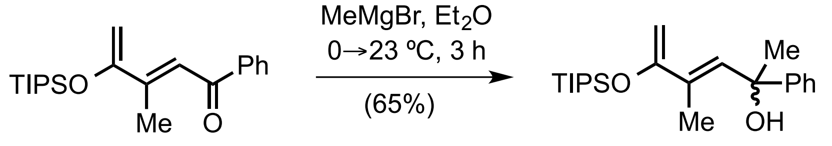

S4 (±)-13b

(*E*)-4-methyl-2-phenyl-5-((triisopropylsilyl)oxy)hexa-3,5-dien-2-ol ((±)-13b): To a solution of diene **S4** (3.28 g, 9.5 mmol, 1.0 equiv) in anhydrous Et_2O (50 mL) at 0 ºC was added MeMgBr (3.0 M solution in Et_2O, 4.8 mL, 14.4 mmol, 1.5 equiv) over a period of 1 h. The reaction was stirred for 2 h at 23 ºC then quenched with a saturated aqueous solution of NH_4Cl (25 mL). The aqueous layer was extracted with Et_2O (2 x 30 mL) and the combined organic extracts were then washed with brine (25 mL). The organic layer was then dried over anhydrous $MgSO_4$, filtered, and concentrated by rotary evaporation. The residue was purified by an automated flash chromatography system (5→15% EtOAc/hexanes) providing 2.22 g (65% yield) of silyloxydiene alcohol (±)-**13b** as a clear colorless oil: TLC (EtOAc:hexanes, 1:9 *v/v*): R_f = 0.58; ^{1}H-NMR (500 MHz; $CDCl_3$): δ 7.47-7.45 (m, 2H), 7.33-7.30 (m, 2H), 7.24-7.20 (m, 1H), 6.65 (s, 1H), 4.46 (d, J = 1.5 Hz, 1H), 4.32 (d, J = 0.5 Hz, 1H), 1.67 (s, 3H), 1.64 (d, J = 0.7 Hz, 3H), 1.28-1.22 (m, 3H), 1.12 (dd, J = 7.4, 0.8 Hz, 18H).; ^{13}C NMR (125 MHz; $CDCl_3$): δ 157.6, 148.6, 135.0, 134.7, 128.2 (2), 126.6, 125.2 (2), 91.8, 74.2, 34.1, 18.3 (6), 14.6, 12.9 (3); IR (thin film): 3454, 2945, 2867, 1594 cm^{-1}; HRMS (ESI+) *m/z* calcd for $C_{22}H_{37}O_2Si$ $[M+H]^+$: 361.2563, found: 361.2549.

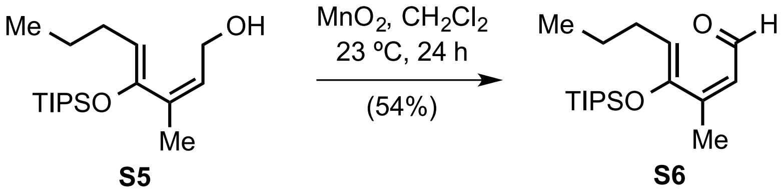

(2*Z*,4*Z*)-3-methyl-4-((triisopropylsilyl)oxy)octa-2,4-dienal (S6): To a solution of silyloxydiene alcohol **S5**[2] (0.96 g, 3.1 mmol, 1.0 equiv) in anhydrous CH_2Cl_2 (20 mL) was added MnO_2 (5.34 g, 61.4 mmol, 20.0 equiv) and the slurry was vigorously stirred at ambient temperature (23 ºC) for 24 h. The mixture was filtered through a short pad of celite and the filtrate was concentrated using rotary evaporation. Purification by an automated flash chromatography system (5→15% EtOAc/hexanes) afforded 0.38 g (54% yield) of aldehyde **S6** as a pale yellow oil: TLC (EtOAc:hexanes, 1:9 *v/v*): R_f = 0.49; ^{1}H NMR (500 MHz, $CDCl_3$): δ 10.11 (d, J = 8.1 Hz, 1H), 6.29 (dd, J = 8.1, 0.4 Hz, 1H), 5.37 (t, J = 7.3 Hz, 1H), 2.26 (d, J = 1.0 Hz, 3H), 2.19 (q, J = 7.4 Hz, 2H), 1.43 (sext, J = 7.4 Hz, 2H), 1.22-1.16 (m, 3H), 1.09 (d, J = 7.0 Hz, 18H), 0.93 (t, J = 7.4 Hz, 3H); ^{13}C NMR (125 MHz; $CDCl_3$): δ 192.0, 154.3, 150.8, 124.9, 118.2, 28.9, 22.6, 18.1 (6), 14.3, 14.1, 14.0 (3); IR (thin film): 2960, 2869, 1668, 1618 cm^{-1}; HRMS (ESI+) *m/z* calcd for $C_{18}H_{35}O_2Si$ $[M+H]^+$: 311.2406, found: 311.2403.

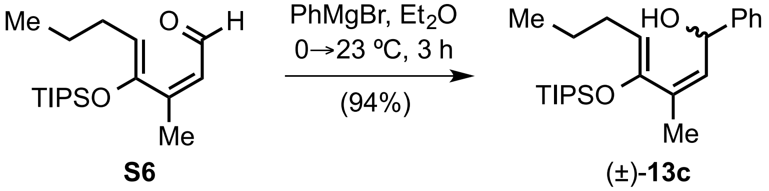

(2*Z*,4*Z*)-3-methyl-1-phenyl-4-((triisopropylsilyl)oxy)octa-2,4-dien-1-ol ((±)-13c): To a solution of aldehyde **S6** (0.38 g, 1.2 mmol, 1.0 equiv) in anhydrous Et_2O (8.0 mL) at 0 ºC was added PhMgBr (3.0 M solution in Et_2O, 0.53 mL, 1.6 mmol, 1.3 equiv) over a period of 1 h. The reaction was stirred for 2 h at 23 ºC then quenched with a saturated aqueous solution of NH_4Cl (4 mL). The aqueous layer was extracted with Et_2O (2 x 10 mL) and the combined organic extracts were then washed with brine (5 mL). The organic layer was then dried over anhydrous $MgSO_4$, filtered, and concentrated by rotary

evaporation. The residue was purified by an automated flash chromatography system (5→20% EtOAc/hexanes) providing 0.44 g (94% yield) of silyloxydiene alcohol (±)-**13c** as a pale yellow oil: TLC (EtOAc:hexanes, 1:9 *v/v*): R_f = 0.42; ^{1}H NMR (500 MHz; $CDCl_3$): δ 7.42-7.25 (m, 5H), 5.98 (dd, *J* = 8.9, 0.4 Hz, 1H), 5.57 (d, *J* = 8.9 Hz, 1H), 4.90 (t, *J* = 7.1 Hz, 1H), 2.19-2.07 (m, 2H), 1.95 (t, *J* = 0.5 Hz, 3H), 1.40 (dt, *J* = 14.9, 7.4 Hz, 2H), 1.15-1.04 (m, 21H), 0.94 (t, *J* = 7.4 Hz, 3H); ^{13}C NMR (125 MHz; $CDCl_3$): δ 151.2, 143.8, 134.8, 128.5 (2), 128.1, 127.5, 126.0 (2), 111.4, 71.3, 28.5, 23.0, 18.1 (6), 14.7, 14.2, 13.9 (3); IR (thin film): 3356, 2946, 2867 cm^{-1}; HRMS (ESI+) *m/z* calcd for $C_{24}H_{39}OSi$ $[M–OH]^+$: 371.2765, found: 371.2715.

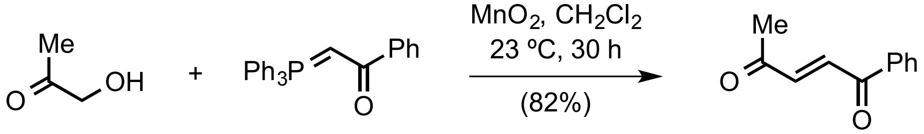

(*E*)-1-phenylpent-2-ene-1,4-dione (S9): To a solution of hydroxyacetone **S7** (2.4 mL, 34.2 mmol, 1.3 equiv) and (benzoylmethylene)triphenylphosphorane **S8** (10.0 g, 26.3 mmol, 1.0 equiv) in anhydrous CH_2Cl_2 (90 mL) was added MnO_2 (23.0 g, 262.9 mmol, 10.0 equiv) and the slurry was vigorously stirred at ambient temperature (23 ºC) for 30 h. The mixture was filtered through a short pad of celite and the filtrate was concentrated using rotary evaporation. The residue was then diluted with cold Et_2O (100 mL), filtered through a plug of celite and washed with additional Et_2O (50 mL). The filtrate was concentrated by rotary evaporation and purified by an automated flash chromatography system (5→25% EtOAc/hexanes) providing 3.76 g (82% yield) of diketone **S9** as a yellow solid: m.p. = 42-47 ºC; TLC (EtOAc:hexanes, 1:9 *v/v*): R_f = 0.28; ^{1}H NMR (500 MHz, $CDCl_3$): δ 7.98-7.96 (m, 2H), 7.68 (d, *J* = 15.8 Hz, 1H), 7.63-7.59 (m, 1H), 7.51-7.48 (m, 2H), 7.06 (d, *J* = 15.7 Hz, 1H), 2.42 (s, 3H); ^{13}C NMR (125 MHz; $CDCl_3$): δ 198.0, 190.4, 138.5, 136.7, 134.02, 133.97, 128.98 (2), 128.90 (2), 29.1; IR (thin film): 1668, 1614 cm^{-1}; HRMS (ESI+) *m/z* calcd for $C_{11}H_{10}LiO_2$ $[M+Li]^+$: 181.0841, found: 181.0833.

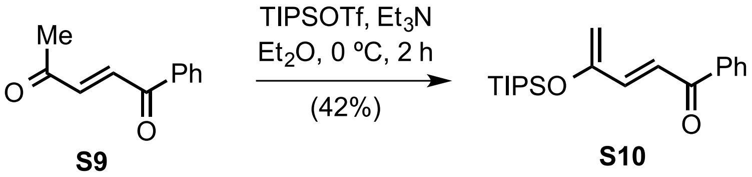

(*E*)-1-phenyl-4-((triisopropylsilyl)oxy)penta-2,4-dien-1-one (S10): To a solution of diketone **S9** (4.77 g, 27.4 mmol, 1.0 equiv) in anhydrous Et_2O (91 mL) at 0 ºC was added Et_3N (5.7 mL, 40.9 mmol, 1.5 equiv) dropwise. After stirring for 10 min, TIPSOTf (8.8 mL, 32.7 mmol, 1.2 equiv) was added over a period of 30 min. The reaction was stirred for 2 h at 0 ºC then quenched with a saturated aqueous solution of $NaHCO_3$ (45 mL). The aqueous layer was extracted with Et_2O (2 x 50 mL) and the combined organic extracts were then washed with brine (50 mL). The organic layer was then dried over anhydrous $MgSO_4$, filtered, and concentrated by rotary evaporation. The residue was purified by an automated flash chromatography system (0.5→10% EtOAc/hexanes) providing 3.76 g (42% yield) of diene **S10** as an orange oil: TLC (EtOAc:hexanes, 1:9 *v/v*): $R_f = 0.79$; ^1H NMR (500 MHz, $CDCl_3$): δ 7.96-7.95 (m, 2H), 7.59-7.55 (m, 1H), 7.50-7.47 (m, 2H), 7.35 (dd, J = 14.9, 0.3 Hz, 1H), 7.21 (d, J = 14.9 Hz, 1H), 4.75 (s, 2H), 1.33-1.27 (m, 3H), 1.15 (d, J = 7.3 Hz, 18H); ^{13}C NMR (125 MHz; $CDCl_3$): δ 190.8, 154.5, 142.5, 138.3, 132.9, 128.8 (2), 128.6 (2), 122.6, 103.4, 18.2 (6), 12.9 (3); IR (thin film): 2946, 2868, 1667, 1607, 1590 cm^{-1}; HRMS (ESI+) *m/z* calcd for $C_{20}H_{31}O_2Si$ $[M+H]^+$: 331.2093, found: 331.2178.

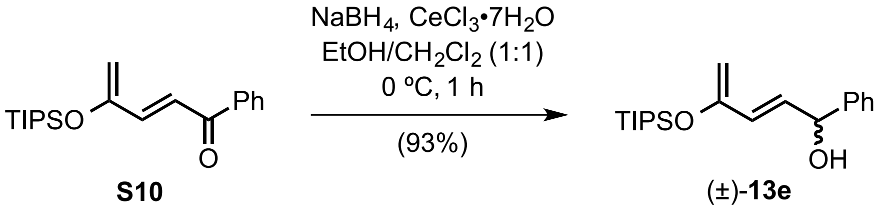

(*E*)-1-phenyl-4-((triisopropylsilyl)oxy)penta-2,4-dien-1-ol ((±)-13e): To a solution of diene **S10** (3.67 g, 11.1 mmol, 1.0 equiv) in absolute EtOH (74 mL) and anhydrous CH_2Cl_2 (74 mL) at 0 ºC was added $CeCl_3$•$7H_2O$ (4.34 g, 11.7 mmol, 1.1 equiv) in one portion. After stirring for 20 min, $NaBH_4$ (1.1 g, 27.8 mmol, 2.5 equiv) was added

portion wise over a period of 30 min. The reaction was stirred for 30 min at 0 ºC then quenched with a saturated aqueous solution of $NaHCO_3$ (30 mL) and warmed to ambient temperature. The aqueous layer was extracted with CH_2Cl_2 (2 x 100 mL) and washed with brine (30 mL). The organic layer was then dried over anhydrous $MgSO_4$, filtered, and concentrated by rotary evaporation. The residue was purified by an automated flash chromatography system (5→20% EtOAc/hexanes) providing 3.43 g (93% yield) of silyloxydiene alcohol (±)-**13e** as a yellow oil: TLC (EtOAc:hexanes, 1:9 *v/v*): R_f = 0.37; ^{1}H NMR (500 MHz, $CDCl_3$): δ 7.41-7.28 (m, 5H), 6.28 (ddd, J = 15.2, 6.1, 0.4 Hz, 1H), 6.17 (dd, J = 15.2, 1.1 Hz, 1H), 5.31 (d, J = 6.1 Hz, 1H), 4.37 (s, 1H), 4.33 (s, 1H), 1.27-1.22 (m, 3H), 1.11 (d, J = 7.4 Hz, 18H); ^{13}C NMR (125 MHz; $CDCl_3$): δ 154.8, 142.9, 132.1, 128.7, 128.6 (2), 127.8, 126.6 (2), 95.7, 74.4, 18.2 (6), 12.9 (3); IR (thin film): 3356, 2945, 2868, 1592 cm^{-1}; HRMS (ESI+) *m/z* calcd for $C_{20}H_{33}O_2Si$ $[M+H]^+$: 333.2250, found: 333.2245.

Representative procedure for the stereodivergent DAL process as described for bicyclic γ-lactones (–)-14a and (+)-14a':

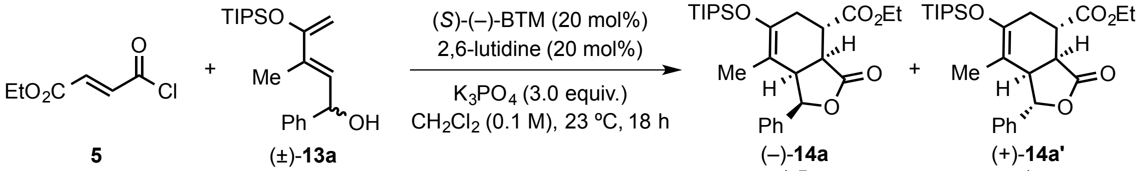

5 (±)-**13a** (–)-**14a** 1.5 48% yield >19:1 endo/exo 99% *ee* (+)-**14a'** 1 31% yield >19:1 endo/exo 98% *ee*

Ethyl (1*R*,3a*S*,4*S*,7a*R*)-7-methyl-3-oxo-1-phenyl-6-((triisopropylsilyl)oxy)-1,3,3a,4,5,7a-hexahydroisobenzofuran-4-carboxylate ((–)-14a) and ethyl (1*S*,3a*S*,4*S*,7a*R*)-7-methyl-3-oxo-1-phenyl-6-((triisopropylsilyl)oxy)-1,3,3a,4,5,7a-hexahydroiso-bezofuran-4-carboxylate ((+)-14a'): To an oven-dried, 5-mL round-bottomed flask equipped with a magnetic stir bar was added silyloxydiene alcohol (±)-**13a** (34 mg, 0.10 mmol, 1.0 equiv), (*S*)-(–)-BTM (5.0 mg, 0.020 mmol, 20 mol%), 2,6-lutidine (2.3 μL, 0.020 mmol, 20 mol%), K_3PO_4 (64.0 mg, 0.30 mmol, 3.0 equiv) and anhydrous CH_2Cl_2 (0.7 mL, to make final concentration of silyloxydiene alcohol 0.1 M) at ambient temperature (23 ºC). With vigorous stirring, ethyl fumaroyl chloride **5** (20 μL, 0.15 mmol, 1.5 equiv) in CH_2Cl_2 (0.3 mL) was added over a period of 5 h by syringe pump addition. After stirring for an additional 13 h, the reaction mixture was filtered through a pad of celite and concentrated by rotary evaporation. Purification by an automated flash chromatography (5→20% EtOAc/hexanes) afforded a single *endo* diastereomer (as judged by ^{1}H NMR) of bicyclic γ-lactone (–)-**14a** (20 mg, 48% yield, 99% *ee*) and a single *endo* diastereomer (as judged by ^{1}H NMR) of bicyclic γ-lactone (+)-**14a'** (13 mg, 31% yield, 98% *ee*).

(–)-**14a**: clear colorless oil; TLC (EtOAc:hexanes, 1:9 *v/v*): R_f = 0.53; $[\alpha]_D^{18.5}$ = –22.86 (*c* = 1.40, $CHCl_3$). Enantiomeric excess was determined by chiral HPLC analysis in comparison with authentic racemic material using a Chiralcel OD-H column: hexanes:^{i}PrOH = 95:05, flow rate 0.5 mL/min, λ = 210 nm: t_{major} = 11.4 min, t_{minor} =

13.4 min; 99% *ee*. Absolute stereochemistry was assigned by analogy to tricyclic γ-lactone (–)-**6a**[2]. ^{1}H NMR (500 MHz; $CDCl_3$): δ 7.42-7.39 (m, 2H), 7.35-7.30 (m, 3H), 5.39 (s, 1H), 4.17-4.04 (m, 2H), 3.29 (ddd, J = 7.7, 3.3, 0.9 Hz, 1H), 3.26 (dd, J = 6.1, 3.2 Hz, 1H), 3.19 (d, J = 7.5 Hz, 1H), 2.57-2.53 (m, 1H), 2.49 (ddt, J = 17.1, 6.0, 2.3 Hz, 1H), 1.80 (s, 3H), 1.21 (t, J = 7.1 Hz, 3H), 1.17-1.12 (m, 3H), 1.09 (dd, J = 6.8, 2.1 Hz, 18H); ^{13}C NMR (125 MHz; $CDCl_3$): δ 177.7, 172.8, 144.4, 139.7, 129.0 (2), 128.3, 124.9 (2), 108.0, 83.6, 61.4, 47.6, 38.5, 38.3, 28.8, 18.1 (6), 14.5, 14.2, 13.3 (3); IR (thin film): 2944, 2867, 1780, 1732, 1676 cm^{-1}; HRMS (ESI+) *m/z* calcd for $C_{27}H_{41}O_5Si$ $[M+H]^+$: 473.2723, found: 473.2734.

(+)-**14a'**: clear colorless oil; TLC (EtOAc:hexanes, 1:9 *v/v*): R_f = 0.50; $[\alpha]_D^{18.8}$ = +30.38 (c = 0.80, $CHCl_3$). Enantiomeric excess was determined by chiral HPLC analysis in comparison with authentic racemic material using a Chiralcel AS-H column: hexanes:^{i}PrOH = 95:05, flow rate 0.5 mL/min, λ = 210 nm: t_{major} = 16.9 min, t_{minor} = 19.4 min; 98% e.e. Absolute stereochemistry was assigned by analogy to a ring-opened derivative of tricyclic γ-lactone (–)-**6a'**[2]. ^{1}H NMR (500 MHz; $CDCl_3$): δ 7.41-7.38 (m, 5H), 5.07 (d, J = 10.2 Hz, 1H), 4.35-4.22 (m, 2H), 3.08-3.04 (m, 1H), 2.96 (d, J = 11.5 Hz, 1H), 2.93-2.88 (m, 1H), 2.56-2.52 (m, 2H), 1.35-1.32 (m, 6H), 1.13-1.08 (m, 3H), 1.06 (dd, J = 6.7, 2.4 Hz, 18H); ^{13}C NMR (125 MHz; $CDCl_3$): δ 173.3, 172.9, 144.8, 137.0, 129.6, 128.8 (2), 128.1 (2), 109.8, 85.4, 61.4, 50.0, 46.5, 39.9, 34.7, 18.1 (6), 14.3, 13.33, 13.27 (3); IR (thin film): 2946, 2869, 1790, 1738, 1663 cm^{-1}; HRMS (ESI+) *m/z* calcd for $C_{27}H_{41}O_5Si$ $[M+H]^+$: 473.2723, found: 473.2735.

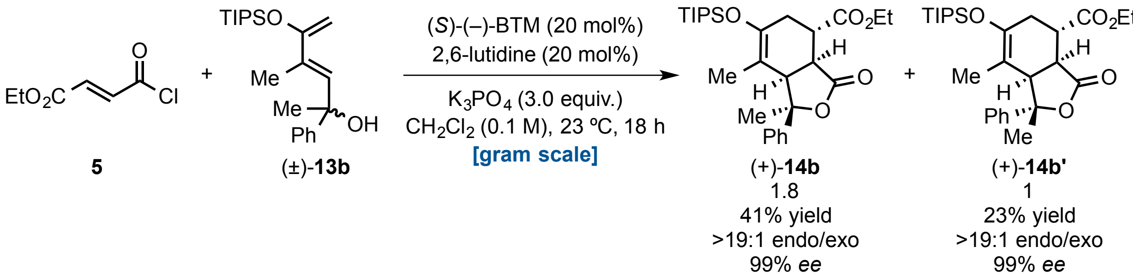

Ethyl (1*R*,3a*S*,4*S*,7a*R*)-1,7-dimethyl-3-oxo-1-phenyl-6-((triisopropylsilyl)oxy)-1,3,3a,4,5,7a-hexahydroisobenzofuran-4-carboxylate ((+)-14b) and ethyl (1*S*,3a*S*,4*S*,7a*R*)-1,7-dimethyl-3-oxo-1-phenyl-6-((triisopropylsilyl)oxy)-1,3,3a,4,5,7a-hexahydroisobenzofuran-4-carboxylate ((+)-14b'): Prepared according to the representative procedure using silyloxydiene alcohol (±)-**13b** (2.03 g, 5.63 mmol, 1.0 equiv), (*S*)-(–)-BTM (284 mg, 1.13 mmol, 20 mol%), 2,6-lutidine (0.13 mL, 1.13 mmol, 20 mol%), K_3PO_4 (3.59 g, 16.89 mmol, 3.0 equiv) in anhydrous CH_2Cl_2 (56 mL, to make initial concentration of silyloxydiene alcohol 0.1 M) and ethyl fumaroyl chloride **5** (1.13 mL, 8.44 mmol, dissolved in 24 mL CH_2Cl_2, 1.5 equiv) at ambient temperature (23 ºC). Upon completion (as judged by TLC), the reaction mixture was purified by an automated flash chromatography (5→20% EtOAc/hexanes) to afford a single *endo* diastereomer (as judged by ^{1}H NMR) of bicyclic γ-lactone (+)-**14b** (1.09 g, 41% yield, 99% e.e.) and a single *endo* diastereomer (as judged by ^{1}H NMR) of bicyclic γ-lactone (+)-**14b'** (0.62 g, 23% yield, 99% e.e.).

(+)-**14b**: clear colorless oil; TLC (EtOAc:hexanes, 1:9 *v/v*): R_f = 0.54; $[\alpha]_D^{20.1}$ = +30.30 (*c* = 3.30, $CHCl_3$). Enantiomeric excess was determined by chiral HPLC analysis in comparison with authentic racemic material using a Chiralcel OD-H column: hexanes:^{i}PrOH = 95:05, flow rate 0.5 mL/min, λ = 210 nm: t_{major} = 12.0 min, t_{minor} = 16.1 min; 99% *ee*. Absolute stereochemistry was assigned by analogy to tricyclic γ-lactone (–)-**6a**[2]. ^{1}H NMR (500 MHz; CDCl$_3$): *δ* 7.33-7.28 (m, 5H), 4.21-4.11 (m, 2H), 3.59 (ddd, *J* = 8.3, 4.4, 1.3 Hz, 1H), 3.21 (d, *J* = 8.3 Hz, 1H), 3.18 (dd, *J* = 9.7, 4.2 Hz, 1H), 2.53-2.49 (m, 1H), 2.38 (ddt, *J* = 16.5, 5.6, 2.1 Hz, 1H), 1.93 (s, 3H), 1.25 (t, *J* = 7.1 Hz, 3H), 1.08-1.04 (m, 3H), 1.02 (dd, *J* = 5.7, 3.7 Hz, 18H), 0.87 (s, 3H); ^{13}C NMR

(125 MHz; $CDCl_3$): δ 177.1, 172.8, 145.6, 141.2, 128.1 (2), 127.9, 126.2 (2), 107.4, 89.3, 61.3, 52.5, 42.4, 39.5, 29.5, 28.1, 18.1 (6), 16.4, 14.2, 13.2 (3); IR (thin film): 2945, 2867, 1770, 1732, 1666 cm^{-1}; HRMS (ESI+) *m/z* calcd for $C_{28}H_{43}O_5Si$ $[M+H]^+$: 487.2880, found: 487.2862.

(+)-**14b'**: clear colorless oil; TLC (EtOAc:hexanes, 1:9 *v/v*): R_f = 0.65; $[\alpha]_D^{19.7}$ = +51.85 (*c* = 2.70, $CHCl_3$). Enantiomeric excess was determined by chiral HPLC analysis in comparison with authentic racemic material using a Chiralcel AD-H column: hexanes:^{i}PrOH = 95:05, flow rate 0.8 mL/min, λ = 210 nm: t_{major} = 6.5 min, t_{minor} = 7.9 min; 99% *ee*. Absolute stereochemistry was assigned by analogy to a ring-opened derivative of tricyclic γ-lactone (–)-**6a'**[2]. ^{1}H NMR (500 MHz; $CDCl_3$): δ 7.43-7.41 (m, 2H), 7.34-7.29 (m, 3H), 4.30-4.15 (m, 2H), 3.02 (dt, *J* = 13.5, 1.4 Hz, 1H), 2.85 (t, *J* = 12.5 Hz, 1H), 2.77 (td, *J* = 10.9, 6.5 Hz, 1H), 2.49-2.36 (m, 2H), 1.92 (s, 3H), 1.88 (s, 3H), 1.29 (t, *J* = 7.1 Hz, 3H), 1.11-1.07 (m, 3H), 1.05 (d, *J* = 6.7 Hz, 18H); ^{13}C NMR (125 MHz; $CDCl_3$): δ 173.6, 173.1, 145.8, 139.9, 128.6 (2), 128.2, 126.2 (2), 109.4, 88.6, 61.3, 54.7, 44.3, 39.9, 35.0, 30.1, 18.1 (6), 14.26, 14.21, 13.2(3); IR (thin film): 2945, 2868, 1786, 1737, 1652 cm^{-1}; HRMS (ESI+) *m/z* calcd for $C_{28}H_{43}O_5Si$ $[M+H]^+$: 487.2880, found: 487.2891.

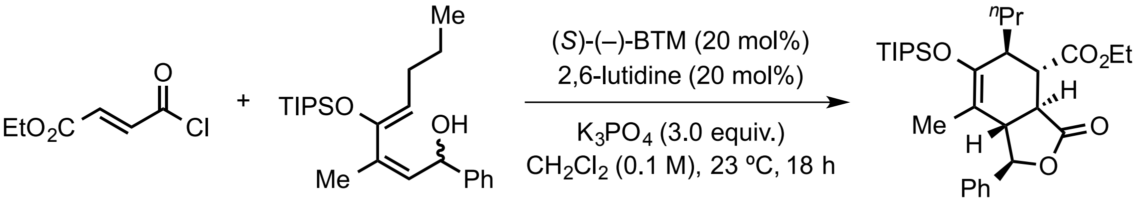

5 (±)-**13c** (+)-**14c**
>19:1 *dr*
40% yield
>19:1 endo/exo
99% *ee*

Ethyl (1*R*,3a*S*,4*S*,5*S*,7a*S*)-7-methyl-3-oxo-1-phenyl-5-propyl-6-((triisopropylsilyl)-oxy)-1,3,3a,4,5,7a-hexahydroisobenzofuran-4-carboxylate ((+)-14c): Prepared according to the representative procedure using silyloxydiene alcohol (±)-**13c** (39 mg, 0.10 mmol, 1.0 equiv), (*S*)-(–)-BTM (5.0 mg, 0.020 mmol, 20 mol%), 2,6-lutidine (2.3 μL, 0.020 mmol, 20 mol%), K_3PO_4 (64 mg, 0.30 mmol, 3.0 equiv) in anhydrous CH_2Cl_2 (0.7 mL, to make final concentration of silyloxydiene alcohol 0.1 M) and ethyl fumaroyl chloride **5** (20 μL, 0.15 mmol, dissolved in 0.3 mL CH_2Cl_2, 1.5 equiv) at ambient temperature (23 ºC). Upon completion (as judged by TLC), the reaction mixture was purified by an automated flash chromatography (5→20% EtOAc/hexanes) to afford a single *endo* diastereomer (as judged by ^{1}H NMR) of bicyclic γ-lactone (+)-**14c** (20 mg, 40% yield, 99% *ee*) as a clear colorless oil: TLC (EtOAc:hexanes, 1:9 *v/v*): R_f = 0.42; $[\alpha]_D^{20.1}$ = +41.03 (*c* = 0.39, $CHCl_3$). Enantiomeric excess was determined by chiral HPLC analysis in comparison with authentic racemic material using a Chiralpak IA column: hexanes:^{i}PrOH = 95:05, flow rate 0.5 mL/min, λ = 210 nm: t_{major} = 28.9 min, t_{minor} = 43.7 min; 99% *ee*. Absolute stereochemistry was assigned by analogy to tricyclic γ-lactone (–)-**6a**[2]. ^{1}H NMR (500 MHz; $CDCl_3$): δ 7.41-7.38 (m, 5H), 5.06 (d, *J* = 10.1 Hz, 1H), 4.32-4.19 (m, 2H), 3.03 (dd, *J* = 13.3, 11.7 Hz, 1H), 2.95 (dd, *J* = 11.6, 6.2 Hz, 1H), 2.91 (dd, *J* = 12.2, 10.9 Hz, 1H), 2.53 (t, *J* = 6.3 Hz, 1H), 1.73-1.60 (m, 2H), 1.33 (t, *J* = 7.2 Hz, 3H), 1.27 (s, 3H), 1.24-1.16 (m, 2H), 1.11-1.08 (m, 3H), 1.06 (d, *J* = 6.4 Hz, 18H), 0.87 (t, *J* = 7.2 Hz, 3H); ^{13}C NMR (125 MHz; $CDCl_3$): δ 173.5, 170.9, 148.5, 137.2, 129.5, 128.8 (2), 128.1 (2), 109.5, 85.1, 61.1, 50.7, 43.8, 43.4, 42.8, 33.0, 22.1,

18.2 (6), 14.8, 14.25, 14.24, 13.8 (3); IR (thin film): 2948, 2870, 1793, 1737, 1653 cm^{-1}; HRMS (ESI+) *m/z* calcd for $C_{30}H_{47}O_5Si$ $[M+H]^+$: 515.3193, found: 515.3211.

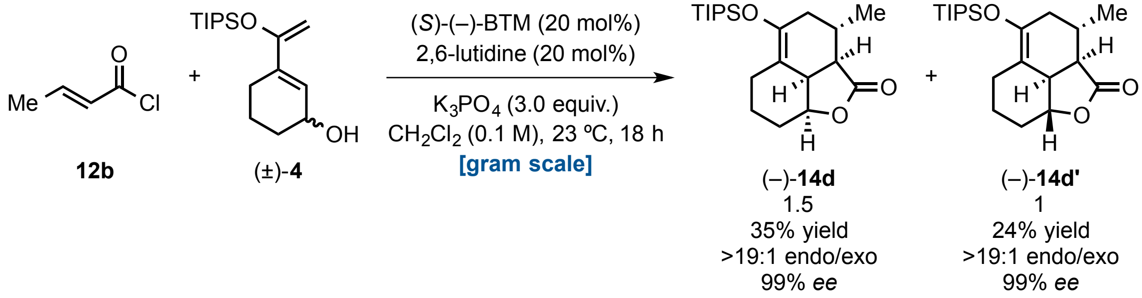

(2a*R*,2a[1]*R*,3*S*,8a*S*)-3-methyl-5-((triisopropylsilyl)oxy)-2a,2a[1],3,4,6,7,8,8a-octahydro-2*H*-naphtho[1,8-*bc*]furan-2-one ((–)-14d) and (2a*R*,2a[1]*R*,3*S*,8a*R*)-3-methyl-5-((triisopropylsilyl)oxy)-2a,2a[1],3,4,6,7,8,8a-octahydro-2*H*-naphtho[1,8-*bc*]furan-2-one ((–)-14d'): Prepared by a modified representative procedure. To an oven-dried, 250-mL round-bottomed flask equipped with a magnetic stir bar was added silyloxydiene alcohol (±)-**4**[2] (2.73 g, 9.21 mmol, 1.0 equiv), (*S*)-(–)-BTM (465 mg, 1.84 mmol, 20 mol%), 2,6-lutidine (0.21 mL, 1.84 mmol, 20 mol%), K_3PO_4 (5.87 g, 27.63 mmol, 3.0 equiv) and anhydrous CH_2Cl_2 (80 mL, to make final concentration of silyloxydiene alcohol 0.1 M) at ambient temperature (23 ºC). With vigorous stirring, crotonoyl chloride **12b** (1.32 mL, 13.82 mmol, 1.5 equiv) in CH_2Cl_2 (12 mL) was added over a period of ~5 min. After stirring for 18 h, the reaction mixture was filtered through a pad of celite and concentrated by rotary evaporation. Purification by an automated flash chromatography (5→20% EtOAc/hexanes) afforded a single *endo* diastereomer (as judged by ^1H NMR) of tricyclic γ-lactone (–)-**14d** (1.17 g, 35% yield, 99% *ee*) and a single *endo* diastereomer (as judged by ^1H NMR) of tricyclic γ-lactone (–)-**14d'** (0.80 g, 24% yield, 99% *ee*).

(–)-**14d**: clear colorless oil; TLC (EtOAc:hexanes, 1:9 *v/v*): $R_f = 0.34$; $[\alpha]_D^{19.2} = -62.86$ (c = 3.50, $CHCl_3$). Enantiomeric excess was determined by chiral HPLC analysis in comparison with authentic racemic material using a Chiralcel OD-H column:

hexanes:^{i}PrOH = 95:05, flow rate 0.5 mL/min, λ = 210 nm: t_{minor} = 11.1 min, t_{major} = 12.1 min; 99% e.e. Absolute stereochemistry was assigned by analogy to tricyclic γ-lactone (–)-**6a**[2]. ^{1}H NMR (500 MHz; $CDCl_3$): δ 4.49 (q, J = 3.3 Hz, 1H), 2.96-2.93 (m, 1H), 2.88 (d, J = 2.2 Hz, 1H), 2.57-2.51 (m, 2H), 2.50-2.44 (m, 1H), 2.19-2.15 (m, 1H), 1.74 (d, J = 17.2 Hz, 1H), 1.71-1.65 (m, 1H), 1.61-1.56 (m, 1H), 1.49 (tt, J = 12.6, 3.3 Hz, 1H), 1.42 (td, J = 12.4, 2.2 Hz, 1H), 1.13-1.07 (m, 6H), 1.05 (d, J = 5.9 Hz, 18H); ^{13}C NMR (125 MHz; $CDCl_3$): δ 178.5, 143.6, 108.6, 79.4, 47.6, 37.6, 33.2, 27.8, 25.0, 24.4, 21.0, 20.3, 18.1 (6), 13.4 (3); IR (thin film): 2943, 2867, 1778, 1675 cm^{-1}; HRMS (ESI+) *m/z* calcd for $C_{21}H_{37}O_3Si$ $[M+H]^+$: 365.2512, found: 365.2510.

(–)-**14d'**: clear colorless oil; TLC (EtOAc:hexanes, 1:9 *v/v*): R_f = 0.49; $[\alpha]_D^{19.6}$ = –12.50 (c = 1.60, $CHCl_3$). Enantiomeric excess was determined by chiral HPLC analysis in comparison with authentic racemic material using a Chiralpak IA column: hexanes:^{i}PrOH = 95:05, flow rate 0.5 mL/min, λ = 230 nm: t_{minor} = 14.1 min, t_{major} = 17.2 min; 99% e.e. Absolute stereochemistry was assigned by analogy to a ring-opened derivative of tricyclic γ-lactone (–)-**6a'**[2]. ^{1}H NMR (500 MHz; $CDCl_3$): δ 3.90 (td, J = 11.2, 3.4 Hz, 1H), 2.76 (ddd, J = 13.9, 5.1, 1.6 Hz, 1H), 2.40 (dd, J = 10.7, 7.8 Hz, 1H), 2.27-2.19 (m, 2H), 2.17-2.10 (m, 1H), 2.09-2.02 (m, 2H), 1.97-1.91 (m, 1H), 1.73-1.61 (m, 2H), 1.49-1.41 (m, 1H), 1.17 (d, J = 6.1 Hz, 3H), 1.14-1.10 (m, 3H), 1.08 (d, J = 5.6 Hz, 18H); ^{13}C NMR (125 MHz; $CDCl_3$): δ 177.8, 141.3, 110.9, 84.5, 48.8, 45.7, 38.9, 30.7, 28.1, 24.9, 24.8, 18.9, 18.1 (6), 13.2 (3); IR (thin film): 2944, 2867, 1766, 1737, 1697 cm^{-1}; HRMS (ESI+) *m/z* calcd for $C_{21}H_{37}O_3Si$ $[M+H]^+$: 365.2512, found: 365.2497.

Representative examples of unsuccessful DAL process (Table S1):

Table S1. Representative examples of unsuccessful DAL process.†

a. Unsuccessful α,β-unsaturated acid chlorides (dienophiles):

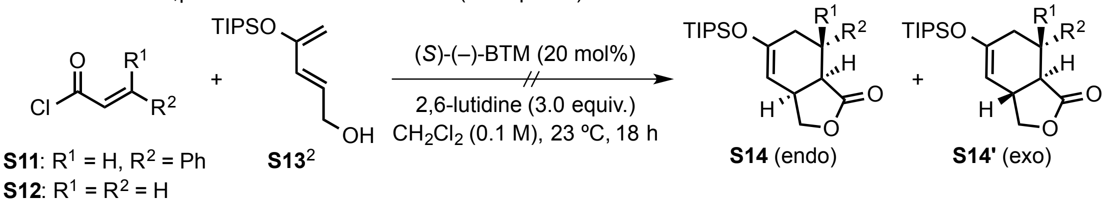

a. Unsuccessful dienes:

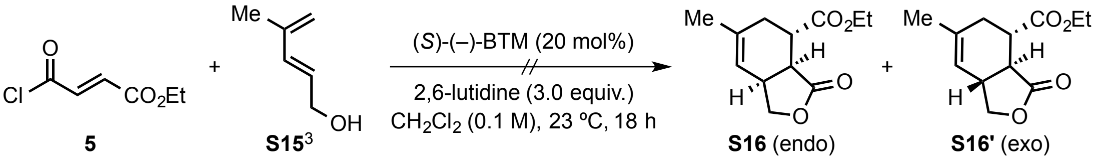

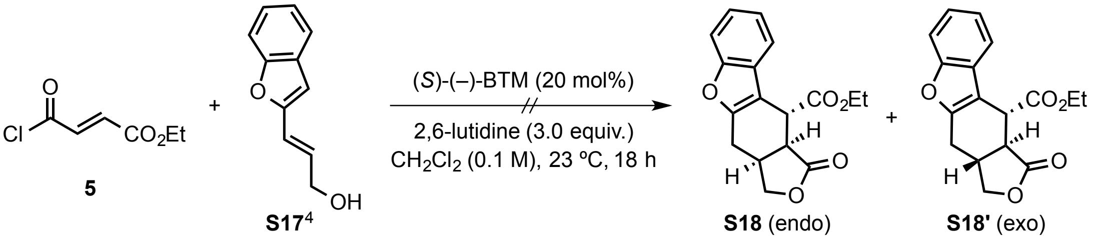

†Reactions were performed with dienes **S13**, **S15**, **S17** (1.0 equiv), acid chlorides **S11**, **S12**, **5** (1.2 equiv), (S)-(–)-BTM (20 mol%) and 2,6-lutidine (3.0 equiv) in CH_2Cl_2 (0.1 M) at ambient temperature (23 ºC) for 18 h. The crude reaction mixtures were then analyzed by LC/MS and ^1H NMR (500 MHz).

Synthetic applications of γ-lactone (–)-14d:

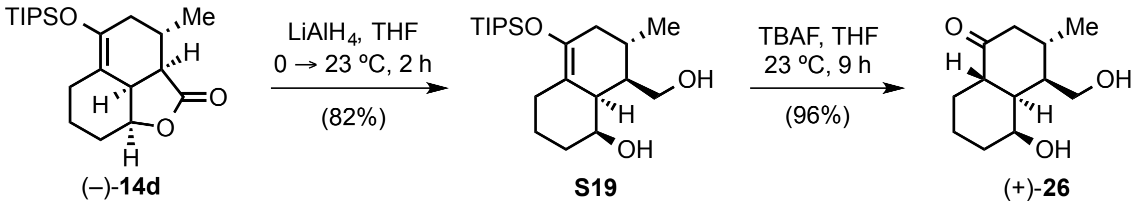

(3*S*,4*R*,4a*R*,5*S*,8a*S*)-5-hydroxy-4-(hydroxymethyl)-3-methyloctahydronaphthalen-1(2*H*)-one ((+)-26): Into an oven-dried, 10-mL round-bottomed flask containing a solution of tricyclic γ-lactone (–)-**14d** (50 mg, 0.14 mmol, 1.0 equiv) in anhydrous THF (2.7 mL, to make initial concentration of tricyclic γ-lactone 0.05 M) was added $LiAlH_4$ (2.0 M solution in THF, 0.21 mL, 0.42 mmol, 3.0 equiv) dropwise at 0 ºC. After stirring for 20 min, the ice bath was removed and the mixture was allowed to warm up to ambient temperature (23 ºC) on its own accord over 40 min. Upon completion (as judged by TLC), the reaction mixture was cooled to 0 ºC and carefully quenched in sequence with 17 μL H_2O, 17 μL 15% aqueous NaOH, and 42 μL H_2O. The ice bath was removed and the mixture was allowed to warm up to ambient temperature (23 ºC) on its own accord. Subsequently, anhydrous $MgSO_4$ was added and the reaction mixture was vigorously stirred for 30 min, filtered through a pad of Celite and concentrated by rotary evaporation to afford crude diol **S19** (41 mg, 82% yield) as a clear colorless oil. The crude material was of sufficient purity (>95% as judged by ^{1}H NMR) to be carried on directly to the next step.

To a solution of crude diol **S19** (52 mg, 0.14 mmol, 1.0 equiv) in anhydrous THF (2.8 mL, to make final concentration of crude diol 0.05 M) at 0 ºC was added TBAF (1.0 M solution in THF, 0.70 mL, 0.69 mmol, 5.0 equiv) dropwise. The reaction was stirred for 10 min at 0 ºC then allowed to warm up to ambient temperature (23 ºC) and stirred for 9 h. The reaction mixture was quenched with a saturated aqueous solution of NH_4Cl (2.0 mL). The aqueous layer was extracted with Et_2O (2 x 5.0 mL) and washed with brine (2.0 mL). The organic layer was then dried over anhydrous $MgSO_4$, filtered, and concentrated by rotary evaporation. The residue was purified by an automated flash

chromatography system (20→80% EtOAc/hexanes) providing 28 mg (96% yield) of ketone (+)-**26** as a clear colorless oil: TLC (EtOAc:hexanes, 3:1 *v/v*): $R_f = 0.36$; $[\alpha]_D^{19.7} = +17.50$ (c = 0.16, $CHCl_3$). ^1H NMR (500 MHz; $CDCl_3$): δ 4.17 (dd, J = 11.2, 8.8 Hz, 1H), 4.13 (s, 1H), 3.71 (dd, J = 11.3, 2.1 Hz, 1H), 2.71 (td, J = 12.0, 3.5 Hz, 1H), 2.41 (dd, J = 14.2, 5.7 Hz, 1H), 2.26-2.21 (m, 1H), 2.10-2.08 (m, 1H), 2.06-2.05 (m, 1H), 1.90 (dd, J = 12.8, 4.0 Hz, 1H), 1.84-1.79 (m, 1H), 1.73-1.66 (m, 1H), 1.61-1.55 (m, 1H), 1.47 (tdd, J = 13.6, 4.1, 2.1 Hz, 1H), 1.29-1.20 (m, 2H), 0.97 (d, J = 7.1 Hz, 3H); ^{13}C NMR (125 MHz; $CDCl_3$): δ 213.4, 69.3, 64.9, 46.6, 44.7, 44.4, 43.9, 35.6, 33.8, 25.9, 20.5, 18.9; IR (thin film): 3332, 2934, 1703 cm^{-1}; HRMS (ESI+) *m/z* calcd for $C_{12}H_{20}LiO_3$ $[M+Li]^+$: 219.1572, found: 219.1582.

Synthesis of the isothiourea catalysts (*R*)-(+)-NBTM and (*R*)-(+)-PBTM:

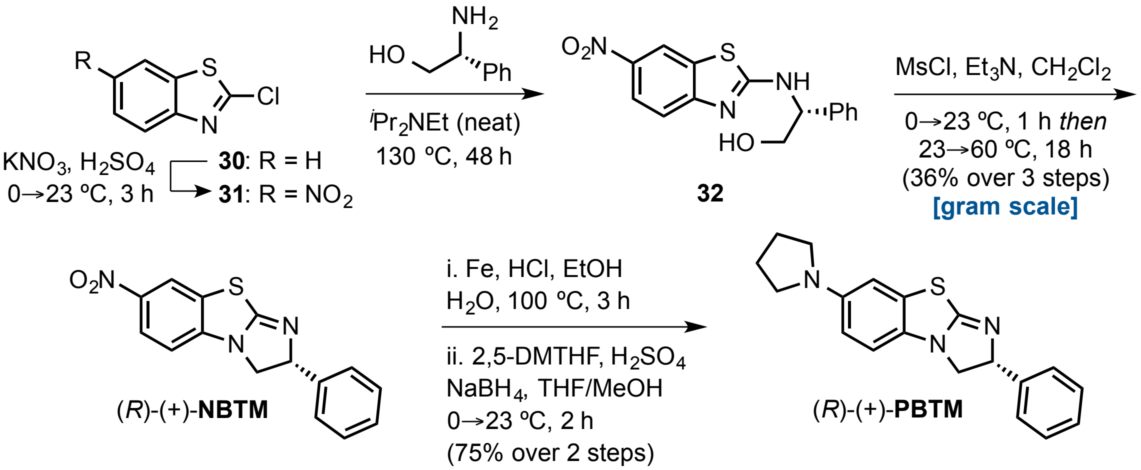

(*R*)-7-nitro-2-phenyl-2,3-dihydrobenzo[*d*]imidazo[2,1-*b*]thiazole ((*R*)-(+)-NBTM): 2-Chlorobenzothiazole (4.00 g, 24.0 mmol, 1.0 equiv) was added dropwise to a concentrated H_2SO_4 (35 mL) in ice water bath (0 ºC). Potassium nitrate (2.63 g, 26.0 mmol, 1.1 equiv) was then added at once. The resulting mixture was stirred at 0 ºC for 1 h and then at room temperature (23 ºC) for 2 h. The solution was subsequently poured onto ice. The precipitate was obtained by filtration and washed several times with ice cold water to obtain **31** with >95% purity as determined by ^{1}H NMR, which was used in the next step without further purification.

A 100 mL pressure tube containing a stirrer bar was charged with (*R*)-(–)-2-phenylglycinol (2.60 g, 19.0 mmol, 1.2 equiv), crude **31** (~3.40 g, 15.8 mmol, 1.0 equiv) and ^{i}Pr$_2$NEt (55.0 mL, to make initial concentration of **31** 0.3 M). The resulting yellow suspension was stirred vigorously and heated to reflux at 130 °C, at which point the suspended solid had dissolved to leave a yellow solution. After 48 h at 130 ºC, the orange reaction mixture was allowed to cool to room temperature (23 ºC). Once cooled, the crude reaction mixture was diluted with EtOAc/PhMe/CH_2Cl_2 (1:1:1, 150 mL) and quenched with 2N HCl (50 mL) under vigorous stirring. The aqueous layer was extracted with CH_2Cl_2 (2 x 50 mL) and washed with brine (40 mL). The organic layer was then dried over anhydrous $MgSO_4$, filtered, concentrated by rotary evaporation to deliver **32** that was used immediately without purification.

To a crude alcohol **32** (~5.00 g, 15.8 mmol, 1.0 equiv) dissolved in CH_2Cl_2 (100 mL, to make initial concentration of **32** 0.15 M) was added dropwise Et_3N (6.6 ml, 47.4 mmol, 3.0 equiv) and MsCl (1.8 ml, 23.7 mmol, 1.5 equiv) at 0 ºC. The reaction mixture was stirred at 0°C for 1 h. MeOH (1.3 ml, 23.7 mmol, 1.5 equiv) was added *via* syringe and the mixture was stirred at room temperature for 30 minutes, then Et_3N (22.0 ml, 158 mmol, 10 equiv) was added. The reaction mixture was refluxed at 60 °C for 18 h, cooled to room temperature, washed with brine, then dried over $MgSO_4$, filtered, and evaporated. The residue was purified by an automated flash chromatography system (10→90% EtOAc/hexanes) providing 2.52 g (36% yield over 3 steps) of (*R*)-(+)-**NBTM** as a pale orange solid: TLC (EtOAc:hexanes, 1:1 *v/v*): R_f = 0.30. $[\alpha]_D^{19.1}$ = +88.42 (*c* = 0.38, $CHCl_3$). ^1H NMR (500 MHz; $CDCl_3$): δ 8.21 (dd, J = 2.1, 0.7 Hz, 1H), 8.15 (ddd, J = 8.7, 2.2, 0.9 Hz, 1H), 7.41-7.31 (m, 5H), 6.68 (dd, J = 8.7, 0.6 Hz, 1H), 5.77 (dd, J = 10.1, 7.8 Hz, 1H), 4.36 (td, J = 9.8, 0.7 Hz, 1H), 3.80 (ddd, J = 9.3, 7.8, 0.8 Hz, 1H); ^{13}C NMR (125 MHz; $CDCl_3$): δ 165.1, 142.0, 141.7, 128.9 (2), 128.4, 128.0, 126.4 (2), 124.1, 119.1, 107.05, 107.04, 76.1, 52.0; IR (thin film): 1614, 1593, 1516 cm^{-1}; HRMS (ESI+) *m/z* calcd for $C_{15}H_{12}N_3O_2S$ $[M+H]^+$: 298.0650, found: 298.0652.

(*R*)-2-phenyl-7-(pyrrolidin-1-yl)-2,3-dihydrobenzo[*d*]imidazo[2,1-*b*]thiazole ((*R*)-(+)-PBTM): A suspension of Fe (280 mg, 5.0 mmol, 10 equiv) in EtOH (5.0 mL, to make initial concentration of (*R*)-(+)-**NBTM** 0.1 M) and H_2O (1.5 mL) was mixed with HCl (0.2 mL) at room temperature (23 ºC). (*R*)-(+)-**NBTM** (150 mg, 0.5 mmol, 1.0 equiv) was added to the suspension and refluxed at 100 ºC for 3 h. The resulting mixture was extracted with CH_2Cl_2 (2 x 20 mL) and washed with brine. The solvent was removed under reduced pressure and the crude was used in the next step without further purification.

A THF (1.0 mL) solution of 2,5-dimethoxytetrahydrofuran (0.10 mL, 0.59 mmol, 1.3 equiv) and 2.5M H_2SO_4 (0.50 mL, 1.13 mmol, 2.5 equiv) was added dropwise (*ca.* 20 min) to an open vessel containing a solution of the crude amine (~120 mg, 0.45 mmol, 1.0 equiv) in MeOH/THF (3.0 mL, 1:1) and $NaBH_4$ (70 mg, 1.8 mmol, 4.0 equiv)

was added under vigorous stirring at 0 ºC. The mixture was then allowed to warm up to room temperature (23 ºC) and stirred for 2 h. Then it was diluted with an aqueous $NaHCO_3$ solution (10 mL), extracted with CH_2Cl_2 (3 × 30 mL), washed with brine, then dried over $MgSO_4$, filtered, and evaporated. The residue was purified by an automated flash chromatography system (10→90% EtOAc/hexanes) providing 121 mg (75% yield over 2 steps) of (*R*)-(+)-**PBTM** as a pale orange solid: TLC (EtOAc:hexanes, 1:1 *v/v*): R_f = 0.45. $[\alpha]_D^{16.2}$ = +97.96 (*c* = 0.49, $CHCl_3$). ^{1}H NMR (500 MHz; $CDCl_3$): δ 7.41-7.35 (m, 4H), 7.30-7.27 (m, 1H), 6.60-6.58 (m, 2H), 6.41 (dd, J = 8.5, 2.3 Hz, 1H), 5.62 (t, J = 9.3 Hz, 1H), 4.22 (t, J = 9.3 Hz, 1H), 3.66 (t, J = 8.5 Hz, 1H), 3.24 (t, J = 6.5 Hz, 4H), 2.03-2.01 (m, 4H); ^{13}C NMR (125 MHz; $CDCl_3$): δ 167.5, 144.3, 143.3, 128.81, 128.63 (2), 127.8, 127.4, 126.6 (2), 109.8, 109.4, 106.7, 75.1, 53.4, 48.2 (2), 25.4 (2); IR (thin film): 2923, 2850, 1594, 1565 cm^{-1}; HRMS (ESI+) *m/z* calcd for $C_{19}H_{20}N_3S$ $[M+H]^+$: 322.1378, found: 322.1386.

Representative procedure for the enantioselective and stereodivergent DAL process leading to a full stereoisomeric complement as described for bicyclic γ-lactones (–)-14e, (+)-14e', (+)-14e'' and (–)-14e''':

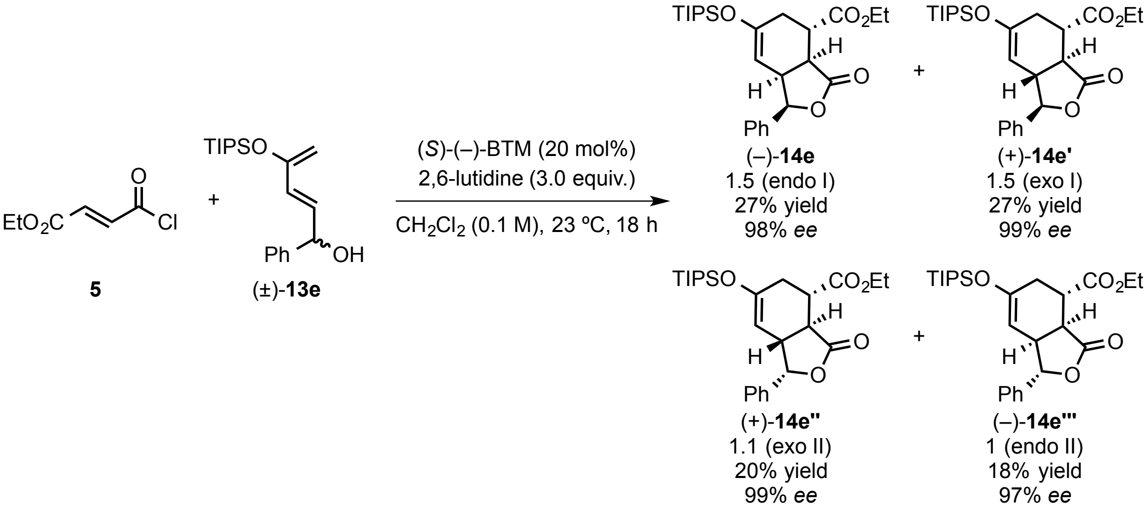

Ethyl (1*R*,3a*S*,4*S*,7a*S*)-3-oxo-1-phenyl-6-((triisopropylsilyl)oxy)-1,3,3a,4,5,7a-hexahydroisobenzofuran-4-carboxylate ((–)-14e), ethyl (1*R*,3a*S*,4*S*,7a*R*)-3-oxo-1-phenyl-6-((triisopropylsilyl)oxy)-1,3,3a,4,5,7a-hexahydroisobenzofuran-4-carboxylate ((+)-14e'), ethyl (1*S*,3a*S*,4*S*,7a*R*)-3-oxo-1-phenyl-6-((triisopropylsilyl)oxy)-1,3,3a, 4,5,7a-hexahydroisobenzofuran-4-carboxylate ((+)-14e'') and ethyl (1*S*,3a*S*,4*S*, 7a*S*)-3-oxo-1-phenyl-6-((triisopropylsilyl)oxy)-1,3,3a,4,5,7a-hexahydro-isobenzo-furan-4-carboxylate ((–)-14e'''): Prepared according to the representative procedure using silyloxydiene alcohol (±)-**13e** (33 mg, 0.10 mmol, 1.0 equiv), (*S*)-(–)-BTM (5.0 mg, 0.020 mmol, 20 mol%), 2,6-lutidine (35 μL, 0.30 mmol, 3.0 equiv) in anhydrous CH_2Cl_2 (0.7 mL, to make final concentration of silyloxydiene alcohol 0.1 M) and ethyl fumaroyl chloride **5** (20 μL, 0.15 mmol, dissolved in 0.3 mL CH_2Cl_2, 1.5 equiv) at ambient temperature (23 ºC). Upon completion (as judged by TLC), the reaction mixture was purified by an automated flash chromatography (5→20% EtOAc/hexanes) to afford bicyclic γ-lactones (–)-**14e** (12.1 mg, 27% yield, 98% *ee*), (+)-**14e'** (12.0 mg, 27% yield, 99% *ee*), (+)-**14e''** (9.0 mg, 20% yield, 99% *ee*) and (–)-**14e'''** (8.3 mg, 18% yield, 97% *ee*).

(–)-**14e:** clear colorless oil; TLC (EtOAc:hexanes, 1:9 *v/v*): $R_f = 0.49$; $[\alpha]_D^{21.0} = -16.40$ (c = 10.00, $CHCl_3$). Enantiomeric excess was determined by chiral HPLC analysis in comparison with authentic racemic material using a Chiralcel OD-H column: hexanes:^{i}PrOH = 95:05, flow rate 0.5 mL/min, λ = 210 nm: t_{major} = 11.3 min, t_{minor} = 13.6 min; 98% *ee*. Absolute stereochemistry was assigned by analogy to amide (–)-**S21**. ^{1}H NMR (500 MHz; $CDCl_3$): δ 7.40-7.37 (m, 2H), 7.34-7.29 (m, 3H), 5.20 (d, J = 2.2 Hz, 1H), 4.92 (dd, J = 2.9, 1.8 Hz, 1H), 4.18-4.07 (m, 2H), 3.28-3.24 (m, 2H), 3.20 (dd, J = 7.6, 4.1 Hz, 1H), 2.51 (ddq, J = 17.6, 2.6, 0.8 Hz, 1H), 2.44 (ddt, J = 17.6, 6.5, 2.1 Hz, 1H), 1.22 (t, J = 7.1 Hz, 3H), 1.19-1.14 (m, 3H), 1.07 (dd, J = 7.2, 1.7 Hz, 18H); ^{13}C NMR (125 MHz; $CDCl_3$): δ 177.1, 172.8, 151.4, 138.8, 128.9 (2), 128.4, 125.0 (2), 101.6, 85.9, 61.4, 42.6, 38.3, 38.1, 28.8, 18.0 (6), 14.2, 12.4 (3); IR (thin film): 2944, 2867, 1779, 1732, 1667 cm^{-1}; HRMS (ESI+) *m/z* calcd for $C_{26}H_{39}O_5Si$ $[M+H]^+$: 459.2567, found: 459.2589.

(+)-**14e':** clear colorless oil; TLC (EtOAc:hexanes, 1:9 *v/v*): $R_f = 0.43$; $[\alpha]_D^{20.2} = +35.20$ (c = 10.00, $CHCl_3$). Enantiomeric excess was determined by chiral HPLC analysis in comparison with authentic racemic material using a Chiralcel OD-H column: hexanes:^{i}PrOH = 95:05, flow rate 0.5 mL/min, λ = 210 nm: t_{major} = 22.8 min, t_{minor} = 34.0 min; 99% *ee*. Absolute stereochemistry was assigned by analogy to amide (–)-**S21**. ^{1}H NMR (500 MHz; $CDCl_3$): δ 7.43-7.33 (m, 5H), 4.96 (d, J = 9.7 Hz, 1H), 4.88 (d, J = 1.4 Hz, 1H), 4.33-4.22 (m, 2H), 2.90-2.86 (m, 2H), 2.85-2.78 (m, 1H), 2.56-2.44 (m, 2H), 1.33 (t, J = 7.1 Hz, 3H), 1.15-1.08 (m, 3H), 1.05 (t, J = 6.6 Hz, 18H); 13C NMR (125 MHz; $CDCl_3$): δ 173.1, 172.8, 153.0, 136.5, 129.1, 129.0 (2), 126.1 (2), 98.7, 84.8, 61.4, 48.2, 46.6, 39.4, 34.4, 18.0 (6), 14.2, 12.6 (3); IR (thin film): 2945, 2868, 1790, 1738, 1650 cm^{-1}; HRMS (ESI+) *m/z* calcd for $C_{26}H_{39}O_5Si$ $[M+H]^+$: 459.2567, found: 459.2544.

(+)-**14e'':** clear colorless oil; TLC (EtOAc:hexanes, 1:9 *v/v*): $R_f = 0.38$; $[\alpha]_D^{21.2} = +30.80$ (c = 10.00, $CHCl_3$). Enantiomeric excess was determined by chiral HPLC analysis in comparison with authentic racemic material using a Chiralcel OD-H column:

hexanes:^{i}PrOH = 95:05, flow rate 0.5 mL/min, λ = 210 nm: t_{minor} = 21.7 min, t_{major} = 37.0 min; 99% *ee*. Absolute stereochemistry was assigned by derivatization as described on page S28. ^{1}H NMR (500 MHz; $CDCl_3$): δ 7.37-7.30 (m, 3H), 7.19-7.17 (m, 2H), 5.64 (d, J = 7.4 Hz, 1H), 4.84 (d, J = 1.2 Hz, 1H), 4.31-4.18 (m, 2H), 3.35 (dddt, J = 13.5, 7.6, 3.6, 1.9 Hz, 1H), 2.83 (ddd, J = 11.6, 10.4, 6.9 Hz, 1H), 2.73 (dd, J = 13.6, 11.6 Hz, 1H), 2.43 (dddd, J = 17.7, 6.9, 2.0, 1.1 Hz, 1H), 2.22 (dddd, J = 17.6, 10.4, 3.5, 1.9 Hz, 1H), 1.31 (t, J = 7.1 Hz, 3H), 1.05-1.02 (m, 3H), 0.94-0.84 (m, 18H); ^{13}C NMR (125 MHz; $CDCl_3$): δ 174.2, 173.0, 151.8, 134.9, 128.5 (2), 128.3, 125.5 (2), 100.0, 81.3, 61.3, 43.7, 40.6, 39.5, 34.1, 17.9 (6), 14.2, 12.3 (3); IR (thin film): 2945, 2867, 1790, 1738, 1650 cm^{-1}; HRMS (ESI+) *m/z* calcd for $C_{26}H_{39}O_5Si$ $[M+H]^+$: 459.2567, found: 459.2584.

(–)-**14e''':** clear colorless oil; TLC (EtOAc:hexanes, 1:9 *v/v*): R_f = 0.31; $[\alpha]_D^{21.1}$ = –15.20 (c = 10.00, $CHCl_3$). Enantiomeric excess was determined by chiral HPLC analysis in comparison with authentic racemic material using a Chiralcel AS-H column: hexanes:^{i}PrOH = 95:05, flow rate 0.5 mL/min, λ = 210 nm: t_{major} = 15.5 min, t_{minor} = 28.2 min; 97% *ee*. Absolute stereochemistry was assigned by analogy to amide (–)-**S21**. ^{1}H NMR (500 MHz; $CDCl_3$): δ 7.37-7.32 (m, 2H), 7.29-7.26 (m, 1H), 7.25-7.23 (m, 2H), 5.63 (d, J = 5.2 Hz, 1H), 4.19-4.11 (m, 2H), 3.99-3.99 (m, 1H), 3.59-3.55 (m, 1H), 3.49 (dd, J = 6.8, 2.6 Hz, 1H), 3.34 (dt, J = 5.4, 2.8 Hz, 1H), 2.48-2.46 (m, 2H), 1.25 (t, J = 7.1 Hz, 3H), 0.90-0.82 (m, 21H); ^{13}C NMR (125 MHz; $CDCl_3$): δ 176.8, 173.0, 151.4, 135.7, 128.5 (2), 127.9, 125.3 (2), 97.8, 82.9, 61.3, 41.9, 40.0, 37.8, 27.9, 17.8 (6), 14.2, 12.2 (3); IR (thin film): 2945, 2867, 1778, 1731, 1665 cm^{-1}; HRMS (ESI+) *m/z* calcd for $C_{26}H_{39}O_5Si$ $[M+H]^+$: 459.2567, found: 459.2592.

Use of a lower catalyst loading for the DAL (10 mol%) as described for bicyclic γ-lactones (–)-14e, (+)-14e', (+)-14e'' and (–)-14e''' on gram scale: This reaction was performed according to the procedure described above for (–)-**14e**, (+)-**14e'**, (+)-**14e''** and (–)-**14e'''** with the exception that a lower catalyst loading (10 *vs*. 20 mol%), and a longer addition time (10 *vs*. 5 h) were employed. Silyloxydiene alcohol (±)-**13e** (3.30 g,

9.92 mmol, 1.0 equiv), (*S*)-(–)-BTM (250 mg, 0.99 mmol, 10 mol%), 2,6-lutidine (3.47 mL, 29.7 mmol, 3.0 equiv) in anhydrous CH_2Cl_2 (80 mL, to make final concentration of silyloxydiene alcohol 0.1 M) and ethyl fumaroyl chloride **5** (2.0 mL, 14.8 mmol, dissolved in 15 mL CH_2Cl_2, 1.5 equiv) at ambient temperature (23 ºC). The solution of ethyl fumaroyl chloride **5** was added by syringe pump over 10 h and the reaction was allowed to stir for 8 h at ambient temperature (23 ºC). Upon completion (as judged by TLC), the reaction mixture was purified by an automated flash chromatography (5→20% EtOAc/hexanes) to afford bicyclic γ-lactones (–)-**14e** (0.80 g, 18% yield, 98% *ee*), (+)-**14e'** (0.74 g, 16% yield, 99% *ee*), (+)-**14e''** (0.69 g, 15% yield, 99% *ee*), (–)-**14e'''** (0.68 g, 15% yield, 97% *ee*) and ester (±)-**S20** (0.54 g, 12% yield). All spectral data matched that reported above.

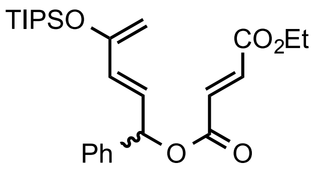

(±)-**S20**

Ethyl ((*E*)-1-phenyl-4-((triisopropylsilyl)oxy)penta-2,4-dien-1-yl) fumarate ((±)-S20): pale yellow oil; TLC (EtOAc:hexanes, 1:9 *v/v*): R_f = 0.77; $[\alpha]_D^{21.1}$ = 0.00 (*c* = 3.00, $CHCl_3$). ^{1}H NMR (500 MHz; $CDCl_3$): δ 7.37-7.36 (m, 3H), 7.32-7.29 (m, 2H), 7.15 (dd, *J* = 15.9, 10.9 Hz, 1H), 6.94-6.85 (m, 2H), 6.48 (d, *J* = 15.9 Hz, 1H), 5.68 (d, *J* = 10.9 Hz, 1H), 4.68 (s, 2H), 4.27 (q, *J* = 7.1 Hz, 2H), 1.33 (t, *J* = 7.1 Hz, 3H), 1.27-1.20 (m, 3H), 1.14 (d, *J* = 7.2 Hz, 18H); ^{13}C NMR (125 MHz; $CDCl_3$): δ 165.0, 164.7, 146.6, 138.0, 134.5, 133.1, 130.4, 128.8 (2), 127.3, 126.3 (2), 122.7, 113.5, 67.1, 61.6, 18.1 (6), 14.2, 13.3 (3); IR (thin film): 2944, 2867, 1727, 1645 cm^{-1}; HRMS (ESI+) *m/z* calcd for $C_{26}H_{39}O_5Si$ $[M+H]^+$: 459.2567, found: 459.2582.

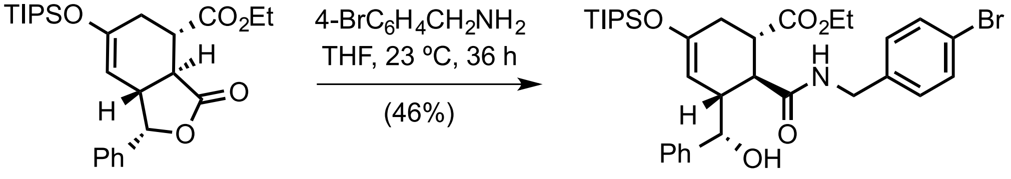

(+)-**14e"** (–)-**S21**

Ethyl (1*S*,5*R*,6*S*)-6-((4-bromobenzyl)carbamoyl)-5-((*S*)-hydroxy(phenyl)methyl)-3-((triisopropylsilyl)oxy)cyclohex-3-ene-1-carboxylate ((–)-S21): Into an oven-dried, 5-mL round-bottomed flask containing a solution of bicyclic γ-lactone (+)-**14e''** (50 mg, 0.11 mmol, 1.0 equiv) in THF (1.1 mL, to make final concentration of bicyclic γ-lactone 0.1 M), was added dropwise 4-bromobenzylamine (70 μL, 0.55 mmol, 5.0 equiv). The reaction was allowed to stir at ambient temperature (23 ºC) for 36 h. Upon completion (as judged by TLC), the reaction was concentrated by rotary evaporation and purified by an automated flash chromatography system (20→50% EtOAc/hexanes) to afford amide (–)-**S21** (32 mg, 46% yield) as a white solid: m.p. 151-155 ºC (recrystallized from Et_2O); TLC (EtOAc:hexanes, 1:2 *v/v*): R_f = 0.49; $[\alpha]_D^{20.4}$ = -13.95 (*c* = 0.86, $CHCl_3$). Absolute stereochemistry was assigned based on X-ray analysis using anomalous dispersion (see **Figure S1**). ^{1}H NMR (500 MHz; $CDCl_3$): δ 7.42 (d, *J* = 8.3 Hz, 2H), 7.37-7.34 (m, 2H), 7.29-7.24 (m, 3H), 7.19 (d, *J* = 8.3 Hz, 2H), 6.73 (t, *J* = 6.0 Hz, 1H), 4.82 (d, *J* = 4.6 Hz, 1H), 4.51 (dd, *J* = 14.9, 6.5 Hz, 1H), 4.42 (s, 1H), 4.30 (dd, *J* = 15.0, 5.5 Hz, 1H), 4.09-3.97 (m, 2H), 3.10-3.06 (m, 2H), 2.83 (dd, *J* = 11.6, 10.4 Hz, 1H), 2.41-2.24 (m, 2H), 1.22 (t, *J* = 7.1 Hz, 3H), 1.07-1.03 (m, 3H), 0.99 (dd, *J* = 14.2, 6.1 Hz, 18H); ^{13}C NMR (125 MHz; $CDCl_3$): δ 174.7, 173.8, 151.9, 142.4, 137.6, 131.8 (2), 129.7 (2), 128.3 (2), 127.2, 125.3 (2), 121.3, 98.5, 73.0, 61.1, 45.9, 45.5, 43.2, 43.1, 32.9, 18.0 (6), 14.2, 12.5 (3); IR (thin film): 3316, 2925, 2866, 1728, 1673, 1645 cm^{-1}; HRMS (ESI+) *m/z* calcd for $C_{33}H_{47}BrNO_5Si$ $[M+H]^+$: 644.2407, found: 644.2384.

Figure S1. Single crystal X-ray structure (ORTEP) of amide (–)-**S21.** The crystals were grown from a concentrated solution of amide (–)-**S21** in Et_2O (2.0 mL), using a slow evaporation method (probability ellipsoids are shown at the 50% level). X-ray crystallographic data have been deposited in the Cambridge Crystallographic Data Centre database (http://www.ccdc.cam.ac.uk/) under accession code CCDC 972246.

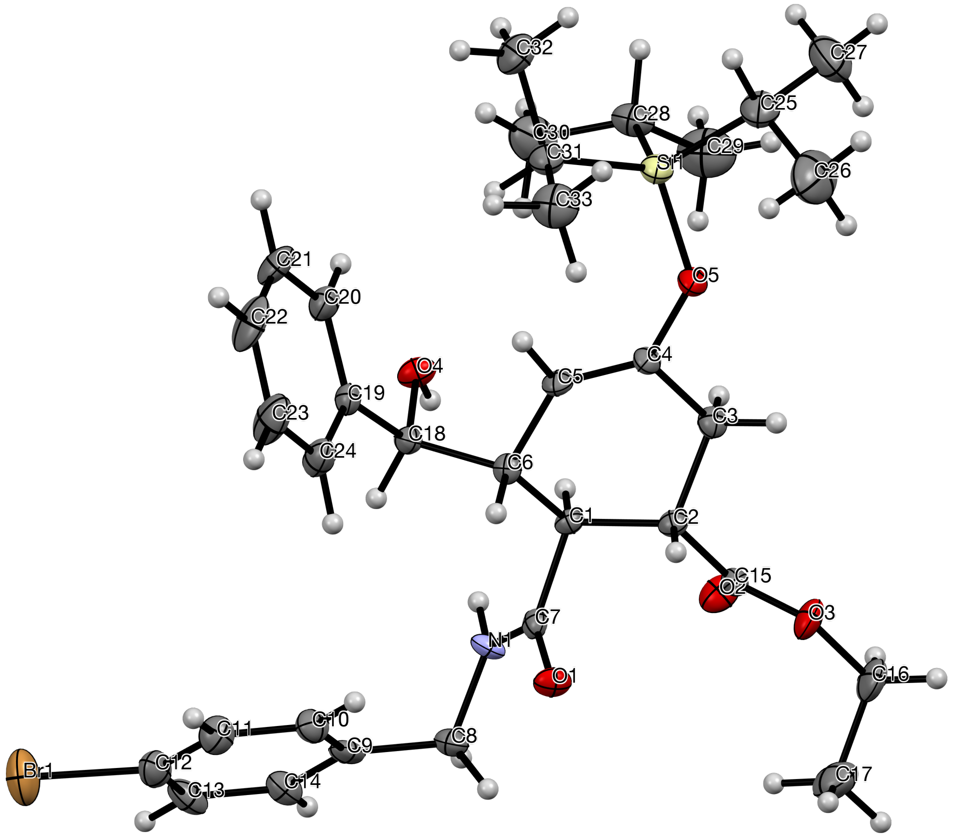

Alert level B:

Crystal system given = orthorhombic

PLAT019_ALERT_1_B Check _diffrn_measured_fraction_theta_full/_max … 0.890. Author Response: Data was collected on a Bruker GADDS instrument with Cu-source and MWPC (multiwire proportional counter) detector. Under these experimental conditions the maximum angle that can be collected is 120 degrees two-theta.

Table 1. Crystal data and structure refinement for DRB_MA_131001_G_1075C.

Crystal Parameters	Crystal Data	
Identification code	1075c	
Empirical formula	$C_{33} H_{46} Br N O_5 Si$	
Formula weight	644.71	
Temperature	110.15 K	
Wavelength	1.54178 Å	
Crystal system	Orthorhombic	
Space group	P 21 21 21	
Unit cell dimensions	a = 9.0910(3) Å	α = 90º
	b = 18.1061(7) Å	β = 90º
	c = 20.6924(7) Å	γ = 90º
Volume	3406.0(2) $Å^3$	
Z	4	
Density (calculated)	1.257 Mg/m^3	
Absorption coefficient	2.285 mm^{-1}	
F(000)	1360	
Crystal size	0.23 x 0.01 x 0.01 mm^3	
Theta range for data collection	3.243 to 62.561º	
Index ranges	$-10 \le h \le 9$, $-20 \le k \le 20$, $-22 \le l \le 23$	
Reflections collected	34574	
Independent reflections	5224 [R(int) = 0.0645]	
Completeness to theta = 67.679º	86.9%	
Absorption correction	Semi-empirical from equivalents	
Max. and min. transmission	0.7522 and 0.6042	
Refinement method	Full-matrix least-squares on F^2	
Data / restraints / parameters	5224 / 0 / 378	

Goodness-of-fit on F^2	1.126
Final R indices [I>2sigma(I)]	R_1 = 0.0328, wR_2 = 0.0707
R indices (all data)	R_1 = 0.0423, wR_2 = 0.0774
Absolute structure parameter	-0.005(8)
Extinction coefficient	N/A
Largest diff. peak and hole	0.319 and -0.512 e.$Å^{-3}$

Table 2. Atomic coordinates (x 10^4) and equivalent isotropic displacement parameters ($Å^2$ x 10^3) for DRB_MA_131001_G_1075C. U(eq) is defined as one third of the trace of the orthogonalized U^{ij} tensor.

Atom	x	y	z	U(eq)
Br(1)	2728(1)	4280(1)	8166(1)	44(1)
Si(1)	3297(1)	-1217(1)	4487(1)	22(1)
O(1)	1441(3)	2784(2)	4768(1)	21(1)
O(2)	4103(3)	2482(2)	3520(2)	28(1)
O(3)	1952(3)	2162(2)	3052(1)	28(1)
O(4)	4949(3)	1042(2)	5764(1)	22(1)
O(5)	2642(3)	-439(1)	4168(1)	22(1)
N(1)	3780(4)	3040(2)	5064(2)	18(1)
C(1)	3183(4)	1797(2)	4697(2)	15(1)
C(2)	2520(4)	1584(2)	4038(2)	17(1)
C(3)	3025(4)	811(2)	3845(2)	19(1)
C(4)	2728(5)	280(2)	4383(2)	18(1)
C(5)	2498(5)	489(2)	4985(2)	19(1)
C(6)	2589(4)	1271(2)	5224(2)	16(1)
C(7)	2740(5)	2585(2)	4851(2)	17(1)
C(8)	3504(5)	3817(2)	5227(2)	21(1)

C(9)	3297(4)	3935(2)	5944(2)	21(1)
C(10)	2274(5)	3523(2)	6290(2)	24(1)
C(11)	2083(5)	3633(2)	6946(2)	28(1)
C(12)	2938(5)	4152(2)	7260(2)	28(1)
C(13)	3945(5)	4578(2)	6928(2)	29(1)
C(14)	4122(5)	4467(2)	6267(2)	23(1)
C(15)	2976(5)	2129(2)	3519(2)	20(1)
C(16)	2231(6)	2687(3)	2533(2)	36(1)
C(17)	1646(6)	3434(3)	2714(3)	43(1)
C(18)	3502(4)	1306(2)	5860(2)	18(1)
C(19)	2751(4)	847(2)	6376(2)	19(1)
C(20)	3374(5)	194(2)	6595(2)	26(1)
C(21)	2599(5)	-255(3)	7025(2)	32(1)
C(22)	1229(5)	-45(3)	7241(2)	42(1)
C(23)	627(5)	615(3)	7036(2)	41(1)
C(24)	1383(5)	1063(3)	6611(2)	30(1)
C(25)	2632(6)	-1946(2)	3902(2)	34(1)
C(26)	1200(7)	-1727(3)	3572(3)	57(2)
C(27)	3760(7)	-2181(3)	3392(3)	53(2)
C(28)	5352(5)	-1166(3)	4542(2)	31(1)
C(29)	6061(6)	-795(3)	3948(3)	52(2)
C(30)	5946(6)	-799(3)	5157(3)	46(1)
C(31)	2506(5)	-1392(2)	5311(2)	27(1)
C(32)	3065(6)	-2124(3)	5592(2)	38(1)
C(33)	821(5)	-1363(3)	5330(3)	38(1)

Figure S2: Chiral HPLC determinations of enantiomeric excess of lactones 14a-d and 14a'-d':

Determination of enantiomeric excess of bicyclic γ-lactone (–)-14a:

Chiral HPLC analysis of bicyclic γ-lactone (–)-14a: Chiralcel OD-H column: hexanes:^{i}PrOH = 95:05, flow rate 0.5 mL/min, λ = 210 nm: t_{major} = 11.4 min, t_{minor} = 13.4 min; 99% *ee*.

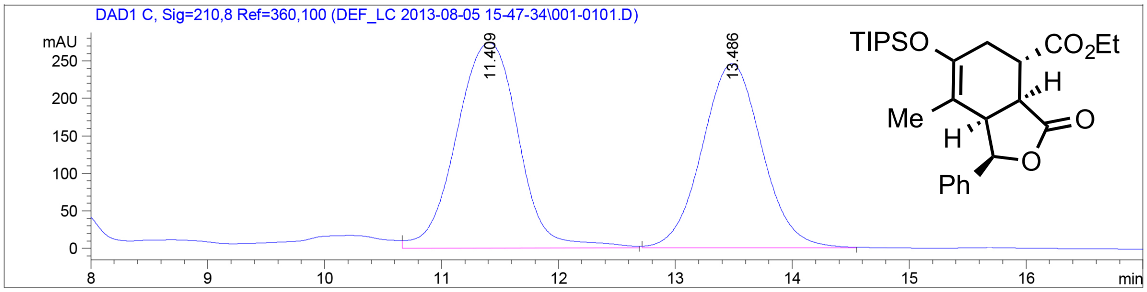

Signal 3: DAD1 C, Sig=210,8 Ref=360,100

Peak #	RetTime [min]	Type	Width [min]	Area [mAU*s]	Height [mAU]	Area %
1	11.409	VB	0.5858	1.00989e4	274.13562	53.6631
2	13.486	BB	0.5504	8720.16016	245.47592	46.3369
Totals :				1.88191e4	519.61154	

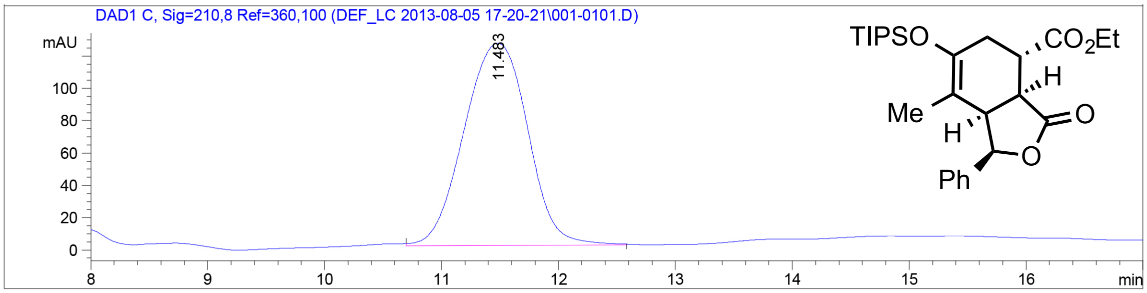

Signal 3: DAD1 C, Sig=210,8 Ref=360,100

Peak #	RetTime [min]	Type	Width [min]	Area [mAU*s]	Height [mAU]	Area %
1	11.483	BB	0.6047	4723.80225	125.05634	100.0000
Totals :				4723.80225	125.05634	

Determination of enantiomeric excess of bicyclic γ-lactone (+)-14a’:

Chiral HPLC analysis of bicyclic γ-lactone (+)-14a’: Chiralcel AS-H column: hexanes:^{i}PrOH = 95:05, flow rate 0.5 mL/min, λ = 210 nm: t_{major} = 16.9 min, t_{minor} = 19.4 min; 98% *ee*.

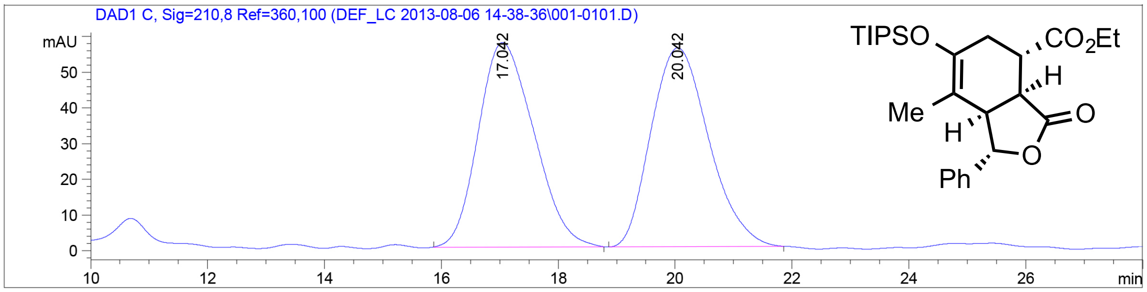

Signal 3: DAD1 C, Sig=210,8 Ref=360,100

Peak #	RetTime [min]	Type	Width [min]	Area [mAU*s]	Height [mAU]	Area %
1	17.042	BB	0.9989	3786.13672	57.29754	49.6845
2	20.042	BB	1.0521	3834.22778	55.77229	50.3155

Totals : 7620.36450 113.06982

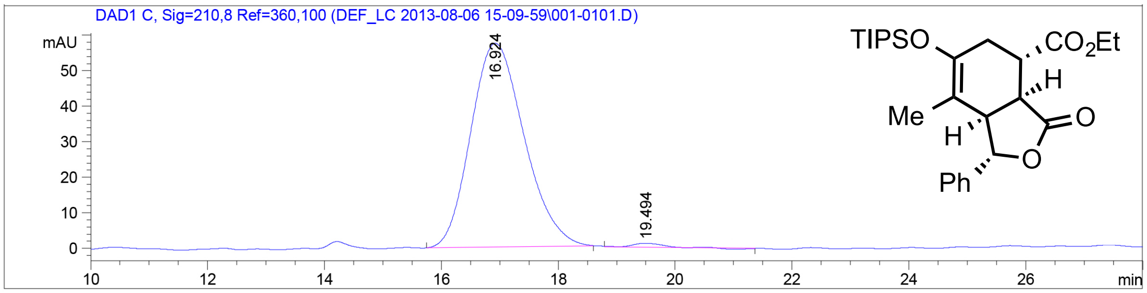

Signal 3: DAD1 C, Sig=210,8 Ref=360,100

Peak #	RetTime [min]	Type	Width [min]	Area [mAU*s]	Height [mAU]	Area %
1	16.924	BB	0.9644	3652.71582	57.39919	98.9651
2	19.494	MM	0.5868	38.19721	1.08484	1.0349

Totals : 3690.91303 58.48403

Determination of enantiomeric excess of bicyclic γ-lactone (+)-14b:

Chiral HPLC analysis of bicyclic γ-lactone (+)-14b: Chiralcel OD-H column: hexanes:^{i}PrOH = 95:05, flow rate 0.5 mL/min, λ = 210 nm: t_{major} = 12.0 min, t_{minor} = 16.1 min; 99% *ee*.

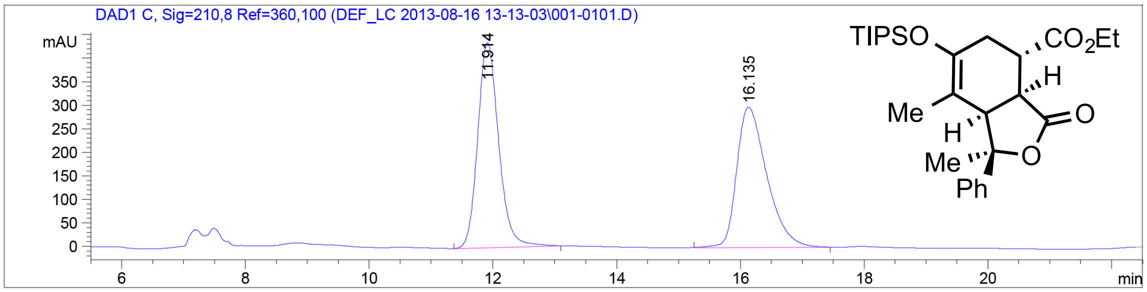

Signal 3: DAD1 C, Sig=210,8 Ref=360,100

Peak #	RetTime [min]	Type	Width [min]	Area [mAU*s]	Height [mAU]	Area %
1	11.914	BB	0.3629	1.02704e4	435.91010	50.5007
2	16.135	BB	0.5138	1.00668e4	298.20029	49.4993
Totals :				2.03372e4	734.11038	

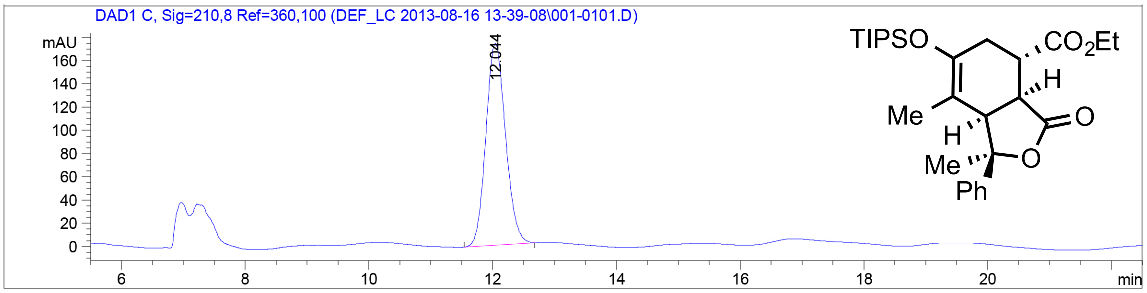

Signal 3: DAD1 C, Sig=210,8 Ref=360,100

Peak #	RetTime [min]	Type	Width [min]	Area [mAU*s]	Height [mAU]	Area %
1	12.044	BB	0.3434	3834.70825	173.81255	100.0000
Totals :				3834.70825	173.81255	

Determination of enantiomeric excess of bicyclic γ-lactone (+)-14b':

Chiral HPLC analysis of bicyclic γ-lactone (+)-14b': Chiralcel AD-H column: hexanes:^{i}PrOH = 95:05, flow rate 0.8 mL/min, λ = 210 nm: t_{major} = 6.5 min, t_{minor} = 7.9 min; 99% *ee*.

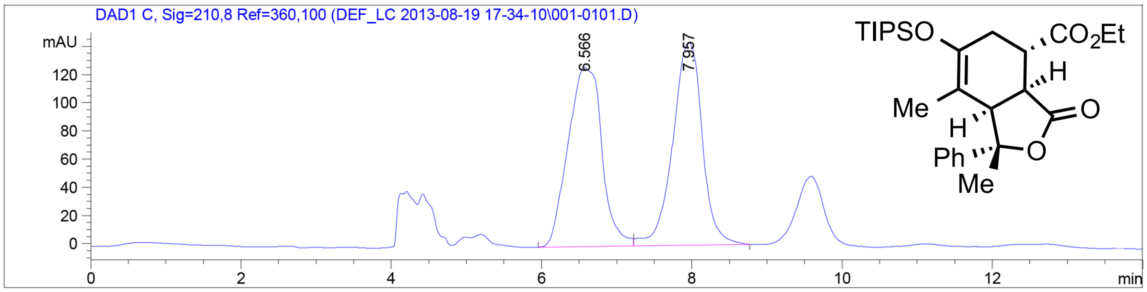

Signal 3: DAD1 C, Sig=210,8 Ref=360,100

Peak #	RetTime [min]	Type	Width [min]	Area [mAU*s]	Height [mAU]	Area %
1	6.566	BV	0.4437	3982.09033	127.05120	50.2380
2	7.957	VB	0.4200	3944.36377	143.80565	49.7620

Totals : 7926.45410 270.85685

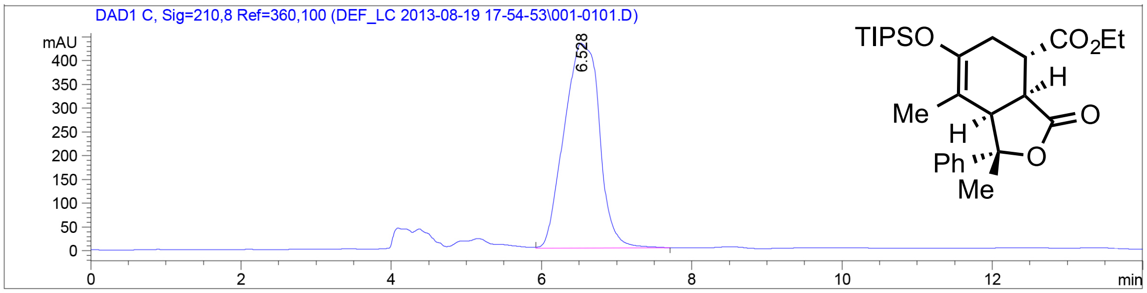

Signal 3: DAD1 C, Sig=210,8 Ref=360,100

Peak #	RetTime [min]	Type	Width [min]	Area [mAU*s]	Height [mAU]	Area %
1	6.528	BB	0.4637	1.41129e4	431.31570	100.0000

Totals : 1.41129e4 431.31570

Determination of enantiomeric excess of bicyclic γ-lactone (+)-14c:

Chiral HPLC analysis of bicyclic γ-lactone (+)-14c: Chiralpak IA column: hexanes:^{i}PrOH = 95:05, flow rate 0.5 mL/min, λ = 210 nm: t_{major} = 28.9 min, t_{minor} = 43.7 min; 99% *ee*.

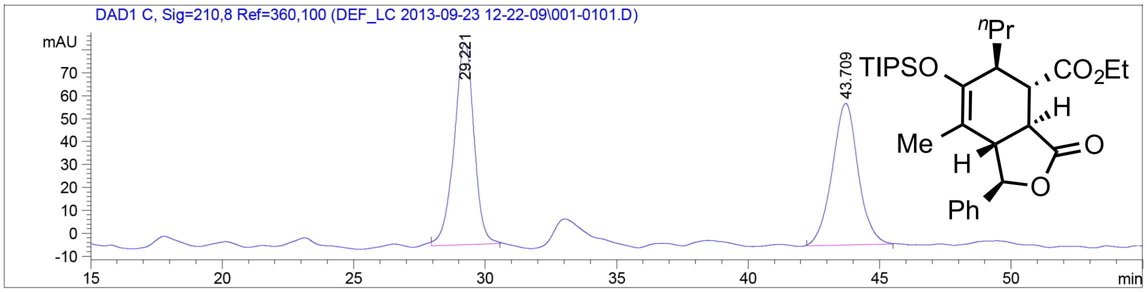

Signal 3: DAD1 C, Sig=210,8 Ref=360,100

Peak #	RetTime [min]	Type	Width [min]	Area [mAU*s]	Height [mAU]	Area %
1	29.221	BB	0.6228	4598.02588	88.11724	51.9386
2	43.709	BB	0.9143	4254.78027	61.71974	48.0614
Totals :				8852.80615	149.83698	

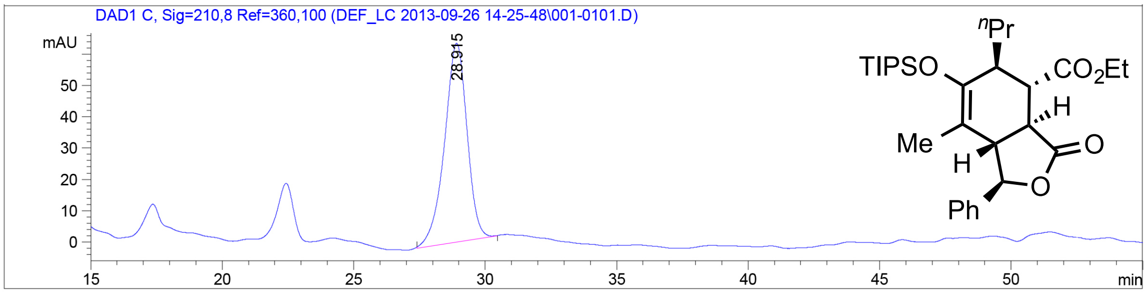

Signal 3: DAD1 C, Sig=210,8 Ref=360,100

Peak #	RetTime [min]	Type	Width [min]	Area [mAU*s]	Height [mAU]	Area %
1	28.915	BB	0.8574	3764.60156	63.51937	100.0000
Totals :				3764.60156	63.51937	

Determination of enantiomeric excess of tricyclic γ-lactone (–)-14d:

Chiral HPLC analysis of tricyclic γ-lactone (–)-14d: Chiralcel OD-H column: hexanes:^{i}PrOH = 95:05, flow rate 0.5 mL/min, λ = 210 nm: t_{minor} = 11.1 min, t_{major} = 12.1 min; 99% *ee*.

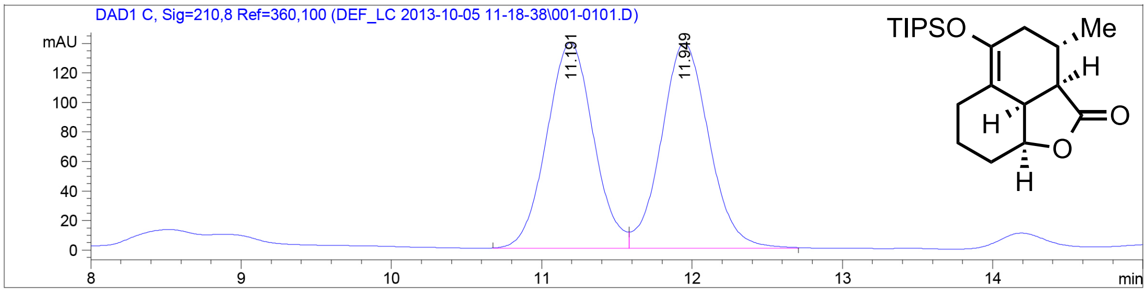

Signal 3: DAD1 C, Sig=210,8 Ref=360,100

Peak #	RetTime [min]	Type	Width [min]	Area [mAU*s]	Height [mAU]	Area %
1	11.191	BV	0.3332	2997.38525	139.20857	49.5180
2	11.949	VB	0.3369	3055.73779	138.77129	50.4820

Totals : 6053.12305 277.97986

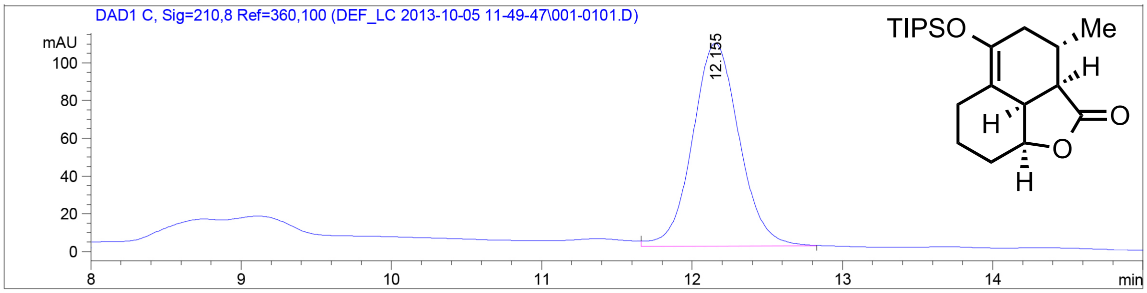

Signal 3: DAD1 C, Sig=210,8 Ref=360,100

Peak #	RetTime [min]	Type	Width [min]	Area [mAU*s]	Height [mAU]	Area %
1	12.155	VB	0.3300	2325.51343	107.66201	100.0000

Totals : 2325.51343 107.66201

Determination of enantiomeric excess of tricyclic γ-lactone (–)-14d’:

Chiral HPLC analysis of tricyclic γ-lactone (–)-14d’: Chiralpak IA column: hexanes:^{i}PrOH = 95:05, flow rate 0.5 mL/min, λ = 230 nm: t_{minor} = 14.1 min, t_{major} = 17.2 min; 99% *ee*.

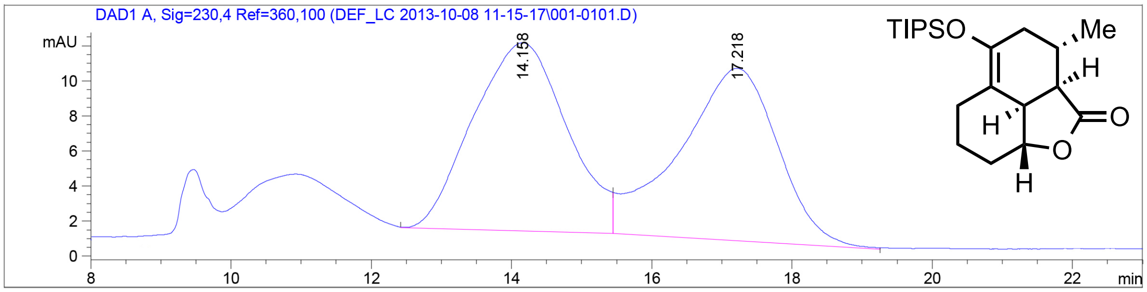

Signal 1: DAD1 A, Sig=230,4 Ref=360,100

Peak #	RetTime [min]	Type	Width [min]	Area [mAU*s]	Height [mAU]	Area %
1	14.158	MM	1.5510	999.61298	10.74140	51.0243
2	17.218	MM	1.6256	959.47900	9.83724	48.9757

Totals : 1959.09198 20.57864

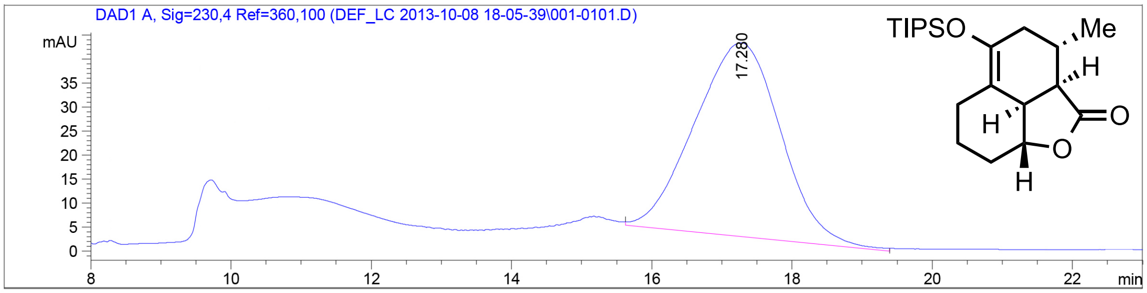

Signal 1: DAD1 A, Sig=230,4 Ref=360,100

Peak #	RetTime [min]	Type	Width [min]	Area [mAU*s]	Height [mAU]	Area %
1	17.280	MM	1.4246	3447.87793	40.33750	100.0000

Totals : 3455.97684 41.11370

Figure S3: Determination of enantiomeric excess of bicyclic γ-lactones (–)-14e, (+)-14e', (+)-14e'' and (–)-14e''':

Chiral HPLC analysis of bicyclic γ-lactone (–)-14e: Chiralcel OD-H column: hexanes:^{i}PrOH = 95:05, flow rate 0.5 mL/min, λ = 210 nm: t_{major} = 11.3 min, t_{minor} = 13.6 min; 98% *ee*.

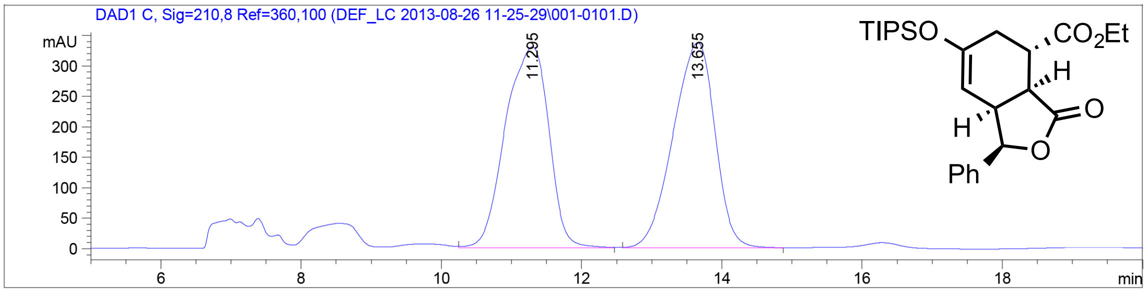

Signal 3: DAD1 C, Sig=210,8 Ref=360,100

Peak #	RetTime [min]	Type	Width [min]	Area [mAU*s]	Height [mAU]	Area %
1	11.295	VB	0.6265	1.42095e4	329.90613	50.4425
2	13.655	BB	0.6656	1.39602e4	336.77118	49.5575
Totals :				2.81697e4	666.67731	

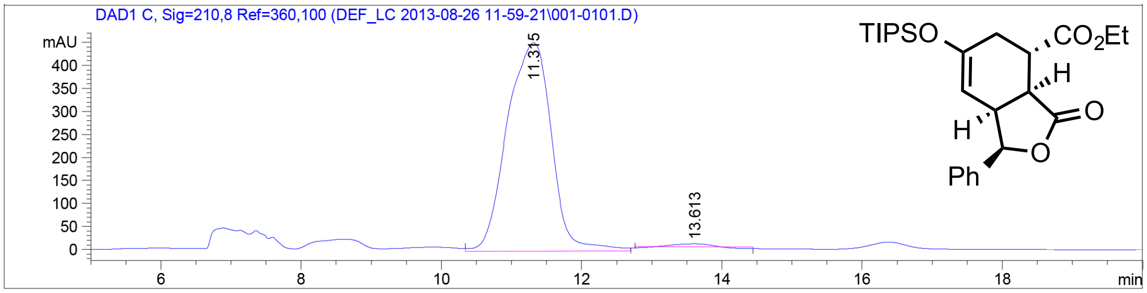

Signal 3: DAD1 C, Sig=210,8 Ref=360,100

Peak #	RetTime [min]	Type	Width [min]	Area [mAU*s]	Height [mAU]	Area %
1	11.315	MM	0.7342	1.99413e4	452.69849	98.9250
2	13.613	MM	0.5690	216.69820	6.34695	1.0750
Totals :				2.01580e4	459.04544	

Determination of enantiomeric excess of bicyclic γ-lactone (+)-14e':

Chiral HPLC analysis of bicyclic γ-lactone (+)-14e': Chiralcel OD-H column: hexanes:^{i}PrOH = 95:05, flow rate 0.5 mL/min, λ = 210 nm: t_{major} = 22.8 min, t_{minor} = 34.0 min; 99% *ee*.

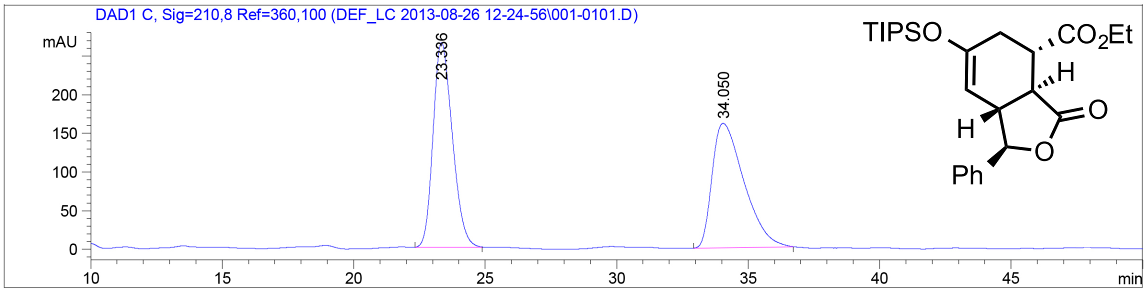


```
Signal 3: DAD1 C, Sig=210,8 Ref=360,100

Peak RetTime Type  Width     Area      Height     Area
  #   [min]        [min]   [mAU*s]     [mAU]        %
----|-------|----|-------|----------|----------|--------|
   1  23.336 BB     0.8007 1.34848e4   264.52606  49.8611
   2  34.050 BB     1.2792 1.35599e4   160.89601  50.1389

Totals :                   2.70446e4   425.42207
```

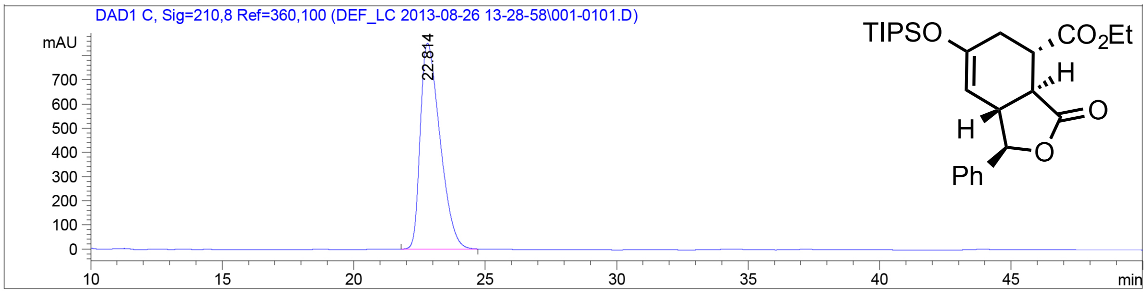


```
Signal 3: DAD1 C, Sig=210,8 Ref=360,100

Peak RetTime Type  Width     Area      Height     Area
  #   [min]        [min]   [mAU*s]     [mAU]        %
----|-------|----|-------|----------|----------|--------|
   1  22.814 BB     0.8057 4.43888e4   852.21271 100.0000

Totals :                   4.43888e4   852.21271
```

Determination of enantiomeric excess of bicyclic γ-lactone (+)-14e'':

Chiral HPLC analysis of bicyclic γ-lactone (+)-14e'': Chiralcel OD-H column: hexanes:^{i}PrOH = 95:05, flow rate 0.5 mL/min, λ = 210 nm: t_{minor} = 21.7 min, t_{major} = 37.0 min; 99% *ee*.

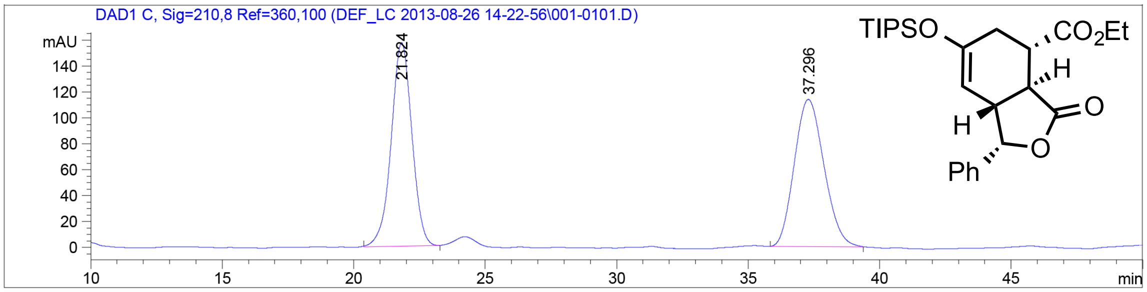

Signal 3: DAD1 C, Sig=210,8 Ref=360,100

Peak #	RetTime [min]	Type	Width [min]	Area [mAU*s]	Height [mAU]	Area %
1	21.824	BB	0.8517	8578.78125	156.95447	49.0045
2	37.296	BB	1.1605	8927.34082	113.63794	50.9955

Totals : 1.75061e4 270.59241

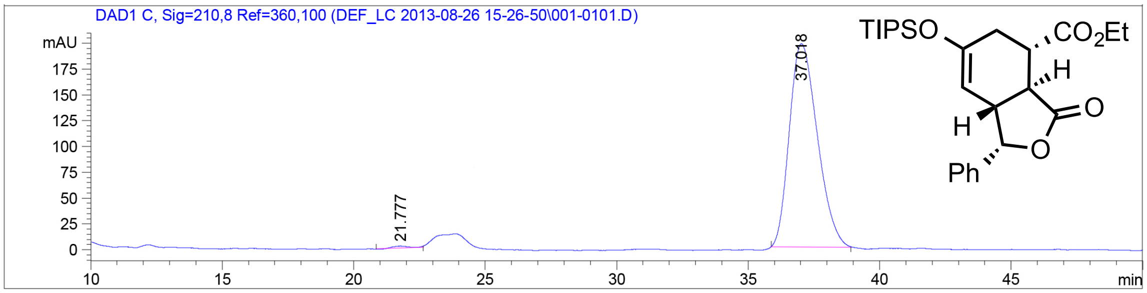

Signal 3: DAD1 C, Sig=210,8 Ref=360,100

Peak #	RetTime [min]	Type	Width [min]	Area [mAU*s]	Height [mAU]	Area %
1	21.777	MM	0.6535	7.68978	1.96131e-1	0.4870
2	37.018	BB	1.0976	1571.21863	21.17800	99.5130

Totals : 1578.90841 21.37413

Determination of enantiomeric excess of bicyclic γ-lactone (–)-14e''':

Chiral HPLC analysis of bicyclic γ-lactone (–)-14e''': Chiralcel AS-H column: hexanes:^{i}PrOH = 95:05, flow rate 0.5 mL/min, λ = 210 nm: t_{major} = 15.5 min, t_{minor} = 28.2 min; 97% *ee*.

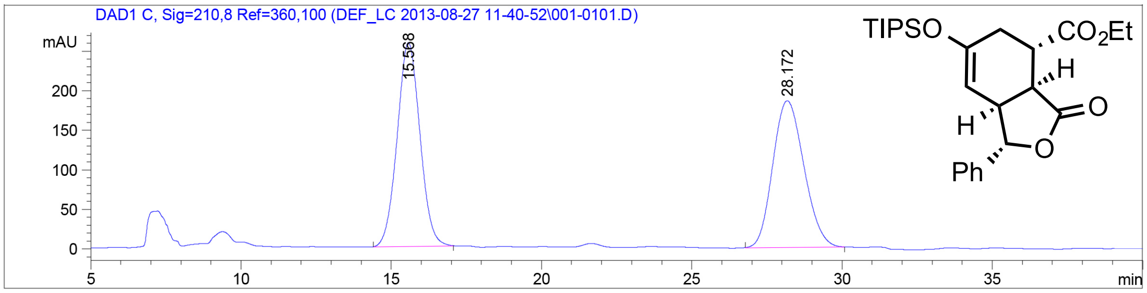

Signal 3: DAD1 C, Sig=210,8 Ref=360,100

Peak #	RetTime [min]	Type	Width [min]	Area [mAU*s]	Height [mAU]	Area %
1	15.568	BB	0.8089	1.35702e4	257.45016	50.4264
2	28.172	BB	1.1175	1.33407e4	185.39282	49.5736
Totals :				2.69109e4	442.84299	

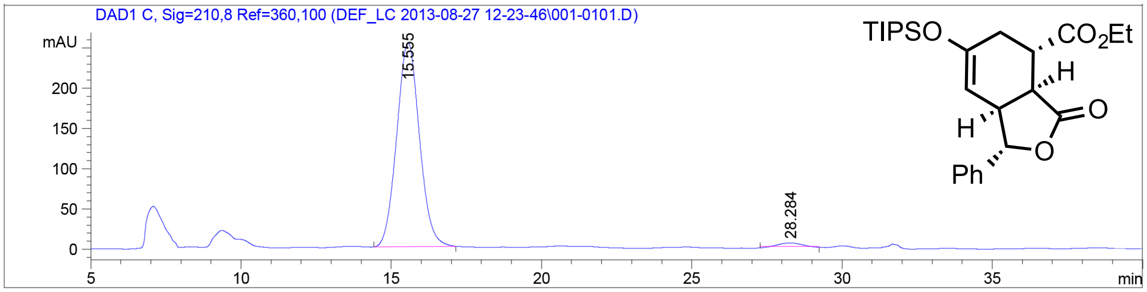

Signal 3: DAD1 C, Sig=210,8 Ref=360,100

Peak #	RetTime [min]	Type	Width [min]	Area [mAU*s]	Height [mAU]	Area %
1	15.555	BB	0.8209	1.33634e4	253.56432	98.3984
2	28.284	MM	0.8160	217.51129	4.44277	1.6016
Totals :				1.35809e4	258.00708	

References:

1. D. Seebach, R. Imwinkelried and G. Stucky, *Helv. Chim. Acta.*, **1987**, *70*, 448-464.
2. M. E. Abbasov, B. M. Hudson, D. J. Tantillo and D. Romo, *J. Am. Chem. Soc.*, **2014**, *136*, 4492-4495.
3. M. T. Crimmins and J. G. Lever, *Tetrahedron Lett.*, **1986** , *27*, 291-294.
4. C. Morrill, G. L. Beutner and R. H. Grubbs, *J. Org. Chem.*, **2006**, *71*, 7813-7825.
5. Gaussian 09, Revision D.01, Frisch, M. J.; Trucks, G. W.; Schlegel, H. B.; Scuseria, G. E.; Robb, M. A.; Cheeseman, J. R.; Scalmani, G.; Barone, V.; Mennucci, B.; Petersson, G. A.; Nakatsuji, H.; Caricato, M.; Li, X.; Hratchian, H. P.; Izmaylov, A. F.; Bloino, J.; Zheng, G.; Sonnenberg, J. L.; Hada, M.; Ehara, M.; Toyota, K.; Fukuda, R.; Hasegawa, J.; Ishida, M.; Nakajima, T.; Honda, Y.; Kitao, O.; Nakai, H.; Vreven, T.; Montgomery, Jr., J. A.; Peralta, J. E.; Ogliaro, F.; Bearpark, M.; Heyd, J. J.; Brothers, E.; Kudin, K. N.; Staroverov, V. N.; Kobayashi, R.; Normand, J.; Raghavachari, K.; Rendell, A.; Burant, J. C.; Iyengar, S. S.; Tomasi, J.; Cossi, M.; Rega, N.; Millam, N. J.; Klene, M.; Knox, J. E.; Cross, J. B.; Bakken, V.; Adamo, C.; Jaramillo, J.; Gomperts, R.; Stratmann, R. E.; Yazyev, O.; Austin, A. J.; Cammi, R.; Pomelli, C.; Ochterski, J. W.; Martin, R. L.; Morokuma, K.; Zakrzewski, V. G.; Voth, G. A.; Salvador, P.; Dannenberg, J. J.; Dapprich, S.; Daniels, A. D.; Farkas, Ö.; Foresman, J. B.; Ortiz, J. V.; Cioslowski, J.; Fox, D. J. *Gaussian, Inc.*, Wallingford CT, **2009**.
6. (a) Becke, A. D. *J. Chem. Phys.* **1993**, *98*, 1372-1377. (b) Becke, A. D. *J. Chem. Phys.* **1993**, *98*, 5648-5652. (c) Lee, C.; Yang, W.; Parr, R. G. *Phys. Rev. B* **1988**, *37*, 785-789. (d) Stephens, P. J.; Devlin, F. J.; Chabalowski, C. F.; Frisch, M. J. *J. Phys. Chem.* **1994**, *98*, 11623-11627. (e) Tirado-Rives, J.; Jorgensen, W. L. *J. Chem. Theory Comput.* **2008**, *4*, 297-306. (f) Grimme, S.; Antony, J.; Ehrlich, S.; Krieg, H. *J. Chem. Phys.* **2010**, *132*, 154104.
7. Chai, J.-D.; Head-Gordon, M. *Phys. Chem. Chem. Phys.* **2008**, *10*, 6615-6620.
8. Zhao, Y.; Truhlar, D. G. *Theor. Chem. Acc.* **2008**, *120*, 215-241.

Stereodivergent, Diels-Alder-Initiated Organocascades Employing α,β–Unsaturated Acylammonium Salts: Scope, Mechanism, and Application

Mikail E. Abbasov,[†] Brandi M. Hudson,[‡] Dean J. Tantillo[‡,*] and Daniel Romo[†,*]

[†]*Department of Chemistry and Biochemistry, Baylor University, One Bear Place 97348, Waco, Texas 76798, United States*

[‡]*Department of Chemistry, University of California–Davis, One Shields Avenue, Davis, California 95616, United States*

Table of Contents, *cont.*

• ^{1}H and ^{13}C NMR spectra of new compounds S2–S26

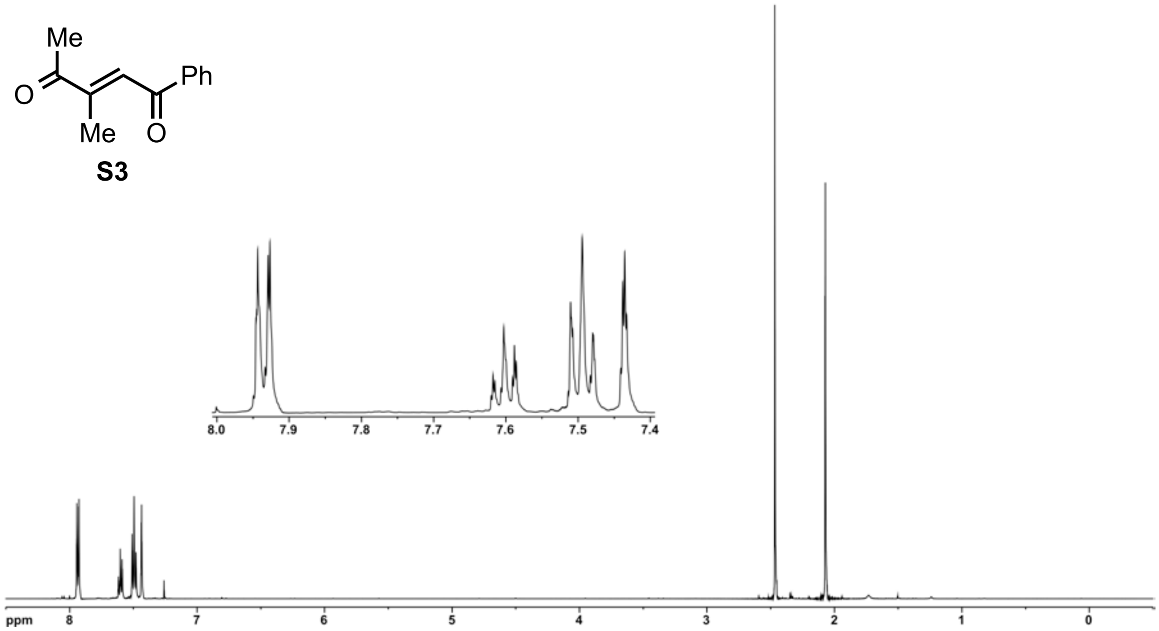

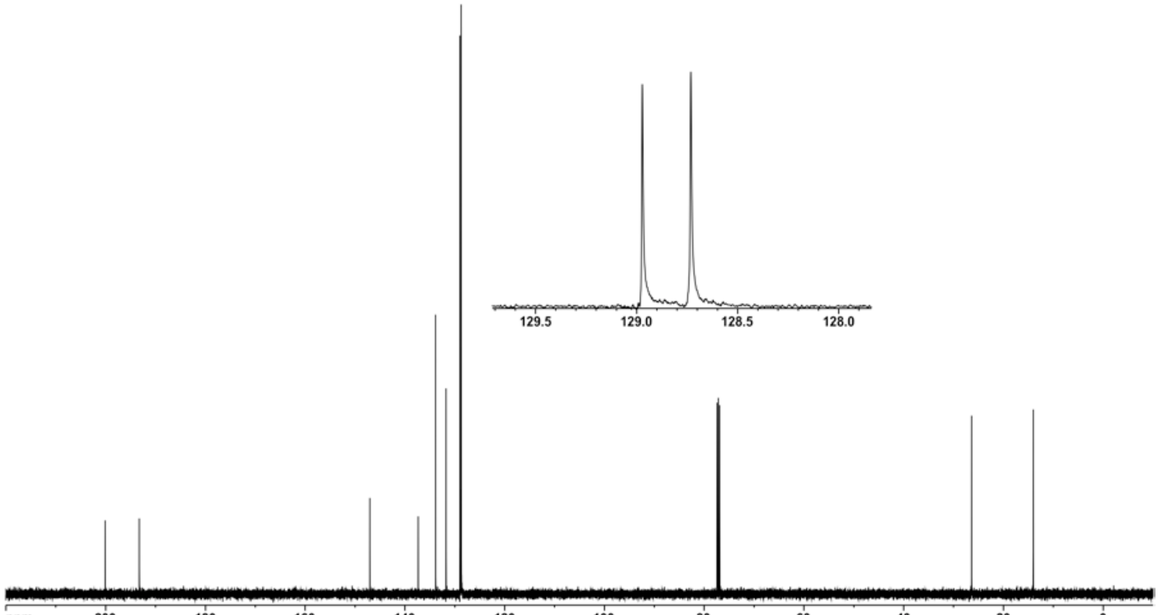

^{1}H (500 MHz) and ^{13}C NMR (125 MHz) spectra of diketone **S3** in $CDCl_3$

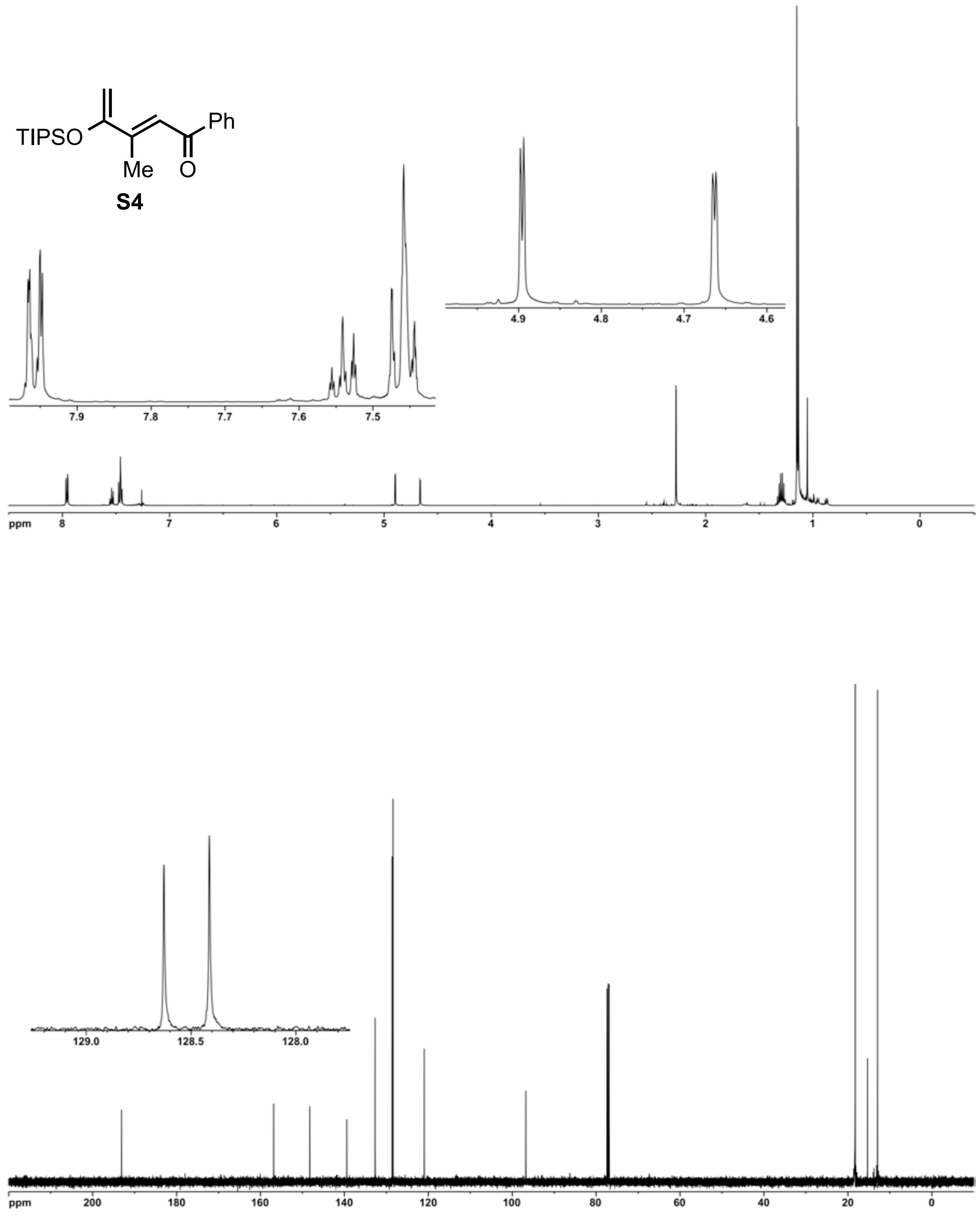

^{1}H (500 MHz) and ^{13}C NMR (125 MHz) spectra of diene **S4** in $CDCl_3$

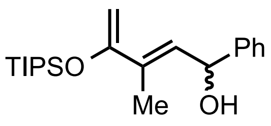

(±)-**13a**

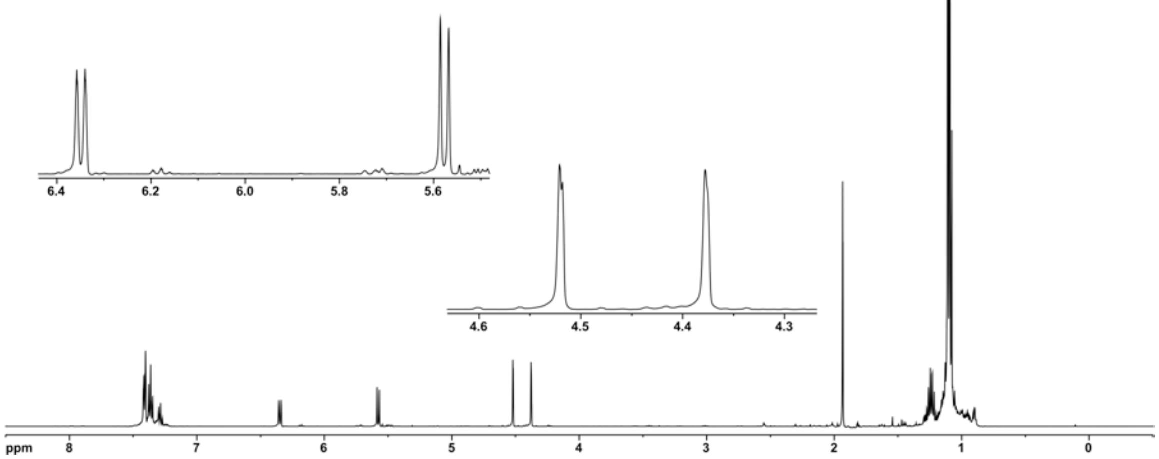

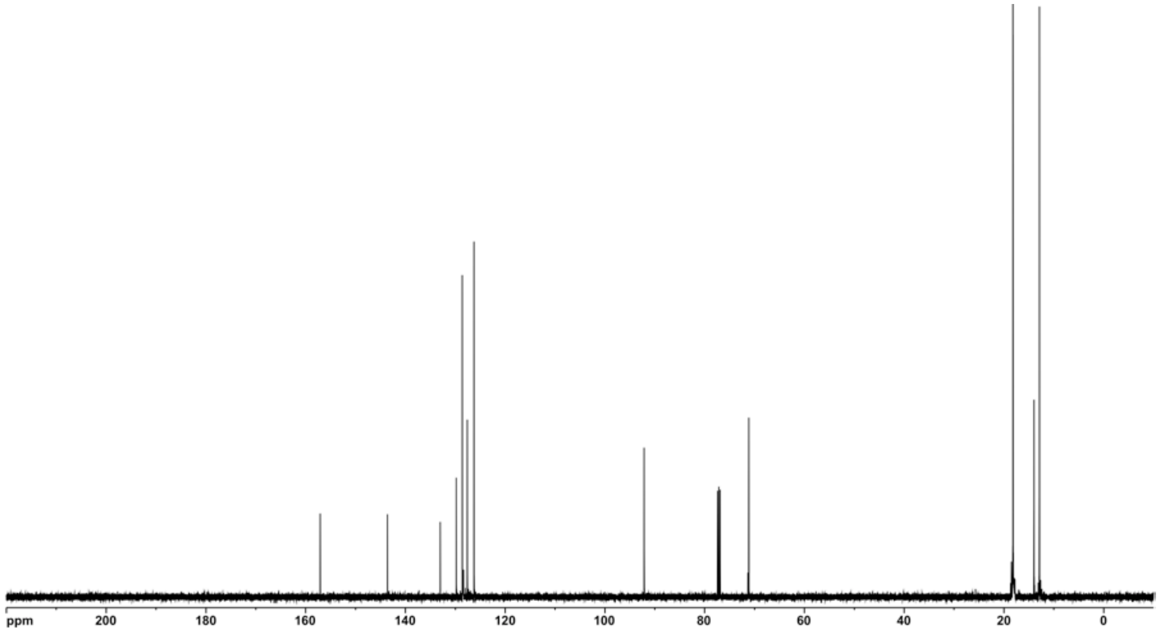

^{1}H (500 MHz) and ^{13}C NMR (125 MHz) spectra of silyloxydiene alcohol (±)-**13a** in $CDCl_3$

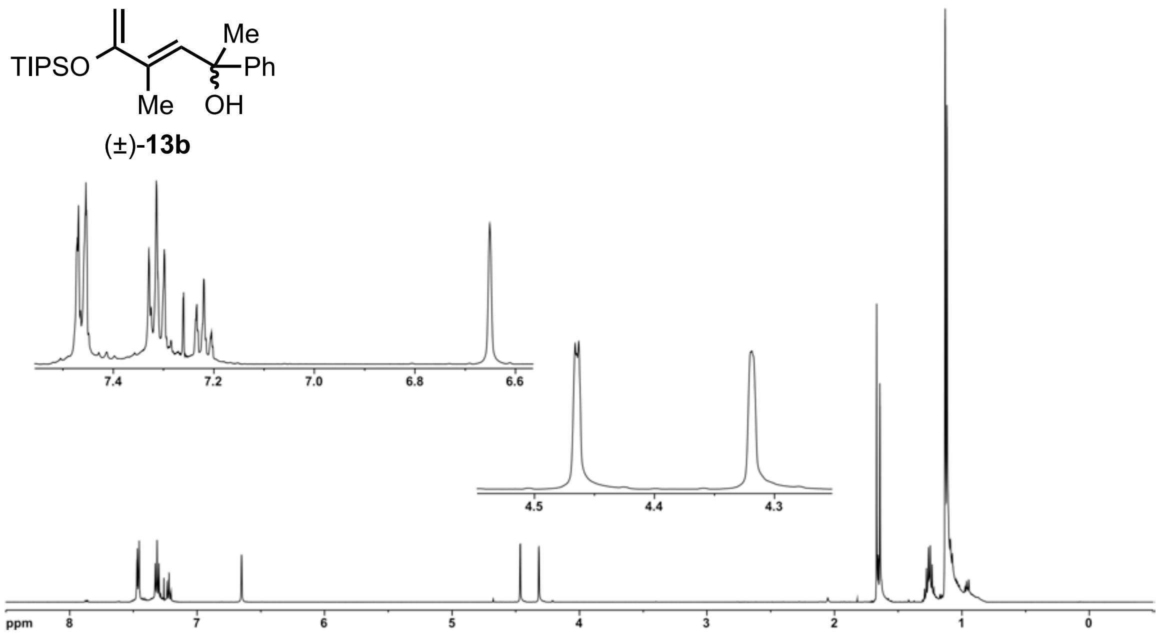

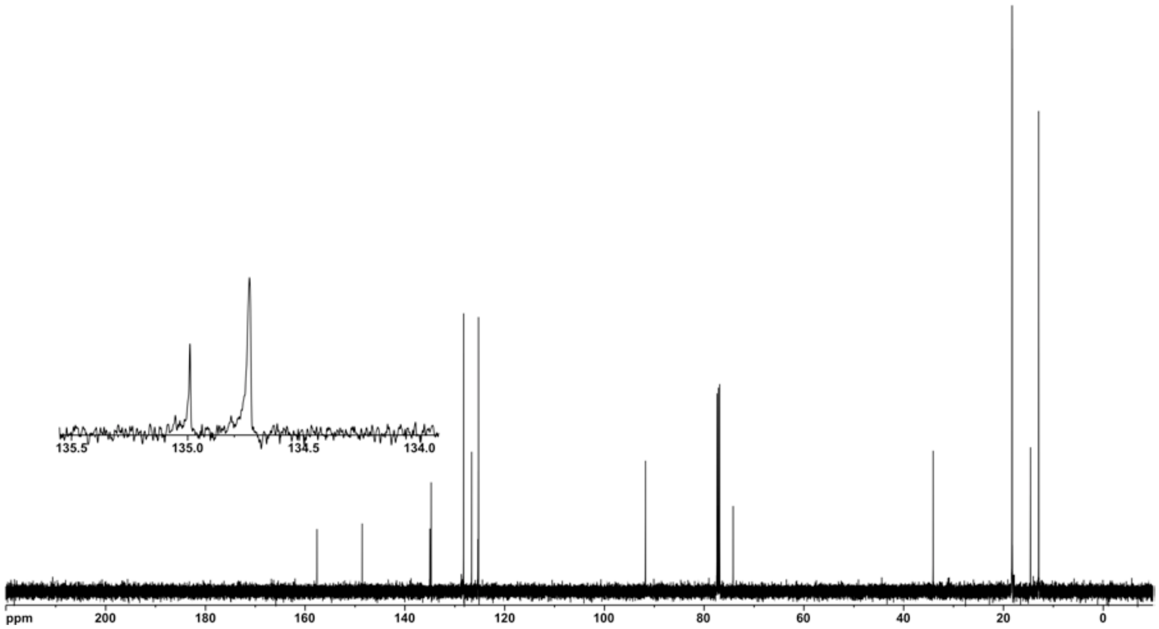

^{1}H (500 MHz) and ^{13}C NMR (125 MHz) spectra of silyloxydiene alcohol (±)-**13b** in $CDCl_3$

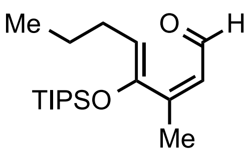

S6

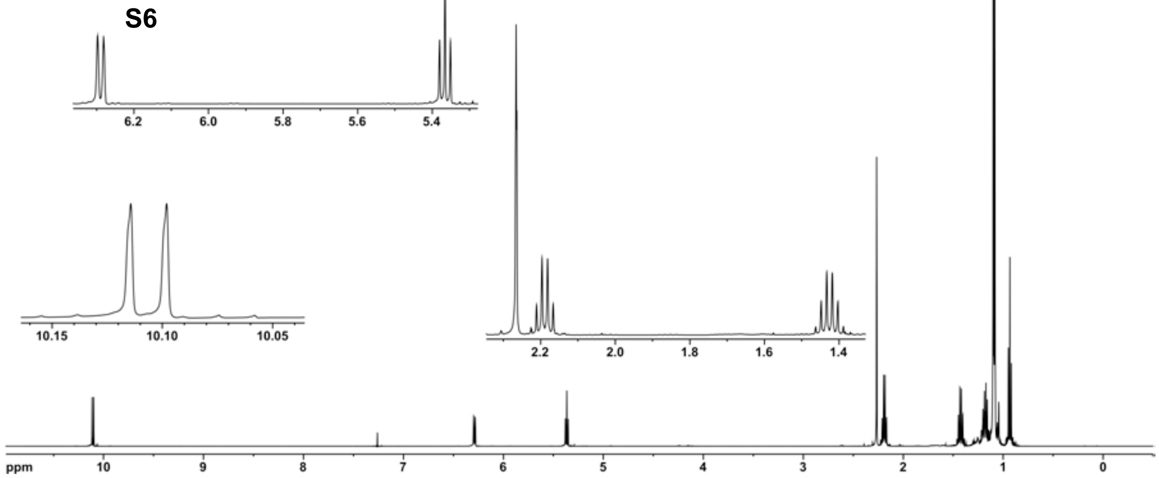

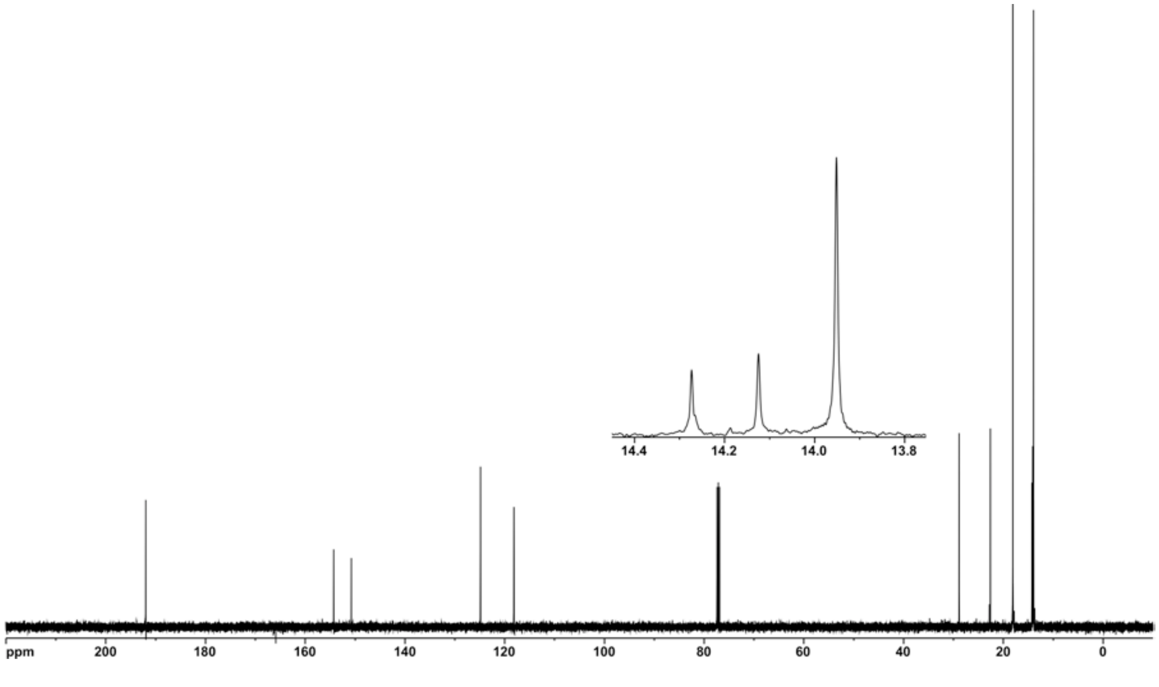

^{1}H (500 MHz) and ^{13}C NMR (125 MHz) spectra of aldehyde **S6** in $CDCl_3$

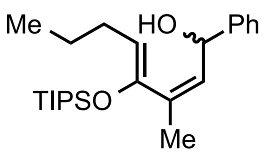

(±)-**13c**

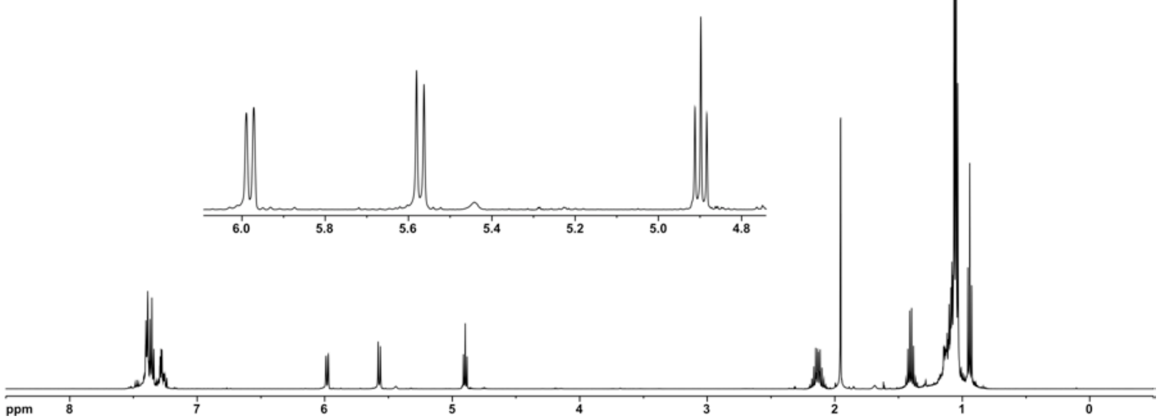

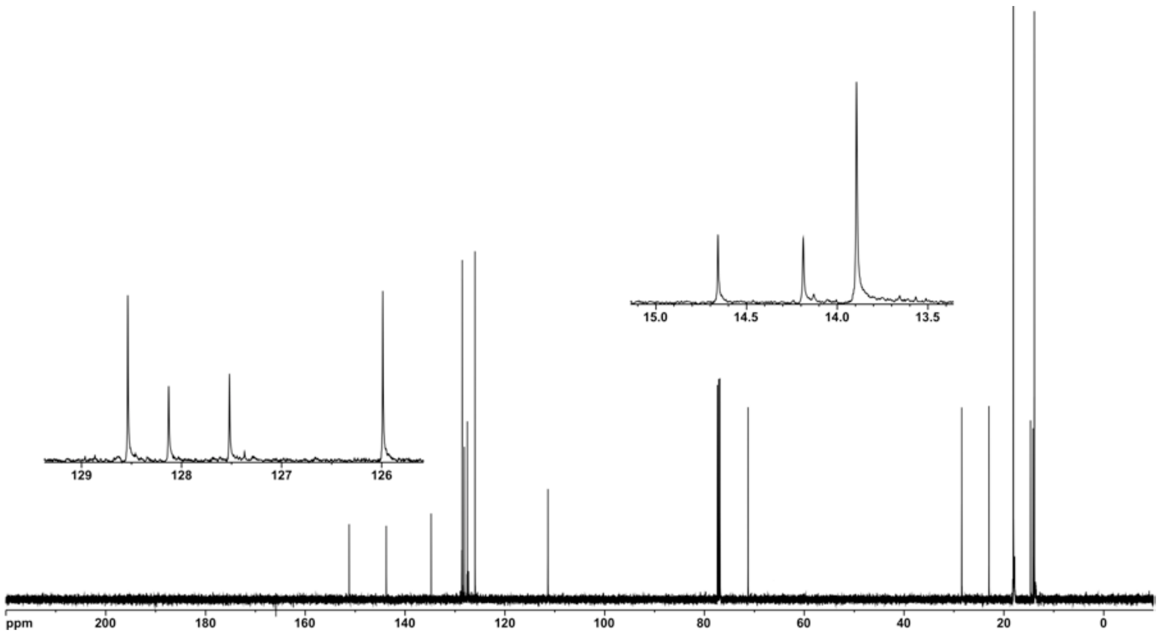

^{1}H (500 MHz) and ^{13}C NMR (125 MHz) spectra of silyloxydiene alcohol (±)-**13c** in $CDCl_3$

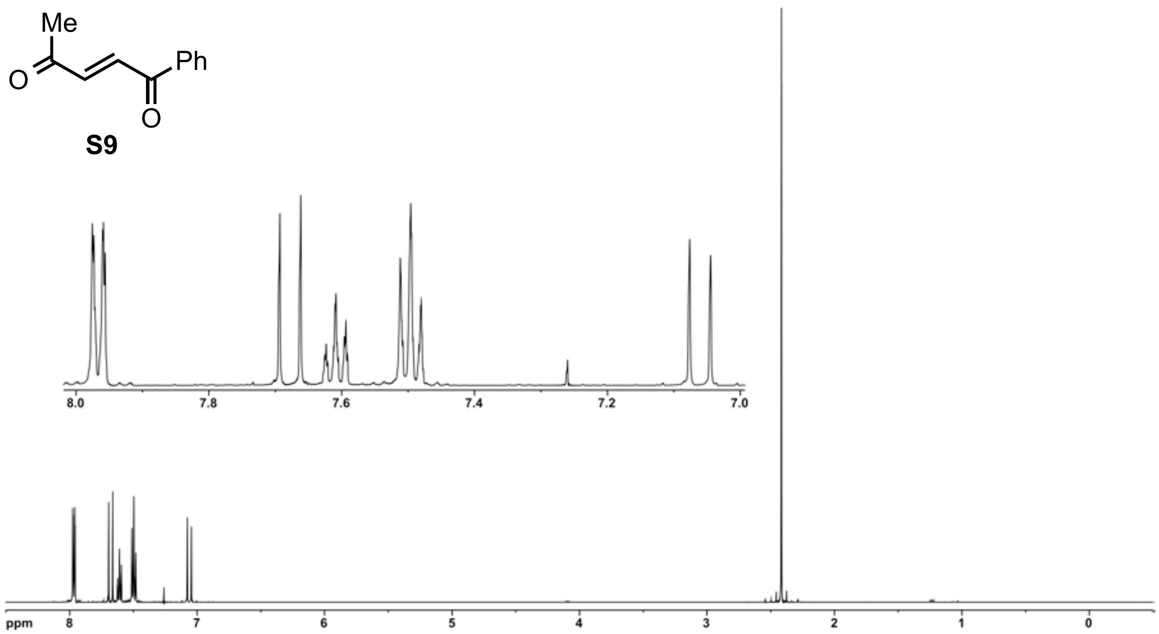

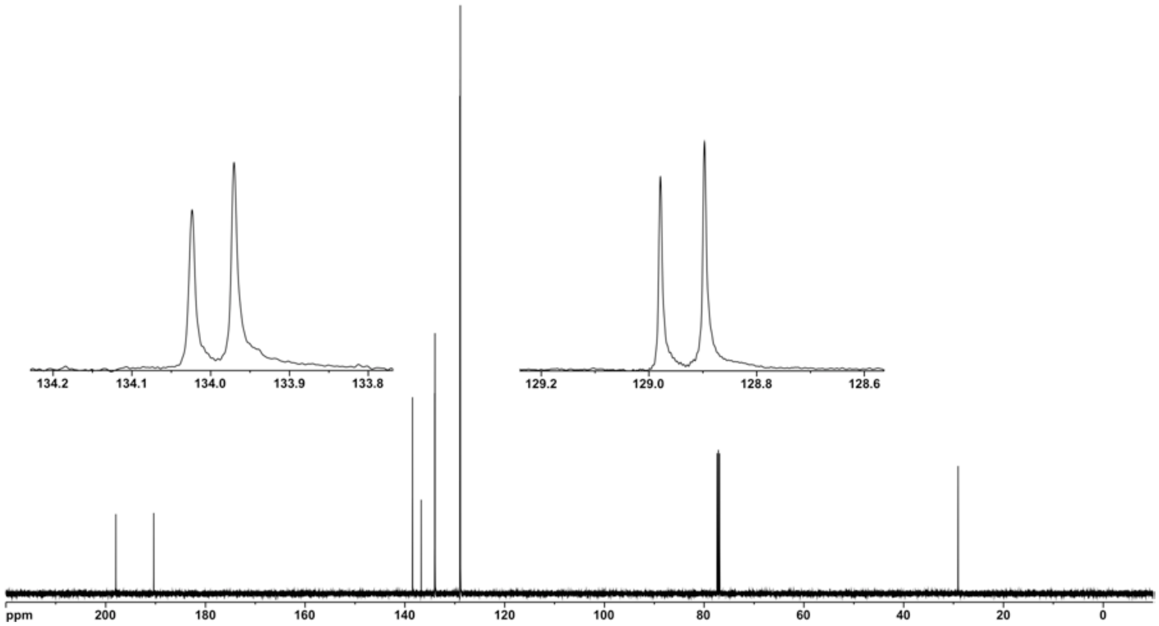

^{1}H (500 MHz) and ^{13}C NMR (125 MHz) spectra of diketone **S9** in $CDCl_3$

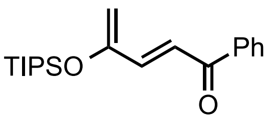

S10

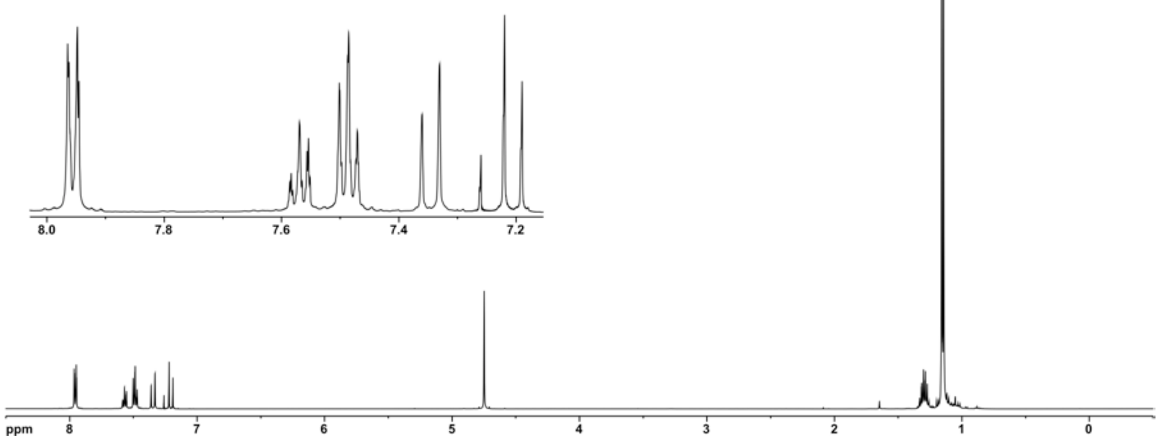

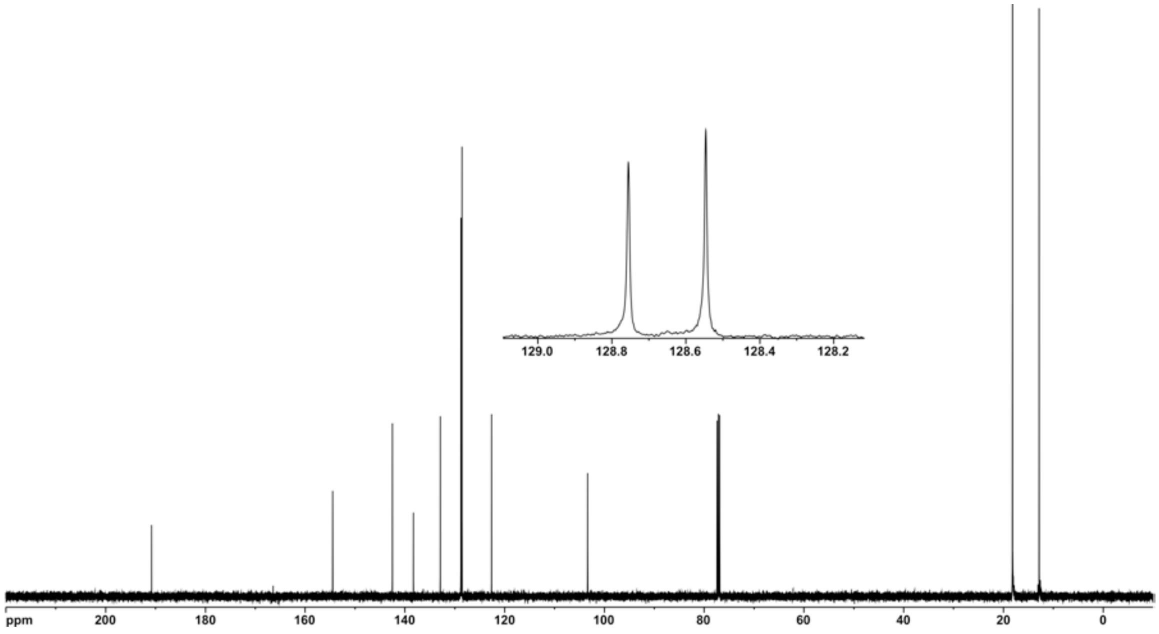

^{1}H (500 MHz) and ^{13}C NMR (125 MHz) spectra of diene **S10** in $CDCl_3$

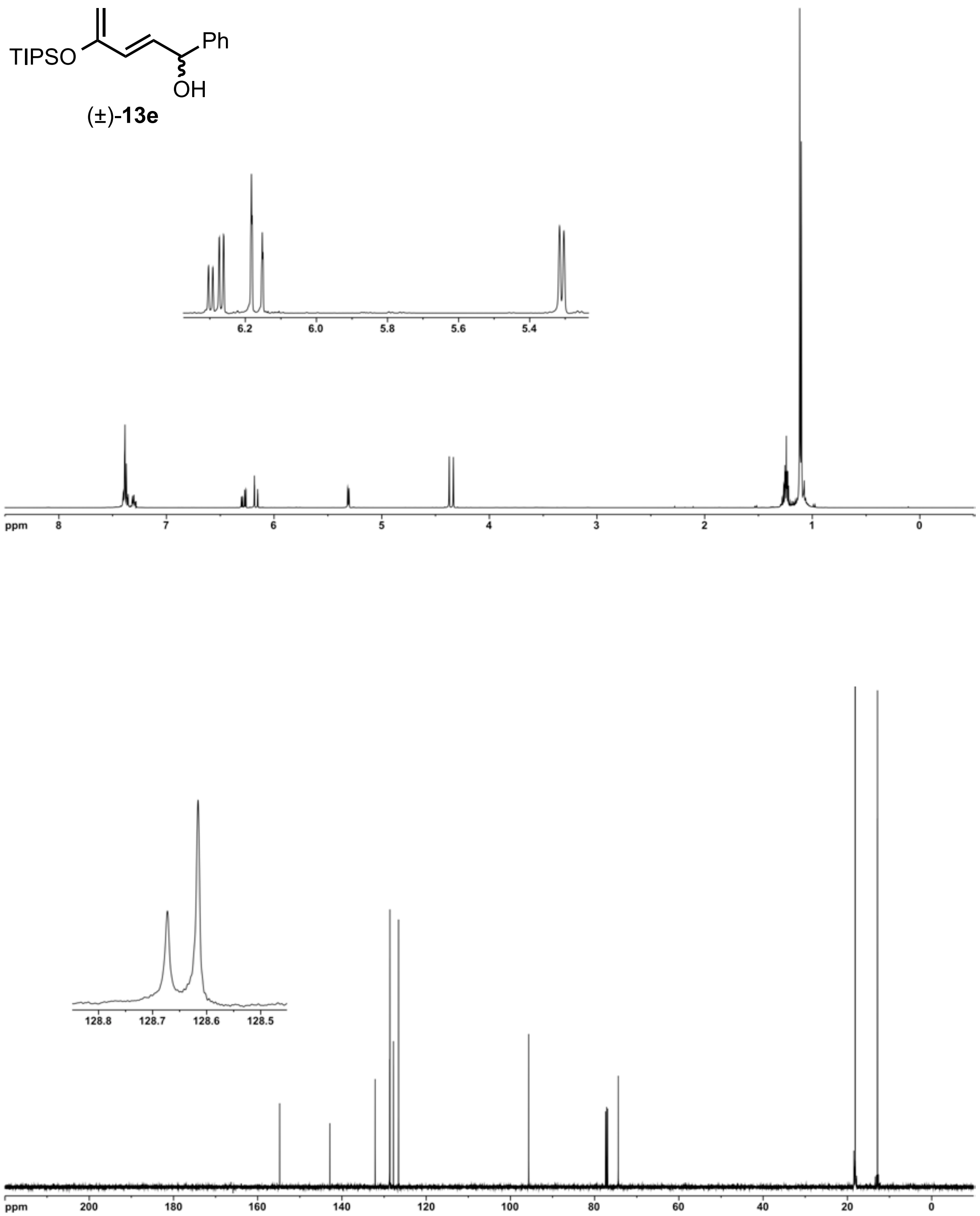

^{1}H (500 MHz) and ^{13}C NMR (125 MHz) spectra of silyloxydiene alcohol (±)-**13e** in $CDCl_3$

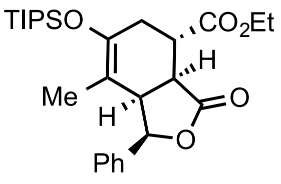

(–)-**14a**

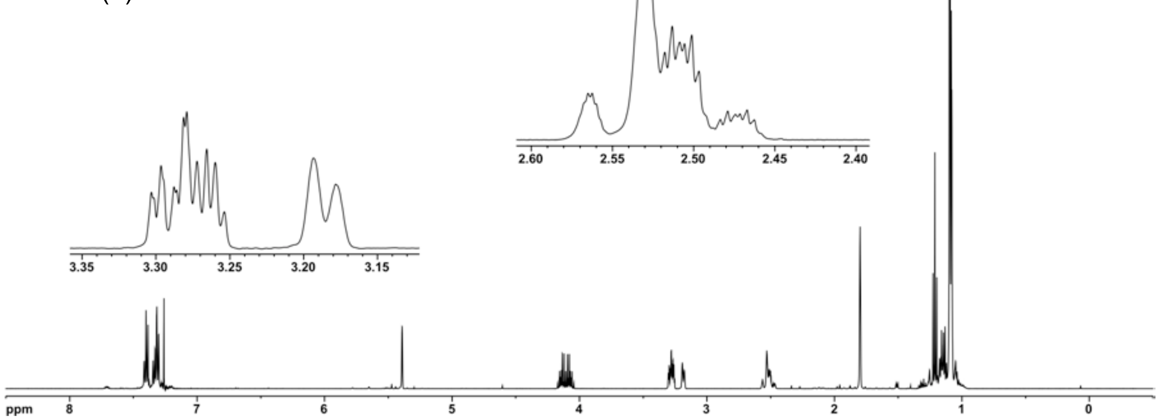

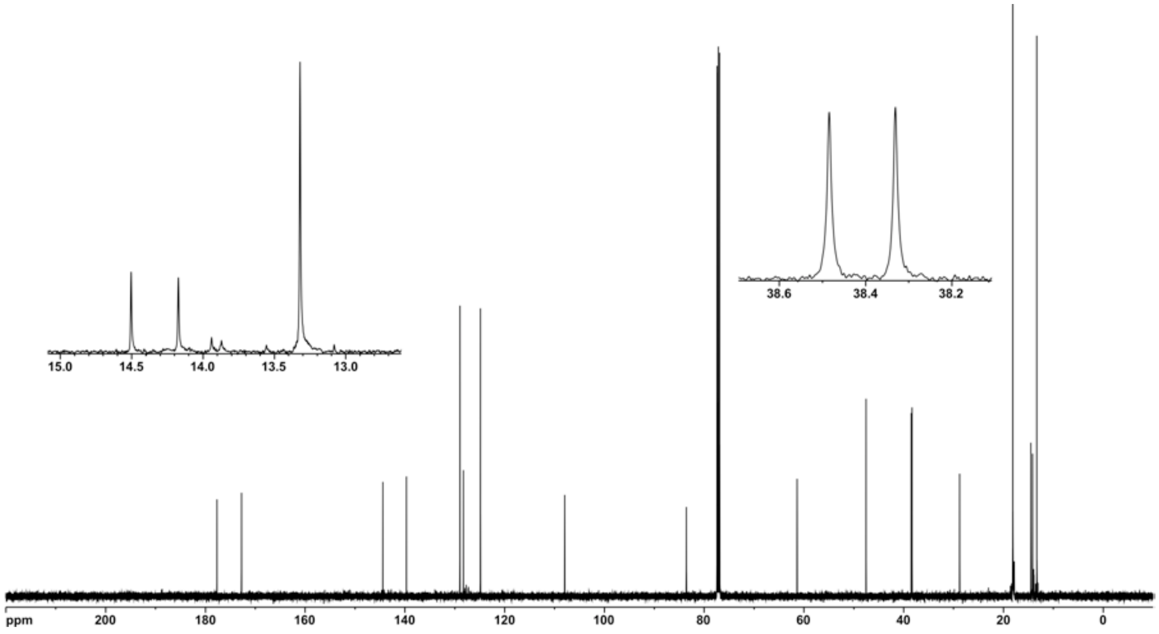

^{1}H (500 MHz) and ^{13}C NMR (125 MHz) spectra of bicyclic γ-lactone (–)-**14a** in $CDCl_3$

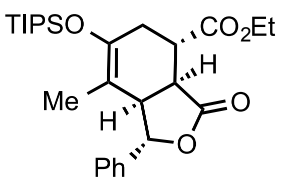

(+)-**14a'**

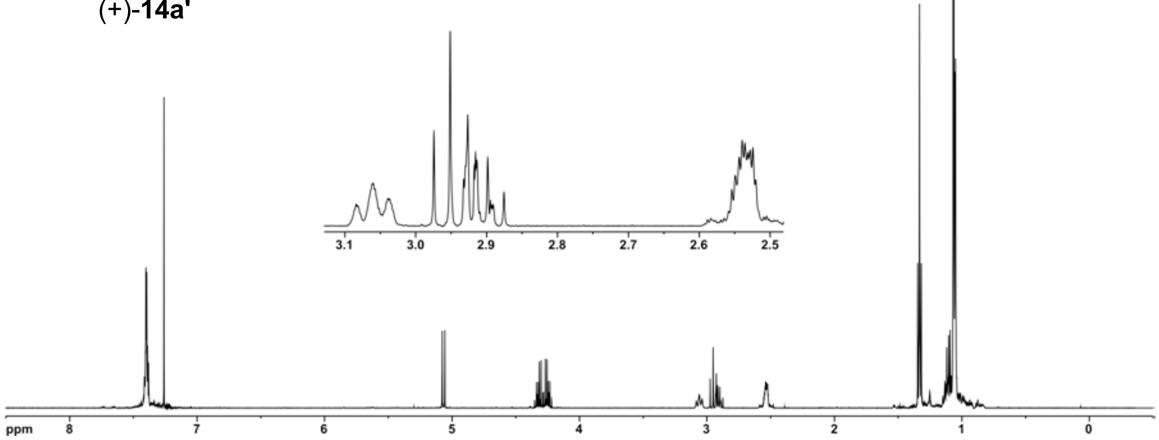

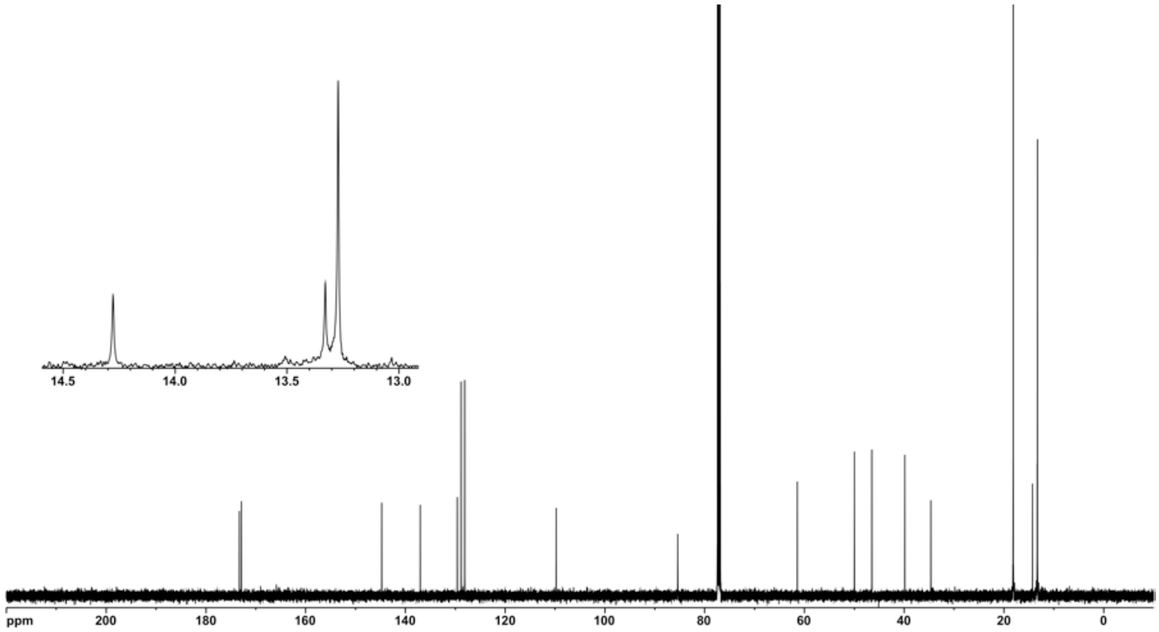

^{1}H (500 MHz) and ^{13}C NMR (125 MHz) spectra of bicyclic γ-lactone (+)-**14a’** in $CDCl_3$

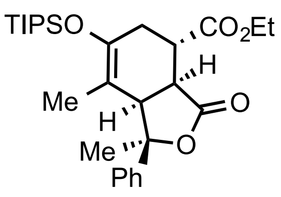

(+)-**14b**

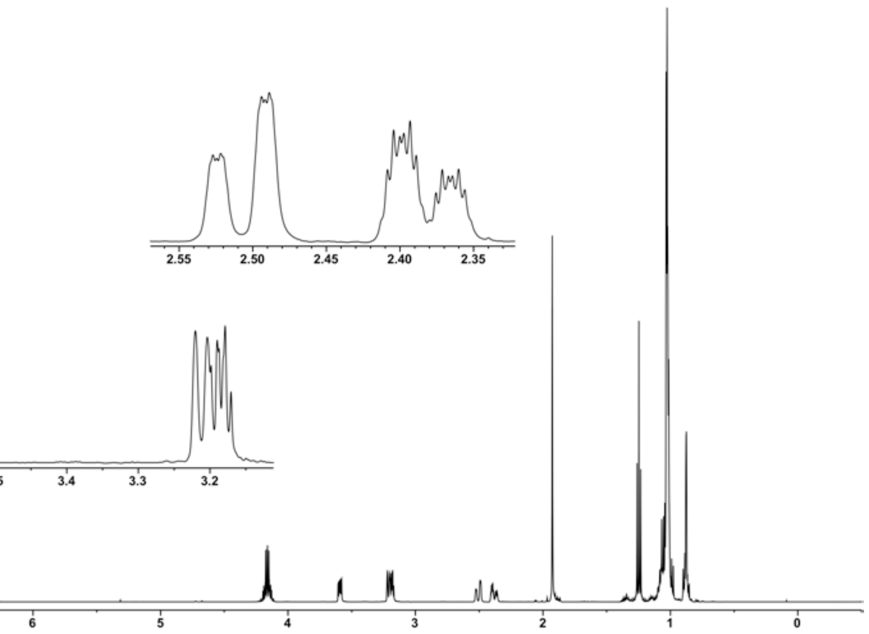

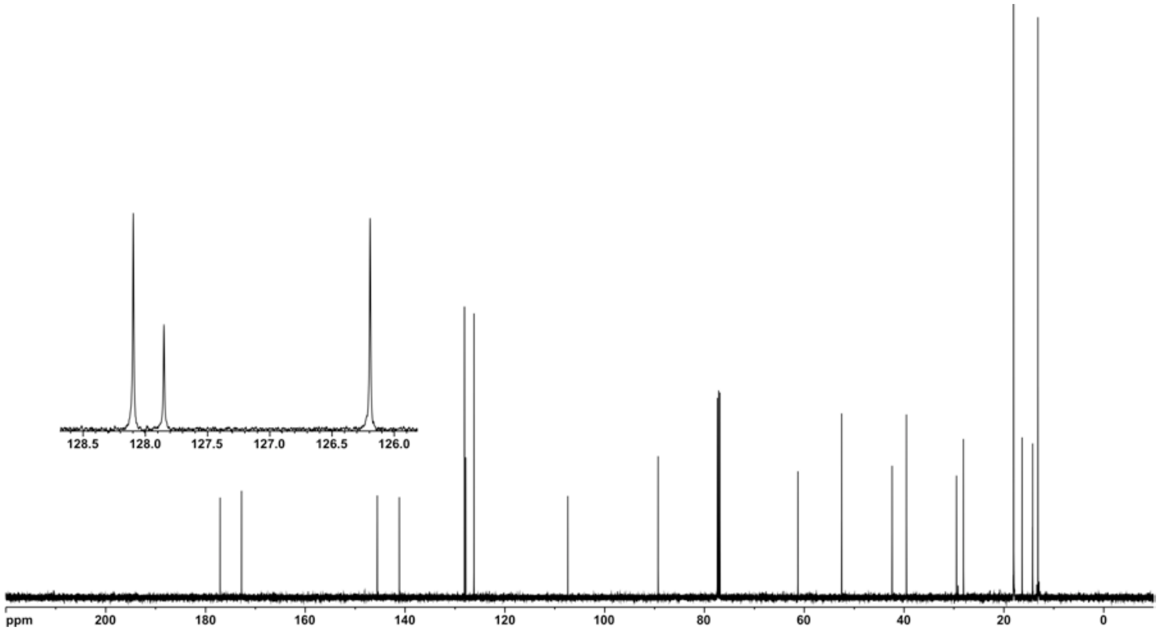

^{1}H (500 MHz) and ^{13}C NMR (125 MHz) spectra of bicyclic γ-lactone (+)-**14b** in $CDCl_3$

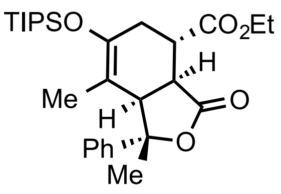

(+)-**14b'**

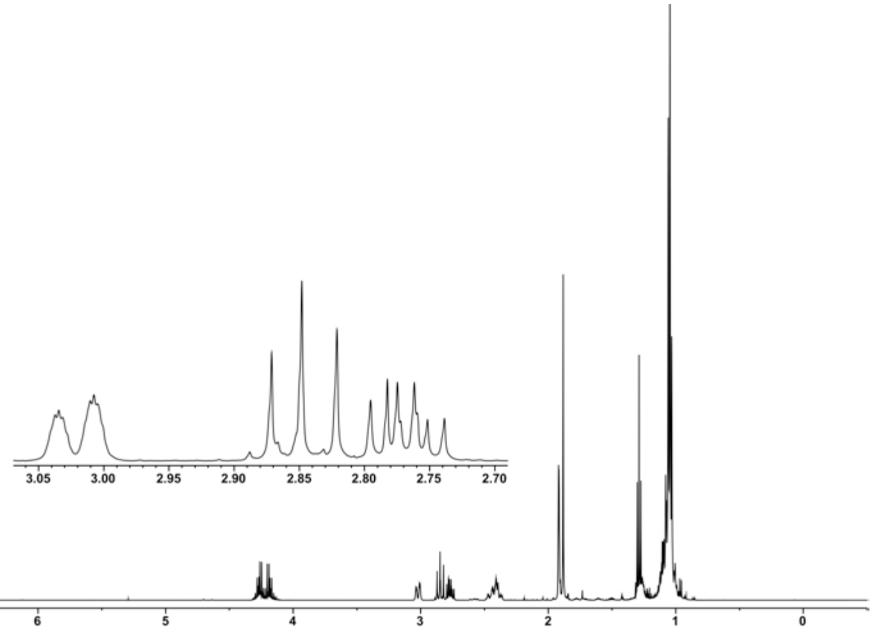

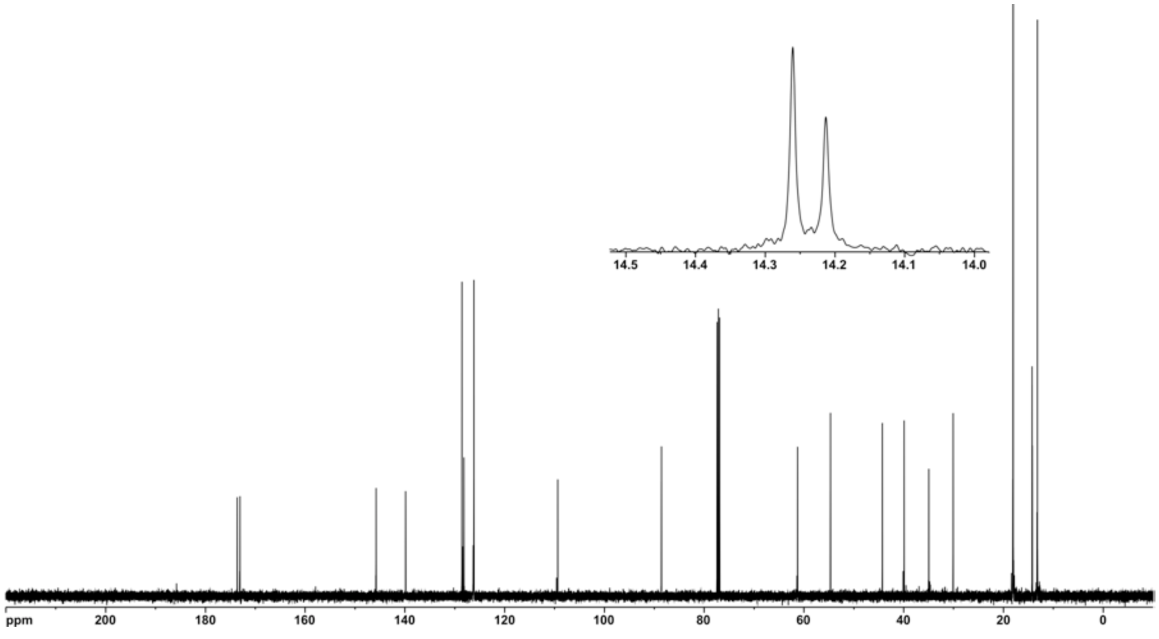

^{1}H (500 MHz) and ^{13}C NMR (125 MHz) spectra of bicyclic γ-lactone (+)-**14b’** in $CDCl_3$

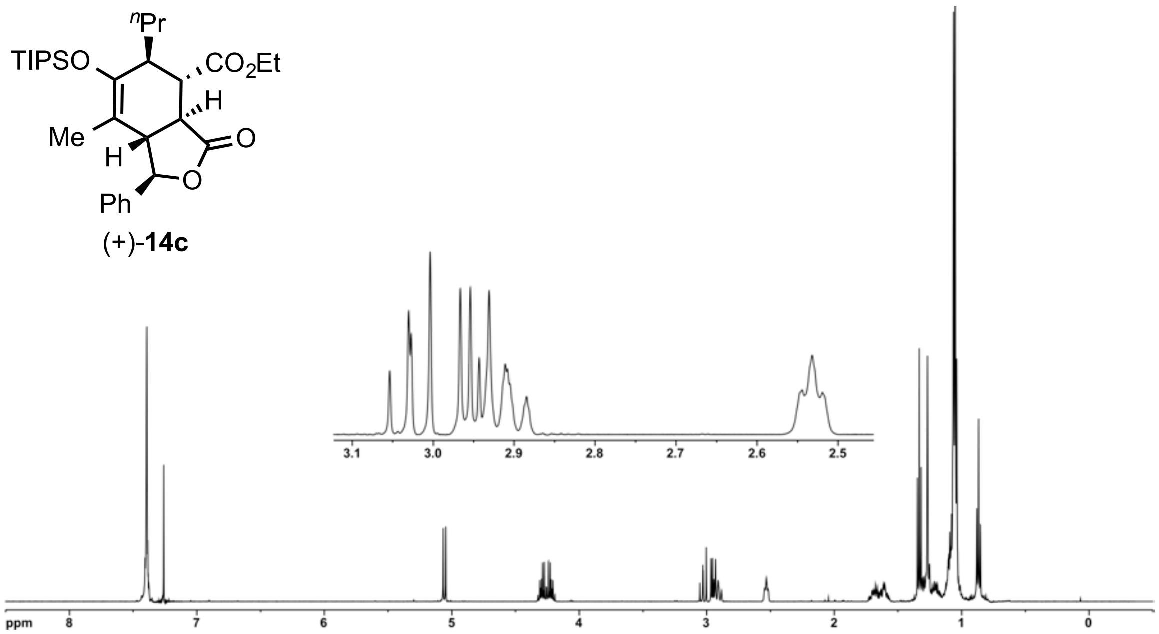

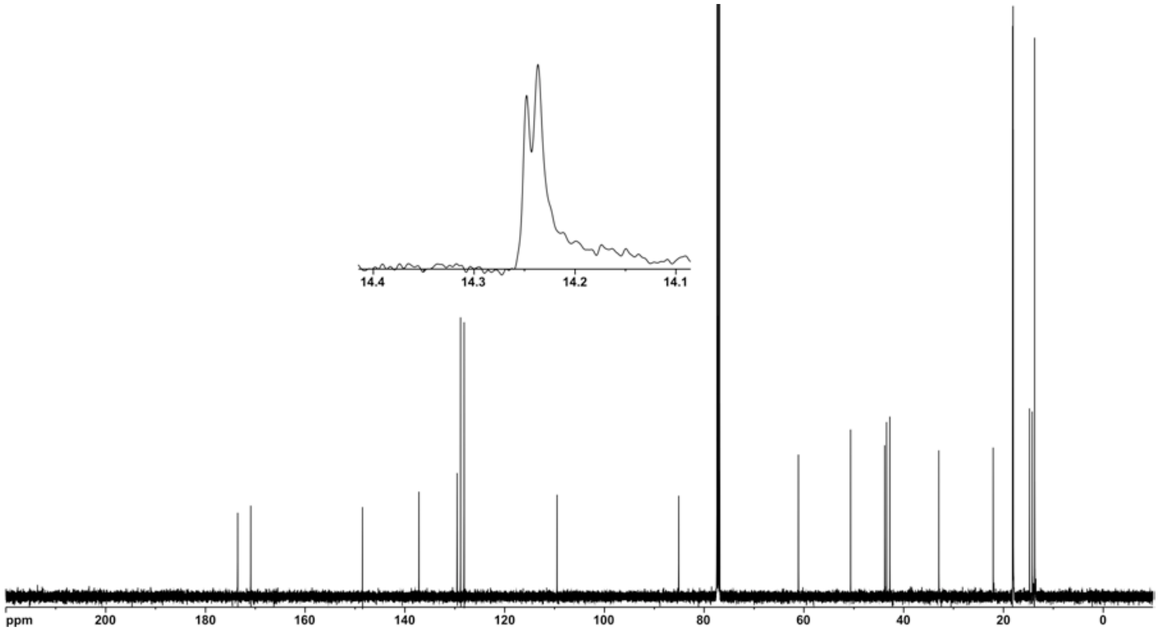

^{1}H (500 MHz) and ^{13}C NMR (125 MHz) spectra of bicyclic γ-lactone (+)-**14c** in $CDCl_3$

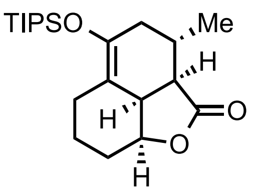

(–)-**14d**

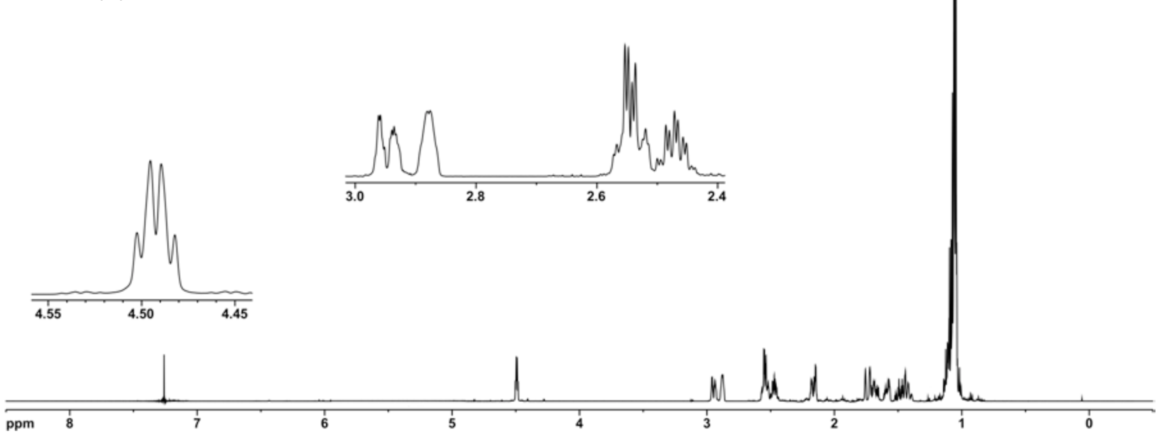

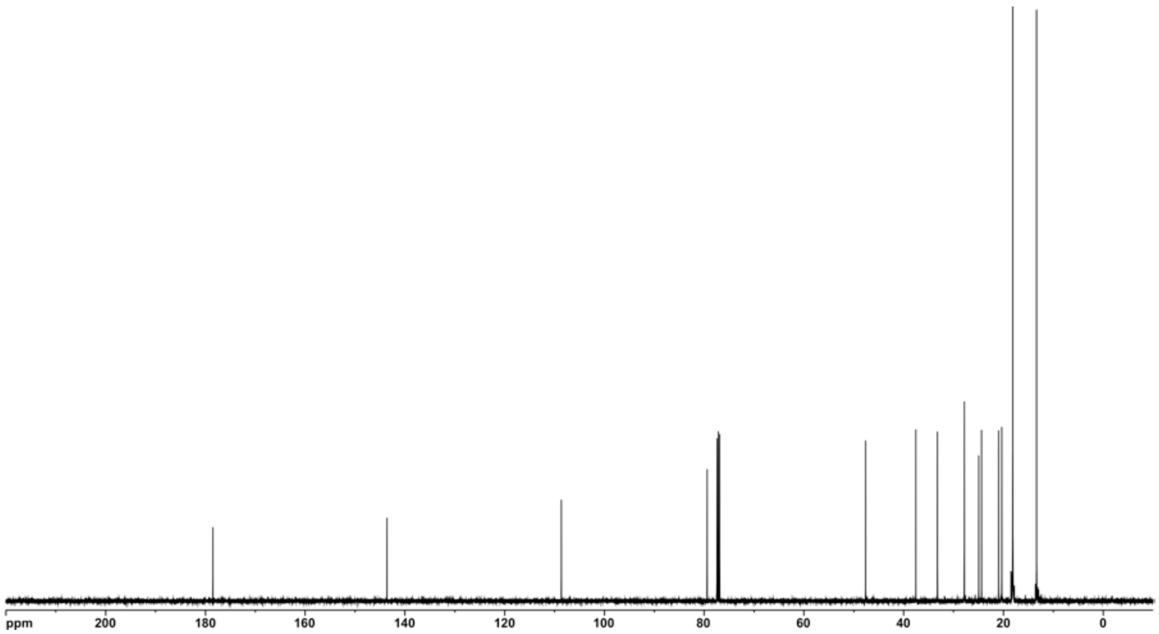

^{1}H (500 MHz) and ^{13}C NMR (125 MHz) spectra of tricyclic γ-lactone (–)-**14d** in $CDCl_3$

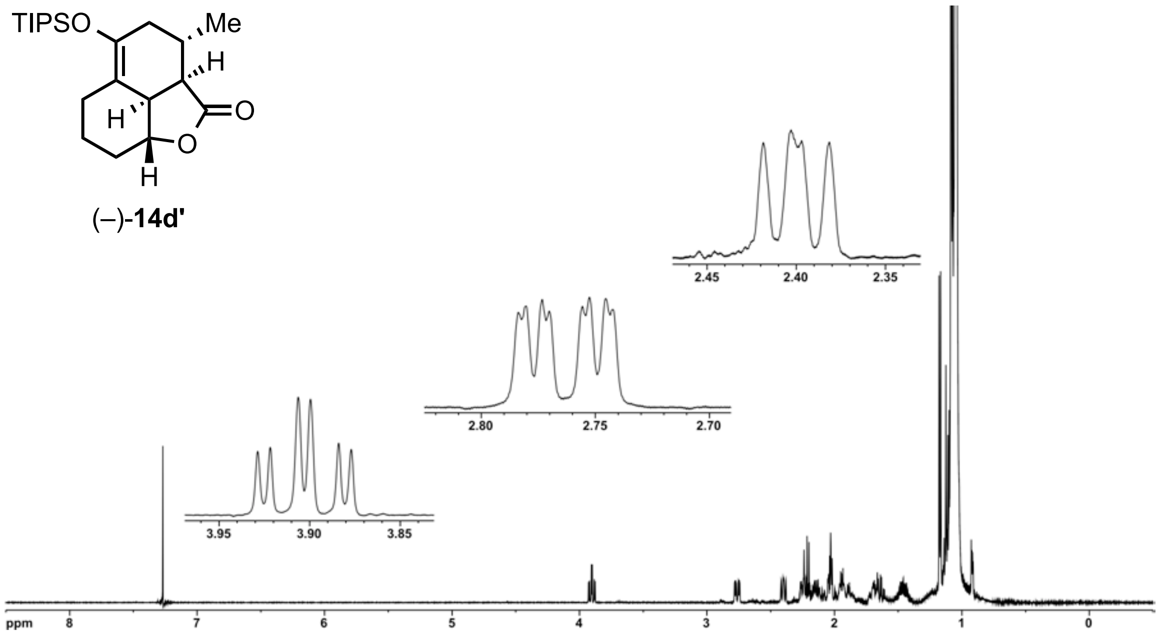

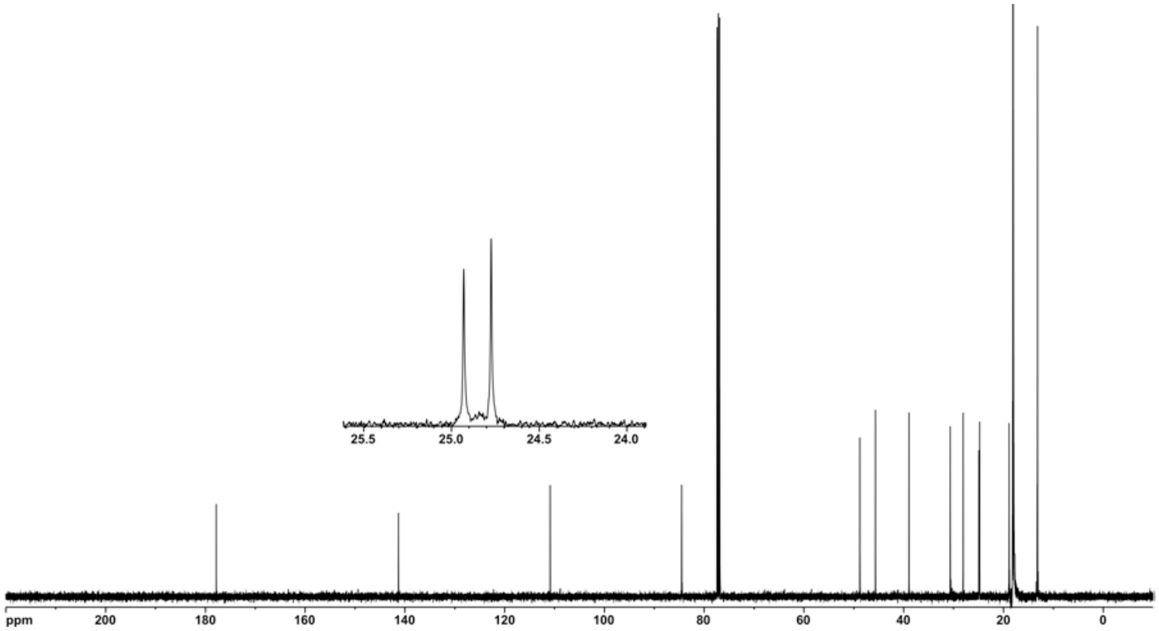

^{1}H (500 MHz) and ^{13}C NMR (125 MHz) spectra of tricyclic γ-lactone (–)-**14d’** in $CDCl_3$

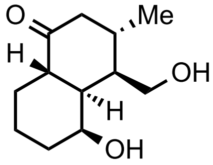
(+)-**26**

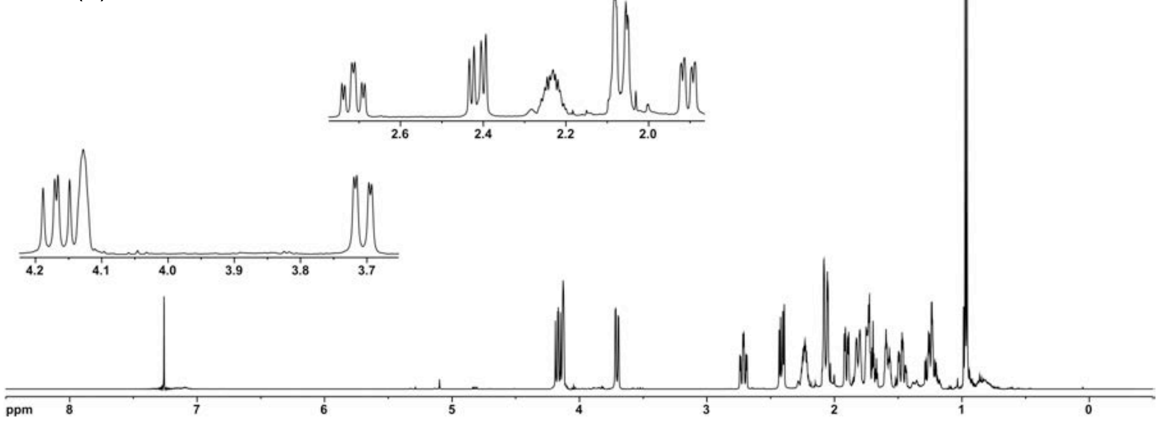

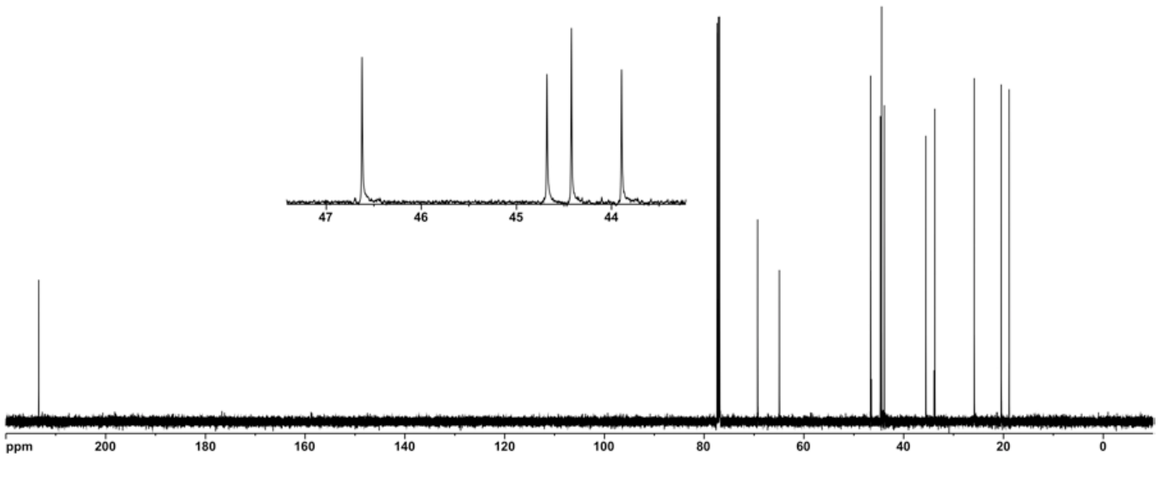

^{1}H (500 MHz) and ^{13}C NMR (125 MHz) spectra of ketone (+)-**26** in $CDCl_3$

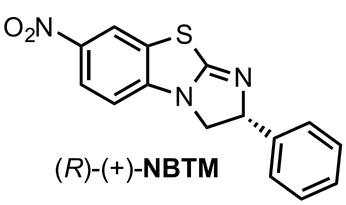

(*R*)-(+)-**NBTM**

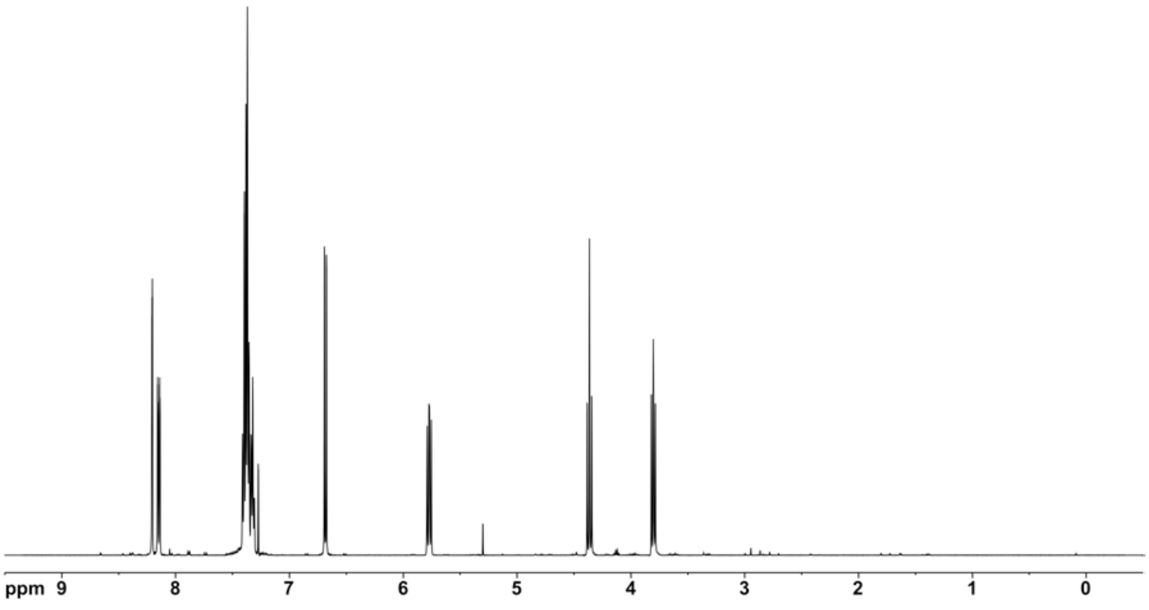

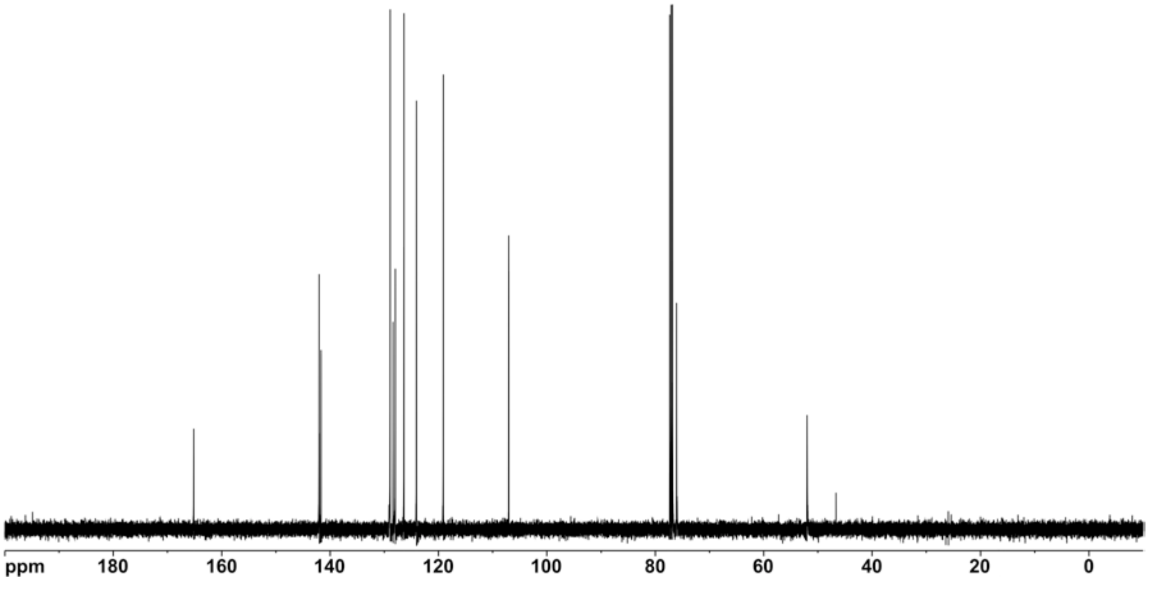

^{1}H (500 MHz) and ^{13}C NMR (125 MHz) spectra of (*R*)-(+)-**NBTM** in $CDCl_3$

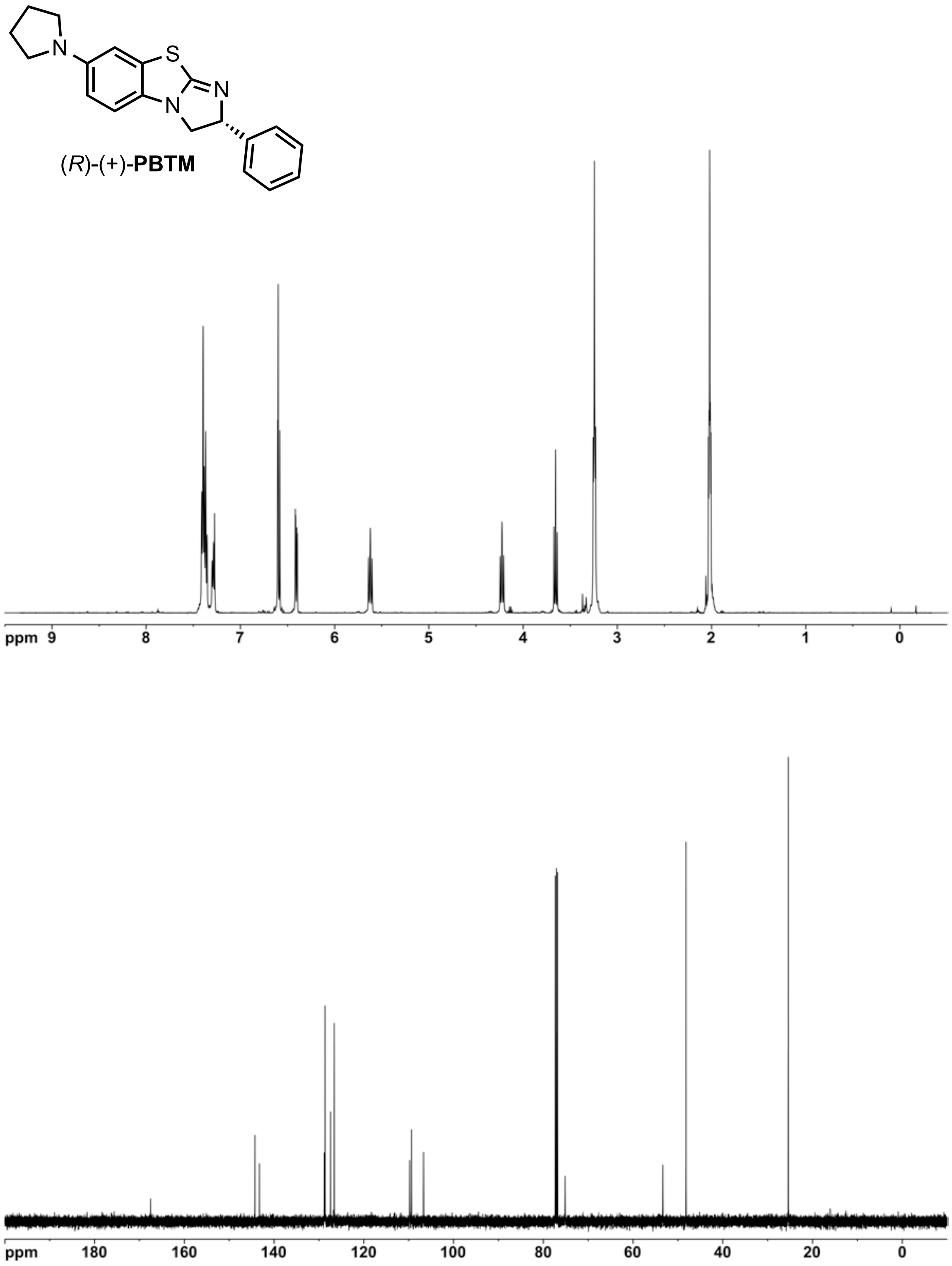

^{1}H (500 MHz) and ^{13}C NMR (125 MHz) spectra of (*R*)-(+)-**PBTM** in $CDCl_3$

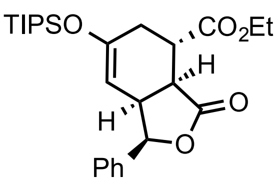

(–)-**14e**

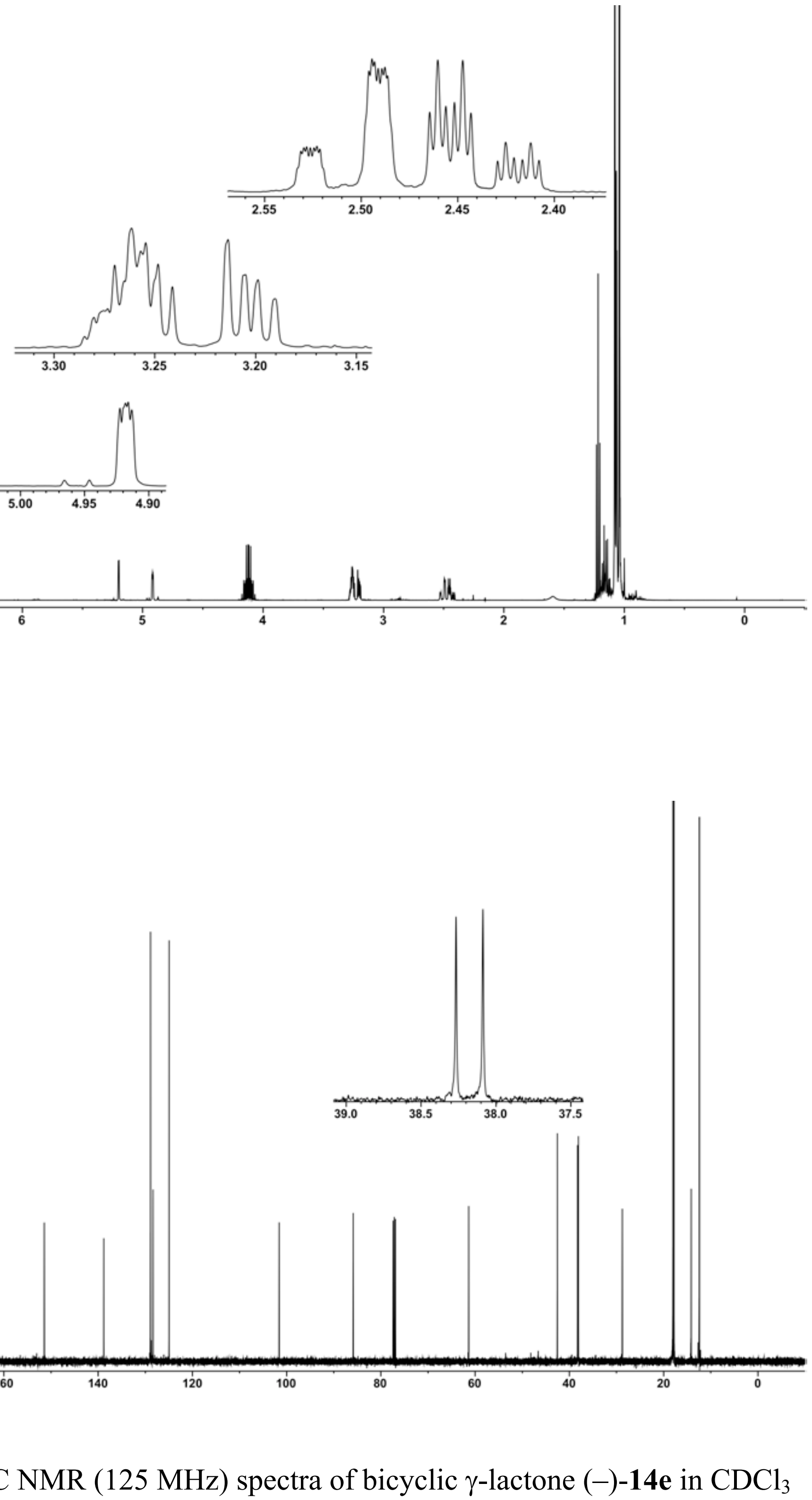

^{1}H (500 MHz) and ^{13}C NMR (125 MHz) spectra of bicyclic γ-lactone (–)-**14e** in $CDCl_3$

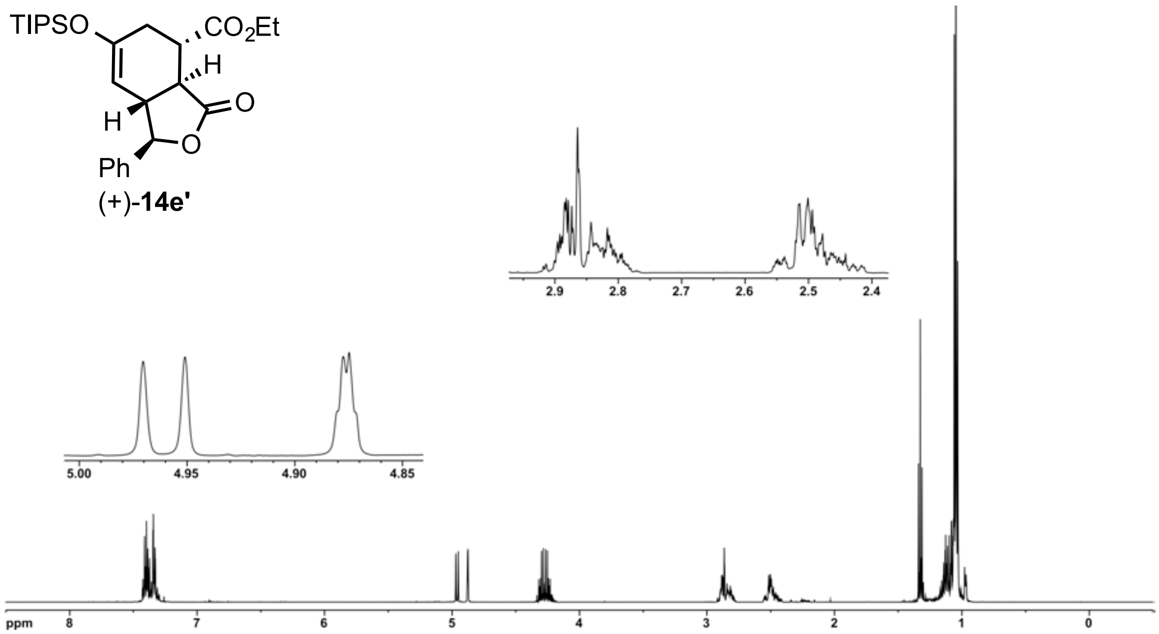

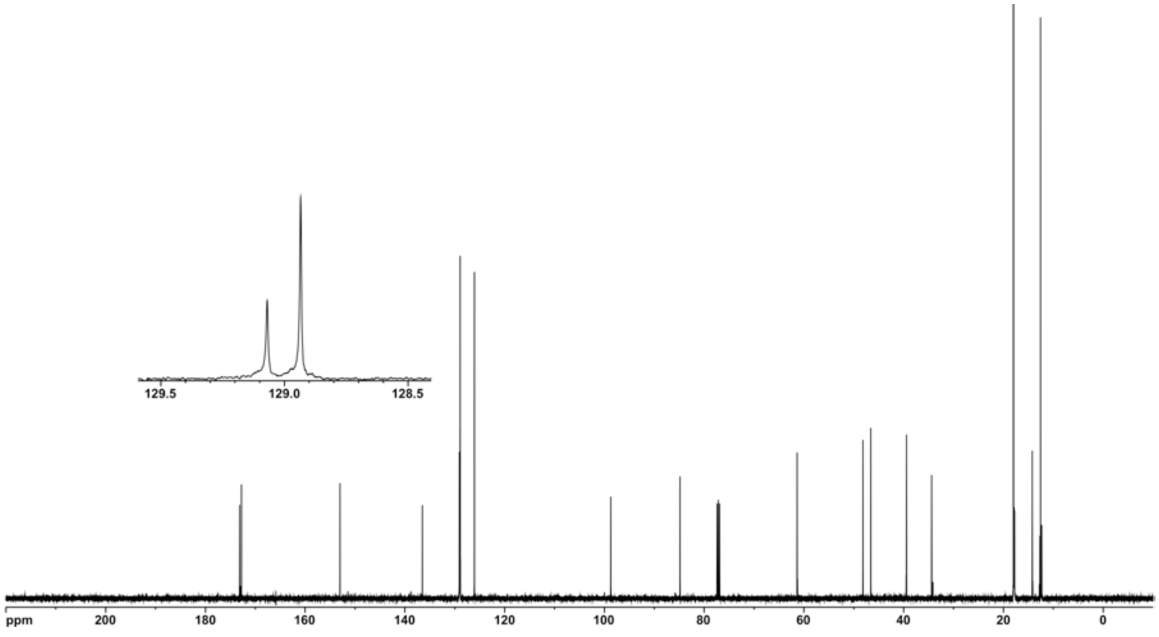

^{1}H (500 MHz) and ^{13}C NMR (125 MHz) spectra of bicyclic γ-lactone (+)-**14e'** in $CDCl_3$

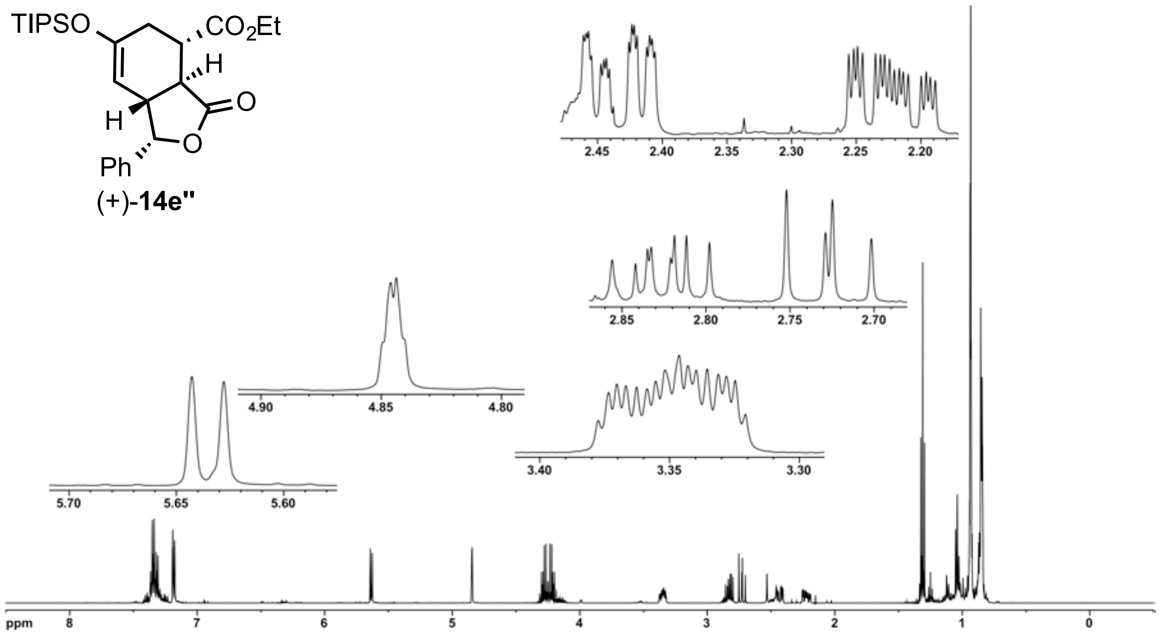

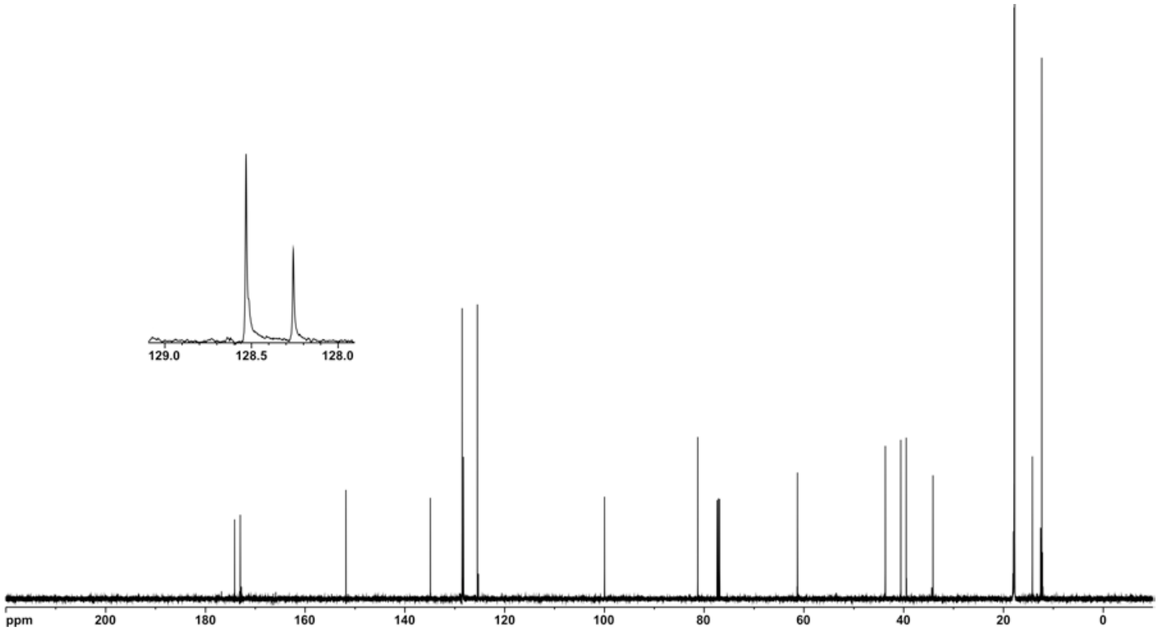

^{1}H (500 MHz) and ^{13}C NMR (125 MHz) spectra of bicyclic γ-lactone (+)-**14e''** in $CDCl_3$

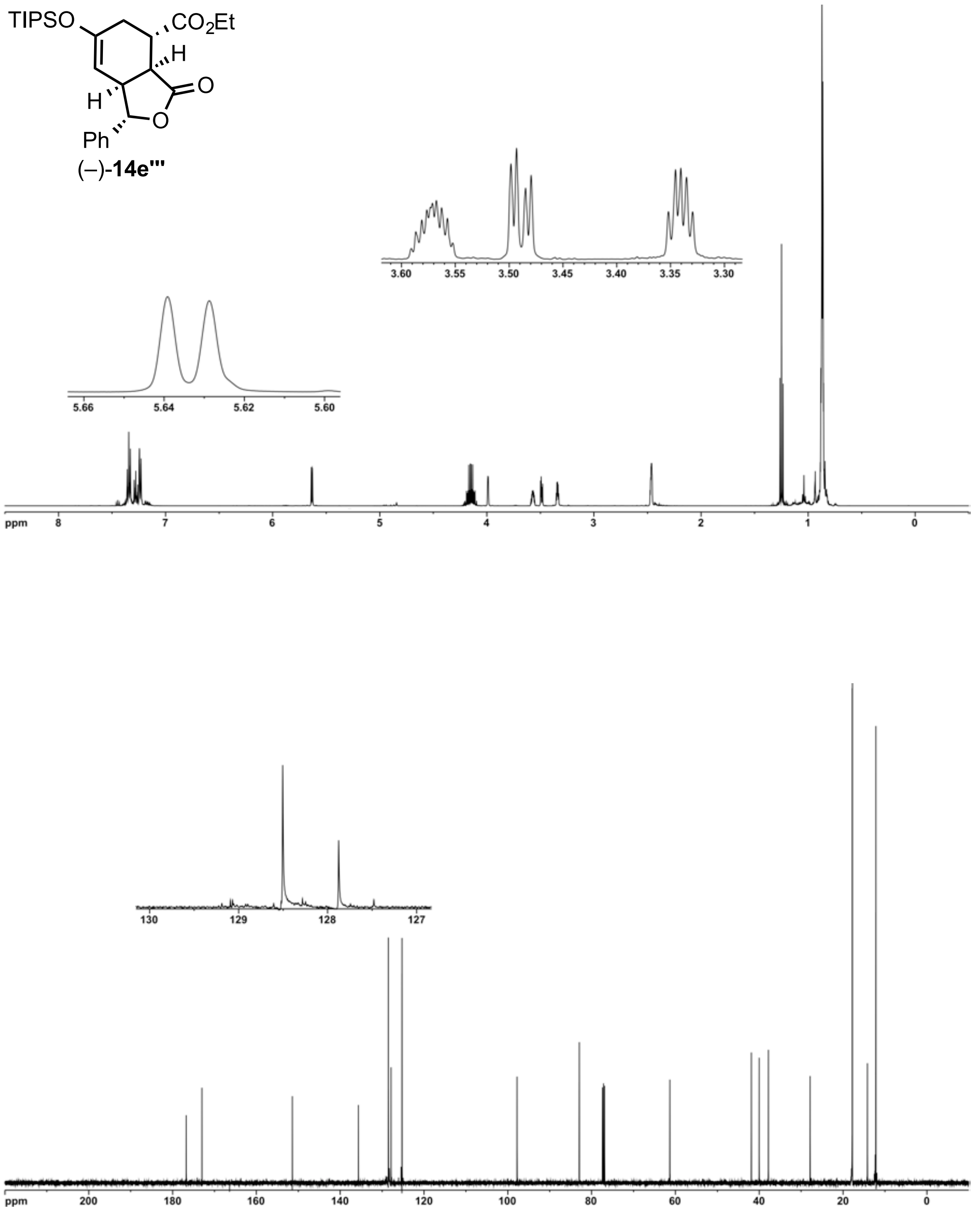

^{1}H (500 MHz) and ^{13}C NMR (125 MHz) spectra of bicyclic γ-lactone (–)-**14e’’** in $CDCl_3$

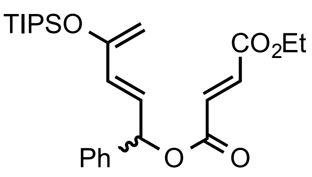

(±)-**S20**

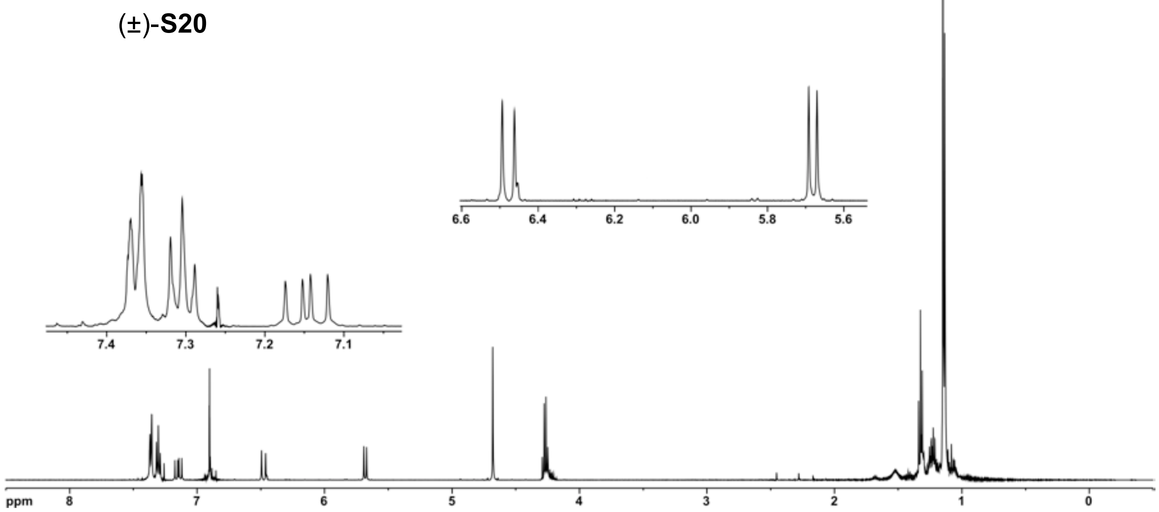

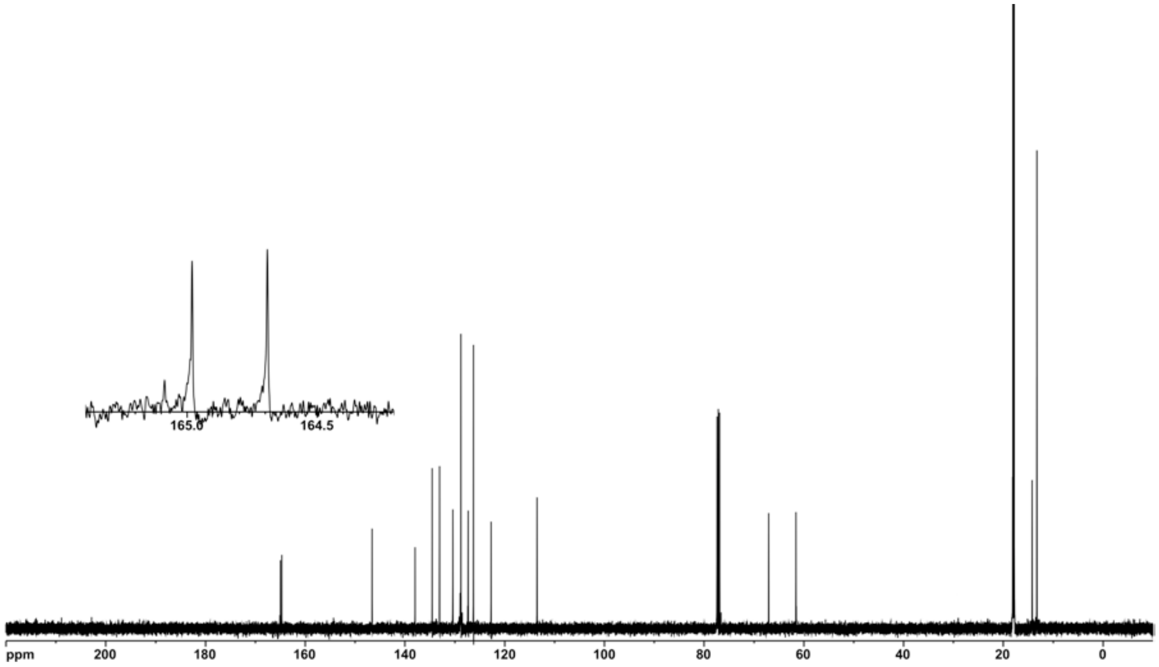

^{1}H (500 MHz) and ^{13}C NMR (125 MHz) spectra of ester (±)-**S20** in $CDCl_3$

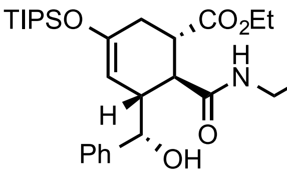

(–)-**S21**

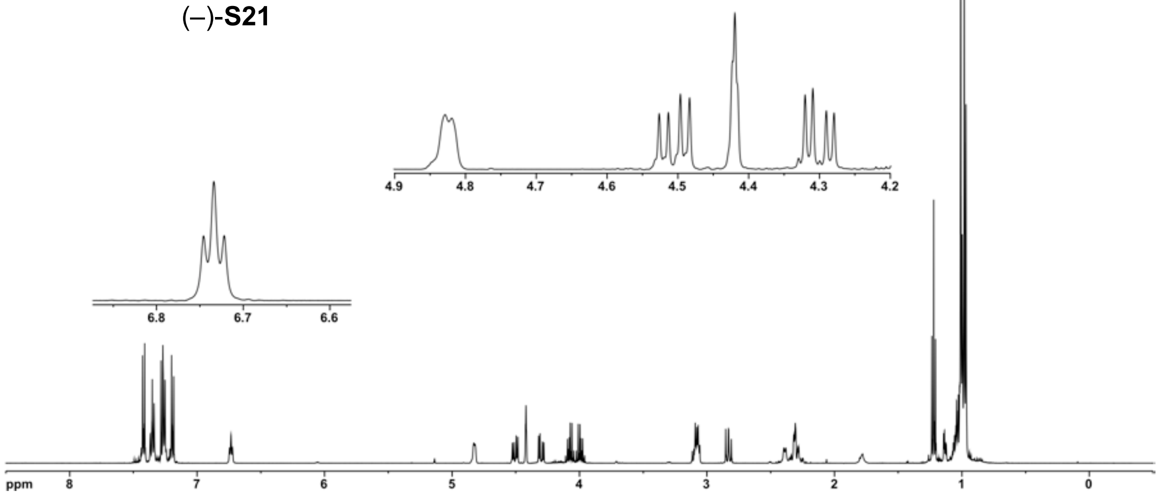

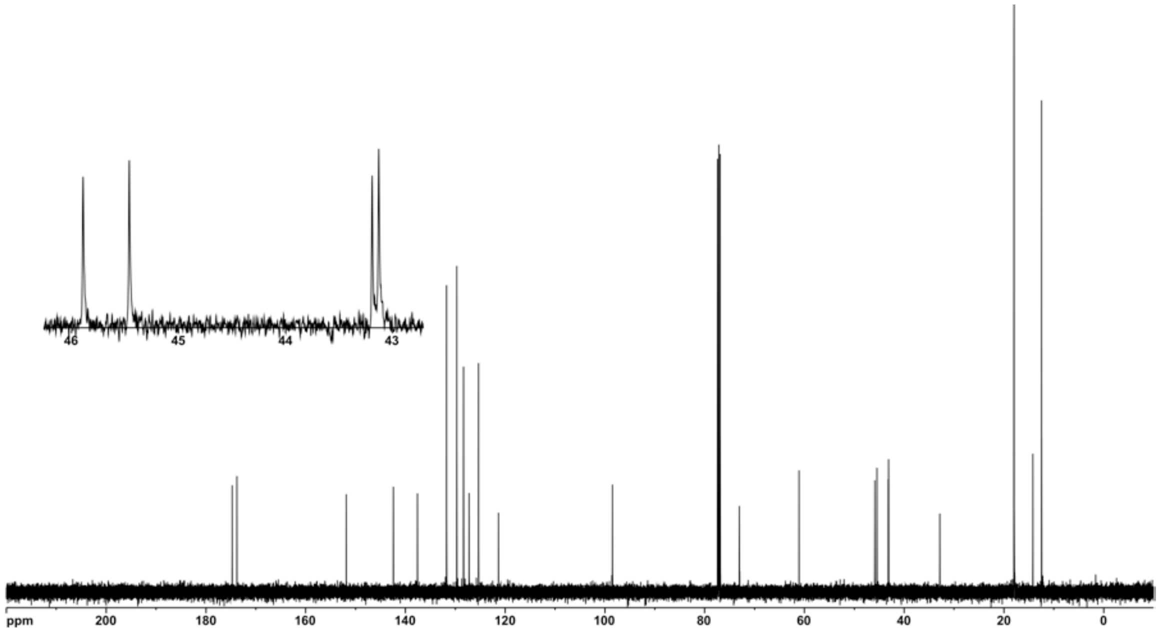

^{1}H (500 MHz) and ^{13}C NMR (125 MHz) spectra of amide (–)-**S21** in $CDCl_3$

Stereodivergent, Diels-Alder-Initiated Organocascades Employing α,β–Unsaturated Acylammonium Salts: Scope, Mechanism, and Application

Mikail E. Abbasov,[†] Brandi M. Hudson,[‡] Dean J. Tantillo[‡,*] and Daniel Romo[†,*]

[†]*Department of Chemistry and Biochemistry, Baylor University, One Bear Place 97348, Waco, Texas 76798, United States*

[‡]*Department of Chemistry, University of California–Davis, One Shields Avenue, Davis, California 95616, United States*

Table of Contents, *cont.*

• Calculated coordinates and energies of the acylammonium salt formation and the DAL processes (**Tables S1 and S2, Figures S4 and S5, Schemes S1-S4**)................. S2–S204

a. Acylammonium salt formation
b. Diels-Alder step of the DAL organocascade
   i. Acylammonium salts derived from Brønsted bases
   ii. Modeling explicit base
   iii. Entropy-controlled diastereoselection
c. Lactonization step of DAL organocascade
   i. Proton-relay deprotonation pathway
   ii. Ketene pathway
   iii. Intramolecular proton-transfer pathway

Calculated coordinates and energies of the acylammonium salt formation and the DAL processes (Tables S3 and S4, Figures S3 and S4, Schemes S1-S4):

a. **Acylammonium salt formation**

Ethyl fumaroyl chloride (**5**):

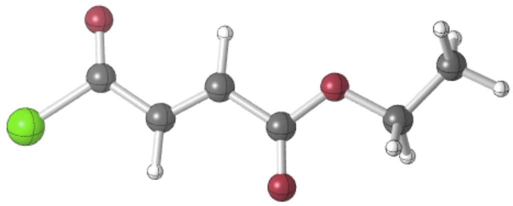

Charge = 0 Multiplicity = 1

HF = -918.5039922 hartrees (-576370.440145422 kcal/mol)

Imaginary Frequencies: none found

Zero-point correction = 0.126267 (Hartree/Particle)

Temperature 298.150 Kelvin. Pressure 1.00000 Atm.

Sum of electronic and thermal Free Energies =

-918.416338 hartrees (-576315.43625838 kcal/mol)

Coordinates (from last standard orientation):

Center Number	Atomic Number	Coordinates (Angstroms) X	Y	Z
1	6	-2.404785	0.535097	-0.020979
2	6	0.008958	0.346013	-0.031898
3	6	-1.169862	-0.277188	-0.025157
4	6	1.268965	-0.453229	-0.032192
5	6	3.616705	-0.317867	-0.008249
6	6	4.675205	0.755901	0.080148
7	1	0.088461	1.428999	-0.034584
8	1	-1.250380	-1.358672	-0.017684
9	1	3.699592	-0.904604	-0.927911
10	1	3.655164	-1.005150	0.841606
11	1	4.570953	1.326879	1.006880
12	1	5.665622	0.292605	0.068215
13	1	4.604344	1.443500	-0.767095
14	8	-2.491100	1.719758	-0.059931

15	8	2.334266	0.343047	-0.005009
16	8	1.311464	-1.661878	-0.054280
17	17	-3.896583	-0.468432	0.059665

(S)-(–)-BTM catalyst:

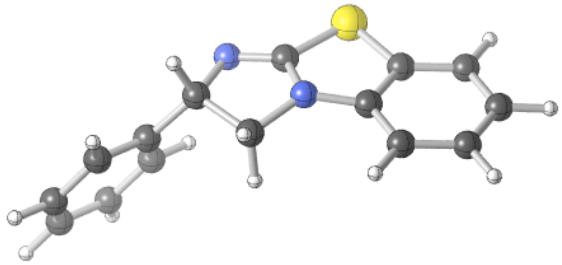

Charge = 0 Multiplicity = 1

HF = -1086.2217193 hartrees (-681614.991077943 kcal/mol)

Imaginary Frequencies: none found

Zero-point correction = 0.238948 (Hartree/Particle)

Temperature 298.150 Kelvin. Pressure 1.00000 Atm.

Sum of electronic and thermal Free Energies =

-1086.024409 hartrees (-681491.17689159 kcal/mol)

Coordinates (from last standard orientation):

Center Number	Atomic Number	Coordinates (Angstroms) X	Y	Z
1	6	-2.658926	-1.872440	0.032577
2	6	-2.123816	-0.600345	-0.141211
3	6	-2.895105	0.543447	0.132450
4	6	-4.207606	0.432725	0.561787
5	6	-4.746737	-0.845244	0.732328
6	6	-3.978352	-1.979555	0.473061
7	1	-2.058599	-2.752062	-0.178240
8	1	-4.800047	1.317910	0.772337
9	1	-5.771715	-0.949580	1.072628
10	1	-4.410212	-2.965542	0.613459
11	6	-0.539268	1.070167	-0.533267
12	16	-1.966889	2.036351	-0.147291
13	7	-0.860156	-0.271737	-0.595974
14	6	0.395774	-1.012047	-0.630557
15	6	1.379397	0.139158	-1.027978

16	7	0.675758	1.406318	-0.721608
17	6	2.713946	0.013122	-0.330895
18	6	3.702254	-0.807909	-0.878940
19	6	2.957188	0.651293	0.886801
20	6	4.914045	-0.995064	-0.218440
21	1	3.520707	-1.300931	-1.831631
22	6	4.171891	0.470832	1.545054
23	1	2.193991	1.298559	1.309570
24	6	5.151970	-0.354415	0.996466
25	1	5.674328	-1.635977	-0.655503
26	1	4.353595	0.977589	2.488602
27	1	6.098468	-0.494531	1.510257
28	1	0.621577	-1.422838	0.361589
29	1	0.374413	-1.817883	-1.366373
30	1	1.544570	0.099252	-2.112384

Pyridine:

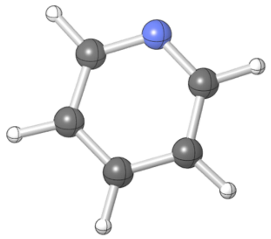

Charge = 0 Multiplicity = 1

HF = -248.1825344 hartrees (-155737.022161344 kcal/mol)

Imaginary Frequencies: none found

Zero-point correction = 0.089996 (Hartree/Particle)

Temperature 298.150 Kelvin. Pressure 1.00000 Atm.

Sum of electronic and thermal Free Energies =

-248.119925 hartrees (-155697.73413675 kcal/mol)

Coordinates (from last standard orientation):

Center Number	Atomic Number	Coordinates (Angstroms) X	Y	Z
1	6	-1.142102	-0.719597	-0.000217
2	6	-1.196328	0.671850	-0.000121
3	6	0.000902	1.381216	0.000104
4	6	1.197209	0.670340	0.000218
5	6	1.141159	-0.721043	0.000118

6	7	-0.000923	-1.415997	-0.000098
7	1	0.001567	2.467257	0.000147
8	1	-2.059831	-1.303684	-0.000375
9	1	-2.154573	1.180453	-0.000237
10	1	2.156120	1.177679	0.000362
11	1	2.058136	-1.306319	0.000177

2,6-dimethylpyridine/2,6-lutidine (DMP):

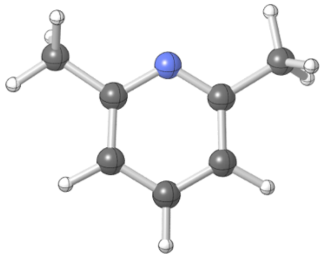

Charge = 0 Multiplicity = 1

HF = -326.7824947 hartrees (-205059.283249197 kcal/mol)

Imaginary Frequencies: none found

Zero-point correction = 0.145307 (Hartree/Particle)

Temperature 298.150 Kelvin. Pressure 1.00000 Atm.

Sum of electronic and thermal Free Energies =

-326.669833 hartrees (-204988.58690583 kcal/mol)

Coordinates (from last standard orientation):

Center Number	Atomic Number	Coordinates (Angstroms) X	Y	Z
1	6	-1.160473	-0.261946	-0.004035
2	6	-1.195166	1.131299	-0.003236
3	6	0.008859	1.831739	0.000842
4	6	1.199720	1.121255	0.003073
5	6	1.149738	-0.278042	0.001039
6	7	-0.006143	-0.949731	-0.002114
7	1	0.013558	2.918072	0.001370
8	1	-2.147267	1.652127	-0.005575
9	1	2.159615	1.628875	0.005136
10	6	2.418847	-1.088539	-0.000521
11	1	3.018921	-0.873332	-0.891088
12	1	3.035787	-0.852185	0.872816
13	1	2.182057	-2.154074	0.013626

14	6	-2.421617	-1.082545	0.002318
15	1	-2.405224	-1.812259	-0.812977
16	1	-2.505201	-1.644612	0.938663
17	1	-3.308700	-0.453813	-0.104057

2,6-di-tert-butylpyridine (DTBP):

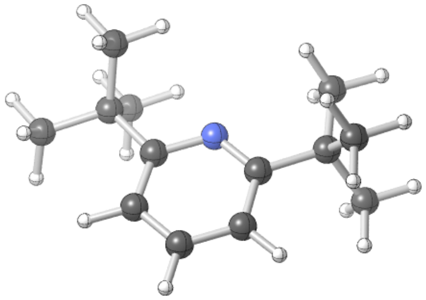

Charge = 0 Multiplicity = 1

HF = -562.5492239 hartrees (-353005.263489489 kcal/mol)

Imaginary Frequencies: none found

Zero-point correction = 0.317107 (Hartree/Particle)

Temperature 298.150 Kelvin. Pressure 1.00000 Atm.

Sum of electronic and thermal Free Energies =

-562.273067 hartrees (-352831.97227317 kcal/mol)

Coordinates (from last standard orientation):

Center Number	Atomic Number	Coordinates (Angstroms) X	Y	Z
1	6	-1.180576	0.408335	0.000012
2	6	-1.196169	1.801476	0.000030
3	6	0.020059	2.483821	0.000015
4	6	1.203313	1.764779	0.000014
5	6	1.140468	0.362841	0.000020
6	7	-0.025905	-0.281671	0.000002
7	1	0.036770	3.570270	0.000014
8	1	-2.129443	2.351939	0.000055
9	1	2.161256	2.275372	-0.000046
10	6	2.427044	-0.465323	0.000012
11	6	-2.447816	-0.446644	0.000006
12	6	3.248229	-0.121989	-1.253476
13	1	2.685170	-0.348760	-2.165585
14	1	3.526194	0.936952	-1.277977

15	1	4.172053	-0.711602	-1.267600
16	6	3.248604	-0.121667	1.253141
17	1	2.685684	-0.347765	2.165520
18	1	4.172188	-0.711639	1.267336
19	1	3.527053	0.937160	1.277196
20	6	-2.437773	-1.336562	-1.252285
21	1	-1.544821	-1.967951	-1.271301
22	1	-3.322060	-1.983973	-1.261600
23	1	-2.448767	-0.730163	-2.165274
24	6	-3.719983	0.403906	-0.000109
25	1	-4.597561	-0.251212	0.000066
26	1	-3.781104	1.042591	0.888226
27	1	-3.781200	1.042218	-0.888705
28	6	-2.437923	-1.336436	1.252372
29	1	-2.448957	-0.729938	2.165298
30	1	-3.322269	-1.983765	1.261674
31	1	-1.545030	-1.967899	1.271548
32	6	2.126182	-1.964744	0.000270
33	1	1.552170	-2.259233	-0.883510
34	1	3.069237	-2.522631	-0.000029
35	1	1.552819	-2.259038	0.884539

Triethylamine (Et_3N):

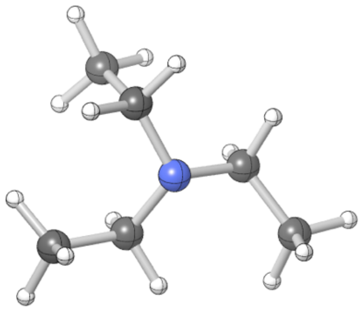

Charge = 0 Multiplicity = 1

HF = -292.2638816 hartrees (-183398.508342816 kcal/mol)

Imaginary Frequencies: none found

Zero-point correction = 0.208620 (Hartree/Particle)

Temperature 298.150 Kelvin. Pressure 1.00000 Atm.

Sum of electronic and thermal Free Energies =

-292.089062 hartrees (-183288.80729562 kcal/mol)

Coordinates (from last standard orientation):

Center Number	Atomic Number	Coordinates (Angstroms) X	Y	Z
1	7	0.183969	0.005692	-0.445264
2	6	-0.204347	1.123379	0.414541
3	1	0.638993	1.820019	0.450668
4	1	-0.380524	0.793774	1.456147
5	6	-1.425177	1.877805	-0.095980
6	1	-2.344811	1.290972	-0.007201
7	1	-1.561711	2.797111	0.481725
8	1	-1.292766	2.151361	-1.148164
9	6	1.368106	-0.677779	0.073941
10	1	1.527512	-1.567188	-0.547173
11	1	1.215926	-1.035565	1.109206
12	6	2.625140	0.185070	0.026900
13	1	2.604173	0.991360	0.765704
14	1	3.506243	-0.428581	0.239127
15	1	2.744239	0.630089	-0.966356
16	6	-0.909729	-0.934232	-0.704327
17	1	-1.682109	-0.412647	-1.279751
18	1	-0.510072	-1.714369	-1.362800
19	6	-1.551309	-1.587076	0.522755
20	1	-2.009638	-0.842789	1.182978
21	1	-2.337664	-2.280561	0.207131
22	1	-0.821674	-2.155837	1.108622

N,N-diisopropylethylamine (Hünig's base):

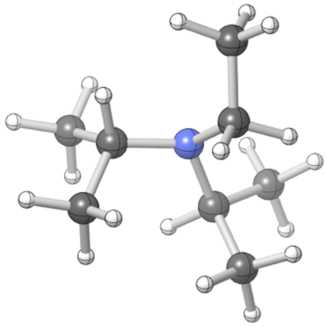

Charge = 0 Multiplicity = 1

HF = -370.8512296 hartrees (-232712.855086296 kcal/mol)

Imaginary Frequencies: none found

Zero-point correction = 0.264971 (Hartree/Particle)

Temperature 298.150 Kelvin. Pressure 1.00000 Atm.

Sum of electronic and thermal Free Energies =

-370.623000 hartrees (-232569.63873 kcal/mol)

Coordinates (from last standard orientation):

Center Number	Atomic Number	Coordinates (Angstroms) X	Y	Z
1	7	-0.006149	0.269353	-0.238357
2	6	1.059871	-0.745243	-0.178059
3	6	-1.375137	-0.238523	-0.070324
4	1	-2.012282	0.651431	-0.014384
5	6	0.270351	1.477969	0.532653
6	1	1.353621	1.635972	0.530137
7	1	-0.023669	1.376472	1.593245
8	6	-1.623659	-1.055763	1.204151
9	1	-1.314056	-0.509602	2.101703
10	1	-2.690827	-1.282688	1.303522
11	1	-1.083257	-2.008995	1.175890
12	6	-0.381945	2.720065	-0.066839
13	1	-1.474801	2.664507	-0.058023
14	1	-0.057128	2.849836	-1.104117
15	6	2.145273	-0.410267	-1.201556
16	1	1.716725	-0.343747	-2.206163
17	1	2.617300	0.552285	-0.970067
18	1	2.931039	-1.173875	-1.204178
19	1	0.601667	-1.691992	-0.485529
20	1	-0.094660	3.610729	0.501865
21	6	-1.819231	-1.020263	-1.307062
22	1	-2.885234	-1.263479	-1.242411
23	1	-1.650251	-0.428922	-2.212203
24	1	-1.274600	-1.965634	-1.408852
25	6	1.690193	-0.970464	1.204786
26	1	0.939207	-1.160258	1.976242
27	1	2.361975	-1.835005	1.167265
28	1	2.287975	-0.107567	1.518051

*Acylammonium formation between ethyl fumaroyl chloride (**5**) and (S)-(–)-BTM:*

Reactant:

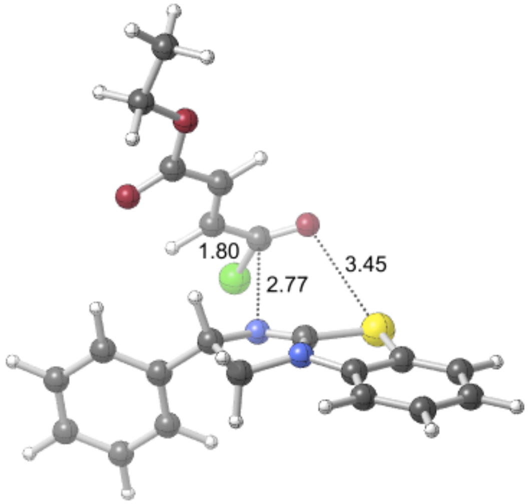

Charge = 0 Multiplicity = 1

HF = -2004.7354502 hartrees (-1257991.542355 kcal/mol)

Imaginary Frequencies: none found

Zero-point correction = 0.366518 (Hartree/Particle)

Temperature 298.150 Kelvin. Pressure 1.00000 Atm.

Sum of electronic and thermal Free Energies =

-2004.430122 hartrees (-1257799.94585622 kcal/mol)

Coordinates (from last standard orientation):

Center Number	Atomic Number	Coordinates (Angstroms) X	Y	Z
1	6	4.016255	-0.750918	-1.998485
2	6	3.259790	-0.501244	-0.858454
3	6	3.801530	-0.715302	0.421748
4	6	5.094135	-1.189442	0.578939
5	6	5.853737	-1.441601	-0.565986
6	6	5.319468	-1.220478	-1.835275
7	1	3.592958	-0.583080	-2.983890
8	1	5.509950	-1.351574	1.568622
9	1	6.869138	-1.809285	-0.461213
10	1	5.923341	-1.419464	-2.715145
11	6	1.494327	0.242923	0.472386
12	16	2.636113	-0.299426	1.701476

13	7	1.949409	-0.063504	-0.793986
14	6	1.100575	0.659484	-1.736775
15	1	1.594154	1.586983	-2.055080
16	1	0.851405	0.055083	-2.609959
17	6	-0.133684	0.930631	-0.824512
18	7	0.375597	0.850508	0.569851
19	6	-0.843907	2.231573	-1.104753
20	6	-2.129435	2.224942	-1.647200
21	6	-0.225405	3.453587	-0.824667
22	6	-2.785762	3.424734	-1.922230
23	1	-2.620123	1.273276	-1.841152
24	6	-0.880019	4.651035	-1.095775
25	1	0.768328	3.462197	-0.382736
26	6	-2.160780	4.638783	-1.649903
27	1	-3.786666	3.408481	-2.343711
28	1	-0.392567	5.595838	-0.873115
29	1	-2.671501	5.573898	-1.860521
30	1	-0.851966	0.110325	-0.961783
31	6	-1.188677	-0.049738	2.672937
32	6	-1.999878	-0.363416	1.475493
33	6	-2.110974	-1.629256	1.073431
34	6	-2.890706	-1.942999	-0.154787
35	6	-3.757958	-3.680892	-1.482621
36	6	-3.751378	-5.191427	-1.494734
37	1	-2.480699	0.454999	0.948125
38	1	-4.771155	-3.274411	-1.415224
39	1	-3.278592	-3.258979	-2.370683
40	1	-4.221830	-5.589014	-0.591224
41	1	-4.309962	-5.550733	-2.363489
42	1	-2.729380	-5.575181	-1.557370
43	1	-1.641989	-2.449145	1.608776
44	8	-0.424087	-0.766861	3.233512
45	8	-3.343798	-1.113150	-0.914108
46	8	-3.018747	-3.257622	-0.318854
47	17	-1.529421	1.590397	3.329944

Transition state (TS):

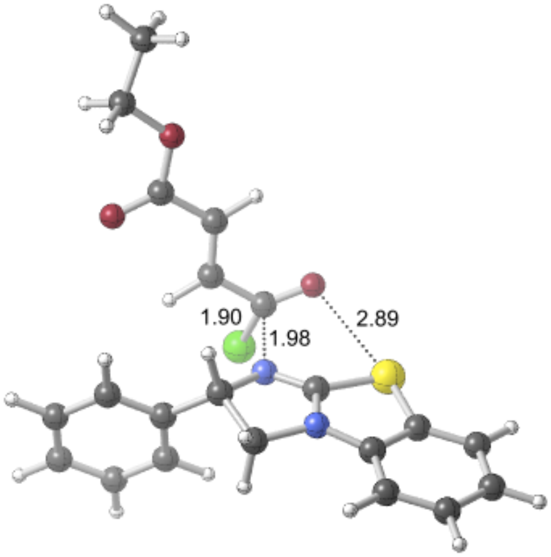

Charge = 0 Multiplicity = 1

HF = -2004.7265389 hartrees (-1257985.95042514 kcal/mol)

Imaginary Frequencies: 1 (-211.9153 1/cm)

Zero-point correction = 0.366867 (Hartree/Particle)

Temperature 298.150 Kelvin. Pressure 1.00000 Atm.

Sum of electronic and thermal Free Energies =

-2004.418503 hartrees (-1257792.65481753 kcal/mol)

Coordinates (from last standard orientation):

Center Number	Atomic Number	Coordinates (Angstroms) X	Y	Z
1	6	4.559707	-0.410702	-1.948327
2	6	3.618700	-0.601882	-0.942296
3	6	3.902199	-1.410750	0.169062
4	6	5.123726	-2.057076	0.284853
5	6	6.070459	-1.868125	-0.723074
6	6	5.789556	-1.053841	-1.821812
7	1	4.331335	0.217803	-2.803138
8	1	5.341674	-2.686043	1.142434
9	1	7.034590	-2.359678	-0.645840
10	1	6.539308	-0.917268	-2.594496
11	6	1.654749	-0.365836	0.272716
12	16	2.531186	-1.480526	1.301619

13	7	2.333643	-0.089462	-0.873726
14	6	1.639340	0.984536	-1.581092
15	1	2.165679	1.933794	-1.428453
16	1	1.556460	0.774002	-2.648254
17	6	0.257817	0.959748	-0.857679
18	7	0.525020	0.239848	0.415058
19	6	-0.343474	2.329822	-0.655021
20	6	-1.494154	2.692866	-1.355070
21	6	0.251334	3.250751	0.212246
22	6	-2.043346	3.965227	-1.199794
23	1	-1.965725	1.972168	-2.019250
24	6	-0.298363	4.518923	0.371728
25	1	1.138909	2.966413	0.772248
26	6	-1.445547	4.879253	-0.336773
27	1	-2.939561	4.236456	-1.749487
28	1	0.164266	5.226670	1.053191
29	1	-1.874419	5.868841	-0.209985
30	1	-0.443799	0.349687	-1.441078
31	6	-0.710020	-0.516679	1.767721
32	6	-1.936313	-0.331476	0.942140
33	6	-2.663757	-1.386669	0.583184
34	6	-3.873806	-1.189375	-0.256890
35	6	-5.699015	-2.271687	-1.273391
36	6	-6.284058	-3.663371	-1.337220
37	1	-2.203876	0.678990	0.646580
38	1	-6.390171	-1.556195	-0.818810
39	1	-5.422517	-1.895965	-2.263245
40	1	-6.548943	-4.017542	-0.337043
41	1	-7.189109	-3.653520	-1.950859
42	1	-5.571665	-4.364736	-1.780516
43	1	-2.402758	-2.395905	0.885811
44	8	-0.233949	-1.560660	2.123255
45	8	-4.240505	-0.124946	-0.705384
46	8	-4.510820	-2.344314	-0.462479
47	17	-0.611854	0.927949	3.002384

Product:

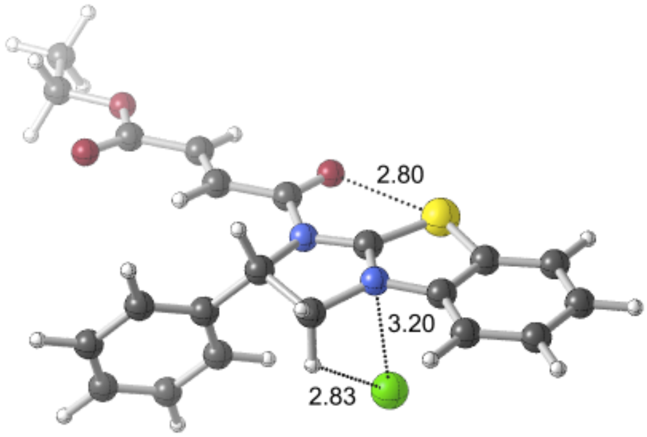

Charge = 0 Multiplicity = 1

HF = -2004.7674034 hartrees (-1258011.59330753 kcal/mol)

Imaginary Frequencies: none found

Zero-point correction = 0.369358 (Hartree/Particle)

Temperature 298.150 Kelvin. Pressure 1.00000 Atm.

Sum of electronic and thermal Free Energies =

-2004.458009 hartrees (-1257817.44522759 kcal/mol)

Coordinates (from last standard orientation):
atom 47 is isolated, type=Cl

Center Number	Atomic Number	Coordinates (Angstroms) X	Y	Z
1	6	4.968076	0.506345	-0.972001
2	6	3.837681	-0.226767	-0.625492
3	6	3.929947	-1.507647	-0.067999
4	6	5.170030	-2.099315	0.149735
5	6	6.303535	-1.371506	-0.194842
6	6	6.203022	-0.086036	-0.744257
7	1	4.875312	1.501290	-1.394426
8	1	5.250813	-3.090896	0.582818
9	1	7.283033	-1.808010	-0.030072
10	1	7.106452	0.459360	-0.996033
11	6	1.641523	-0.729392	-0.307411
12	16	2.333027	-2.175298	0.290600
13	7	2.508464	0.145384	-0.777523
14	6	1.864146	1.430251	-1.055253

15	1	2.082110	2.094605	-0.213310
16	1	2.212824	1.849128	-1.998701
17	6	0.362350	1.038406	-1.106831
18	7	0.366245	-0.293207	-0.412090
19	6	-0.538433	2.065267	-0.464819
20	6	-1.368486	2.842715	-1.273629
21	6	-0.530744	2.252798	0.919594
22	6	-2.189727	3.812088	-0.701695
23	1	-1.376697	2.683320	-2.349387
24	6	-1.359495	3.217186	1.485875
25	1	0.137024	1.656364	1.541044
26	6	-2.188186	3.997034	0.678874
27	1	-2.834486	4.415097	-1.333616
28	1	-1.355647	3.361672	2.562122
29	1	-2.833837	4.746812	1.126494
30	1	0.057585	0.862650	-2.143065
31	6	-0.699828	-1.134672	-0.085313
32	6	-2.061485	-0.655405	-0.414817
33	6	-3.116138	-1.363041	-0.010429
34	6	-4.488954	-0.896531	-0.355779
35	6	-6.782628	-1.349797	-0.118533
36	6	-7.659461	-2.400665	0.520158
37	1	-2.197845	0.255799	-0.987315
38	1	-6.961361	-0.355038	0.299858
39	1	-6.925663	-1.296518	-1.201660
40	1	-7.490721	-2.442760	1.599642
41	1	-8.710163	-2.155149	0.342349
42	1	-7.457755	-3.386877	0.092996
43	1	-3.010621	-2.273056	0.572359
44	8	-0.463024	-2.213467	0.424781
45	8	-4.732399	0.094861	-1.006469
46	8	-5.412461	-1.715035	0.144315
47	17	2.457697	0.904880	2.327868

*Acylammonium formation between ethyl fumaroyl chloride (**5**) and pyridine:*

Reactant:

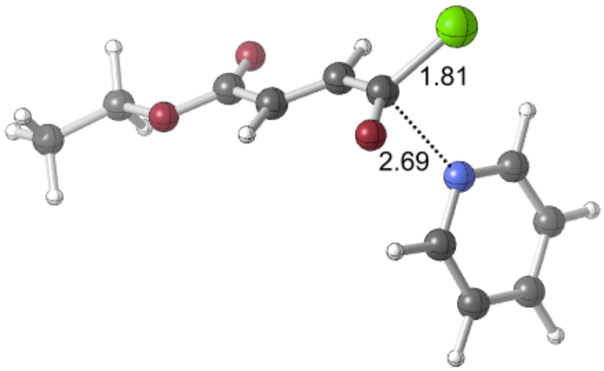

Charge = 0 Multiplicity = 1

HF = -1166.6946578 hartrees (-732112.564716078 kcal/mol)

Imaginary Frequencies: none found

Zero-point correction = 0.217776 (Hartree/Particle)

Temperature 298.150 Kelvin. Pressure 1.00000 Atm.

Sum of electronic and thermal Free Energies =

-1166.525651 hartrees (-732006.51125901 kcal/mol)

Coordinates (from last standard orientation):

Center Number	Atomic Number	Coordinates (Angstroms) X	Y	Z
1	6	0.708752	1.798313	0.607036
2	6	-0.332224	1.152919	-0.223175
3	6	-1.431804	0.680994	0.363521
4	6	-2.501686	0.064084	-0.468005
5	6	-4.633268	-0.926498	-0.418989
6	6	-5.682644	-1.281518	0.607738
7	1	-0.183860	1.107588	-1.296433
8	1	-5.004888	-0.207767	-1.155227
9	1	-4.267343	-1.806950	-0.955388
10	1	-6.025506	-0.387819	1.136557
11	1	-6.541068	-1.739906	0.108936
12	1	-5.286783	-1.992575	1.338164
13	1	-1.584683	0.740382	1.436813
14	8	0.835484	1.735716	1.786843
15	8	-2.451887	-0.060933	-1.671024
16	8	-3.527671	-0.328082	0.286741

17	17	1.771965	2.894914	-0.355258
18	6	2.129871	-1.168586	1.009448
19	6	2.891876	-2.329386	1.107584
20	6	3.705069	-2.679376	0.033490
21	6	3.724359	-1.856699	-1.089084
22	6	2.925895	-0.716332	-1.089041
23	7	2.142355	-0.374101	-0.063315
24	1	4.314301	-3.577467	0.070430
25	1	1.482110	-0.855652	1.827621
26	1	2.847366	-2.938516	2.004121
27	1	4.342327	-2.090051	-1.949606
28	1	2.915559	-0.047207	-1.947006

Transition state (TS):

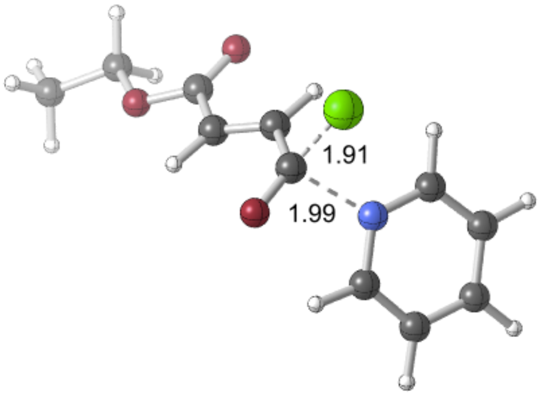

Charge = 0 Multiplicity = 1

HF = -1166.6889739 hartrees (-732108.998011989 kcal/mol)

Imaginary Frequencies: 1 (-149.0531 1/cm)

Zero-point correction = 0.217993 (Hartree/Particle)

Temperature 298.150 Kelvin. Pressure 1.00000 Atm.

Sum of electronic and thermal Free Energies =

-1166.516493 hartrees (-732000.76452243 kcal/mol)

Coordinates (from last standard orientation):

Center Number	Atomic Number	Coordinates (Angstroms) X	Y	Z
1	6	0.868671	1.253407	0.588388
2	6	-0.270005	0.747059	-0.229682
3	6	-1.436745	0.494813	0.359021

4	6	-2.580762	-0.003900	-0.447987
5	6	-4.812764	-0.740860	-0.352224
6	6	-5.858413	-1.017510	0.702580
7	1	-0.120469	0.604874	-1.295713
8	1	-5.145965	0.012254	-1.072849
9	1	-4.539510	-1.644043	-0.905392
10	1	-6.110005	-0.104545	1.249452
11	1	-6.766711	-1.397882	0.227084
12	1	-5.500581	-1.766052	1.415138
13	1	-1.583899	0.642897	1.424257
14	8	0.897262	1.358637	1.781921
15	8	-2.561273	-0.177207	-1.646635
16	8	-3.642920	-0.243461	0.324399
17	17	1.750922	2.610327	-0.430519
18	6	2.543287	-0.878999	1.193980
19	6	3.427043	-1.946641	1.089171
20	6	3.893189	-2.300219	-0.173132
21	6	3.466917	-1.576873	-1.285148
22	6	2.584338	-0.522664	-1.094064
23	7	2.144317	-0.198455	0.123743
24	1	4.585255	-3.128169	-0.290668
25	1	2.140269	-0.537273	2.144764
26	1	3.737424	-2.483471	1.978483
27	1	3.810039	-1.819684	-2.284505
28	1	2.225170	0.085292	-1.920321

Product:

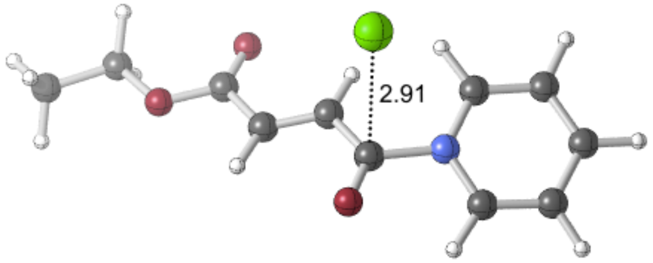

Charge = 0 Multiplicity = 1

HF = -1166.7014384 hartrees (-732116.819610384 kcal/mol)

Imaginary Frequencies: none found

Zero-point correction = 0.220157 (Hartree/Particle)

Temperature 298.150 Kelvin. Pressure 1.00000 Atm.

Sum of electronic and thermal Free Energies =

-1166.527748 hartrees (-732007.82714748 kcal/mol)

Coordinates (from last standard orientation):
atom 17 is isolated, type=Cl

Center Number	Atomic Number	Coordinates (Angstroms) X	Y	Z
1	6	0.881273	-0.311717	0.840776
2	6	-0.281936	-0.195798	-0.061075
3	6	-1.505205	-0.363075	0.442767
4	6	-2.679946	-0.287966	-0.467747
5	6	-5.027766	-0.253099	-0.570380
6	6	-6.193772	-0.290489	0.388786
7	1	-0.148957	-0.032387	-1.123378
8	1	-4.997602	0.677937	-1.143558
9	1	-5.048234	-1.088629	-1.276441
10	1	-6.143378	0.544841	1.092603
11	1	-7.129691	-0.215345	-0.171932
12	1	-6.203097	-1.226403	0.954257
13	1	-1.678674	-0.530282	1.501159
14	8	0.842400	-0.513396	2.018776
15	8	-2.608184	-0.185262	-1.672405
16	8	-3.822250	-0.345885	0.214949
17	17	1.105480	2.560580	0.460337
18	6	3.171763	-1.105114	0.852925
19	6	4.433859	-1.257757	0.319455

20	6	4.716801	-0.691544	-0.921627
21	6	3.727203	0.021400	-1.593040
22	6	2.479994	0.153854	-1.017351
23	7	2.224553	-0.417609	0.179388
24	1	5.703258	-0.801138	-1.359804
25	1	2.869604	-1.510242	1.810131
26	1	5.178294	-1.815788	0.873894
27	1	3.913731	0.490628	-2.551191
28	1	1.690366	0.744388	-1.458701

*Acylammonium formation between ethyl fumaroyl chloride (**5**) and 2,6-lutidine:*

Reactant:

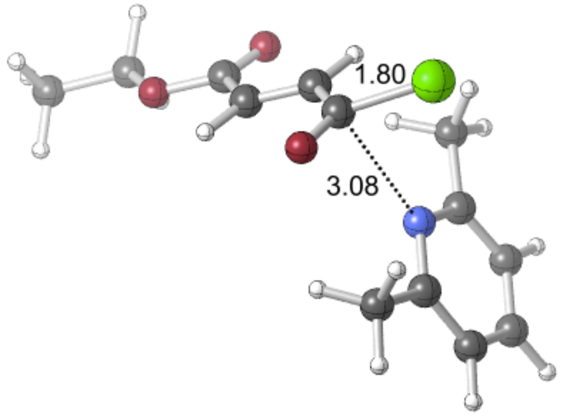

Charge = 0 Multiplicity = 1

HF = -1245.2952046 hartrees (-781435.193838546 kcal/mol)

Imaginary Frequencies: none found

Zero-point correction = 0.273816 (Hartree/Particle)

Temperature 298.150 Kelvin. Pressure 1.00000 Atm.

Sum of electronic and thermal Free Energies =

-1245.073343 hartrees (-781295.97346593 kcal/mol)

Coordinates (from last standard orientation):

Center Number	Atomic Number	Coordinates (Angstroms) X	Y	Z
1	6	-0.345275	-2.206787	0.063153
2	6	0.666383	-1.296443	-0.513907
3	6	1.704399	-0.918594	0.231724
4	6	2.712568	0.015192	-0.341475

5	6	4.752913	1.114687	0.059510
6	6	5.807895	1.157591	1.139706
7	1	0.540835	-0.959209	-1.535928
8	1	5.148527	0.739717	-0.888495
9	1	4.306983	2.097459	-0.119486
10	1	6.233454	0.164190	1.306290
11	1	6.613268	1.832108	0.836145
12	1	5.386931	1.522822	2.080438
13	1	1.837569	-1.260181	1.253713
14	8	-0.360126	-2.659582	1.162395
15	8	2.628136	0.525835	-1.436990
16	8	3.715505	0.219098	0.509150
17	17	-1.622926	-2.672984	-1.111808
18	6	-2.604627	0.572900	1.174039
19	6	-3.583690	1.560013	1.310622
20	6	-3.723092	2.509662	0.306132
21	6	-2.879370	2.449328	-0.796667
22	6	-1.923905	1.432126	-0.860015
23	7	-1.794168	0.513055	0.108013
24	1	-4.476142	3.288858	0.383983
25	1	-4.218222	1.574493	2.190895
26	1	-2.952887	3.174829	-1.600684
27	6	-0.980763	1.324170	-2.027637
28	1	-1.126348	0.371205	-2.548904
29	1	-1.140704	2.134227	-2.743251
30	1	0.060544	1.358650	-1.688700
31	6	-2.412972	-0.480748	2.230369
32	1	-2.687682	-1.468378	1.844388
33	1	-1.362758	-0.530852	2.533359
34	1	-3.025351	-0.271975	3.111117

Transition state (TS):

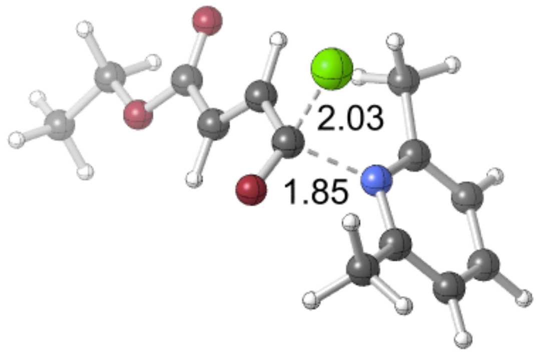

Charge = 0 Multiplicity = 1

HF = -1245.2722364 hartrees (-781420.781063364 kcal/mol)

Imaginary Frequencies: 1 (-238.9462 1/cm)

Zero-point correction = 0.274824 (Hartree/Particle)

Temperature 298.150 Kelvin. Pressure 1.00000 Atm.

Sum of electronic and thermal Free Energies =

-1245.045606 hartrees (-781278.56822106 kcal/mol)

Coordinates (from last standard orientation):

Center Number	Atomic Number	Coordinates (Angstroms) X	Y	Z
1	6	-0.777521	-1.223493	0.334995
2	6	0.533371	-0.946159	-0.340662
3	6	1.536939	-0.455485	0.383642
4	6	2.846067	-0.199922	-0.271674
5	6	5.066440	0.491757	0.081244
6	6	5.923387	0.974001	1.228107
7	1	0.667608	-1.195354	-1.385202
8	1	5.462406	-0.422713	-0.370203
9	1	4.970007	1.245853	-0.705286
10	1	5.996403	0.209483	2.006559
11	1	6.930836	1.194028	0.863930
12	1	5.507478	1.884930	1.667648
13	1	1.434395	-0.228240	1.439938
14	8	-0.842846	-1.504922	1.498771
15	8	3.063500	-0.335925	-1.456286
16	8	3.756999	0.211273	0.612216
17	17	-1.754503	-2.489180	-0.915799

18	6	-2.839645	0.430625	0.924420
19	6	-3.495100	1.659148	0.991445
20	6	-3.180496	2.669160	0.097932
21	6	-2.264940	2.398119	-0.906877
22	6	-1.628048	1.160501	-0.954002
23	7	-1.868453	0.231866	0.001568
24	1	-3.674229	3.634179	0.154401
25	1	-4.263497	1.794589	1.744730
26	1	-2.043619	3.126951	-1.678750
27	6	-0.740792	0.860278	-2.131918
28	1	-0.883764	-0.166013	-2.478477
29	1	-1.014117	1.535039	-2.946226
30	1	0.318747	1.016882	-1.912758
31	6	-3.311680	-0.667138	1.844107
32	1	-3.267623	-1.643914	1.362594
33	1	-2.717385	-0.718500	2.758280
34	1	-4.347028	-0.445958	2.114277

Product:

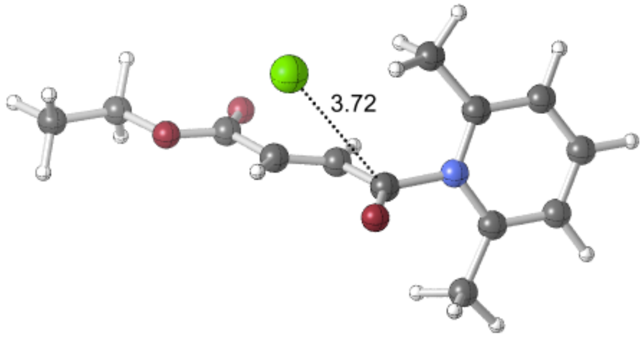

Charge = 0 Multiplicity = 1

HF = -1245.2926707 hartrees (-781433.603790957 kcal/mol)

Imaginary Frequencies: none found

Zero-point correction = 0.275979 (Hartree/Particle)

Temperature 298.150 Kelvin. Pressure 1.00000 Atm.

Sum of electronic and thermal Free Energies =

-1245.066450 hartrees (-781291.6480395 kcal/mol)

Coordinates (from last standard orientation):
atom 17 is isolated, type=Cl

Center Number	Atomic Number	Coordinates (Angstroms) X	Y	Z
1	6	-0.923356	0.162442	0.786778
2	6	0.187117	0.626700	-0.050039
3	6	1.436646	0.358831	0.337653
4	6	2.577644	0.794854	-0.520605
5	6	4.919139	0.900937	-0.686591
6	6	6.118921	0.562847	0.166143
7	1	-0.032984	1.138086	-0.982637
8	1	4.916696	0.349250	-1.631145
9	1	4.862752	1.969723	-0.913073
10	1	6.142387	-0.505829	0.395614
11	1	7.033391	0.822784	-0.374415
12	1	6.098738	1.124162	1.104308
13	1	1.645654	-0.203753	1.241127
14	8	-0.892262	-0.202541	1.922640
15	8	2.451661	1.329446	-1.599775
16	8	3.742716	0.527663	0.059706
17	17	1.185423	-2.896642	0.855859
18	6	-3.184315	1.083279	0.554885
19	6	-4.433927	1.089747	-0.039855
20	6	-4.722551	0.187815	-1.058737
21	6	-3.754044	-0.711727	-1.482316
22	6	-2.497021	-0.708190	-0.894761
23	7	-2.261477	0.189147	0.101319
24	1	-5.704761	0.186451	-1.520398
25	1	-5.171196	1.802936	0.309448
26	1	-3.953682	-1.429741	-2.268976
27	6	-1.407947	-1.642372	-1.315821
28	1	-0.822959	-2.037602	-0.473700
29	1	-1.853731	-2.478567	-1.857724
30	1	-0.713071	-1.133229	-1.994088
31	6	-2.816268	2.014784	1.667801
32	1	-2.744017	1.471674	2.614346
33	1	-1.855470	2.505618	1.484890
34	1	-3.586757	2.780694	1.759815

*Acylammonium formation between ethyl fumaroyl chloride (**5**) and DTBP:*

Reactant:

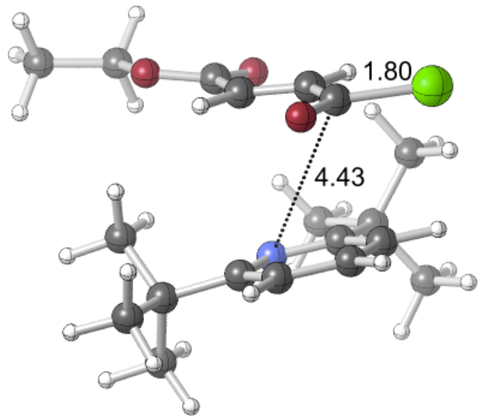

Charge = 0 Multiplicity = 1

HF = -1481.0665023 hartrees (-929384.040858273 kcal/mol)

Imaginary Frequencies: none found

Zero-point correction = 0.445172 (Hartree/Particle)

Temperature 298.150 Kelvin. Pressure 1.00000 Atm.

Sum of electronic and thermal Free Energies =

-1480.678568 hartrees (-929140.60820568 kcal/mol)

Coordinates (from last standard orientation):

Center Number	Atomic Number	Coordinates (Angstroms) X	Y	Z
1	6	2.062780	2.142789	0.994814
2	6	1.137393	1.902124	-0.130945
3	6	-0.177804	1.928220	0.092299
4	6	-1.115599	1.705143	-1.045816
5	6	-3.366897	1.790545	-1.719134
6	6	-4.704846	2.126845	-1.104774
7	1	1.553065	1.719108	-1.115814
8	1	-3.139803	2.419184	-2.585131
9	1	-3.308767	0.743369	-2.031342
10	1	-4.730150	3.169415	-0.775793
11	1	-5.493910	1.978284	-1.847136
12	1	-4.910962	1.480875	-0.246285
13	1	-0.594150	2.134983	1.074346
14	8	1.789131	2.346691	2.132793
15	8	-0.779984	1.310951	-2.140674

16	8	-2.362436	2.021420	-0.710485
17	17	3.792407	2.121224	0.480116
18	6	-0.364692	-1.269019	1.362433
19	6	0.720365	-0.923390	2.166622
20	6	1.991230	-0.887691	1.593979
21	6	2.139773	-1.182810	0.248688
22	6	0.998374	-1.511309	-0.502770
23	7	-0.212220	-1.541974	0.052404
24	1	2.856289	-0.628451	2.199412
25	1	0.591496	-0.696547	3.218349
26	1	3.122421	-1.174049	-0.212857
27	6	1.135571	-1.902879	-1.975282
28	6	-1.785524	-1.442105	1.901943
29	6	-0.231564	-2.152868	-2.613703
30	1	-0.858786	-1.257671	-2.560617
31	1	-0.095853	-2.418413	-3.668307
32	1	-0.762876	-2.971190	-2.118504
33	6	1.970438	-3.192839	-2.054933
34	1	2.064408	-3.513328	-3.098888
35	1	2.980139	-3.046618	-1.656143
36	1	1.495198	-4.003610	-1.491267
37	6	-2.180503	-2.916413	1.718771
38	1	-1.499933	-3.575810	2.269545
39	1	-3.196326	-3.082378	2.095307
40	1	-2.150679	-3.197764	0.661914
41	6	-1.889678	-1.078894	3.384281
42	1	-1.597353	-0.038472	3.567298
43	1	-2.926430	-1.197567	3.717102
44	1	-1.264388	-1.727375	4.007741
45	6	-2.751754	-0.557817	1.103333
46	1	-2.563219	0.503136	1.295768
47	1	-2.647747	-0.738135	0.028130
48	1	-3.785940	-0.777096	1.394181
49	6	1.850169	-0.788493	-2.755589
50	1	2.828834	-0.548310	-2.325333
51	1	2.013782	-1.109277	-3.791227
52	1	1.239183	0.119392	-2.771634

Transition state (TS):

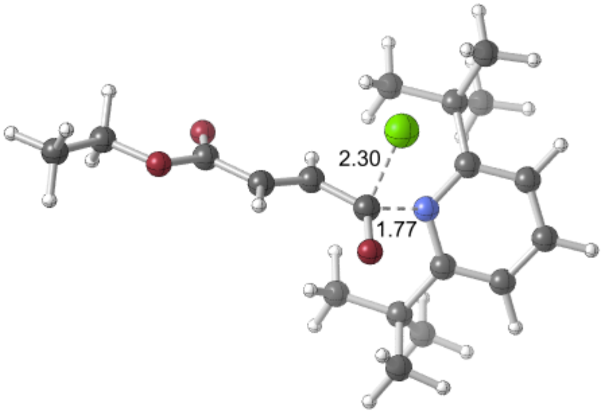

Charge = 0 Multiplicity = 1

HF = -1481.0001713 hartrees (-929342.417492463 kcal/mol)

Imaginary Frequencies: 1 (-232.4717 1/cm)

Zero-point correction = 0.445757 (Hartree/Particle)

Temperature 298.150 Kelvin. Pressure 1.00000 Atm.

Sum of electronic and thermal Free Energies =

-1480.609899 hartrees (-929097.51772149 kcal/mol)

Coordinates (from last standard orientation):
atom 17 is isolated, type=Cl

Center Number	Atomic Number	Coordinates (Angstroms) X	Y	Z
1	6	-0.020012	0.261614	-0.808756
2	6	-1.199251	-0.074500	0.030128
3	6	-2.419489	0.161775	-0.448790
4	6	-3.596133	-0.128174	0.410146
5	6	-5.945145	-0.108974	0.514245
6	6	-7.107235	0.254588	-0.379933
7	1	-1.057173	-0.454395	1.030239
8	1	-5.962673	-1.164554	0.800704
9	1	-5.927504	0.491235	1.428613
10	1	-7.101125	-0.350628	-1.290592
11	1	-8.046539	0.073514	0.150119
12	1	-7.066644	1.310893	-0.660260
13	1	-2.591307	0.543039	-1.450271
14	8	0.047819	1.125152	-1.621404
15	8	-3.537502	-0.556401	1.542283

16	8	-4.736511	0.145565	-0.226635
17	17	0.117559	-1.687410	-2.020787
18	6	2.167395	1.352775	-0.077982
19	6	3.479419	1.420110	-0.519810
20	6	4.168510	0.251348	-0.814055
21	6	3.595408	-0.958662	-0.468424
22	6	2.286890	-1.016203	0.010653
23	7	1.540535	0.134851	0.017240
24	1	5.170359	0.289829	-1.230398
25	1	3.959879	2.384440	-0.632763
26	1	4.173464	-1.871673	-0.535680
27	6	1.888769	-2.308494	0.749817
28	6	1.510773	2.657443	0.413523
29	6	2.830733	-2.310900	1.984056
30	1	2.698752	-1.400822	2.580255
31	1	2.584998	-3.172621	2.613668
32	1	3.882935	-2.386105	1.695864
33	6	2.159668	-3.579074	-0.068733
34	1	1.974299	-4.448280	0.571723
35	1	1.497075	-3.633339	-0.934404
36	1	3.195153	-3.646087	-0.414099
37	6	2.436113	3.161786	1.548747
38	1	3.453845	3.359761	1.203399
39	1	2.028306	4.096368	1.947493
40	1	2.485250	2.433937	2.366301
41	6	1.458146	3.718215	-0.694739
42	1	0.821327	3.389693	-1.519314
43	1	1.044001	4.645033	-0.282840
44	1	2.452786	3.945461	-1.090713
45	6	0.118629	2.504718	1.036119
46	1	-0.671075	2.434126	0.287874
47	1	0.063699	1.638111	1.705067
48	1	-0.087389	3.395700	1.637434
49	6	0.468244	-2.368567	1.302714
50	1	-0.283563	-2.412710	0.514226
51	1	0.378163	-3.281539	1.900602
52	1	0.269419	-1.525827	1.972951

Product:

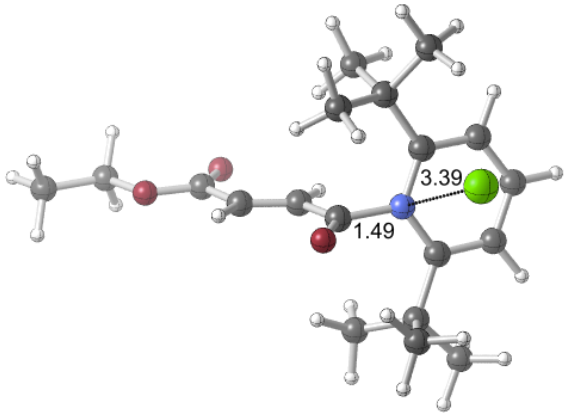

Charge = 0 Multiplicity = 1

HF = -1481.0337097 hartrees (-929363.463173847 kcal/mol)

Imaginary Frequencies: none found

Zero-point correction = 0.448553 (Hartree/Particle)

Temperature 298.150 Kelvin. Pressure 1.00000 Atm.

Sum of electronic and thermal Free Energies =

-1480.640709 hartrees (-929116.85130459 kcal/mol)

Coordinates (from last standard orientation):
atom 17 is isolated, type=Cl

Center Number	Atomic Number	X	Y	Z
1	6	-0.007259	0.294015	-0.601445
2	6	-1.252989	0.225290	0.194328
3	6	-2.434472	0.195735	-0.424172
4	6	-3.683429	0.099101	0.386808
5	6	-6.032758	0.073190	0.288251
6	6	-7.109061	0.167255	-0.767005
7	1	-1.177714	0.195087	1.277513
8	1	-6.071111	-0.874185	0.833799
9	1	-6.092697	0.889857	1.013419
10	1	-7.013915	-0.645285	-1.492495
11	1	-8.091289	0.093834	-0.292293
12	1	-7.050185	1.121473	-1.297544
13	1	-2.520154	0.227691	-1.506863
14	8	0.073316	0.494437	-1.774800
15	8	-3.702741	-0.020710	1.590947

16	8	-4.760624	0.157316	-0.388891
17	17	3.733184	-1.034320	-1.752138
18	6	1.920742	1.240135	0.591202
19	6	2.941885	1.071262	1.510658
20	6	3.225472	-0.179344	2.039680
21	6	2.536192	-1.284277	1.590070
22	6	1.535268	-1.161388	0.631210
23	7	1.220702	0.108685	0.222118
24	1	4.002976	-0.288513	2.788651
25	1	3.515340	1.924409	1.837998
26	1	2.787720	-2.262193	1.972200
27	6	0.827330	-2.449599	0.145685
28	6	1.627579	2.639019	0.003612
29	6	-0.276331	-2.831804	1.150849
30	1	-1.112950	-2.127795	1.152113
31	1	-0.670328	-3.815667	0.874081
32	1	0.123949	-2.899414	2.167960
33	6	1.872868	-3.589033	0.141259
34	1	1.415881	-4.464999	-0.326765
35	1	2.753908	-3.296067	-0.437571
36	1	2.174649	-3.893202	1.146257
37	6	2.394641	3.705498	0.808653
38	1	3.478139	3.580664	0.738039
39	1	2.155799	4.683144	0.381829
40	1	2.101604	3.715331	1.863934
41	6	2.162399	2.707204	-1.441314
42	1	1.691806	1.979038	-2.102146
43	1	1.971991	3.712195	-1.833655
44	1	3.242397	2.530880	-1.455415
45	6	0.139858	3.052391	0.066180
46	1	-0.467748	2.647580	-0.743397
47	1	-0.314128	2.786580	1.027278
48	1	0.088390	4.140757	-0.029190
49	6	0.254430	-2.406535	-1.283913
50	1	0.981059	-1.980936	-1.982079
51	1	0.049790	-3.437895	-1.584502
52	1	-0.696009	-1.876741	-1.361265

*Acylammonium formation between ethyl fumaroyl chloride (**5**) and Et_3N:*

Reactant:

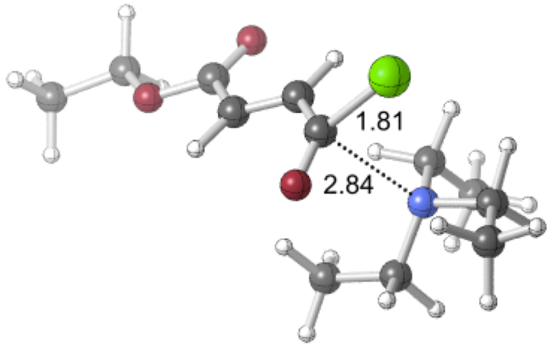

Charge = 0 Multiplicity = 1

HF = -1210.7783215 hartrees (-759775.504524465 kcal/mol)

Imaginary Frequencies: none found

Zero-point correction = 0.337212 (Hartree/Particle)

Temperature 298.150 Kelvin. Pressure 1.00000 Atm.

Sum of electronic and thermal Free Energies =

-1210.492958 hartrees (-759596.43607458 kcal/mol)

Coordinates (from last standard orientation):

Center Number	Atomic Number	Coordinates (Angstroms) X	Y	Z
1	6	-0.858953	-1.703864	0.361891
2	6	0.290086	-1.127550	-0.366335
3	6	1.447018	-0.939192	0.267639
4	6	2.593417	-0.334996	-0.466320
5	6	4.842605	0.330693	-0.290078
6	6	5.940506	0.305754	0.747094
7	1	0.168670	-0.889655	-1.417675
8	1	5.107232	-0.241942	-1.183433
9	1	4.590594	1.349489	-0.598719
10	1	6.169334	-0.721201	1.045039
11	1	6.846442	0.754629	0.330508
12	1	5.649503	0.873665	1.635008
13	1	1.585087	-1.198490	1.313129
14	8	-1.021849	-1.773919	1.536513
15	8	2.546319	0.053251	-1.612290
16	8	3.674264	-0.266072	0.308754

17	17	-2.021418	-2.509869	-0.767227
18	7	-1.989306	0.881464	0.001965
19	6	-3.392292	0.656098	-0.356017
20	1	-3.920685	1.609130	-0.537905
21	6	-1.271549	1.562517	-1.081308
22	1	-0.195945	1.491223	-0.881537
23	1	-1.454255	0.981432	-1.994198
24	6	-1.873578	1.557594	1.296100
25	1	-2.341420	0.911191	2.045023
26	1	-2.440677	2.507472	1.300108
27	6	-1.635808	3.028091	-1.331920
28	1	-1.386563	3.659332	-0.472443
29	1	-1.072247	3.402374	-2.192589
30	1	-2.700348	3.156781	-1.550530
31	6	-0.437401	1.811278	1.731036
32	1	0.089414	2.504450	1.067815
33	1	0.121460	0.871776	1.767381
34	6	-4.170047	-0.137809	0.689925
35	1	-3.589418	-0.993985	1.047130
36	1	-4.444131	0.474778	1.553621
37	1	-5.097761	-0.515055	0.248309
38	1	-3.394913	0.106258	-1.304299
39	1	-0.434028	2.246113	2.735303

Transition state (TS):

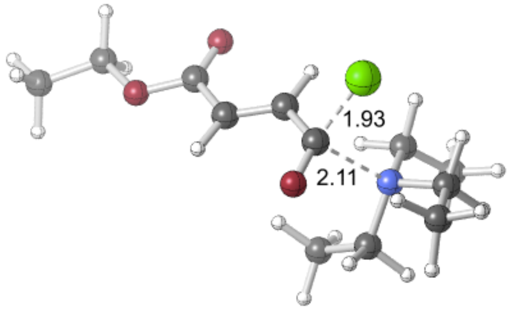

Charge = 0 Multiplicity = 1

HF = -1210.7743959 hartrees (-759773.041171209 kcal/mol)

Imaginary Frequencies: 1 (-131.8994 1/cm)

Zero-point correction = 0.338405 (Hartree/Particle)

Temperature 298.150 Kelvin. Pressure 1.00000 Atm.

Sum of electronic and thermal Free Energies =

-1210.484528 hartrees (-759591.14616528 kcal/mol)

Coordinates (from last standard orientation):

Center Number	Atomic Number	Coordinates (Angstroms) X	Y	Z
1	6	-0.812985	-1.058155	0.367390
2	6	0.396742	-0.570356	-0.354985
3	6	1.562618	-0.510442	0.286568
4	6	2.785491	-0.092914	-0.448496
5	6	5.104341	0.256549	-0.265958
6	6	6.171428	0.179522	0.800838
7	1	0.321683	-0.346568	-1.412826
8	1	5.304229	-0.427215	-1.096420
9	1	5.003038	1.266685	-0.673408
10	1	6.252717	-0.836519	1.196936
11	1	7.137007	0.462994	0.372801
12	1	5.945368	0.860861	1.625856
13	1	1.654134	-0.763562	1.338526
14	8	-0.902151	-1.284163	1.539226
15	8	2.814854	0.230521	-1.615667
16	8	3.856459	-0.119997	0.347016
17	17	-1.609345	-2.329916	-0.850518
18	7	-2.154589	0.537328	0.025845
19	6	-3.531938	0.015488	-0.143181
20	1	-4.215624	0.868940	-0.244641
21	6	-1.753611	1.305022	-1.172693
22	1	-0.716155	1.618205	-1.034296
23	1	-1.772662	0.601660	-2.013551
24	6	-2.078299	1.310244	1.284054
25	1	-2.163500	0.580751	2.094013
26	1	-2.953731	1.972516	1.346775
27	6	-2.605191	2.531035	-1.495912
28	1	-2.660759	3.219879	-0.646657
29	1	-2.141922	3.067126	-2.329563
30	1	-3.622438	2.267914	-1.796092
31	6	-0.803165	2.114628	1.480040
32	1	-0.693306	2.919954	0.747950
33	1	0.083732	1.478669	1.435936
34	6	-4.028752	-0.868727	0.994611
35	1	-3.329204	-1.671865	1.230587
36	1	-4.220387	-0.293638	1.904721
37	1	-4.977082	-1.319555	0.686279
38	1	-3.550995	-0.535060	-1.086235
39	1	-0.836521	2.572842	2.472943

Product:

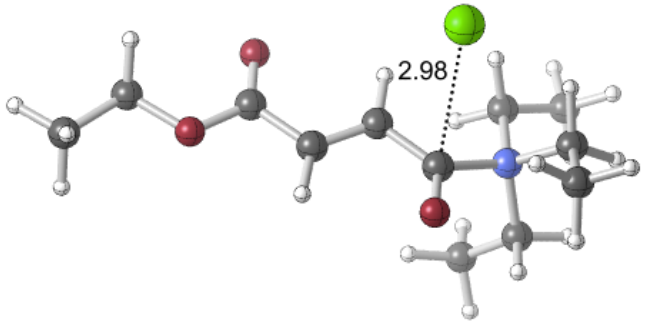

Charge = 0 Multiplicity = 1

HF = -1210.7906184 hartrees (-759783.220952184 kcal/mol)

Imaginary Frequencies: none found

Zero-point correction = 0.340948 (Hartree/Particle)

Temperature 298.150 Kelvin. Pressure 1.00000 Atm.

Sum of electronic and thermal Free Energies =

-1210.499612 hartrees (-759600.61152612 kcal/mol)

Coordinates (from last standard orientation):

Center Number	Atomic Number	Coordinates (Angstroms) X	Y	Z
1	6	0.842040	-0.022438	-0.684159
2	6	-0.373239	-0.056806	0.165040
3	6	-1.554578	0.126218	-0.429483
4	6	-2.794040	0.102788	0.391025
5	6	-5.144102	0.140221	0.309974
6	6	-6.230179	0.228388	-0.736412
7	1	-0.311885	-0.213988	1.232035
8	1	-5.196507	-0.792919	0.878562
9	1	-5.186085	0.973535	1.017664
10	1	-6.153946	-0.601038	-1.444913
11	1	-7.209222	0.180225	-0.251721
12	1	-6.162426	1.169727	-1.288763
13	1	-1.650132	0.268364	-1.501248
14	8	0.835910	-0.114852	-1.875736
15	8	-2.820793	0.018918	1.598960
16	8	-3.879763	0.185114	-0.379457
17	17	0.948032	-2.722907	0.561995
18	7	2.183810	0.406643	-0.047954
19	6	3.293992	-0.624507	-0.287082

20	1	4.214674	-0.085508	-0.059267
21	6	2.047143	0.614889	1.446800
22	1	1.217658	1.305934	1.598238
23	1	1.771514	-0.365305	1.844057
24	6	2.570669	1.695891	-0.759365
25	1	2.610545	1.444205	-1.818629
26	1	3.579616	1.935671	-0.422046
27	6	3.292999	1.153223	2.127544
28	1	3.581658	2.140625	1.756649
29	1	3.053360	1.258406	3.189278
30	1	4.144417	0.474455	2.048468
31	6	1.614903	2.846784	-0.506151
32	1	1.639655	3.185890	0.532273
33	1	0.583098	2.599835	-0.774815
34	6	3.376604	-1.236540	-1.671438
35	1	2.525860	-1.887026	-1.874842
36	1	3.474005	-0.495960	-2.468431
37	1	4.282392	-1.851465	-1.674004
38	1	3.124258	-1.405349	0.452994
39	1	1.928167	3.682491	-1.137677

*Acylammonium formation between ethyl fumaroyl chloride (**5**) and Hünig's base:*

Reactant:

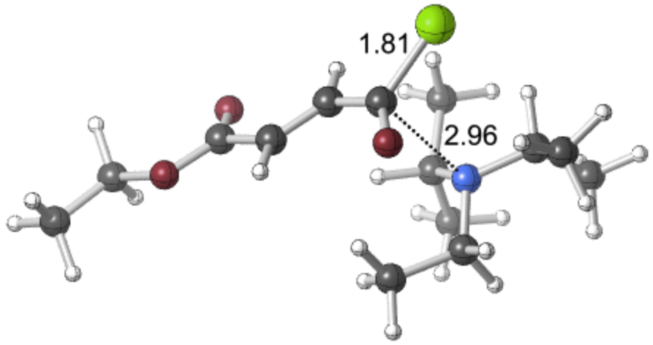

Charge = 0 Multiplicity = 1

HF = -1289.3633803 hartrees (-809088.414772053 kcal/mol)

Imaginary Frequencies: none found

Zero-point correction = 0.394577 (Hartree/Particle)

Temperature 298.150 Kelvin. Pressure 1.00000 Atm.

Sum of electronic and thermal Free Energies =

-1289.022464 hartrees (-808874.48638464 kcal/mol)

Coordinates (from last standard orientation):

Center Number	Atomic Number	Coordinates (Angstroms) X	Y	Z
1	6	-0.384596	-1.942868	0.294707
2	6	0.697016	-1.204357	-0.388663
3	6	1.837303	-0.952673	0.254170
4	6	2.915324	-0.200794	-0.448412
5	6	5.131376	0.570042	-0.287479
6	6	6.247797	0.563350	0.729788
7	1	0.553296	-0.904991	-1.421322
8	1	5.413440	0.057151	-1.211720
9	1	4.811500	1.584345	-0.542556
10	1	6.536262	-0.460858	0.981706
11	1	7.120326	1.077752	0.317714
12	1	5.942008	1.078055	1.644817
13	1	2.014251	-1.273546	1.276578
14	8	-0.512805	-2.138040	1.460220
15	8	2.802637	0.284368	-1.552128
16	8	4.014704	-0.129936	0.298510
17	17	-1.510495	-2.724170	-0.881980
18	7	-1.830511	0.638211	0.277162
19	6	-3.212605	0.261915	-0.085477
20	6	-1.117129	1.512442	-0.678976
21	1	-0.049574	1.347844	-0.477802
22	6	-1.706909	1.086647	1.665731
23	1	-2.183884	0.334393	2.299339
24	1	-2.244434	2.033851	1.845692
25	6	-1.347858	3.026064	-0.530984
26	1	-1.124531	3.380867	0.479691
27	1	-0.677949	3.555586	-1.217844
28	1	-2.371876	3.321005	-0.775112
29	6	-0.267172	1.230142	2.147760
30	1	0.299318	1.985459	1.594427
31	1	0.257613	0.274615	2.070272
32	6	-3.777754	-0.779588	0.887397
33	1	-3.021500	-1.514214	1.177334
34	1	-4.174702	-0.315906	1.797130
35	1	-4.604185	-1.311677	0.405461
36	1	-3.140195	-0.228694	-1.061632
37	1	-0.271223	1.528400	3.201203
38	6	-1.355113	1.091726	-2.128359
39	1	-0.606775	1.568100	-2.769734
40	1	-1.272290	0.007957	-2.258052
41	1	-2.343748	1.398555	-2.487214

42	6	-4.217241	1.417658	-0.217850
43	1	-4.233343	2.039105	0.684888
44	1	-4.001045	2.059434	-1.075477
45	1	-5.225696	1.011448	-0.357200

Transition state (TS):

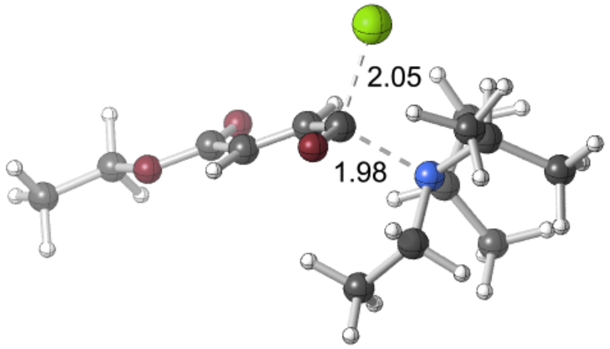

Charge = 0 Multiplicity = 1

HF = -1289.3534775 hartrees (-809082.200666025 kcal/mol)

Imaginary Frequencies: 1 (-194.0286 1/cm)

Zero-point correction = 0.396673 (Hartree/Particle)

Temperature 298.150 Kelvin. Pressure 1.00000 Atm.

Sum of electronic and thermal Free Energies =

-1289.006201 hartrees (-808864.28118951 kcal/mol)

Coordinates (from last standard orientation):

Center Number	Atomic Number	Coordinates (Angstroms) X	Y	Z
1	6	-0.476335	-1.026409	0.443975
2	6	0.715802	-0.516416	-0.301024
3	6	1.892962	-0.459722	0.320492
4	6	3.099974	-0.029697	-0.432221
5	6	5.420522	0.328906	-0.285039
6	6	6.503061	0.255975	0.766455
7	1	0.629769	-0.275774	-1.353647
8	1	5.612297	-0.352432	-1.119466
9	1	5.307972	1.339121	-0.689308
10	1	6.594567	-0.760107	1.160215
11	1	7.461382	0.544596	0.325786
12	1	6.284847	0.935197	1.595354

13	1	2.007166	-0.729078	1.366030
14	8	-0.480897	-1.383615	1.586449
15	8	3.109333	0.302709	-1.597372
16	8	4.184250	-0.055795	0.345683
17	17	-1.117000	-2.471957	-0.865548
18	7	-1.880029	0.351767	0.244422
19	6	-3.207423	-0.314095	-0.032926
20	6	-1.468783	1.358209	-0.801863
21	1	-0.406061	1.520730	-0.604452
22	6	-1.871929	0.940313	1.612664
23	1	-1.995051	0.107213	2.304490
24	1	-2.747289	1.587662	1.720406
25	6	-2.126457	2.745561	-0.716117
26	1	-2.201721	3.135296	0.300779
27	1	-1.494702	3.436768	-1.283797
28	1	-3.119061	2.770937	-1.167350
29	6	-0.606020	1.694495	1.999627
30	1	-0.404820	2.568133	1.374574
31	1	0.269856	1.043376	1.980772
32	6	-3.578211	-1.324746	1.055606
33	1	-2.738937	-1.947611	1.364512
34	1	-3.993461	-0.826233	1.938107
35	1	-4.356059	-1.979960	0.651989
36	1	-3.052846	-0.861126	-0.962509
37	1	-0.731679	2.047922	3.027652
38	6	-1.614466	0.813442	-2.221162
39	1	-1.062639	1.471118	-2.899900
40	1	-1.223462	-0.200093	-2.334224
41	1	-2.659913	0.808862	-2.543544
42	6	-4.384410	0.645899	-0.236506
43	1	-4.481565	1.375807	0.572971
44	1	-4.340310	1.177894	-1.187313
45	1	-5.300282	0.046011	-0.244615

Product:

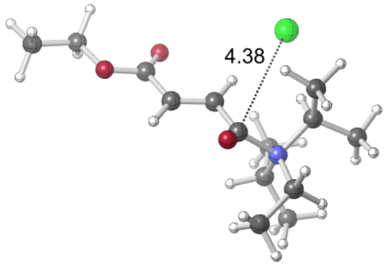

Charge = 0 Multiplicity = 1

HF = -1289.364557 hartrees (-809089.15316307 kcal/mol)

Imaginary Frequencies: none found

Zero-point correction = 0.397584 (Hartree/Particle)

Temperature 298.150 Kelvin. Pressure 1.00000 Atm.

Sum of electronic and thermal Free Energies =

-1289.021178 hartrees (-808873.67940678 kcal/mol)

Coordinates (from last standard orientation):

Center Number	Atomic Number	Coordinates (Angstroms) X	Y	Z
1	6	-0.593365	-0.853838	-0.763860
2	6	0.578926	-0.063923	-0.315641
3	6	1.752983	-0.696960	-0.270789
4	6	2.988213	0.021166	0.156849
5	6	5.333751	-0.112954	0.246203
6	6	6.418908	-1.090558	-0.139375
7	1	0.477004	1.001137	-0.088333
8	1	1.849494	-1.745077	-0.541019
9	8	-0.552456	-1.690818	-1.615669
10	8	3.007264	1.112822	0.673928
11	8	4.070709	-0.711071	-0.108711
12	17	-0.193118	3.456694	-0.131252
13	7	-1.933271	-0.570201	-0.091895
14	6	-2.359877	0.843528	-0.583142
15	6	-1.726263	-0.655443	1.466288
16	1	-0.908144	-1.375015	1.569433
17	6	-2.932874	-1.611710	-0.561743
18	1	-3.074462	-1.445417	-1.628653
19	1	-3.860429	-1.355377	-0.055742
20	6	-2.937823	-1.206064	2.218590

21	1	-3.230602	-2.212761	1.921832
22	1	-2.644119	-1.252919	3.271080
23	1	-3.804946	-0.545378	2.152235
24	6	-2.538669	-3.065844	-0.292104
25	1	-1.824736	-3.171535	0.528916
26	1	-2.096172	-3.524179	-1.175547
27	6	-2.145822	1.007865	-2.083945
28	1	-1.097723	0.916396	-2.381730
29	1	-2.753113	0.324994	-2.685034
30	1	-2.447050	2.030072	-2.328267
31	1	-1.678649	1.537716	-0.086772
32	1	-3.435508	-3.629075	-0.020807
33	6	-1.313000	0.666935	2.107666
34	1	-1.040630	0.433771	3.140943
35	1	-0.459756	1.166900	1.649709
36	1	-2.146183	1.375268	2.136089
37	6	-3.792910	1.180201	-0.199627
38	1	-4.522795	0.631517	-0.801675
39	1	-4.006223	1.020952	0.858711
40	1	-3.926482	2.247082	-0.401700
41	1	5.329253	0.095041	1.320179
42	1	5.428747	0.838320	-0.285233
43	1	6.296355	-2.037747	0.393240
44	1	6.400150	-1.287247	-1.214885
45	1	7.395437	-0.671696	0.118960

***b.* Diels-Alder step of the DAL organocascade**

***i.* Acylammonium salts derived from Brønsted bases:**

Diene:

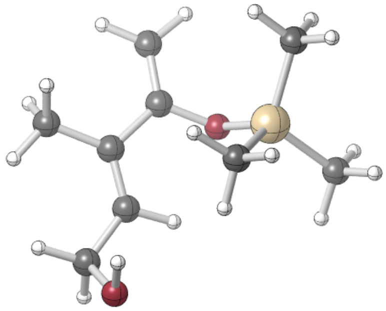

Charge = 0 Multiplicity = 1

HF = -793.4977666 hartrees (-497927.783519166 kcal/mol)

Imaginary Frequencies: none found

Zero-point correction = 0.255956 (Hartree/Particle)

Temperature   298.150 Kelvin. Pressure   1.00000 Atm.

Sum of electronic and thermal Free Energies =

-793.286374 hartrees (-497795.13254874 kcal/mol)

Coordinates (from last standard orientation):

Center Number	Atomic Number	Coordinates (Angstroms) X	Y	Z
1	6	-1.789481	-0.288014	-0.572437
2	6	0.380319	2.656051	-0.177700
3	6	-3.090830	-0.997507	-0.350989
4	6	-0.048458	1.411338	-0.423352
5	6	0.791570	-1.302206	1.391004
6	6	3.117543	0.624855	0.841751
7	6	2.564231	-1.710651	-1.107445
8	6	-1.445877	0.955064	-0.200382
9	6	-2.378693	1.946485	0.443736
10	1	-1.034051	-0.907485	-1.051103
11	1	1.413073	2.925175	-0.374643
12	1	-0.283560	3.425212	0.196776
13	1	-3.831018	-0.372046	0.161086
14	1	-3.524191	-1.299905	-1.310550

15	1	0.046637	-1.982972	0.964440
16	1	1.448027	-1.888470	2.045693
17	1	0.266990	-0.572411	2.018642
18	1	3.697007	1.161466	0.082416
19	1	2.678684	1.363738	1.521178
20	1	3.816673	0.014203	1.425967
21	1	3.238273	-2.390030	-0.573475
22	1	1.786506	-2.316488	-1.585546
23	1	3.140843	-1.217469	-1.897471
24	1	-2.480754	-1.965170	1.218249
25	1	-1.969850	2.302424	1.395567
26	1	-2.512158	2.823853	-0.199200
27	1	-3.365079	1.520991	0.631188
28	8	0.810376	0.473233	-0.934987
29	8	-2.871154	-2.210857	0.364149
30	14	1.810874	-0.464008	0.059455

Ethyl fumaroyl chloride (**5**):

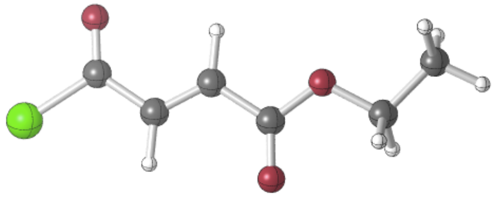

Charge = 0 Multiplicity = 1

HF = -918.5039922 hartrees (-576370.440145422 kcal/mol)

Imaginary Frequencies: none found

Zero-point correction = 0.126267 (Hartree/Particle)

Temperature 298.150 Kelvin. Pressure 1.00000 Atm.

Sum of electronic and thermal Free Energies =

-918.416338 hartrees (-576315.43625838 kcal/mol)

Coordinates (from last standard orientation):

Center Number	Atomic Number	Coordinates (Angstroms) X	Y	Z
1	6	-2.404785	0.535097	-0.020979
2	6	0.008958	0.346013	-0.031898
3	6	-1.169862	-0.277188	-0.025157
4	6	1.268965	-0.453229	-0.032192

5	6	3.616705	-0.317867	-0.008249
6	6	4.675205	0.755901	0.080148
7	1	0.088461	1.428999	-0.034584
8	1	-1.250380	-1.358672	-0.017684
9	1	3.699592	-0.904604	-0.927911
10	1	3.655164	-1.005150	0.841606
11	1	4.570953	1.326879	1.006880
12	1	5.665622	0.292605	0.068215
13	1	4.604344	1.443500	-0.767095
14	8	-2.491100	1.719758	-0.059931
15	8	2.334266	0.343047	-0.005009
16	8	1.311464	-1.661878	-0.054280
17	17	-3.896583	-0.468432	0.059665

*Acylammonium dienophile derived from ethyl fumaroyl chloride (**5**) and pyridine:*

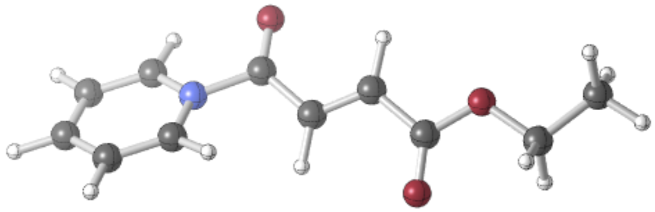

Charge = 1 Multiplicity = 1

HF = -706.3614774 hartrees (-443248.890683274 kcal/mol)

Imaginary Frequencies: none found

Zero-point correction = 0.219936 (Hartree/Particle)

Temperature 298.150 Kelvin. Pressure 1.00000 Atm.

Sum of electronic and thermal Free Energies =

-706.184460 hartrees (-443137.8104946 kcal/mol)

Coordinates (from last standard orientation):

Center Number	Atomic Number	Coordinates (Angstroms) X	Y	Z
1	6	1.051231	0.913486	-0.033286
2	6	-0.104591	0.030699	0.197673
3	6	-1.324891	0.487782	-0.089769
4	6	-2.511005	-0.381272	0.178032
5	6	-4.855612	-0.505437	0.083648
6	6	-6.008857	0.387741	-0.305468
7	1	0.032740	-0.956437	0.627479

8	1	-4.816468	-1.416647	-0.519783
9	1	-4.892207	-0.792768	1.138021
10	1	-5.943936	0.670577	-1.359698
11	1	-6.949916	-0.146518	-0.148541
12	1	-6.020433	1.295617	0.303588
13	1	-1.488608	1.473743	-0.515198
14	8	1.022942	2.097287	-0.199073
15	8	-2.436884	-1.500857	0.629671
16	8	-3.638609	0.240541	-0.141916
17	6	3.446761	1.022276	0.323927
18	6	4.714337	0.482942	0.322625
19	6	4.886892	-0.846616	-0.059652
20	6	3.781412	-1.607507	-0.434671
21	6	2.529101	-1.032120	-0.407323
22	7	2.385651	0.257686	-0.025419
23	1	5.878869	-1.286061	-0.069007
24	1	3.225619	2.046497	0.597662
25	1	5.552403	1.101988	0.618259
26	1	3.880167	-2.638244	-0.752242
27	1	1.633962	-1.563159	-0.706486

*Acylammonium dienophile derived from ethyl fumaroyl chloride (**5**) and 2,6-lutidine:*

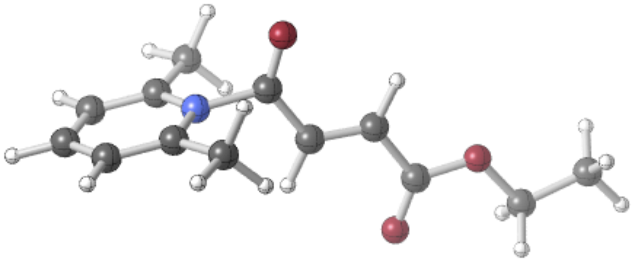

Charge = 1 Multiplicity = 1

HF = -784.9616092 hartrees (-492571.259389092 kcal/mol)

Imaginary Frequencies: none found

Zero-point correction = 0.274661 (Hartree/Particle)

Temperature 298.150 Kelvin. Pressure 1.00000 Atm.

Sum of electronic and thermal Free Energies =

-784.734120 hartrees (-492428.5076412 kcal/mol)

Coordinates (from last standard orientation):

Center Number	Atomic Number	Coordinates (Angstroms) X	Y	Z
1	6	-0.787054	-0.033321	0.930371
2	6	0.359096	-0.017980	0.008600
3	6	1.601895	-0.017905	0.496266
4	6	2.756148	0.001628	-0.453823
5	6	5.099831	0.013430	-0.624761
6	6	6.291154	0.000536	0.302689
7	1	0.166558	-0.003692	-1.060937
8	1	5.079417	-0.859866	-1.282792
9	1	5.070358	0.913310	-1.245419
10	1	6.295332	-0.902597	0.918944
11	1	7.209916	0.018954	-0.290127
12	1	6.283984	0.875940	0.957782
13	1	1.806036	-0.029515	1.563365
14	8	-0.772960	-0.062317	2.124348
15	8	2.636389	0.025966	-1.657141
16	8	3.911748	-0.009572	0.198240
17	6	-2.675414	1.209324	-0.016083
18	6	-3.917356	1.237094	-0.628037
19	6	-4.555280	0.045925	-0.955086
20	6	-3.947625	-1.171437	-0.669025
21	6	-2.706222	-1.196089	-0.055782
22	7	-2.118469	-0.005197	0.243174
23	1	-5.529112	0.066451	-1.433437
24	1	-4.371209	2.198863	-0.837003
25	1	-4.425535	-2.113414	-0.911950
26	6	-1.921525	2.441689	0.373492
27	1	-1.740387	2.467632	1.452495
28	1	-0.954949	2.491582	-0.140218
29	1	-2.503801	3.320486	0.096457
30	6	-1.987058	-2.460020	0.296750
31	1	-1.004000	-2.503719	-0.185596
32	1	-1.843670	-2.539581	1.378954
33	1	-2.574614	-3.314327	-0.039752

*Acylammonium dienophile derived from ethyl fumaroyl chloride (**5**) and DTBP:*

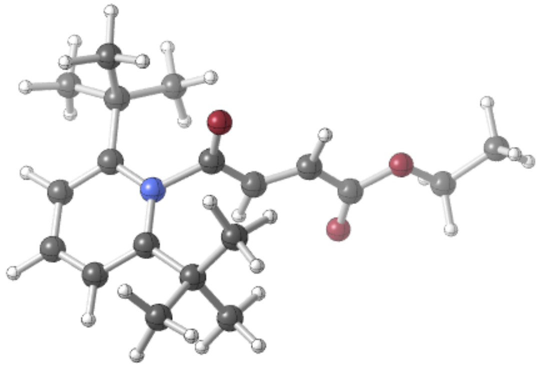

Charge = 1 Multiplicity = 1

HF = -1020.7003767 hartrees (-640499.693383017 kcal/mol)

Imaginary Frequencies: none found

Zero-point correction = 0.447918 (Hartree/Particle)

Temperature   298.150 Kelvin. Pressure   1.00000 Atm.

Sum of electronic and thermal Free Energies =

-1020.305883 hartrees (-640252.14464133 kcal/mol)

Coordinates (from last standard orientation):

Center Number	Atomic Number	Coordinates (Angstroms) X	Y	Z
1	6	0.289212	-0.196706	0.803466
2	6	-0.878713	-0.095559	-0.093134
3	6	-2.109334	-0.179559	0.415885
4	6	-3.284767	-0.064710	-0.498459
5	6	-5.623235	-0.253665	-0.647511
6	6	-6.778905	-0.586669	0.265263
7	1	-0.708760	0.050499	-1.156527
8	1	-5.706469	0.751099	-1.070996
9	1	-5.528170	-0.967951	-1.470270
10	1	-6.851596	0.137145	1.081523
11	1	-7.710989	-0.559413	-0.305836
12	1	-6.662142	-1.587321	0.690124
13	1	-2.287104	-0.330009	1.477568
14	8	0.286367	-0.462575	1.966884
15	8	-3.199329	0.217234	-1.671876
16	8	-4.417374	-0.314292	0.146490
17	6	2.319380	-1.030330	-0.311334

18	6	3.408117	-0.780342	-1.132798
19	6	3.730968	0.512022	-1.515143
20	6	2.996324	1.575958	-1.028419
21	6	1.910526	1.369891	-0.185518
22	7	1.587693	0.065405	0.105042
23	1	4.567866	0.688545	-2.182905
24	1	4.006898	-1.601955	-1.493933
25	1	3.268217	2.583172	-1.306994
26	6	1.147629	2.612333	0.337801
27	6	2.005538	-2.473002	0.143605
28	6	0.147549	3.076770	-0.739662
29	1	-0.679529	2.373968	-0.873967
30	1	-0.276800	4.036509	-0.426592
31	1	0.644408	3.219374	-1.704543
32	6	2.178182	3.747651	0.546078
33	1	1.671200	4.578445	1.043049
34	1	3.010551	3.426724	1.180302
35	1	2.576168	4.137900	-0.392911
36	6	2.851204	-3.464009	-0.678445
37	1	3.923945	-3.336018	-0.511971
38	1	2.593375	-4.474064	-0.350475
39	1	2.641299	-3.392222	-1.750681
40	6	2.433649	-2.636443	1.615880
41	1	1.876944	-1.987313	2.292586
42	1	2.258287	-3.675953	1.912186
43	1	3.501829	-2.424196	1.728203
44	6	0.534262	-2.898756	-0.057788
45	1	-0.136989	-2.560658	0.731870
46	1	0.147060	-2.571283	-1.028631
47	1	0.496385	-3.991486	-0.040973
48	6	0.421180	2.454883	1.687670
49	1	1.057225	1.989623	2.446375
50	1	0.163091	3.458879	2.033526
51	1	-0.519959	1.908655	1.618987

*Acylammonium dienophile derived from ethyl fumaroyl chloride (**5**) and Et_3N:*

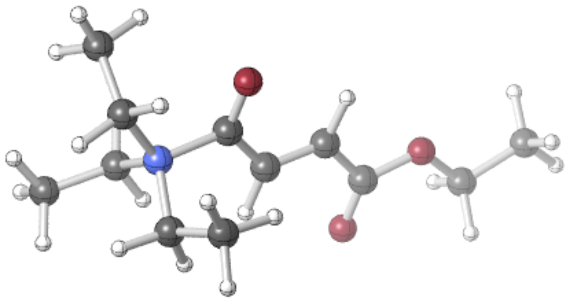

Charge = 1 Multiplicity = 1

HF = -750.4551881 hartrees (-470918.135084631 kcal/mol)

Imaginary Frequencies: none found

Zero-point correction = 0.340766 (Hartree/Particle)

Temperature 298.150 Kelvin. Pressure 1.00000 Atm.

Sum of electronic and thermal Free Energies =

-750.161330 hartrees (-470733.7361883 kcal/mol)

Coordinates (from last standard orientation):

Center Number	Atomic Number	Coordinates (Angstroms) X	Y	Z
1	6	-0.931358	0.662278	-0.039854
2	6	0.281412	-0.180919	-0.009686
3	6	1.471248	0.407580	-0.147895
4	6	2.711337	-0.424495	-0.085710
5	6	5.061738	-0.356668	-0.111688
6	6	6.148448	0.686966	-0.211790
7	1	0.218829	-1.252410	0.144774
8	1	5.104612	-0.909034	0.831384
9	1	5.101700	-1.076428	-0.934288
10	1	6.071386	1.407625	0.606962
11	1	7.125669	0.200154	-0.153041
12	1	6.085024	1.224444	-1.161770
13	1	1.576385	1.478252	-0.297077
14	8	-0.943578	1.853292	-0.128318
15	8	2.714073	-1.628139	0.035399
16	8	3.795328	0.336039	-0.174632
17	7	-2.263632	-0.068448	0.102818
18	6	-2.293775	-0.702762	1.492514
19	1	-3.303949	-1.095553	1.611670

20	6	-2.348011	-1.151215	-0.968240
21	1	-2.175077	-0.651231	-1.923436
22	1	-1.521840	-1.836498	-0.783210
23	6	-3.406958	0.923020	-0.027938
24	1	-3.185598	1.737952	0.659152
25	1	-4.287657	0.392449	0.336272
26	6	-3.659985	-1.915372	-0.968194
27	1	-4.525047	-1.276406	-1.158386
28	1	-3.601049	-2.646458	-1.779258
29	1	-3.816807	-2.467599	-0.039091
30	6	-3.621342	1.453529	-1.434294
31	1	-3.981443	0.687302	-2.124267
32	1	-2.719240	1.910571	-1.845329
33	6	-1.954289	0.265982	2.610445
34	1	-0.942993	0.673878	2.522410
35	1	-2.668818	1.089029	2.679658
36	1	-2.001039	-0.293980	3.548168
37	1	-1.595346	-1.543282	1.466156
38	1	-4.389647	2.229269	-1.372787

*Acylammonium dienophile derived from ethyl fumaroyl chloride (**5**) and Hünig's base:*

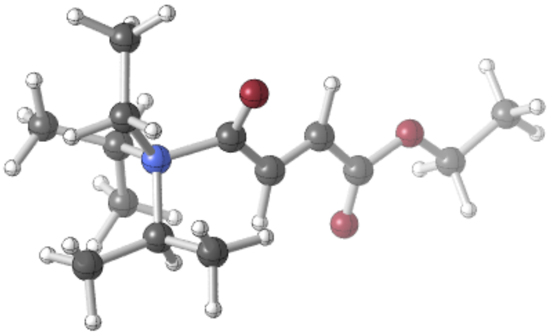

Charge = 1 Multiplicity = 1

HF = -829.0284781 hartrees (-520223.660292531 kcal/mol)

Imaginary Frequencies: none found

Zero-point correction = 0.398120 (Hartree/Particle)

Temperature 298.150 Kelvin. Pressure 1.00000 Atm.

Sum of electronic and thermal Free Energies =

-828.679307 hartrees (-520004.55193557 kcal/mol)

Coordinates (from last standard orientation):

Center Number	Atomic Number	Coordinates (Angstroms) X	Y	Z
1	6	0.624539	0.753464	-0.486237
2	6	-0.581148	-0.091066	-0.331366
3	6	-1.757283	0.495965	-0.108087
4	6	-2.977516	-0.357512	0.011718
5	6	-5.324435	-0.337195	0.143803
6	6	-6.436934	0.683380	0.125206
7	1	-0.521961	-1.168120	-0.438886
8	1	-1.865093	1.572402	-0.011177
9	8	0.608301	1.865377	-0.920370
10	8	-2.954692	-1.566715	0.052205
11	8	-4.076414	0.385769	0.057176
12	7	1.965213	0.148182	-0.074666
13	6	2.204708	-1.071719	-1.012046
14	6	1.868130	-0.234494	1.453700
15	1	1.170400	0.509346	1.849960
16	6	3.047226	1.194873	-0.296512
17	1	3.072746	1.386093	-1.367873
18	1	3.974269	0.699058	-0.020755
19	6	3.193564	-0.099292	2.202861
20	1	3.618380	0.903455	2.183636
21	1	2.975192	-0.336353	3.247887
22	1	3.943068	-0.812536	1.855100
23	6	2.870358	2.505551	0.476965
24	1	2.203437	2.414244	1.337940
25	1	2.467800	3.286923	-0.166021
26	6	1.912091	-0.725396	-2.469980
27	1	0.890561	-0.376201	-2.644389
28	1	2.612316	0.008914	-2.876195
29	1	2.035121	-1.648836	-3.042643
30	1	1.480129	-1.812709	-0.676106
31	1	3.845366	2.829453	0.849825
32	6	1.298360	-1.626729	1.711341
33	1	1.175866	-1.709024	2.794670
34	1	0.318272	-1.808755	1.271050
35	1	1.988569	-2.414816	1.399068
36	6	3.602342	-1.656283	-0.869146
37	1	4.367242	-0.996189	-1.285627
38	1	3.862106	-1.908602	0.159536
39	1	3.615369	-2.584073	-1.448000
40	1	-5.317471	-0.925116	1.065982
41	1	-5.381754	-1.026846	-0.702974

42	1	-6.344714	1.375923	0.966328
43	1	-6.423039	1.256277	-0.805908
44	1	-7.400244	0.172076	0.202811

DAL with acylammonium ion formed with pyridine (endo):

Transition state 1 (TS1):

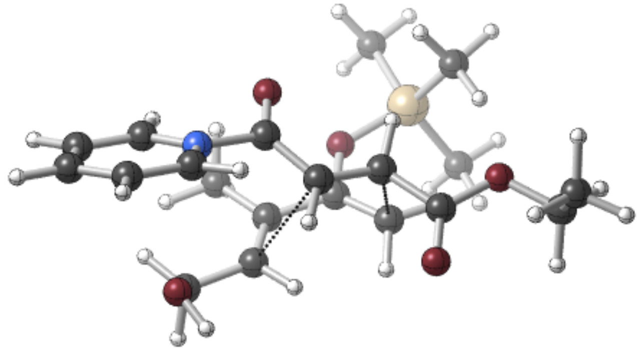

Charge = 1 Multiplicity = 1

HF = -1499.8681024 hartrees (-941182.232937024 kcal/mol)

Imaginary Frequencies: 1 (-297.0671 1/cm)

Zero-point correction = 0.478219 (Hartree/Particle)

Temperature 298.150 Kelvin. Pressure 1.00000 Atm.

Sum of electronic and thermal Free Energies =

-1499.450841 hartrees (-940920.39723591 kcal/mol)

Coordinates (from last standard orientation):

Center Number	Atomic Number	Coordinates (Angstroms) X	Y	Z
1	6	-1.355240	0.173294	-1.003274
2	6	-0.747325	1.241525	-0.289047
3	6	0.633895	1.300955	-0.316752
4	6	-1.187315	-0.652720	1.876975
5	6	1.533052	0.038267	1.187684
6	6	-2.634602	-0.816794	2.248355
7	6	0.977267	-1.199868	0.911087
8	6	4.397319	-2.115558	1.000714
9	6	3.448458	-0.722501	-1.623518
10	6	3.257881	-3.780939	-1.333078
11	6	-0.384384	-1.570836	1.288578

12	6	1.284944	2.521792	0.253564
13	6	3.242367	3.848862	0.212674
14	6	2.794661	5.096949	-0.519480
15	6	-0.834763	-2.941374	0.859380
16	1	-1.326200	1.920338	0.324222
17	1	-0.772525	0.303943	2.186320
18	1	2.576381	0.218004	0.949213
19	1	1.141130	0.618405	2.013249
20	1	1.166241	0.791768	-1.113316
21	1	-3.099349	-1.665423	1.742099
22	1	-2.712900	-1.001481	3.330088
23	1	5.421633	-2.250716	0.633950
24	1	4.368191	-1.177192	1.563093
25	1	4.182398	-2.935642	1.694809
26	1	2.653504	-0.708983	-2.378127
27	1	3.479217	0.256986	-1.134441
28	1	4.399771	-0.855361	-2.153621
29	1	3.066296	-4.613740	-0.647932
30	1	2.507807	-3.817385	-2.130246
31	1	4.241289	-3.940214	-1.789528
32	1	4.284676	3.609980	-0.002698
33	1	3.108354	3.941733	1.292893
34	1	3.412293	5.944420	-0.207906
35	1	2.905259	4.967784	-1.599889
36	1	1.750885	5.328597	-0.292770
37	1	-3.143829	1.054802	2.468788
38	1	-1.856517	-3.156760	1.173251
39	1	-0.764394	-3.033546	-0.230084
40	1	-0.181442	-3.709068	1.286223
41	8	-0.816479	-0.751259	-1.576328
42	8	1.605706	-2.117560	0.179954
43	8	0.764426	3.248094	1.070155
44	8	2.509660	2.691387	-0.246360
45	8	-3.395051	0.325879	1.878458
46	14	3.207061	-2.148815	-0.439494
47	6	-3.457648	-1.040482	-0.969792
48	6	-3.566331	1.310674	-0.988266
49	6	-4.833052	-1.130937	-0.904907
50	6	-4.945038	1.273513	-0.941431
51	6	-5.587943	0.039811	-0.885680
52	1	-5.298909	-2.108344	-0.870358
53	1	-5.499790	2.203717	-0.950257
54	1	-6.670652	-0.010062	-0.836172
55	7	-2.857951	0.165074	-0.993140
56	1	-3.001898	2.233244	-1.042918
57	1	-2.793437	-1.896050	-1.002772

Intermediate (INT):

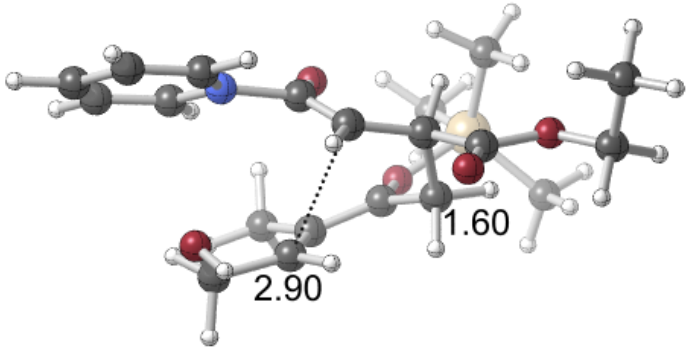

Charge = 1 Multiplicity = 1

HF = -1499.8843345 hartrees (-941192.418742095 kcal/mol)

Imaginary Frequencies: none found

Zero-point correction = 0.480252 (Hartree/Particle)

Temperature   298.150 Kelvin. Pressure   1.00000 Atm.

Sum of electronic and thermal Free Energies =

-1499.465718 hartrees (-940929.73270218 kcal/mol)

Coordinates (from last standard orientation):

Center Number	Atomic Number	Coordinates (Angstroms) X	Y	Z
1	6	-1.201483	0.258537	-0.891424
2	6	-0.611702	1.312436	-0.252096
3	6	0.859433	1.193255	-0.124291
4	6	-1.264632	-0.444087	1.966798
5	6	1.379626	0.132361	0.951937
6	6	-2.717790	-0.450965	2.349482
7	6	0.741168	-1.207925	0.895910
8	6	4.268675	-2.203147	0.691412
9	6	2.840457	-1.148944	-1.886318
10	6	2.674552	-4.148233	-1.116784
11	6	-0.568807	-1.479142	1.426914
12	6	1.518209	2.503375	0.263843
13	6	3.598488	3.631454	0.376236
14	6	3.502041	4.691514	-0.701071
15	6	-1.107603	-2.866021	1.201412
16	1	-1.144438	2.145636	0.183937

17	1	-0.759753	0.490047	2.191542
18	1	2.457922	0.029045	0.813493
19	1	1.213727	0.560785	1.943562
20	1	1.289505	0.861104	-1.073054
21	1	-3.250289	-1.291919	1.900081
22	1	-2.804854	-0.550750	3.441756
23	1	5.198741	-2.531883	0.212466
24	1	4.414020	-1.167887	1.016517
25	1	4.116170	-2.825768	1.579530
26	1	1.829832	-1.045076	-2.296582
27	1	3.193783	-0.164445	-1.561121
28	1	3.501844	-1.488952	-2.692331
29	1	2.599558	-4.851443	-0.280655
30	1	1.776106	-4.253442	-1.734295
31	1	3.538700	-4.438278	-1.724510
32	1	4.619485	3.262622	0.486181
33	1	3.243325	4.002158	1.340249
34	1	4.140121	5.540405	-0.437747
35	1	3.836740	4.293460	-1.663128
36	1	2.474672	5.051108	-0.802165
37	1	-3.042927	1.473092	2.405138
38	1	-2.133216	-2.969546	1.558029
39	1	-1.068135	-3.119643	0.137360
40	1	-0.494830	-3.600861	1.733928
41	8	-0.661205	-0.798505	-1.275373
42	8	1.375347	-2.220216	0.408545
43	8	0.948986	3.445109	0.764416
44	8	2.831597	2.461928	0.017651
45	8	-3.367685	0.720341	1.884520
46	14	2.857367	-2.402457	-0.510354
47	6	-3.359163	-0.862580	-0.922587
48	6	-3.357435	1.470130	-1.176588
49	6	-4.740499	-0.893642	-0.955094
50	6	-4.737348	1.495010	-1.222330
51	6	-5.441875	0.299913	-1.100994
52	1	-5.251023	-1.845297	-0.867586
53	1	-5.244430	2.442142	-1.361660
54	1	-6.526440	0.297911	-1.132539
55	7	-2.701989	0.306614	-1.014447
56	1	-2.744009	2.355805	-1.286344
57	1	-2.731326	-1.741098	-0.828238

Transition state 2 (TS2):

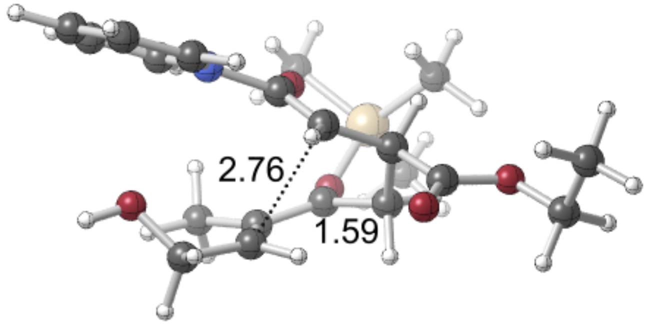

Charge = 1 Multiplicity = 1

HF = -1499.8828805 hartrees (-941191.506342555 kcal/mol)

Imaginary Frequencies: 1 (-49.0622 1/cm)

Zero-point correction = 0.480411 (Hartree/Particle)

Temperature 298.150 Kelvin. Pressure 1.00000 Atm.

Sum of electronic and thermal Free Energies =

-1499.463173 hartrees (-940928.13568923 kcal/mol)

Coordinates (from last standard orientation):

Center Number	Atomic Number	Coordinates (Angstroms) X	Y	Z
1	6	0.831220	-0.501253	-0.871777
2	6	-0.056110	-1.446814	-0.448111
3	6	-1.448501	-0.943081	-0.290633
4	6	0.756946	-0.610226	2.054671
5	6	-1.674492	0.186861	0.809718
6	6	1.995204	-1.313424	2.524951
7	6	-0.546911	1.126818	1.074234
8	6	-2.238910	4.692752	0.363690
9	6	-2.246467	2.376172	-1.694080
10	6	0.324296	4.010680	-1.249559
11	6	0.642694	0.716057	1.758675
12	6	-2.402112	-2.063886	0.073412
13	6	-4.683365	-2.685232	0.157449
14	6	-4.786278	-3.772037	-0.892474
15	6	1.714770	1.754302	1.952033
16	1	-0.127696	-1.237949	2.061910
17	1	-2.561486	0.757359	0.533390
18	1	-1.895617	-0.308088	1.761752

19	1	-1.791209	-0.485789	-1.224332
20	1	2.106940	-1.127271	3.605510
21	1	1.848991	-2.394111	2.398440
22	1	-1.639179	5.231901	1.104544
23	1	-2.602816	5.421467	-0.369355
24	1	-3.110400	4.269493	0.874862
25	1	-1.654992	1.553266	-2.105946
26	1	-3.180581	1.981493	-1.282228
27	1	-2.512399	3.041890	-2.525248
28	1	0.086846	4.675043	-2.088793
29	1	0.922500	4.579271	-0.529695
30	1	0.925426	3.178786	-1.629525
31	1	-5.600631	-2.097462	0.216156
32	1	-4.455259	-3.096728	1.143509
33	1	-5.615117	-4.441613	-0.644086
34	1	-4.976942	-3.336594	-1.877462
35	1	-3.866353	-4.360480	-0.934998
36	1	3.913846	-1.265560	2.221825
37	1	2.622084	1.313549	2.361094
38	1	1.952194	2.244332	1.002141
39	1	1.355358	2.532816	2.633913
40	8	0.582106	0.722160	-0.982733
41	8	-0.698371	2.399150	0.860707
42	8	-2.070151	-3.108957	0.586038
43	8	-3.664661	-1.723626	-0.191594
44	8	3.130930	-0.872094	1.804183
45	14	-1.240496	3.363329	-0.475219
46	6	3.175177	0.110060	-0.998380
47	6	2.650684	-2.165497	-1.214290
48	6	4.527154	-0.163059	-1.094678
49	6	3.986831	-2.493624	-1.301329
50	6	4.943711	-1.481193	-1.234889
51	1	5.235371	0.655730	-1.050288
52	1	4.266245	-3.531865	-1.434801
53	1	5.999770	-1.720391	-1.305650
54	7	2.270279	-0.882561	-1.048421
55	1	1.863447	-2.902812	-1.297609
56	1	2.763443	1.103245	-0.878002
57	1	0.171145	-2.480933	-0.233618

Product:

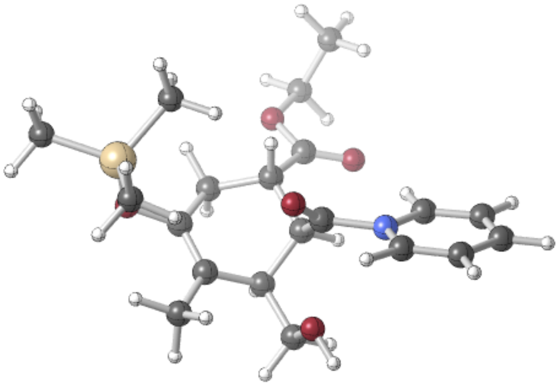

Charge = 1 Multiplicity = 1

HF = -1499.9394201 hartrees (-941226.985506951 kcal/mol)

Imaginary Frequencies: none found

Zero-point correction = 0.483631 (Hartree/Particle)

Temperature 298.150 Kelvin. Pressure 1.00000 Atm.

Sum of electronic and thermal Free Energies =

-1499.516793 hartrees (-940961.78277543 kcal/mol)

Coordinates (from last standard orientation):

Center Number	Atomic Number	Coordinates (Angstroms) X	Y	Z
1	6	1.162692	0.791527	-0.348331
2	6	0.826697	-0.099513	0.821398
3	6	-0.074447	-1.261957	0.330467
4	6	0.124983	0.731171	1.943061
5	6	-1.468991	-1.255507	0.991728
6	6	1.027554	1.850964	2.434524
7	6	-2.029025	0.144540	1.042632
8	6	-5.573386	0.317435	-1.057697
9	6	-2.769004	-0.461533	-2.121765
10	6	-3.335114	2.428620	-1.359028
11	6	-1.272753	1.142745	1.518618
12	6	0.657966	-2.587005	0.472750
13	6	0.409800	-4.939130	0.502728
14	6	0.894439	-5.357907	-0.869246
15	6	-1.747146	2.556283	1.682364
16	1	0.049072	0.025657	2.784023
17	1	-2.142090	-1.913801	0.439846

18	1	-1.383928	-1.657957	2.010393
19	1	-0.239705	-1.157856	-0.749856
20	1	0.523497	2.411721	3.230471
21	1	1.941530	1.411794	2.857854
22	1	-6.123963	0.931661	-0.336863
23	1	-5.956447	0.551991	-2.057406
24	1	-5.800754	-0.733332	-0.848177
25	1	-1.714096	-0.165598	-2.152901
26	1	-2.829327	-1.520656	-1.848858
27	1	-3.164132	-0.359455	-3.140022
28	1	-3.564800	2.634466	-2.411731
29	1	-3.912639	3.130002	-0.747630
30	1	-2.269321	2.632635	-1.206077
31	1	-0.398699	-5.580295	0.855948
32	1	1.217821	-4.938690	1.237584
33	1	1.270139	-6.384363	-0.824064
34	1	0.076082	-5.322050	-1.594109
35	1	1.704893	-4.709527	-1.212289
36	1	1.940115	3.398854	1.661108
37	1	-1.657335	2.881664	2.726315
38	1	-1.153149	3.248931	1.076543
39	1	-2.797095	2.643431	1.396472
40	8	0.395523	1.314385	-1.096668
41	8	-3.331328	0.296299	0.645975
42	8	1.865942	-2.695126	0.546659
43	8	-0.177918	-3.618483	0.461323
44	8	1.347615	2.699688	1.340295
45	14	-3.738411	0.644995	-0.960649
46	6	2.974934	2.224024	-1.105873
47	6	3.512311	0.009787	-0.475633
48	6	4.291473	2.481294	-1.421905
49	6	4.840016	0.219625	-0.793788
50	6	5.237755	1.469402	-1.261355
51	1	4.566816	3.462687	-1.788452
52	1	5.545332	-0.593941	-0.676890
53	1	6.278594	1.653755	-1.506311
54	7	2.620919	1.012639	-0.626589
55	1	3.128477	-0.945131	-0.126917
56	1	2.173293	2.945368	-1.205608
57	1	1.739950	-0.503677	1.258246

DAL with acylammonium ion formed with pyridine (exo):

Transition state (TS):

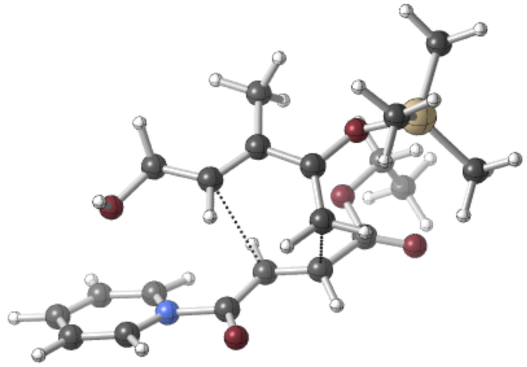

Charge = 1 Multiplicity = 1

HF = -1499.8657335 hartrees (-941180.746428585 kcal/mol)

Imaginary Frequencies: 1 (-300.5724 1/cm)

Zero-point correction = 0.477241 (Hartree/Particle)

Temperature 298.150 Kelvin. Pressure 1.00000 Atm.

Sum of electronic and thermal Free Energies =

-1499.450191 hartrees (-940919.98935441 kcal/mol)

Coordinates (from last standard orientation):

Center Number	Atomic Number	Coordinates (Angstroms) X	Y	Z
1	6	-2.239009	0.431928	-1.405567
2	6	0.056699	1.072106	-1.307164
3	6	-1.177675	1.029631	-0.672439
4	6	-0.986278	-1.473063	0.762492
5	6	0.946446	-0.855862	-1.326614
6	6	0.308702	-1.218421	1.064801
7	6	1.283341	-1.001132	0.008966
8	6	-2.096019	-1.564997	1.777240
9	6	4.526040	0.169636	-1.534949
10	6	3.862687	-2.816173	-1.035342
11	6	5.184539	-1.101136	1.184400
12	6	0.785967	-1.017336	2.478787
13	6	1.208455	1.796957	-0.691932
14	6	2.212258	2.479932	1.330448
15	6	2.150108	3.992421	1.281125
16	1	0.090687	1.041280	-2.392092

17	1	-1.275270	1.335575	0.358397
18	1	-1.282821	-1.700674	-0.258839
19	1	1.738472	-0.700213	-2.053406
20	1	0.034802	-1.294017	-1.718758
21	1	-1.844082	-2.299571	2.555660
22	1	-2.221593	-0.600172	2.285245
23	1	5.510984	-0.101293	-1.936318
24	1	3.830476	0.245834	-2.375699
25	1	4.608537	1.162812	-1.082859
26	1	3.468347	-3.533785	-0.307092
27	1	3.207081	-2.825394	-1.912206
28	1	4.847678	-3.175196	-1.356298
29	1	6.219446	-1.268944	0.866153
30	1	5.146304	-0.137358	1.704276
31	1	4.920823	-1.884329	1.903405
32	1	-3.323024	-2.786214	0.888461
33	1	1.052311	0.032517	2.644469
34	1	0.025181	-1.300665	3.208359
35	1	1.684965	-1.609993	2.670691
36	1	3.134730	2.097403	0.886534
37	1	2.127097	2.107028	2.352481
38	1	1.209683	4.353780	1.706898
39	1	2.976144	4.411044	1.863777
40	1	2.236430	4.350663	0.252335
41	8	-2.171591	-0.096388	-2.497411
42	8	2.517175	-0.727580	0.444239
43	8	-3.345681	-1.861831	1.184922
44	8	1.092753	1.890936	0.635891
45	8	2.147832	2.213452	-1.330680
46	14	4.028937	-1.115153	-0.278521
47	6	-4.465719	-0.524946	-1.245929
48	6	-3.899357	1.107710	0.346349
49	6	-5.708651	-0.695078	-0.671683
50	6	-5.124566	0.966305	0.963492
51	6	-6.042635	0.050320	0.454300
52	1	-6.396371	-1.412298	-1.102882
53	1	-5.353473	1.578789	1.827085
54	1	-7.011046	-0.074025	0.927431
55	7	-3.587413	0.360098	-0.732956
56	1	-3.166832	1.829612	0.679604
57	1	-4.124809	-1.072264	-2.115193

Product:

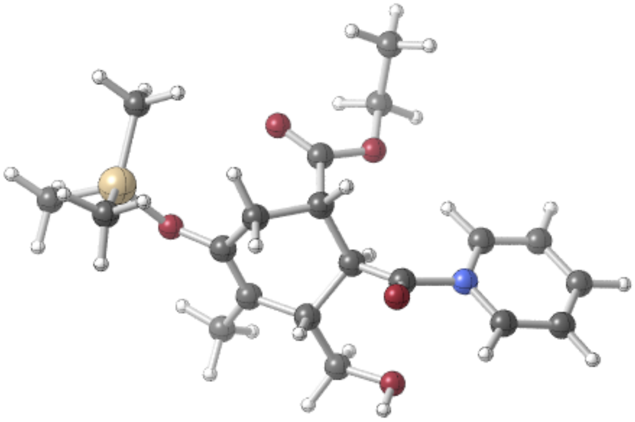

Charge = 1 Multiplicity = 1

HF = -1499.9126182 hartrees (-941210.167046682 kcal/mol)

Imaginary Frequencies: none found

Zero-point correction = 0.483794 (Hartree/Particle)

Temperature 298.150 Kelvin. Pressure 1.00000 Atm.

Sum of electronic and thermal Free Energies =

-1499.491126 hartrees (-940945.67647626 kcal/mol)

Coordinates (from last standard orientation):

Center Number	Atomic Number	Coordinates (Angstroms) X	Y	Z
1	6	-2.097784	-0.791058	-0.965127
2	6	0.069551	0.457140	-0.804857
3	6	-0.933817	-0.475265	-0.061188
4	6	-0.270366	-1.829049	0.350576
5	6	1.403230	-0.246925	-1.094685
6	6	1.107812	-1.591387	0.932565
7	6	1.934733	-0.842013	0.187022
8	6	-1.220392	-2.574038	1.285004
9	6	4.595222	1.675408	-0.689888
10	6	4.586505	-1.186188	-1.893077
11	6	5.989021	-0.621577	0.794253
12	6	1.519575	-2.213333	2.238005
13	6	0.342948	1.730327	-0.011825
14	6	-0.662566	3.538902	1.154235
15	6	-0.553098	4.694246	0.180276
16	1	-0.371898	0.791185	-1.752530

17	1	-1.279123	0.000671	0.858143
18	1	-0.183857	-2.424432	-0.572109
19	1	2.096081	0.483414	-1.514794
20	1	1.236122	-1.025217	-1.849414
21	1	-0.794109	-3.537401	1.584195
22	1	-1.402965	-1.989144	2.192130
23	1	5.532624	1.932407	-1.196043
24	1	3.764128	1.996967	-1.323412
25	1	4.541984	2.251492	0.239245
26	1	4.414580	-2.243760	-1.666253
27	1	3.835020	-0.867337	-2.621660
28	1	5.568268	-1.103128	-2.372323
29	1	6.946054	-0.370803	0.324954
30	1	5.929120	-0.084134	1.745906
31	1	5.986975	-1.694676	1.009142
32	1	-2.419129	-3.497596	0.050252
33	1	0.842615	-1.939322	3.055176
34	1	1.528885	-3.308537	2.180233
35	1	2.524090	-1.881882	2.505003
36	1	0.207719	3.476275	1.809584
37	1	-1.568232	3.600841	1.758889
38	1	-1.416841	4.715778	-0.489021
39	1	-0.519505	5.636715	0.733215
40	1	0.359234	4.610509	-0.414661
41	8	-2.029267	-1.368058	-2.007495
42	8	3.198698	-0.567892	0.591753
43	8	-2.490672	-2.753113	0.667704
44	8	-0.791013	2.283388	0.446116
45	8	1.436692	2.209664	0.155577
46	14	4.564689	-0.160038	-0.322678
47	6	-4.500789	-1.115185	-0.906706
48	6	-3.630967	0.808794	0.152237
49	6	-5.786239	-0.756071	-0.564224
50	6	-4.902914	1.215030	0.505206
51	6	-5.992378	0.422532	0.151815
52	1	-6.610862	-1.394248	-0.855060
53	1	-5.028602	2.144546	1.045820
54	1	-6.997102	0.722117	0.429148
55	7	-3.459983	-0.339369	-0.535499
56	1	-2.739604	1.386759	0.372033
57	1	-4.246660	-2.005603	-1.466750

DAL with acylammonium ion formed with 2,6-lutidine (endo):

Transition state 1 (TS1):

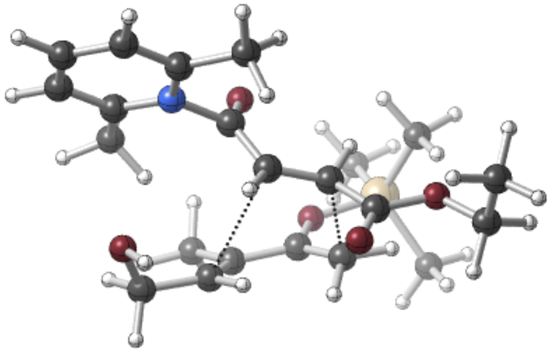

Charge = 1 Multiplicity = 1

HF = -1578.4602561 hartrees (-990499.595305311 kcal/mol)

Imaginary Frequencies: 1 (-360.8406 1/cm)

Zero-point correction = 0.534254 (Hartree/Particle)

Temperature   298.150 Kelvin. Pressure   1.00000 Atm.

Sum of electronic and thermal Free Energies =

-1577.991119 hartrees (-990205.20708369 kcal/mol)

Coordinates (from last standard orientation):

Center Number	Atomic Number	Coordinates (Angstroms) X	Y	Z
1	6	-1.340562	-0.008583	-0.808114
2	6	-0.777947	0.883603	0.140782
3	6	0.565724	1.215518	0.008893
4	6	-0.414922	-1.172889	2.190090
5	6	1.938228	0.116391	1.158855
6	6	-1.707856	-1.645867	2.792403
7	6	1.619185	-1.206201	0.878365
8	6	5.091061	-1.308650	0.239130
9	6	3.366477	-0.082417	-2.047956
10	6	3.880341	-3.122243	-1.952798
11	6	0.477178	-1.894967	1.469881
12	6	0.999893	2.492374	0.661044
13	6	2.628889	4.204424	0.646968
14	6	1.877015	5.402103	0.104729
15	6	0.334234	-3.353946	1.122283
16	1	-1.410972	1.382583	0.864337
17	1	-0.202023	-0.129213	2.405251
18	1	2.850258	0.532522	0.743325

19	1	1.648228	0.521521	2.120832
20	1	1.065340	0.962226	-0.921359
21	1	-1.936158	-2.681147	2.535052
22	1	-1.639429	-1.585201	3.887658
23	1	5.021394	-0.367380	0.793253
24	1	5.140293	-2.128883	0.963716
25	1	6.037474	-1.297462	-0.314551
26	1	2.427687	-0.198451	-2.601511
27	1	3.330753	0.862143	-1.494174
28	1	4.175148	-0.002981	-2.784744
29	1	4.052591	-3.987469	-1.303725
30	1	2.986329	-3.321364	-2.553334
31	1	4.732002	-3.040496	-2.637137
32	1	3.675853	4.214429	0.341375
33	1	2.571600	4.149963	1.736499
34	1	2.329017	6.321316	0.488894
35	1	1.924536	5.423972	-0.987664
36	1	0.829425	5.376650	0.415778
37	1	-2.722025	0.020205	2.701464
38	1	0.221339	-3.481188	0.040415
39	1	1.235641	-3.902094	1.414370
40	1	-0.518493	-3.815939	1.620807
41	8	-0.786304	-0.737682	-1.602369
42	8	2.256491	-1.912691	-0.045980
43	8	0.421771	3.008282	1.591410
44	8	2.108157	2.974602	0.095706
45	8	-2.799051	-0.866719	2.313624
46	14	3.677496	-1.565615	-0.954973
47	6	-3.512506	-1.167337	-0.585170
48	6	-3.509301	1.149677	-1.178269
49	6	-4.900077	-1.174331	-0.604617
50	6	-4.894631	1.145919	-1.208476
51	6	-5.596847	-0.014684	-0.906767
52	1	-5.415191	-2.102187	-0.384652
53	1	-5.409860	2.060026	-1.479768
54	1	-6.682005	-0.016334	-0.923834
55	7	-2.860903	-0.001723	-0.839439
56	6	-2.736705	2.381602	-1.551168
57	1	-2.370213	2.908498	-0.665535
58	1	-1.878897	2.145069	-2.185300
59	1	-3.402009	3.051814	-2.098234
60	6	-2.736365	-2.417155	-0.323119
61	1	-2.416618	-2.863376	-1.269977
62	1	-1.847612	-2.214515	0.270284
63	1	-3.373587	-3.126980	0.206943

Intermediate (INT):

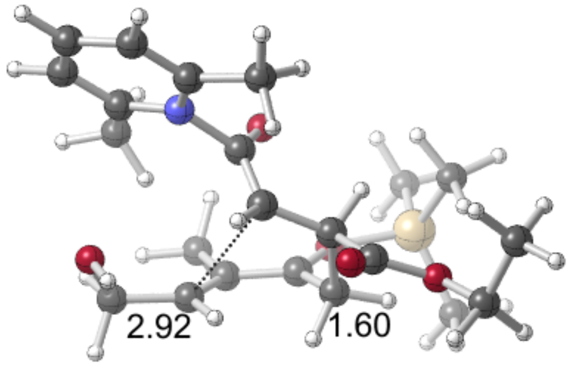

Charge = 1 Multiplicity = 1

HF = -1578.449279 hartrees (-990492.70706529 kcal/mol)

Imaginary Frequencies: none found

Zero-point correction = 0.536360 (Hartree/Particle)

Temperature 298.150 Kelvin. Pressure 1.00000 Atm.

Sum of electronic and thermal Free Energies =

-1577.979898 hartrees (-990198.16579398 kcal/mol)

Coordinates (from last standard orientation):

Center Number	Atomic Number	Coordinates (Angstroms) X	Y	Z
1	6	-1.256624	0.104618	-0.619897
2	6	-0.727411	0.878055	0.380122
3	6	0.745341	1.039813	0.345891
4	6	-0.627223	-1.449269	2.136504
5	6	1.644098	-0.005431	1.151657
6	6	-1.945472	-1.914215	2.691323
7	6	1.333808	-1.402127	0.777003
8	6	4.893267	-1.580381	0.211208
9	6	3.201658	-0.030692	-1.926046
10	6	3.575864	-3.092964	-2.163284
11	6	0.204105	-2.122262	1.295410
12	6	1.164154	2.430042	0.806082
13	6	3.008914	3.899685	1.024353
14	6	2.776435	4.911638	-0.080096
15	6	-0.010425	-3.513932	0.762526
16	1	-1.333494	1.519119	1.005408
17	1	-0.337642	-0.471276	2.502184

18	1	2.687949	0.219007	0.929168
19	1	1.493492	0.150233	2.220573
20	1	1.080712	0.937151	-0.692644
21	1	-2.245414	-2.886866	2.300203
22	1	-1.851377	-2.007833	3.782433
23	1	4.799256	-0.763245	0.932174
24	1	4.953934	-2.522980	0.763435
25	1	5.839033	-1.443787	-0.323969
26	1	2.219512	-0.040543	-2.410545
27	1	3.272920	0.853062	-1.283744
28	1	3.959368	0.066514	-2.711497
29	1	3.639103	-4.028482	-1.599719
30	1	2.687281	-3.132253	-2.800466
31	1	4.455877	-3.024586	-2.810382
32	1	4.071597	3.721039	1.191452
33	1	2.541506	4.210844	1.960872
34	1	3.238506	5.864605	0.191495
35	1	3.221131	4.565094	-1.016592
36	1	1.707105	5.075830	-0.231320
37	1	-2.792216	-0.154131	2.758281
38	1	-0.130225	-3.479954	-0.324747
39	1	0.860393	-4.139051	0.979626
40	1	-0.886921	-3.990606	1.201505
41	8	-0.693043	-0.686792	-1.389990
42	8	2.054311	-2.025202	-0.087771
43	8	0.415488	3.272876	1.240662
44	8	2.482474	2.606830	0.656303
45	8	-2.977789	-1.007981	2.338640
46	14	3.494889	-1.632666	-1.019405
47	6	-3.570465	-0.813083	-0.652030
48	6	-3.253534	1.497483	-1.141036
49	6	-4.946376	-0.652909	-0.750854
50	6	-4.625848	1.666162	-1.253321
51	6	-5.481282	0.593600	-1.036115
52	1	-5.581329	-1.519445	-0.609332
53	1	-5.009225	2.642565	-1.524382
54	1	-6.555314	0.725438	-1.113367
55	7	-2.768370	0.270921	-0.800183
56	6	-2.318841	2.634152	-1.450876
57	1	-1.927053	3.103299	-0.545443
58	1	-1.465744	2.288823	-2.039296
59	1	-2.867404	3.383169	-2.024248
60	6	-2.967003	-2.165271	-0.446242
61	1	-2.665608	-2.580022	-1.412748
62	1	-2.079387	-2.110700	0.176218
63	1	-3.704538	-2.823280	0.015943

Transition state 2 (TS2):

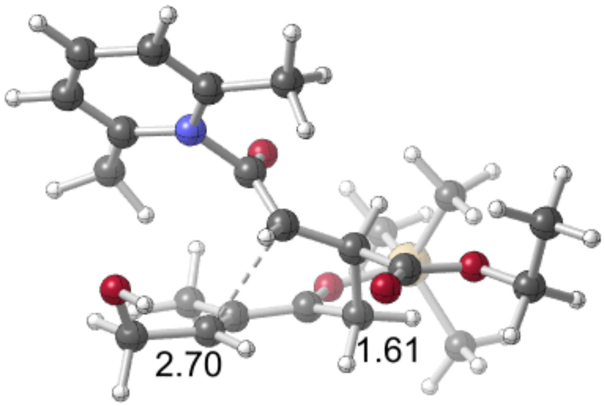

Charge = 1 Multiplicity = 1

HF = -1578.4733262 hartrees (-990507.796923762 kcal/mol)

Imaginary Frequencies: 1 (-66.1112 1/cm)

Zero-point correction = 0.536888 (Hartree/Particle)

Temperature   298.150 Kelvin. Pressure   1.00000 Atm.

Sum of electronic and thermal Free Energies =

-1577.999511 hartrees (-990210.47314761 kcal/mol)

Coordinates (from last standard orientation):

Center Number	Atomic Number	Coordinates (Angstroms) X	Y	Z
1	6	-1.243256	-0.041929	-0.610838
2	6	-0.748507	0.774677	0.384619
3	6	0.700211	1.076801	0.296622
4	6	-0.482166	-1.256824	2.138721
5	6	1.736688	0.137458	1.086778
6	6	-1.788480	-1.728066	2.719834
7	6	1.500351	-1.295300	0.826127
8	6	5.063084	-1.258347	0.185970
9	6	3.202325	-0.062620	-2.024171
10	6	3.830188	-3.093924	-1.984838
11	6	0.395101	-1.994176	1.384659
12	6	0.997026	2.482416	0.790732
13	6	2.579133	4.242260	0.720304
14	6	1.871130	5.308915	-0.089035
15	6	0.216711	-3.432214	0.972488
16	1	-1.394373	1.393000	0.993351
17	1	-0.182095	-0.283091	2.506822
18	1	2.740242	0.429762	0.774234

19	1	1.643021	0.348575	2.154439
20	1	1.021218	1.005937	-0.747346
21	1	-2.053782	-2.733233	2.391509
22	1	-1.675796	-1.755141	3.814191
23	1	5.157909	-2.086742	0.896124
24	1	5.996596	-1.202764	-0.386507
25	1	4.967967	-0.326891	0.753341
26	1	2.213424	-0.183204	-2.480246
27	1	3.218067	0.876324	-1.460295
28	1	3.933886	0.027332	-2.836272
29	1	4.034531	-3.957298	-1.342885
30	1	2.921638	-3.303163	-2.559343
31	1	4.660206	-2.996611	-2.693244
32	1	3.655818	4.254236	0.544178
33	1	2.386991	4.352642	1.790017
34	1	2.242458	6.295792	0.202838
35	1	2.060663	5.169706	-1.157065
36	1	0.793150	5.279265	0.089487
37	1	-2.692720	0.000518	2.691099
38	1	0.101144	-3.508325	-0.113629
39	1	1.103268	-4.012448	1.247343
40	1	-0.646973	-3.894321	1.452023
41	8	-0.634927	-0.802665	-1.368319
42	8	2.256992	-1.961322	0.008276
43	8	0.294663	3.114166	1.545681
44	8	2.163754	2.921612	0.308243
45	8	-2.866518	-0.888518	2.340781
46	14	3.636718	-1.544977	-0.979816
47	6	-3.501193	-1.088653	-0.620007
48	6	-3.305846	1.213423	-1.202609
49	6	-4.880412	-1.017208	-0.767552
50	6	-4.680313	1.292324	-1.366564
51	6	-5.477912	0.179334	-1.129541
52	1	-5.464100	-1.915214	-0.599808
53	1	-5.109240	2.232433	-1.693314
54	1	-6.554843	0.242332	-1.248385
55	7	-2.759140	0.032302	-0.797061
56	6	-2.435544	2.396062	-1.517963
57	1	-2.093384	2.903229	-0.612089
58	1	-1.554169	2.098703	-2.092020
59	1	-3.021717	3.101481	-2.109929
60	6	-2.832964	-2.391109	-0.321154
61	1	-2.607133	-2.905201	-1.261604
62	1	-1.897483	-2.253469	0.210915
63	1	-3.508303	-3.016561	0.266138

Product:

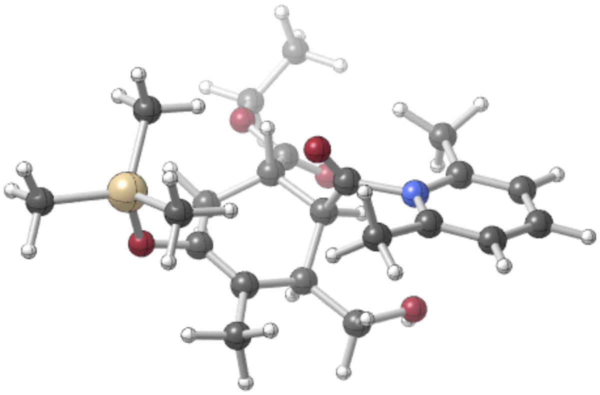

Charge = 1 Multiplicity = 1

HF = -1578.5331871 hartrees (-990545.360237121 kcal/mol)

Imaginary Frequencies: none found

Zero-point correction = 0.540279 (Hartree/Particle)

Temperature 298.150 Kelvin. Pressure 1.00000 Atm.

Sum of electronic and thermal Free Energies =

-1578.055823 hartrees (-990245.80949073 kcal/mol)

Coordinates (from last standard orientation):

Center Number	Atomic Number	Coordinates (Angstroms) X	Y	Z
1	6	-0.938848	-0.414758	-0.697654
2	6	-0.669988	0.494475	0.470708
3	6	0.398133	1.528537	0.073923
4	6	-0.252906	-0.281607	1.761801
5	6	1.762716	1.315764	0.781273
6	6	-1.342134	-1.234925	2.265678
7	6	2.053596	-0.137502	1.058816
8	6	5.660947	-1.425656	-0.481078
9	6	3.391433	0.058768	-1.989942
10	6	2.921227	-2.780654	-0.973385
11	6	1.107643	-0.927518	1.587632
12	6	-0.103831	2.935341	0.350235
13	6	0.419480	5.228067	0.093746
14	6	-0.549224	5.680575	-0.979192
15	6	1.379656	-2.325070	2.071808
16	1	-1.593079	1.006473	0.733156
17	1	-0.179339	0.528117	2.507177

18	1	2.560597	1.756682	0.180951
19	1	1.751613	1.852136	1.740760
20	1	0.579974	1.470864	-1.004241
21	1	-1.365795	-2.159319	1.682428
22	1	-1.114281	-1.521194	3.299857
23	1	5.891082	-2.130350	0.325431
24	1	6.082161	-1.828622	-1.408930
25	1	6.173457	-0.482728	-0.262135
26	1	2.309502	0.234412	-2.028994
27	1	3.895848	1.020933	-1.849644
28	1	3.693775	-0.334560	-2.968103
29	1	3.144910	-3.542070	-0.218737
30	1	1.835680	-2.626941	-0.999960
31	1	3.220338	-3.179554	-1.950559
32	1	1.369865	5.759205	0.025615
33	1	0.004278	5.358788	1.095377
34	1	-0.750404	6.749607	-0.862236
35	1	-0.124888	5.516484	-1.973795
36	1	-1.496939	5.141218	-0.900582
37	1	-2.700871	0.058700	2.799618
38	1	0.813414	-3.091615	1.529682
39	1	2.441939	-2.556567	1.969256
40	1	1.114001	-2.426585	3.131217
41	8	-0.178116	-0.721072	-1.565671
42	8	3.343704	-0.544553	0.856542
43	8	-1.146592	3.212191	0.901961
44	8	0.773145	3.839108	-0.085696
45	8	-2.641115	-0.676685	2.167204
46	14	3.820738	-1.170617	-0.645837
47	6	-2.413577	-2.379793	-0.720230
48	6	-3.394727	-0.192419	-0.834078
49	6	-3.676820	-2.941888	-0.653324
50	6	-4.659177	-0.757267	-0.796150
51	6	-4.805380	-2.130959	-0.671714
52	1	-3.759151	-4.020960	-0.599183
53	1	-5.518476	-0.100571	-0.870627
54	1	-5.795057	-2.572618	-0.618858
55	7	-2.313045	-1.017484	-0.749086
56	6	-3.224133	1.285489	-1.035003
57	1	-3.290521	1.827243	-0.086166
58	1	-2.279774	1.544064	-1.521230
59	1	-4.039937	1.632691	-1.672537
60	6	-1.187047	-3.238441	-0.794005
61	1	-0.814891	-3.264590	-1.822884
62	1	-0.374822	-2.879151	-0.158675
63	1	-1.453655	-4.251844	-0.491032

DAL with acylammonium ion formed with 2,6-lutidine (exo):

Transition state 1 (TS1):

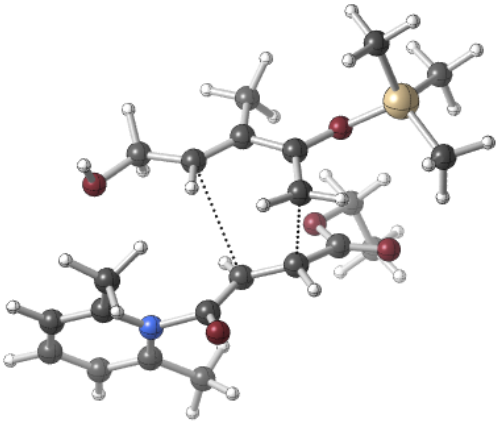

Charge = 1 Multiplicity = 1

HF = -1578.4594581 hartrees (-990499.094552331 kcal/mol)

Imaginary Frequencies: 1 (-364.3541 1/cm)

Zero-point correction = 0.533503 (Hartree/Particle)

Temperature   298.150 Kelvin. Pressure   1.00000 Atm.

Sum of electronic and thermal Free Energies =

-1577.990860 hartrees (-990205.0445586 kcal/mol)

Coordinates (from last standard orientation):

Center Number	Atomic Number	Coordinates (Angstroms) X	Y	Z
1	6	-2.172011	0.369589	-1.378111
2	6	0.166587	0.899399	-1.144820
3	6	-1.073327	0.703274	-0.542624
4	6	-0.388912	-1.847227	0.879864
5	6	1.245321	-0.879908	-1.333000
6	6	0.889986	-1.463311	1.086627
7	6	1.712762	-1.033063	-0.034482
8	6	-1.374475	-2.189176	1.964693
9	6	4.464429	0.080102	-2.160002
10	6	4.720565	-2.571986	-0.595946
11	6	5.583907	0.125976	0.705594
12	6	1.498869	-1.351984	2.459004
13	6	1.236109	1.681273	-0.450061
14	6	2.071965	2.392023	1.641778

15	6	1.863929	3.892094	1.602581
16	1	0.192842	1.009204	-2.225722
17	1	-1.224103	0.850219	0.518600
18	1	-0.767925	-1.955935	-0.131513
19	1	1.951852	-0.586075	-2.102716
20	1	0.387592	-1.445596	-1.676963
21	1	-0.966586	-2.970323	2.622765
22	1	-1.560035	-1.311636	2.595514
23	1	5.507704	0.300177	-2.418711
24	1	4.059957	-0.565310	-2.946875
25	1	3.911119	1.024788	-2.160827
26	1	4.697883	-3.042226	0.393166
27	1	3.954213	-3.050142	-1.217183
28	1	5.695009	-2.784597	-1.050117
29	1	6.626701	-0.035097	0.409165
30	1	5.406015	1.206851	0.716510
31	1	5.462196	-0.253329	1.725748
32	1	-2.531963	-3.451446	1.042605
33	1	1.757036	-0.311714	2.681710
34	1	0.817484	-1.711282	3.232093
35	1	2.425228	-1.932180	2.520682
36	1	3.055085	2.103427	1.261792
37	1	1.951180	2.002198	2.653718
38	1	0.859655	4.148863	1.952237
39	1	2.591789	4.377193	2.259783
40	1	1.999386	4.280144	0.590288
41	8	-2.202999	0.133429	-2.566483
42	8	2.910812	-0.561464	0.313699
43	8	-2.632975	-2.572426	1.442268
44	8	1.062551	1.705125	0.872141
45	8	2.161433	2.206346	-1.029187
46	14	4.430709	-0.731417	-0.477802
47	6	-4.140197	-0.905275	-0.618980
48	6	-4.013547	1.422559	-0.075679
49	6	-5.334079	-1.005456	0.081098
50	6	-5.215076	1.331853	0.607843
51	6	-5.871286	0.110761	0.704576
52	1	-5.832932	-1.966883	0.117955
53	1	-5.626549	2.230667	1.052184
54	1	-6.807292	0.035888	1.248579
55	7	-3.500719	0.294589	-0.643456
56	6	-3.303317	2.735592	-0.222785
57	1	-2.484948	2.828694	0.496993
58	1	-2.891170	2.864277	-1.226509
59	1	-4.019081	3.537102	-0.032303
60	6	-3.589541	-2.069908	-1.381958

61	1	-3.782216	-1.934651	-2.450090
62	1	-2.513885	-2.186548	-1.247147
63	1	-4.088687	-2.978285	-1.042765

Intermediate (INT):

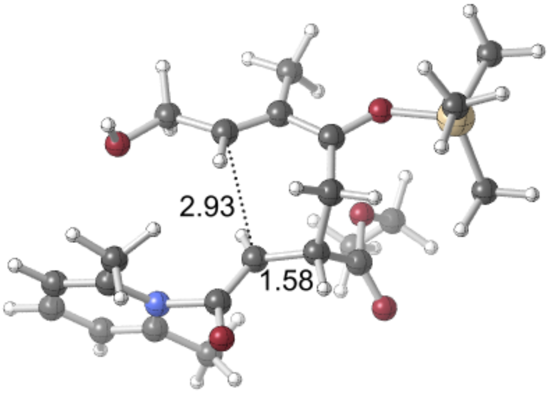

Charge = 1 Multiplicity = 1

HF = -1578.474036 hartrees (-990508.24233036 kcal/mol)

Imaginary Frequencies: none found

Zero-point correction = 0.536237 (Hartree/Particle)

Temperature 298.150 Kelvin. Pressure 1.00000 Atm.

Sum of electronic and thermal Free Energies =

-1578.003152 hartrees (-990212.75791152 kcal/mol)

Coordinates (from last standard orientation):

Center Number	Atomic Number	Coordinates (Angstroms) X	Y	Z
1	6	-2.083170	0.518586	-1.399358
2	6	0.376851	0.625848	-1.212131
3	6	-0.970674	0.432083	-0.597413
4	6	-0.271771	-2.114757	0.669989
5	6	1.322953	-0.641091	-1.288641
6	6	0.970442	-1.704677	1.029479
7	6	1.821162	-1.128027	0.024618
8	6	-1.307683	-2.630092	1.624462
9	6	4.322760	1.353463	-0.913462
10	6	4.698041	-1.575717	-1.956207
11	6	5.787205	-0.746698	0.822748

12	6	1.512651	-1.779622	2.431709
13	6	1.029330	1.796942	-0.506671
14	6	1.977225	2.477779	1.563648
15	6	0.811503	3.086346	2.315047
16	1	0.229224	0.926168	-2.252586
17	1	-1.079065	0.381548	0.479406
18	1	-0.566680	-2.104309	-0.373811
19	1	2.168701	-0.398576	-1.934778
20	1	0.744649	-1.425365	-1.783403
21	1	-0.915985	-3.508603	2.158654
22	1	-1.508857	-1.865614	2.384876
23	1	5.238303	1.679119	-1.423429
24	1	3.484784	1.567950	-1.584365
25	1	4.217289	1.964378	-0.011399
26	1	5.736444	-1.529939	-2.305485
27	1	4.489800	-2.615681	-1.681173
28	1	4.054866	-1.306943	-2.799636
29	1	6.779316	-0.452826	0.462851
30	1	5.569151	-0.162217	1.723047
31	1	5.827395	-1.804654	1.101990
32	1	-2.435086	-3.727859	0.482761
33	1	0.768278	-2.164868	3.130276
34	1	2.390228	-2.432295	2.473508
35	1	1.827067	-0.787121	2.772085
36	1	2.504550	3.225138	0.966950
37	1	2.683800	1.988166	2.237256
38	1	0.264355	2.313467	2.863676
39	1	1.177286	3.827579	3.031692
40	1	0.127992	3.585353	1.621873
41	8	-2.259007	0.666027	-2.609283
42	8	3.051948	-0.979530	0.360637
43	8	-2.537730	-2.907983	0.992358
44	8	1.510358	1.427749	0.693448
45	8	1.057892	2.930750	-0.922922
46	14	4.506228	-0.447698	-0.486709
47	6	-4.061150	-0.820668	-0.737338
48	6	-3.842520	1.365516	0.182762
49	6	-5.239793	-1.012255	-0.029002
50	6	-5.025873	1.194788	0.888298
51	6	-5.722080	-0.004062	0.793658
52	1	-5.766153	-1.953235	-0.139298
53	1	-5.390544	2.011376	1.500827
54	1	-6.644948	-0.145548	1.347309
55	7	-3.387712	0.344474	-0.584788
56	6	-3.072364	2.651486	0.232688
57	1	-2.189464	2.559115	0.871385

58	1	-2.735623	2.946460	-0.764688
59	1	-3.715577	3.434110	0.639071
60	6	-3.541936	-1.842048	-1.699787
61	1	-3.703981	-1.497771	-2.724934
62	1	-2.470097	-2.000379	-1.565479
63	1	-4.068975	-2.784521	-1.546058

Transition state 2 (TS2):

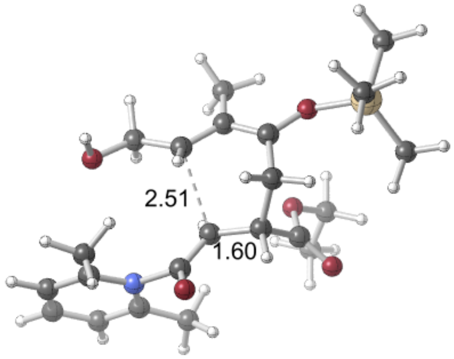

Charge = 1 Multiplicity = 1

HF = -1579.0760202 hartrees (-990885.993435702 kcal/mol)

Imaginary Frequencies: 1 (-127.2151 1/cm)

Zero-point correction = 0.530002 (Hartree/Particle)

Temperature 298.150 Kelvin. Pressure 1.00000 Atm.

Sum of electronic and thermal Free Energies =

-1578.611781 hartrees (-990594.67869531 kcal/mol)

Coordinates (from last standard orientation):

Center Number	Atomic Number	Coordinates (Angstroms) X	Y	Z
1	6	-2.095272	0.087568	-1.273617
2	6	0.330278	0.490182	-1.175388
3	6	-0.957876	0.219916	-0.455347
4	6	-0.306761	-1.903441	0.709123
5	6	1.387571	-0.715760	-1.230413
6	6	0.986815	-1.545647	1.097147
7	6	1.882385	-1.102005	0.113179
8	6	-1.336362	-2.286894	1.737541
9	6	4.764979	1.179528	-0.876019

10	6	4.761366	-1.808271	-1.799883
11	6	5.885414	-1.034907	0.985233
12	6	1.379633	-1.521243	2.557749
13	6	1.010787	1.759631	-0.678435
14	6	1.871275	2.888798	1.253532
15	6	0.858203	3.950368	1.643434
16	1	0.104391	0.671007	-2.227982
17	1	-1.080517	0.560612	0.561923
18	1	-0.496287	-2.243115	-0.300979
19	1	2.205308	-0.415800	-1.887306
20	1	0.858140	-1.540460	-1.715474
21	1	-0.869214	-2.970314	2.465434
22	1	-1.649776	-1.406286	2.310991
23	1	5.719309	1.346013	-1.393590
24	1	3.963068	1.479829	-1.559191
25	1	4.742201	1.849133	-0.008170
26	1	4.121475	-1.528916	-2.644394
27	1	5.796258	-1.828367	-2.166533
28	1	4.502074	-2.831003	-1.499688
29	1	6.909263	-0.893017	0.616217
30	1	5.756346	-0.392005	1.864116
31	1	5.791000	-2.077262	1.312429
32	1	-2.289793	-3.733313	0.843942
33	1	0.684891	-0.901980	3.138277
34	1	1.360421	-2.526569	2.997265
35	1	2.383414	-1.115042	2.687924
36	1	2.610287	3.278988	0.551410
37	1	2.382932	2.483223	2.128945
38	1	0.114380	3.548640	2.340290
39	1	1.375103	4.780119	2.139689
40	1	0.344366	4.347849	0.762482
41	8	-2.180461	-0.094971	-2.485261
42	8	3.133619	-0.863984	0.466070
43	8	-2.517279	-2.857129	1.199672
44	8	1.220400	1.732806	0.652040
45	8	1.334209	2.682424	-1.401619
46	14	4.646600	-0.616900	-0.353826
47	6	-4.289770	-0.909820	-0.614179
48	6	-3.821018	1.341459	0.065974
49	6	-5.517959	-0.849243	0.036131
50	6	-5.054798	1.406444	0.705096
51	6	-5.900480	0.302826	0.712969
52	1	-6.169536	-1.714257	-0.008386
53	1	-5.343029	2.336918	1.180997
54	1	-6.859720	0.349381	1.218988
55	7	-3.449363	0.168442	-0.535878

56	6	-2.938180	2.556935	0.001357
57	1	-2.151154	2.531354	0.761645
58	1	-2.459971	2.665968	-0.975505
59	1	-3.551698	3.441587	0.189471
60	6	-3.919909	-2.112131	-1.431268
61	1	-4.061142	-1.896988	-2.495392
62	1	-2.884150	-2.413939	-1.283177
63	1	-4.571345	-2.943395	-1.152675

Product:

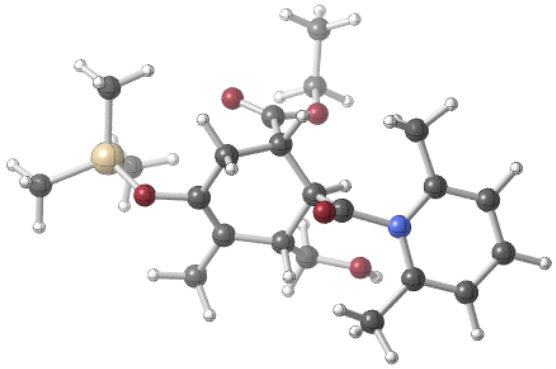

Charge = 1 Multiplicity = 1

HF = -1578.5370109 hartrees (-990547.759709859 kcal/mol)

Imaginary Frequencies: none found

Zero-point correction = 0.540052 (Hartree/Particle)

Temperature   298.150 Kelvin. Pressure   1.00000 Atm.

Sum of electronic and thermal Free Energies =

-1578.059553 hartrees (-990248.15010303 kcal/mol)

Coordinates (from last standard orientation):

Center Number	Atomic Number	Coordinates (Angstroms) X	Y	Z
1	6	-1.909183	-0.620549	-1.113813
2	6	0.212739	0.717072	-0.976351
3	6	-0.876490	-0.038167	-0.186595
4	6	-0.317620	-1.173144	0.742003
5	6	1.264739	-0.210914	-1.566204
6	6	1.159189	-1.477895	0.586514
7	6	1.851542	-1.080817	-0.489466
8	6	-0.713815	-0.838536	2.179125

9	6	4.365587	-0.018490	1.458382
10	6	4.819867	0.758479	-1.505150
11	6	5.891144	-1.914414	-0.447238
12	6	1.757884	-2.388572	1.625687
13	6	0.882504	1.763556	-0.089022
14	6	0.506622	3.532098	1.444341
15	6	0.741703	4.780185	0.620304
16	1	-0.269192	1.291412	-1.778399
17	1	-1.400350	0.665819	0.460518
18	1	-0.839025	-2.112406	0.509545
19	1	2.060842	0.383940	-2.021570
20	1	0.820624	-0.823318	-2.358998
21	1	-0.388614	-1.632400	2.860382
22	1	-0.237898	0.104352	2.489657
23	1	5.201433	0.648545	1.701151
24	1	3.438782	0.550919	1.587319
25	1	4.374500	-0.842056	2.180742
26	1	5.805404	1.207602	-1.331286
27	1	4.800059	0.398968	-2.540373
28	1	4.066438	1.543689	-1.392165
29	1	6.862130	-1.475501	-0.190322
30	1	5.718277	-2.762278	0.224912
31	1	5.961366	-2.303419	-1.469127
32	1	-2.418767	-0.519476	3.087519
33	1	1.087407	-3.231954	1.835195
34	1	2.713280	-2.790874	1.282380
35	1	1.928807	-1.869354	2.576812
36	1	1.422787	3.186475	1.928042
37	1	-0.263706	3.686681	2.200895
38	1	-0.180734	5.092610	0.122309
39	1	1.072699	5.592709	1.273864
40	1	1.513655	4.609485	-0.134496
41	8	-1.740494	-1.189443	-2.147303
42	8	3.130385	-1.503276	-0.718270
43	8	-2.130568	-0.726459	2.184501
44	8	-0.010075	2.462433	0.620530
45	8	2.073152	1.972382	-0.058532
46	14	4.529228	-0.643617	-0.298852
47	6	-4.018635	-1.508754	-0.134408
48	6	-3.884321	0.795144	-0.787336
49	6	-5.325863	-1.307123	0.286043
50	6	-5.187484	0.997091	-0.370487
51	6	-5.911291	-0.054602	0.180172
52	1	-5.868703	-2.151532	0.694636
53	1	-5.624147	1.980890	-0.495670
54	1	-6.933405	0.100097	0.510284

55	7	-3.337505	-0.446751	-0.641737
56	6	-3.073313	1.888091	-1.414211
57	1	-2.330483	2.288526	-0.714046
58	1	-2.556391	1.542564	-2.315280
59	1	-3.739735	2.704453	-1.695301
60	6	-3.365566	-2.847288	0.013097
61	1	-2.655236	-3.069462	-0.784315
62	1	-2.848123	-2.880507	0.977785
63	1	-4.139482	-3.617089	0.013761

DAL with acylammonium ion formed with DTBP (endo):

Transition state (TS1):

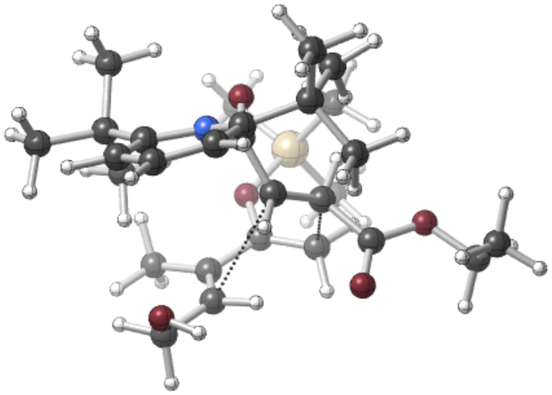

Charge = 1 Multiplicity = 1

HF = -1814.1921076 hartrees (-1138423.68944008 kcal/mol)

Imaginary Frequencies: 1 (-368.4471 1/cm)

Zero-point correction = 0.706519 (Hartree/Particle)

Temperature   298.150 Kelvin. Pressure   1.00000 Atm.

Sum of electronic and thermal Free Energies =

-1813.557470 hartrees (-1138025.4479997 kcal/mol)

Coordinates (from last standard orientation):

Center Number	Atomic Number	Coordinates (Angstroms) X	Y	Z
1	6	-0.857695	-0.221258	-0.789079
2	6	-0.414939	0.531396	0.330382
3	6	0.720405	1.330737	0.180978

4	6	1.035671	-0.972860	2.639015
5	6	2.586529	0.801098	0.981885
6	6	0.165827	-1.718231	3.607788
7	6	2.642461	-0.585243	0.878515
8	6	5.693064	0.123644	-0.745912
9	6	3.124338	0.608095	-2.451137
10	6	4.461973	-2.169543	-2.434904
11	6	1.994211	-1.482126	1.836365
12	6	0.724898	2.578880	1.010684
13	6	1.423092	4.834332	1.086139
14	6	0.120114	5.586715	0.911803
15	6	2.390198	-2.932628	1.765711
16	1	-1.058098	0.640429	1.194861
17	1	0.833350	0.096485	2.612115
18	1	3.193321	1.402021	0.312166
19	1	2.465488	1.208665	1.978552
20	1	1.133488	1.428545	-0.818462
21	1	0.227372	-2.799372	3.470934
22	1	0.490867	-1.496191	4.635245
23	1	5.490511	0.999770	-0.121665
24	1	6.226049	-0.613731	-0.136049
25	1	6.365258	0.437458	-1.553028
26	1	2.097496	0.244801	-2.575007
27	1	3.092530	1.604024	-1.996175
28	1	3.561744	0.718918	-3.450721
29	1	4.958865	-2.924855	-1.817055
30	1	3.526534	-2.597813	-2.810740
31	1	5.106121	-1.961753	-3.296635
32	1	2.255946	5.362084	0.620065
33	1	1.651289	4.655681	2.139349
34	1	0.213345	6.581341	1.357897
35	1	-0.113464	5.705828	-0.150245
36	1	-0.703403	5.063706	1.404738
37	1	-1.306510	-0.442568	3.681451
38	1	2.229067	-3.330123	0.760061
39	1	3.457402	-3.042989	1.987265
40	1	1.833823	-3.543100	2.477356
41	8	-0.267836	-0.547508	-1.792947
42	8	3.185994	-1.207013	-0.150245
43	8	0.192419	2.672014	2.096141
44	8	1.381717	3.562434	0.400231
45	8	-1.200211	-1.372989	3.424147
46	14	4.138004	-0.613909	-1.468329
47	6	-2.724479	-1.814235	-0.225267
48	6	-3.214945	0.514598	-0.639852
49	6	-3.957799	-1.947866	0.396878

50	6	-4.434140	0.363671	0.014947
51	6	-4.786832	-0.849558	0.572469
52	1	-4.273252	-2.911272	0.767279
53	1	-5.107341	1.207497	0.088374
54	1	-5.724366	-0.954720	1.108917
55	6	-1.851734	-3.050966	-0.505877
56	6	-1.723649	-3.229471	-2.031679
57	1	-1.136174	-4.132665	-2.228680
58	1	-2.712031	-3.364067	-2.484621
59	1	-1.229799	-2.383245	-2.508342
60	6	-0.473711	-3.001563	0.177170
61	1	-0.029116	-3.999334	0.103495
62	1	0.226396	-2.302638	-0.281702
63	1	-0.583414	-2.750287	1.236910
64	6	-2.546672	-4.313771	0.031392
65	1	-2.651277	-4.295976	1.121336
66	1	-3.530893	-4.470486	-0.420067
67	1	-1.925629	-5.174746	-0.230115
68	6	-2.957027	1.861971	-1.354922
69	6	-4.224853	2.158083	-2.195134
70	1	-4.394447	1.372326	-2.938549
71	1	-5.128834	2.268013	-1.593266
72	1	-4.072345	3.102419	-2.725710
73	6	-1.790028	1.915420	-2.354580
74	1	-1.789657	1.059140	-3.033976
75	1	-1.921616	2.820731	-2.954299
76	1	-0.813440	1.996071	-1.876705
77	6	-2.785958	2.972982	-0.304512
78	1	-2.675382	3.933937	-0.818006
79	1	-3.655559	3.039173	0.357253
80	1	-1.897700	2.804841	0.311962
81	7	-2.357308	-0.553797	-0.643624

Intermediate (INT):

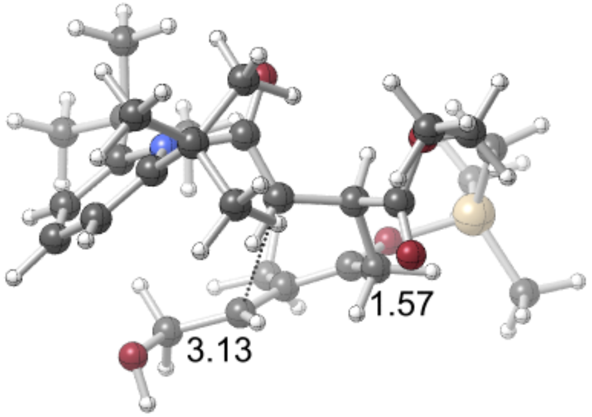

Charge = 1 Multiplicity = 1

HF = -1814.2122996 hartrees (-1138436.360122 kcal/mol)

Imaginary Frequencies: none found

Zero-point correction = 0.707579 (Hartree/Particle)

Temperature 298.150 Kelvin. Pressure 1.00000 Atm.

Sum of electronic and thermal Free Energies =

-1813.578165 hartrees (-1138038.43431915 kcal/mol)

Coordinates (from last standard orientation):

Center Number	Atomic Number	Coordinates (Angstroms) X	Y	Z
1	6	-1.023092	0.303636	-0.912540
2	6	-0.333042	0.493262	0.258629
3	6	1.064403	1.028194	0.202564
4	6	0.545524	-2.038835	1.877039
5	6	2.056172	0.380542	1.230807
6	6	-0.427483	-3.132572	2.193829
7	6	2.439420	-0.999038	0.836046
8	6	5.794068	0.230656	1.091461
9	6	4.156732	1.299261	-1.346663
10	6	5.695247	-1.376236	-1.571481
11	6	1.674135	-2.178619	1.135086
12	6	1.050083	2.511819	0.545555
13	6	1.089103	4.703668	-0.339516
14	6	-0.278666	5.264760	-0.015038
15	6	2.152087	-3.487044	0.564477
16	1	-0.818191	0.372720	1.219086
17	1	0.290596	-1.077626	2.310130

18	1	2.958898	0.995073	1.282558
19	1	1.594513	0.408348	2.218543
20	1	1.444123	0.920332	-0.817818
21	1	-0.838057	-3.532647	1.255753
22	1	0.096509	-3.965993	2.685121
23	1	5.242284	0.888524	1.770897
24	1	6.072114	-0.676910	1.637252
25	1	6.719444	0.747160	0.810247
26	1	3.433734	1.011273	-2.117114
27	1	3.693454	2.040723	-0.687870
28	1	4.994204	1.793308	-1.855456
29	1	5.987967	-2.284219	-1.034055
30	1	5.052504	-1.664234	-2.409942
31	1	6.602708	-0.922677	-1.985327
32	1	1.470996	5.101619	-1.280864
33	1	1.806424	4.916041	0.456955
34	1	-0.202482	6.349907	0.107261
35	1	-0.985232	5.058763	-0.823366
36	1	-0.661795	4.839402	0.916121
37	1	-1.224966	-2.572043	3.873633
38	1	2.299797	-3.410872	-0.517209
39	1	3.113315	-3.768277	1.007565
40	1	1.438016	-4.289526	0.759622
41	8	-0.738068	0.457917	-2.094875
42	8	3.481194	-1.189712	0.111998
43	8	1.033223	2.944006	1.677743
44	8	1.052119	3.275011	-0.550103
45	8	-1.519631	-2.671421	2.953985
46	14	4.831235	-0.182241	-0.446906
47	6	-2.599122	-1.659789	-0.587061
48	6	-3.251099	0.496553	0.230732
49	6	-3.429243	-2.260815	0.344684
50	6	-4.080133	-0.112815	1.175908
51	6	-4.115641	-1.488192	1.278372
52	1	-3.541318	-3.335556	0.360596
53	1	-4.681600	0.502184	1.833373
54	1	-4.713721	-1.968171	2.046414
55	6	-1.966211	-2.473627	-1.730972
56	6	-2.445974	-1.865198	-3.063746
57	1	-2.066918	-2.478040	-3.889097
58	1	-3.540768	-1.869036	-3.115567
59	1	-2.082275	-0.844688	-3.187372
60	6	-0.430753	-2.562001	-1.682767
61	1	-0.106532	-3.247971	-2.473417
62	1	0.062724	-1.605735	-1.848507
63	1	-0.107045	-2.982355	-0.727334

64	6	-2.479663	-3.921965	-1.686614
65	1	-2.137307	-4.459579	-0.795052
66	1	-3.571901	-3.975320	-1.733011
67	1	-2.083523	-4.448722	-2.559813
68	6	-3.342350	2.023690	0.056992
69	6	-4.844623	2.340191	-0.136959
70	1	-5.243280	1.825810	-1.017854
71	1	-5.452993	2.067534	0.728478
72	1	-4.956650	3.417858	-0.291576
73	6	-2.620571	2.596835	-1.170012
74	1	-2.837699	2.021715	-2.074832
75	1	-2.985606	3.618136	-1.319883
76	1	-1.537790	2.647036	-1.043463
77	6	-2.844773	2.737812	1.322824
78	1	-3.026496	3.813391	1.219528
79	1	-3.371807	2.393862	2.219257
80	1	-1.771072	2.580807	1.467632
81	7	-2.451787	-0.293024	-0.531909

Transition state 2 (TS2):

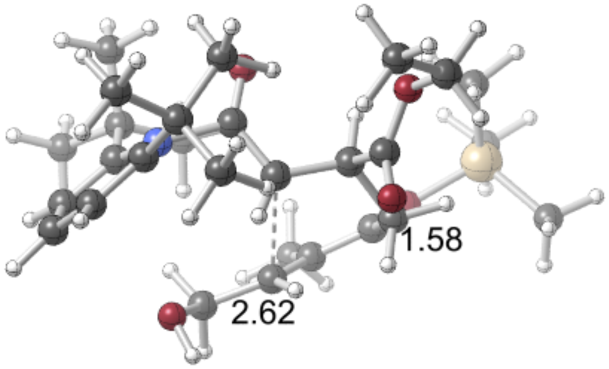

Charge = 1 Multiplicity = 1

HF = -1814.20984 hartrees (-1138434.8166984 kcal/mol)

Imaginary Frequencies: 1 (-113.5750 1/cm)

Zero-point correction = 0.707072 (Hartree/Particle)

Temperature 298.150 Kelvin. Pressure 1.00000 Atm.

Sum of electronic and thermal Free Energies =

-1813.573163 hartrees (-1138035.29551413 kcal/mol)

Coordinates (from last standard orientation):

Center Number	Atomic Number	Coordinates (Angstroms) X	Y	Z
1	6	0.977150	0.093936	0.775674
2	6	0.285954	0.430150	-0.386404
3	6	-1.092320	0.981398	-0.209111
4	6	-0.429960	-1.575084	-1.921669
5	6	-2.224714	0.438548	-1.175549
6	6	0.758811	-2.410690	-2.278790
7	6	-2.502817	-0.997594	-0.945974
8	6	-5.883457	0.236429	-0.956091
9	6	-4.216126	0.719675	1.649505
10	6	-5.815862	-1.903173	1.270414
11	6	-1.573944	-2.002736	-1.288464
12	6	-1.086978	2.478860	-0.473850
13	6	-1.444676	4.618377	0.469120
14	6	-0.072424	5.256425	0.497644
15	6	-1.841851	-3.420772	-0.861626
16	1	0.835742	0.614856	-1.300689
17	1	-0.438557	-0.617064	-2.423976
18	1	-3.126065	1.031093	-1.002186
19	1	-1.905391	0.631945	-2.202087
20	1	-1.405848	0.813288	0.824363
21	1	1.133624	-2.962964	-1.411215
22	1	0.440684	-3.164418	-3.018282
23	1	-6.839257	0.604412	-0.564668
24	1	-5.378910	1.078159	-1.439642
25	1	-6.107444	-0.514202	-1.722066
26	1	-3.510553	0.245771	2.341016
27	1	-3.714820	1.567820	1.171677
28	1	-5.042063	1.121179	2.249416
29	1	-6.133773	-2.659769	0.545440
30	1	-5.191657	-2.391908	2.025805
31	1	-6.711849	-1.519745	1.771102
32	1	-2.055576	4.945447	1.311769
33	1	-1.970250	4.835565	-0.463594
34	1	-0.175511	6.341206	0.394735
35	1	0.433144	5.046831	1.444345
36	1	0.543426	4.888868	-0.328025
37	1	1.597317	-1.310698	-3.647294
38	1	-1.967955	-3.496348	0.223102
39	1	-2.769038	-3.777505	-1.322651
40	1	-1.034219	-4.089173	-1.168190
41	8	0.612811	0.059179	1.942163

42	8	-3.557893	-1.378372	-0.282926
43	8	-0.881369	2.966916	-1.563818
44	8	-1.363751	3.183783	0.625399
45	8	1.827850	-1.626487	-2.758336
46	14	-4.900398	-0.519225	0.434882
47	6	2.763542	-1.669716	0.466954
48	6	3.211521	0.597681	-0.195414
49	6	3.776189	-2.101134	-0.375234
50	6	4.201907	0.147077	-1.066654
51	6	4.441572	-1.204431	-1.203760
52	1	4.044938	-3.147042	-0.404254
53	1	4.785230	0.867529	-1.626345
54	1	5.185725	-1.563541	-1.907330
55	6	2.157365	-2.622956	1.518053
56	6	2.505869	-2.053436	2.909878
57	1	2.171699	-2.764231	3.673678
58	1	3.590726	-1.934889	3.011204
59	1	2.022883	-1.093230	3.086465
60	6	0.648812	-2.895171	1.379431
61	1	0.450897	-3.464959	0.468450
62	1	0.343817	-3.521056	2.225570
63	1	0.024884	-2.004079	1.390579
64	6	2.837816	-3.999889	1.431448
65	1	2.665858	-4.493186	0.468939
66	1	3.915736	-3.939988	1.611054
67	1	2.407259	-4.637290	2.209268
68	6	3.139987	2.110572	0.100677
69	6	4.586224	2.524839	0.472573
70	1	4.944837	1.959144	1.338989
71	1	5.293806	2.388965	-0.348207
72	1	4.585676	3.587160	0.734916
73	6	2.270434	2.521341	1.297409
74	1	2.472631	1.904150	2.178059
75	1	2.522524	3.557348	1.544840
76	1	1.199710	2.488405	1.089836
77	6	2.707902	2.896236	-1.146760
78	1	2.839344	3.966541	-0.954053
79	1	3.314800	2.637066	-2.020676
80	1	1.655037	2.719682	-1.386848
81	7	2.435088	-0.330674	0.431268

Product:

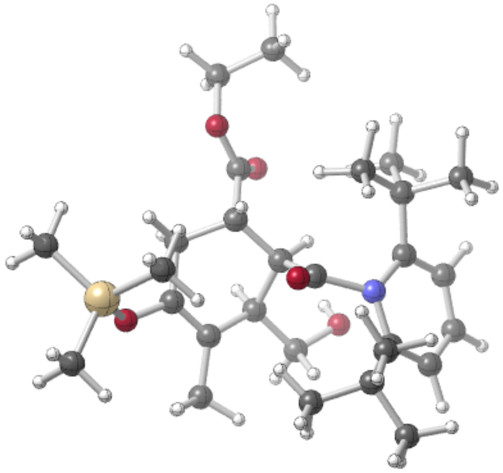

Charge = 1 Multiplicity = 1

HF = -1814.2627014 hartrees (-1138467.98775551 kcal/mol)

Imaginary Frequencies: none found

Zero-point correction = 0.712085 (Hartree/Particle)

Temperature 298.150 Kelvin. Pressure 1.00000 Atm.

Sum of electronic and thermal Free Energies =

-1813.619684 hartrees (-1138064.48790684 kcal/mol)

Coordinates (from last standard orientation):

Center Number	Atomic Number	Coordinates (Angstroms) X	Y	Z
1	6	0.700098	-0.303778	0.482857
2	6	0.188192	0.511558	-0.694579
3	6	-0.967020	1.422057	-0.210355
4	6	-0.280780	-0.354445	-1.914133
5	6	-2.355271	1.083737	-0.810589
6	6	0.824775	-1.238901	-2.485369
7	6	-2.544102	-0.389982	-1.045042
8	6	-5.880060	0.541596	0.569448
9	6	-3.408917	-0.612929	2.044360
10	6	-5.504606	-2.495956	0.770741
11	6	-1.565561	-1.105389	-1.615991
12	6	-0.680957	2.867758	-0.565737
13	6	-1.133792	5.111827	0.021291
14	6	0.172787	5.568032	0.636705
15	6	-1.801193	-2.519191	-2.080663

16	1	1.013880	1.122542	-1.068114
17	1	-0.482865	0.416724	-2.676836
18	1	-3.128879	1.487599	-0.152356
19	1	-2.464730	1.595385	-1.778173
20	1	-1.067218	1.346877	0.874604
21	1	1.047221	-2.074192	-1.815027
22	1	0.483388	-1.668156	-3.436550
23	1	-6.486307	0.558766	1.483079
24	1	-5.408751	1.525123	0.466966
25	1	-6.562433	0.406292	-0.277207
26	1	-2.626829	-1.379880	2.018993
27	1	-2.919671	0.367171	2.009325
28	1	-3.922946	-0.691715	3.009708
29	1	-6.155999	-2.664997	-0.093672
30	1	-4.785035	-3.320800	0.817381
31	1	-6.126600	-2.541384	1.672026
32	1	-1.992145	5.586028	0.498853
33	1	-1.165767	5.302607	-1.053483
34	1	0.289809	6.646967	0.498282
35	1	0.186757	5.352872	1.709168
36	1	1.019323	5.065950	0.159066
37	1	1.895839	0.169125	-3.297747
38	1	-2.534441	-3.013178	-1.437347
39	1	-2.204469	-2.530028	-3.101917
40	1	-0.893207	-3.124016	-2.088144
41	8	0.039613	-0.659431	1.406321
42	8	-3.776962	-0.920625	-0.818115
43	8	-0.000467	3.221978	-1.504163
44	8	-1.326650	3.698348	0.250946
45	8	2.042731	-0.538819	-2.647632
46	14	-4.632168	-0.850506	0.646579
47	6	2.495729	-2.035118	0.218600
48	6	3.093882	0.305389	0.187116
49	6	3.648438	-2.312232	-0.496651
50	6	4.250028	-0.001781	-0.516476
51	6	4.483832	-1.294416	-0.940772
52	1	3.914361	-3.338645	-0.700424
53	1	4.972847	0.778055	-0.718386
54	1	5.356593	-1.530032	-1.541006
55	6	1.733263	-3.173959	0.930621
56	6	1.737723	-2.863391	2.443805
57	1	1.306966	-3.719621	2.972893
58	1	2.766852	-2.733106	2.796951
59	1	1.164913	-1.975163	2.701589
60	6	0.325828	-3.473553	0.385408
61	1	0.406686	-3.945988	-0.597959

62	1	-0.151823	-4.192892	1.058859
63	1	-0.338431	-2.614598	0.300456
64	6	2.514223	-4.493008	0.777247
65	1	2.566347	-4.830102	-0.262368
66	1	3.527372	-4.423323	1.184948
67	1	1.978760	-5.261125	1.342799
68	6	3.018059	1.687376	0.866577
69	6	4.265992	1.729071	1.786580
70	1	4.243855	0.911764	2.515046
71	1	5.201617	1.673050	1.225907
72	1	4.257302	2.677347	2.332740
73	6	1.818975	1.929066	1.788582
74	1	1.684532	1.124156	2.517823
75	1	2.015987	2.848435	2.348020
76	1	0.881769	2.095977	1.256884
77	6	3.119611	2.825077	-0.160103
78	1	3.195764	3.773875	0.381383
79	1	4.009108	2.732372	-0.789973
80	1	2.238372	2.873692	-0.806385
81	7	2.171645	-0.693915	0.392497

DAL with acylammonium ion formed with DTBP (exo):

Transition state (TS1):

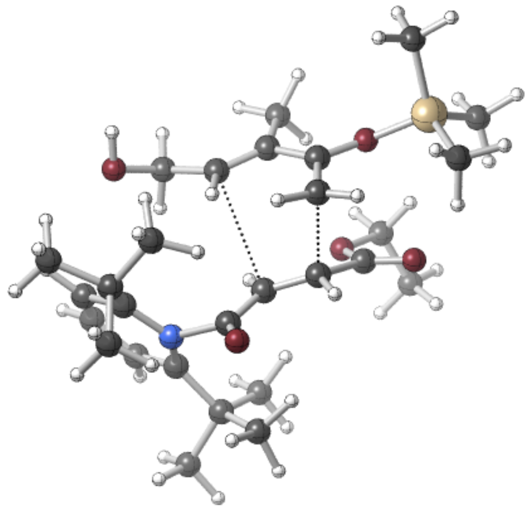

Charge = 1 Multiplicity = 1

HF = -1814.1978087 hartrees (-1138427.26693734 kcal/mol)

Imaginary Frequencies: 1 (-301.8672 1/cm)

Zero-point correction = 0.705979 (Hartree/Particle)

Temperature   298.150 Kelvin. Pressure   1.00000 Atm.

Sum of electronic and thermal Free Energies =

-1813.562416 hartrees (-1138028.55166416 kcal/mol)

Coordinates (from last standard orientation):

Center Number	Atomic Number	Coordinates (Angstroms) X	Y	Z
1	6	-1.662203	0.264167	-1.265148
2	6	0.713384	0.701079	-1.144624
3	6	-0.505990	0.576334	-0.492424
4	6	0.237808	-1.807820	1.211232
5	6	1.757791	-1.146763	-1.181398
6	6	1.539611	-1.460510	1.311812
7	6	2.295796	-1.149235	0.096215
8	6	-0.730067	-2.051982	2.332577
9	6	4.813549	-0.402863	-2.376269
10	6	5.637803	-2.444477	-0.226708
11	6	6.015456	0.607143	0.259016
12	6	2.271265	-1.268731	2.613128
13	6	1.828419	1.493279	-0.538261
14	6	2.704826	2.386518	1.466904
15	6	2.617112	3.879789	1.226683
16	1	0.716465	0.715179	-2.231716
17	1	-0.598523	0.753580	0.570966
18	1	-0.207093	-1.936307	0.230460
19	1	2.412306	-0.948162	-2.021895
20	1	0.872368	-1.729179	-1.403164
21	1	-0.222729	-2.325940	3.264726
22	1	-1.295698	-1.131867	2.531633
23	1	5.816207	-0.205917	-2.777033
24	1	4.407221	-1.258513	-2.925174
25	1	4.195396	0.478576	-2.573111
26	1	5.791898	-2.625765	0.842420
27	1	4.937608	-3.199520	-0.602679
28	1	6.596019	-2.597177	-0.736463
29	1	7.059328	0.524800	-0.065775
30	1	5.657433	1.601932	-0.027829
31	1	5.997058	0.531359	1.351405
32	1	-1.247793	-3.884659	1.923133
33	1	2.537538	-0.215116	2.749093
34	1	1.670772	-1.580568	3.468387
35	1	3.206804	-1.836417	2.618443

36	1	3.665804	1.973794	1.150893
37	1	2.538361	2.144707	2.518061
38	1	1.640062	4.263232	1.535108
39	1	3.386514	4.386294	1.817242
40	1	2.779161	4.117740	0.172849
41	8	-1.764821	0.059944	-2.449834
42	8	3.505420	-0.643853	0.327218
43	8	-1.700013	-3.026580	1.978740
44	8	1.656518	1.677302	0.771399
45	8	2.783910	1.890742	-1.168164
46	14	4.989331	-0.722751	-0.544534
47	6	-3.383404	-1.029867	0.008898
48	6	-3.184348	1.357668	0.334504
49	6	-3.997415	-1.130438	1.248977
50	6	-3.797840	1.234608	1.577762
51	6	-4.146327	-0.012873	2.061182
52	1	-4.347206	-2.091196	1.595050
53	1	-3.992748	2.122447	2.165797
54	1	-4.582219	-0.114899	3.049902
55	7	-2.897486	0.211321	-0.352856
56	6	-2.941069	2.776090	-0.222309
57	6	-3.409723	-2.208493	-0.985190
58	6	-1.905312	3.514992	0.640650
59	1	-1.844836	4.555808	0.305663
60	1	-2.185561	3.515795	1.698781
61	1	-0.911595	3.066588	0.541985
62	6	-2.536177	2.873944	-1.701695
63	1	-3.167439	2.251477	-2.342271
64	1	-2.672989	3.915402	-2.007559
65	1	-1.488983	2.624951	-1.880575
66	6	-4.301943	3.506954	-0.111417
67	1	-5.071061	2.992992	-0.697698
68	1	-4.656158	3.600622	0.917341
69	1	-4.185239	4.517842	-0.513087
70	6	-4.198631	-1.733723	-2.225018
71	1	-4.326014	-2.582738	-2.904733
72	1	-5.194706	-1.385278	-1.929704
73	1	-3.688218	-0.932076	-2.758710
74	6	-2.042094	-2.799235	-1.376590
75	1	-2.217283	-3.565590	-2.139143
76	1	-1.338130	-2.083626	-1.799865
77	1	-1.589409	-3.285528	-0.509245
78	6	-4.202845	-3.377423	-0.377859
79	1	-3.716642	-3.774603	0.517967
80	1	-5.232833	-3.096917	-0.136114
81	1	-4.244157	-4.177529	-1.122949

Intermediate (INT):

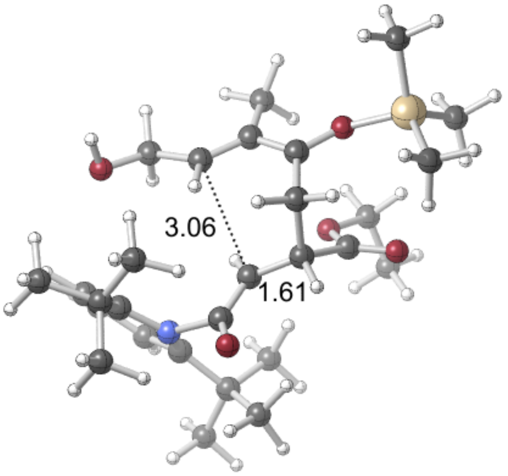

Charge = 1 Multiplicity = 1

HF = -1814.2119509 hartrees (-1138436.14130926 kcal/mol)

Imaginary Frequencies: none found

Zero-point correction = 0.707358 (Hartree/Particle)

Temperature 298.150 Kelvin. Pressure 1.00000 Atm.

Sum of electronic and thermal Free Energies =

-1813.576788 hartrees (-1138037.57023788 kcal/mol)

Coordinates (from last standard orientation):

Center Number	Atomic Number	Coordinates (Angstroms) X	Y	Z
1	6	-1.602800	0.264095	-1.308079
2	6	0.848587	0.383892	-1.145279
3	6	-0.489577	0.341040	-0.504675
4	6	0.299079	-2.072995	1.199042
5	6	1.640057	-1.015889	-1.167039
6	6	1.570062	-1.618689	1.338597
7	6	2.279599	-1.223326	0.149246
8	6	-0.636525	-2.435231	2.311573
9	6	4.728327	-0.376354	-2.414104
10	6	5.875880	-2.234959	-0.219508
11	6	5.773953	0.847253	0.209652
12	6	2.276315	-1.428155	2.653304
13	6	1.803168	1.400501	-0.539743
14	6	2.525844	2.408925	1.482633

15	6	2.120609	3.859901	1.324368
16	1	0.741737	0.633432	-2.204548
17	1	-0.585635	0.437486	0.567604
18	1	-0.127787	-2.193706	0.210143
19	1	2.398181	-0.971379	-1.947126
20	1	0.915680	-1.788767	-1.421249
21	1	-0.107016	-2.943753	3.126742
22	1	-1.050801	-1.507109	2.729311
23	1	5.707733	-0.121276	-2.840601
24	1	4.369930	-1.270342	-2.933481
25	1	4.053028	0.461414	-2.614693
26	1	6.025419	-2.368021	0.856960
27	1	5.311343	-3.094578	-0.596982
28	1	6.859830	-2.244094	-0.702163
29	1	6.839668	0.873978	-0.047572
30	1	5.319269	1.773566	-0.154368
31	1	5.695889	0.812554	1.301025
32	1	-1.424964	-4.106765	1.702928
33	1	2.568106	-0.378959	2.771822
34	1	1.639815	-1.701542	3.495747
35	1	3.190721	-2.027820	2.698383
36	1	3.538368	2.230011	1.116020
37	1	2.460715	2.087541	2.523540
38	1	1.124645	4.032989	1.741638
39	1	2.832628	4.495086	1.860077
40	1	2.122763	4.151660	0.270781
41	8	-1.761175	0.175190	-2.520934
42	8	3.499262	-0.855295	0.322784
43	8	-1.734444	-3.198496	1.853341
44	8	1.619270	1.532866	0.779061
45	8	2.687636	1.956087	-1.154515
46	14	4.998270	-0.636990	-0.592107
47	6	-3.415853	-0.940678	-0.019337
48	6	-3.060823	1.400852	0.378988
49	6	-3.988213	-1.062780	1.239176
50	6	-3.631492	1.270365	1.645683
51	6	-4.039840	0.030205	2.097355
52	1	-4.379105	-2.015443	1.563595
53	1	-3.738940	2.143882	2.275928
54	1	-4.443484	-0.082879	3.098688
55	7	-2.872856	0.274569	-0.360528
56	6	-2.733230	2.819325	-0.128598
57	6	-3.529747	-2.073569	-1.056988
58	6	-1.573681	3.415541	0.683741
59	1	-1.417195	4.456295	0.378297
60	1	-1.782376	3.405459	1.759258

61	1	-0.650501	2.858301	0.500640
62	6	-2.411297	2.936087	-1.625260
63	1	-3.117323	2.373728	-2.242490
64	1	-2.482696	3.993559	-1.898483
65	1	-1.400884	2.603512	-1.865933
66	6	-4.003197	3.669935	0.110245
67	1	-4.864935	3.248650	-0.418415
68	1	-4.258918	3.768428	1.167718
69	1	-3.825443	4.677321	-0.278275
70	6	-4.307289	-1.505205	-2.261461
71	1	-4.492142	-2.313465	-2.977564
72	1	-5.278081	-1.111663	-1.939129
73	1	-3.746362	-0.712900	-2.759368
74	6	-2.200384	-2.702990	-1.512482
75	1	-2.436669	-3.488582	-2.238974
76	1	-1.527219	-1.999419	-2.000704
77	1	-1.703754	-3.178190	-0.662708
78	6	-4.366864	-3.224944	-0.477764
79	1	-3.873073	-3.696409	0.378283
80	1	-5.367742	-2.897937	-0.177752
81	1	-4.486599	-3.983715	-1.256963

Transition state 2 (TS2):

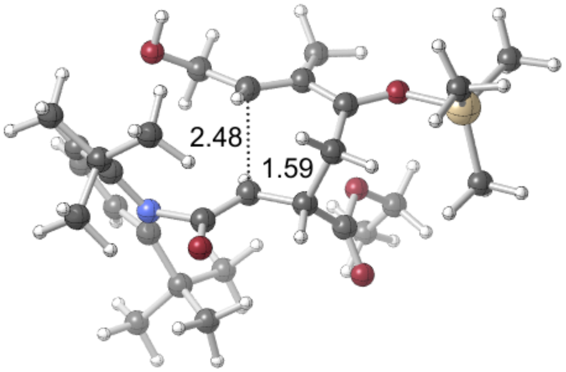

Charge = 1 Multiplicity = 1

HF = -1814.211054 hartrees (-1138435.57849554 kcal/mol)

Imaginary Frequencies: 1 (-212.4157 1/cm)

Zero-point correction = 0.707770 (Hartree/Particle)

Temperature 298.150 Kelvin. Pressure 1.00000 Atm.

Sum of electronic and thermal Free Energies =

-1813.572262 hartrees (-1138034.73012762 kcal/mol)

Coordinates (from last standard orientation):

Center Number	Atomic Number	X	Y	Z
1	6	-1.536014	0.075168	-1.188308
2	6	0.907012	0.271283	-1.183231
3	6	-0.372974	0.207580	-0.417792
4	6	0.147991	-1.683711	1.097819
5	6	1.844901	-1.009640	-1.061311
6	6	1.452315	-1.327306	1.392790
7	6	2.350729	-1.173860	0.321052
8	6	-0.920000	-1.746677	2.156432
9	6	5.045246	-2.407321	-1.513933
10	6	6.384941	-0.995392	0.889289
11	6	5.062216	0.706404	-1.312324
12	6	1.877228	-0.923066	2.783306
13	6	1.670624	1.538860	-0.846372
14	6	2.912488	2.650960	0.848875
15	6	2.034982	3.766881	1.374509
16	1	0.666144	0.335032	-2.246822
17	1	-0.084993	-2.198085	0.174091
18	1	2.660833	-0.910635	-1.779001
19	1	1.230194	-1.859641	-1.369578
20	1	-0.465177	-1.878261	3.146295
21	1	-1.465802	-0.793763	2.185549
22	1	6.052262	-2.541409	-1.926529
23	1	4.792765	-3.316181	-0.956560
24	1	4.353463	-2.318345	-2.357107
25	1	6.285927	-0.180699	1.614941
26	1	6.357043	-1.943738	1.436086
27	1	7.370479	-0.905698	0.418737
28	1	5.947483	0.732342	-1.960038
29	1	4.186656	0.865633	-1.950762
30	1	5.143981	1.548337	-0.617267
31	1	-1.449255	-3.614733	2.044411
32	1	1.293764	-0.058001	3.119680
33	1	1.727136	-1.729352	3.508243
34	1	2.931233	-0.645182	2.798260
35	1	3.523288	2.992560	0.010731
36	1	3.561518	2.246632	1.628640
37	1	1.392401	3.410193	2.185297
38	1	2.666685	4.571013	1.764319
39	1	1.410785	4.175300	0.575313
40	8	-1.676796	-0.131068	-2.382430
41	8	3.614580	-0.988280	0.596049

42	8	-1.879401	-2.754453	1.905847
43	8	2.123466	1.522360	0.415576
44	8	1.841128	2.459289	-1.612412
45	14	5.041410	-0.914617	-0.392129
46	6	-3.447407	-0.987265	0.036058
47	6	-3.005816	1.367839	0.332134
48	6	-4.185617	-0.978662	1.213488
49	6	-3.729571	1.350755	1.521209
50	6	-4.275036	0.169110	1.988173
51	1	-4.675005	-1.881171	1.545620
52	1	-3.867393	2.271133	2.074132
53	1	-4.811245	0.148165	2.931615
54	7	-2.793000	0.176761	-0.302112
55	6	-2.568799	2.741295	-0.226416
56	6	-3.520570	-2.196677	-0.922426
57	6	-1.519654	3.375216	0.699807
58	1	-1.306454	4.392625	0.354480
59	1	-1.868769	3.434281	1.736089
60	1	-0.585016	2.807408	0.677855
61	6	-2.045278	2.770518	-1.670751
62	1	-2.689178	2.210004	-2.353587
63	1	-2.038870	3.816529	-1.991905
64	1	-1.022468	2.400987	-1.765337
65	6	-3.841242	3.624223	-0.222734
66	1	-4.618978	3.191954	-0.860954
67	1	-4.256415	3.775905	0.775768
68	1	-3.579570	4.608371	-0.622558
69	6	-4.257208	-1.705446	-2.188255
70	1	-4.434088	-2.562244	-2.847372
71	1	-5.231138	-1.279256	-1.922117
72	1	-3.676351	-0.957794	-2.728437
73	6	-2.193273	-2.887815	-1.290211
74	1	-2.428799	-3.679018	-2.010342
75	1	-1.458646	-2.237028	-1.760123
76	1	-1.759991	-3.358578	-0.405506
77	6	-4.393147	-3.299654	-0.299756
78	1	-3.961456	-3.680091	0.631075
79	1	-5.417702	-2.964143	-0.111754
80	1	-4.446856	-4.128197	-1.012041
81	1	-0.441391	0.629744	0.575301

Product:

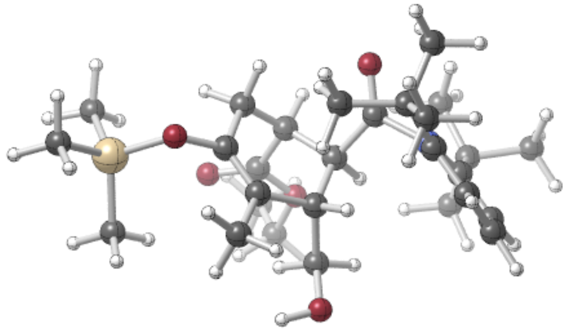

Charge = 1 Multiplicity = 1

HF = -1814.2760903 hartrees (-1138476.38942415 kcal/mol)

Imaginary Frequencies: none found

Zero-point correction = 0.713304 (Hartree/Particle)

Temperature   298.150 Kelvin. Pressure   1.00000 Atm.

Sum of electronic and thermal Free Energies =

-1813.631181 hartrees (-1138071.70238931 kcal/mol)

Coordinates (from last standard orientation):

Center Number	Atomic Number	Coordinates (Angstroms) X	Y	Z
1	6	-1.435818	-0.217896	-1.123592
2	6	0.735170	1.023805	-1.188227
3	6	-0.425885	0.554409	-0.298719
4	6	0.045155	-0.158236	0.998435
5	6	1.685563	-0.113404	-1.524719
6	6	1.341645	-0.928091	0.833709
7	6	2.067410	-0.886577	-0.292944
8	6	0.100479	0.855389	2.169041
9	6	5.271834	0.094901	-1.578383
10	6	5.827123	-2.582100	-0.162951
11	6	4.824215	-0.127774	1.447064
12	6	1.767865	-1.792843	1.993482
13	6	1.486432	2.161143	-0.505081
14	6	1.226429	4.208648	0.666716
15	6	1.610961	5.279281	-0.331826
16	1	0.322688	1.452280	-2.110878
17	1	-0.727360	-0.874994	1.313473
18	1	2.586112	0.293257	-1.991827

19	1	1.224586	-0.788019	-2.257174
20	1	0.999733	1.483082	2.097567
21	1	-0.775322	1.513104	2.107923
22	1	6.339883	0.330164	-1.491681
23	1	5.114868	-0.368277	-2.559024
24	1	4.718822	1.039496	-1.545373
25	1	5.506253	-3.273491	0.624036
26	1	5.771056	-3.112124	-1.120263
27	1	6.877865	-2.330041	0.019526
28	1	5.796277	0.366493	1.562784
29	1	4.052653	0.650337	1.484045
30	1	4.685581	-0.799008	2.300980
31	1	0.909402	-0.063207	3.688678
32	1	2.348395	-1.236472	2.741797
33	1	0.896230	-2.216461	2.504525
34	1	2.402484	-2.613127	1.648001
35	1	2.086937	3.868686	1.247565
36	1	0.445349	4.552161	1.347383
37	1	0.741170	5.586283	-0.919495
38	1	1.995629	6.154582	0.199799
39	1	2.388832	4.917376	-1.009454
40	8	-1.360897	-0.499464	-2.275206
41	8	3.198453	-1.644301	-0.434659
42	8	0.021209	0.222504	3.429174
43	8	0.641879	3.070596	-0.004331
44	8	2.690169	2.250291	-0.436879
45	14	4.764511	-1.046648	-0.183100
46	6	-2.800624	-1.875693	0.155200
47	6	-3.376883	0.479904	0.220305
48	6	-3.522146	-2.058959	1.324936
49	6	-4.066067	0.270268	1.407950
50	6	-4.093004	-0.982418	1.991012
51	1	-3.629583	-3.050326	1.737801
52	1	-4.596301	1.096128	1.865048
53	1	-4.601419	-1.136339	2.937247
54	7	-2.667065	-0.574666	-0.303652
55	6	-3.576818	1.838078	-0.490086
56	6	-2.247642	-3.087099	-0.621015
57	6	-3.099656	3.001292	0.397126
58	1	-3.460507	3.937719	-0.039770
59	1	-3.488521	2.934226	1.417087
60	1	-2.006975	3.061945	0.443342
61	6	-2.969145	1.995503	-1.890905
62	1	-3.254708	1.180443	-2.561503
63	1	-3.369377	2.923564	-2.308869
64	1	-1.882164	2.109618	-1.892340

65	6	-5.111036	1.956109	-0.681611
66	1	-5.496361	1.124793	-1.280486
67	1	-5.652422	1.983445	0.266586
68	1	-5.319964	2.889402	-1.212763
69	6	-2.879177	-3.109657	-2.028135
70	1	-2.529783	-4.006792	-2.549400
71	1	-3.970189	-3.167559	-1.950860
72	1	-2.619787	-2.240008	-2.630075
73	6	-0.709553	-3.140594	-0.673761
74	1	-0.420984	-4.096047	-1.123200
75	1	-0.247343	-2.358815	-1.276416
76	1	-0.286954	-3.101877	0.335890
77	6	-2.689874	-4.388152	0.071297
78	1	-2.252161	-4.499067	1.068347
79	1	-3.778519	-4.466830	0.146579
80	1	-2.339165	-5.224472	-0.539243
81	1	-0.986841	1.438086	0.022347

DAL with acylammonium ion formed with Et_3N (endo):

Transition state (TS1):

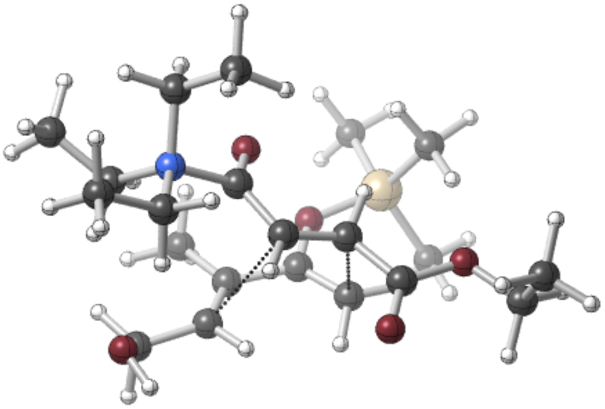

Charge = 1 Multiplicity = 1

HF = -1543.9526324 hartrees (-968845.716357324 kcal/mol)

Imaginary Frequencies: 1 (-290.1271 1/cm)

Zero-point correction = 0.598902 (Hartree/Particle)

Temperature 298.150 Kelvin. Pressure 1.00000 Atm.

Sum of electronic and thermal Free Energies =

-1543.419726 hartrees (-968511.31226226 kcal/mol)

Coordinates (from last standard orientation):

Center Number	Atomic Number	Coordinates (Angstroms) X	Y	Z
1	6	1.305621	0.134358	0.679132
2	6	0.691631	1.172388	-0.077880
3	6	-0.679957	1.282919	0.060148
4	6	0.746670	-0.913161	-2.266531
5	6	-1.817208	-0.021155	-1.256586
6	6	2.051495	-1.241494	-2.933053
7	6	-1.277350	-1.257899	-0.957921
8	6	-4.723815	-1.912003	-0.529723
9	6	-3.340253	-0.556723	1.902152
10	6	-3.383086	-3.634882	1.647663
11	6	-0.002885	-1.734682	-1.497313
12	6	-1.337570	2.496538	-0.519389
13	6	-3.268685	3.859363	-0.433708
14	6	-2.785659	5.122231	0.248346
15	6	0.416113	-3.112524	-1.060567
16	1	1.231733	1.842656	-0.729512
17	1	0.359920	0.066741	-2.538544
18	1	-2.804600	0.232807	-0.885366
19	1	-1.514019	0.487847	-2.162436
20	1	-1.159910	0.833869	0.923071
21	1	2.555797	-2.088694	-2.464676
22	1	1.857311	-1.517573	-3.979988
23	1	-4.626308	-2.704710	-1.279867
24	1	-5.686903	-2.053901	-0.024628
25	1	-4.766138	-0.952026	-1.053500
26	1	-2.441646	-0.563305	2.529169
27	1	-3.395953	0.407402	1.384324
28	1	-4.207429	-0.621011	2.570667
29	1	-3.332993	-4.480542	0.953423
30	1	-2.539260	-3.714637	2.341320
31	1	-4.307208	-3.730767	2.228493
32	1	-4.306328	3.637429	-0.180674
33	1	-3.166597	3.920884	-1.519506
34	1	-3.400760	5.968539	-0.071362
35	1	-2.867429	5.026281	1.334750
36	1	-1.745517	5.333266	-0.013645
37	1	2.625645	0.556817	-3.442872
38	1	0.502821	-3.142527	0.031300
39	1	-0.340465	-3.852511	-1.340320
40	1	1.365802	-3.416410	-1.501562
41	8	0.732904	-0.751142	1.277735

42	8	-1.841615	-2.091106	-0.087111
43	8	-0.848609	3.183129	-1.387712
44	8	-2.532337	2.708639	0.035770
45	8	2.964163	-0.152814	-2.872410
46	14	-3.347868	-2.014535	0.731049
47	7	2.857290	0.125217	0.832212
48	6	3.103176	0.181774	2.329246
49	1	2.584961	-0.685173	2.739997
50	1	4.172990	0.050900	2.485004
51	6	3.500562	1.286544	0.110584
52	1	3.231265	1.158379	-0.939850
53	1	3.032737	2.194390	0.491798
54	6	3.349199	-1.201138	0.240370
55	1	2.484407	-1.865804	0.240072
56	1	3.619124	-0.967901	-0.790873
57	6	4.497717	-1.879772	0.968485
58	1	4.767095	-2.757300	0.372928
59	1	5.388104	-1.253975	1.058098
60	1	4.206503	-2.232173	1.960127
61	6	2.615352	1.468818	2.969252
62	1	1.562032	1.669860	2.751456
63	1	2.715757	1.358548	4.052299
64	1	3.210674	2.334300	2.667632
65	6	5.009575	1.365653	0.248626
66	1	5.511801	0.515322	-0.218277
67	1	5.331037	2.266066	-0.282710
68	1	5.341174	1.461039	1.285884

Intermediate (INT):

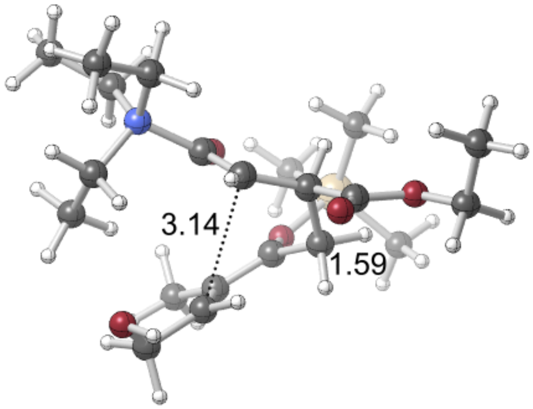

Charge = 1 Multiplicity = 1

HF = -1543.9613262 hartrees (-968851.171803762 kcal/mol)

Imaginary Frequencies: none found

Zero-point correction = 0.601472 (Hartree/Particle)

Temperature 298.150 Kelvin. Pressure 1.00000 Atm.

Sum of electronic and thermal Free Energies =

-1543.424930 hartrees (-968514.5778243 kcal/mol)

Coordinates (from last standard orientation):

Center Number	Atomic Number	Coordinates (Angstroms) X	Y	Z
1	6	1.130325	0.173182	0.675130
2	6	0.540825	1.246473	0.071970
3	6	-0.949332	1.215647	0.070999
4	6	0.539152	-0.457569	-2.562849
5	6	-1.706951	0.169896	-0.855875
6	6	1.679637	-0.538222	-3.534481
7	6	-1.097760	-1.181987	-0.958694
8	6	-4.443184	-2.468998	0.007070
9	6	-2.683260	-1.043490	2.155092
10	6	-2.285242	-4.058066	1.592898
11	6	-0.007532	-1.480658	-1.862540
12	6	-1.535390	2.568413	-0.293137
13	6	-3.531113	3.843811	-0.301496
14	6	-3.288106	4.890410	0.765540
15	6	0.450571	-2.913173	-1.901037
16	1	1.056279	2.136161	-0.260684

17	1	0.114340	0.539037	-2.483202
18	1	-2.725471	0.079526	-0.473003
19	1	-1.780933	0.582862	-1.864077
20	1	-1.302971	0.970421	1.078053
21	1	2.263360	-1.451642	-3.413290
22	1	1.262156	-0.548258	-4.553249
23	1	-4.444472	-3.254728	-0.755848
24	1	-5.254410	-2.683520	0.712253
25	1	-4.670146	-1.515300	-0.481437
26	1	-1.633862	-0.846880	2.398044
27	1	-3.153789	-0.113246	1.818908
28	1	-3.191716	-1.359249	3.074320
29	1	-2.283037	-4.828515	0.814583
30	1	-1.275642	-3.992817	2.012859
31	1	-2.962713	-4.383870	2.389770
32	1	-4.582835	3.557003	-0.348104
33	1	-3.207683	4.189714	-1.285937
34	1	-3.872692	5.786942	0.538291
35	1	-3.595071	4.516995	1.746604
36	1	-2.231534	5.167585	0.802997
37	1	2.133279	1.355279	-3.654548
38	1	0.727719	-3.244551	-0.894852
39	1	-0.357485	-3.564919	-2.248587
40	1	1.303386	-3.050554	-2.566343
41	8	0.583195	-0.908560	0.966786
42	8	-1.613992	-2.189937	-0.355744
43	8	-0.929164	3.473593	-0.817669
44	8	-2.837157	2.617683	0.012043
45	8	2.578968	0.543073	-3.363033
46	14	-2.813541	-2.416120	0.906006
47	7	2.672153	0.293778	1.050286
48	6	3.486191	0.217134	-0.221430
49	1	3.066686	0.959705	-0.902994
50	1	4.510237	0.502539	0.018632
51	6	2.983632	-0.857482	1.977356
52	1	2.290634	-0.755689	2.813937
53	1	2.708965	-1.765636	1.446449
54	6	2.903253	1.611763	1.786165
55	1	2.145622	2.306195	1.435317
56	1	2.687865	1.397158	2.835546
57	6	4.270630	2.260098	1.629695
58	1	4.287529	3.121277	2.304794
59	1	5.105004	1.610561	1.899219
60	1	4.428163	2.636483	0.616115
61	6	3.472849	-1.159288	-0.856643
62	1	2.457808	-1.553421	-0.944603

63	1	3.899537	-1.065454	-1.858427
64	1	4.079809	-1.874783	-0.295559
65	6	4.418631	-0.909770	2.469749
66	1	4.651223	-0.093040	3.157186
67	1	4.540975	-1.847069	3.020255
68	1	5.148612	-0.908613	1.654567

Transition state 2 (TS2):

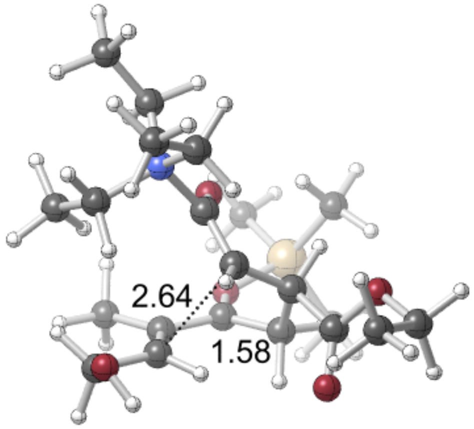

Charge = 1 Multiplicity = 1

HF = -1543.9595835 hartrees (-968850.078242085 kcal/mol)

Imaginary Frequencies: 1 (-114.5760 1/cm)

Zero-point correction = 0.601943 (Hartree/Particle)

Temperature 298.150 Kelvin. Pressure 1.00000 Atm.

Sum of electronic and thermal Free Energies =

-1543.421457 hartrees (-968512.39848207 kcal/mol)

Coordinates (from last standard orientation):

Center Number	Atomic Number	Coordinates (Angstroms) X	Y	Z
1	6	-0.818241	-0.623805	-0.615659
2	6	-0.924943	0.603871	0.007939
3	6	0.248613	1.512896	-0.230884
4	6	-0.013930	-0.127056	2.371868
5	6	1.585394	1.314363	0.594572
6	6	-1.042065	-0.700406	3.298480
7	6	1.905078	-0.077637	0.983455
8	6	5.263427	0.884316	-0.167062
9	6	3.023575	0.284387	-2.264400

10	6	4.761112	-2.014888	-1.151884
11	6	1.108668	-0.776174	1.922684
12	6	-0.239718	2.927688	0.021092
13	6	-1.374592	4.767788	-0.929813
14	6	-2.770537	4.617433	-0.360725
15	6	1.491409	-2.189636	2.271347
16	1	-1.850890	1.002145	0.403243
17	1	-0.105348	0.947096	2.249610
18	1	2.400790	1.747716	0.012102
19	1	1.490745	1.910035	1.506870
20	1	0.539256	1.420207	-1.281331
21	1	-1.212800	-1.761768	3.113398
22	1	-0.651064	-0.613713	4.325966
23	1	5.768922	0.495162	0.723332
24	1	6.028517	1.077969	-0.927987
25	1	4.803142	1.843576	0.093323
26	1	2.061611	-0.237635	-2.314140
27	1	2.835758	1.362083	-2.217868
28	1	3.570062	0.083067	-3.193561
29	1	5.244943	-2.420969	-0.257390
30	1	3.993053	-2.724733	-1.477170
31	1	5.516668	-1.950798	-1.943023
32	1	-1.394850	5.179973	-1.939527
33	1	-0.744533	5.395977	-0.296574
34	1	-3.253609	5.597644	-0.308486
35	1	-3.377641	3.967129	-0.997410
36	1	-2.735111	4.198205	0.648501
37	1	-2.205789	0.852387	3.495726
38	1	1.593383	-2.804229	1.371477
39	1	2.462061	-2.197807	2.779226
40	1	0.763879	-2.655757	2.938493
41	8	0.230052	-1.157123	-0.985901
42	8	2.936596	-0.702799	0.498661
43	8	-0.224014	3.465249	1.107227
44	8	-0.742932	3.478070	-1.082191
45	8	-2.295785	-0.059072	3.172353
46	14	4.019874	-0.343496	-0.818477
47	7	-2.148947	-1.447745	-0.873925
48	6	-2.610974	-2.027069	0.445768
49	1	-2.688384	-1.184113	1.138704
50	1	-3.607643	-2.442125	0.298380
51	6	-1.800251	-2.536496	-1.864590
52	1	-1.426063	-2.018014	-2.749145
53	1	-0.965373	-3.088791	-1.441402
54	6	-3.202967	-0.529977	-1.495254
55	1	-2.950520	0.486936	-1.207211

56	1	-3.051762	-0.606115	-2.574316
57	6	-4.652955	-0.804982	-1.129583
58	1	-5.262378	-0.127953	-1.735933
59	1	-4.975916	-1.825392	-1.345838
60	1	-4.858951	-0.582875	-0.079877
61	6	-1.677224	-3.102748	0.969097
62	1	-0.641568	-2.756732	1.005190
63	1	-1.995034	-3.370657	1.980445
64	1	-1.722043	-4.012850	0.365658
65	6	-2.954032	-3.454270	-2.221906
66	1	-3.740719	-2.940664	-2.779228
67	1	-2.555468	-4.241706	-2.868104
68	1	-3.395355	-3.938929	-1.346413

Product:

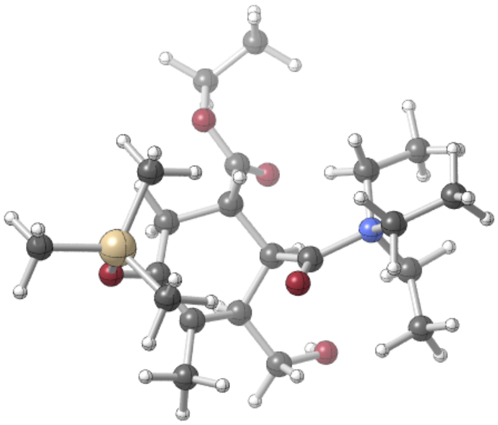

Charge = 1 Multiplicity = 1

HF = -1544.0211088 hartrees (-968888.685983088 kcal/mol)

Imaginary Frequencies: none found

Zero-point correction = 0.605365 (Hartree/Particle)

Temperature 298.150 Kelvin. Pressure 1.00000 Atm.

Sum of electronic and thermal Free Energies =

-1543.478691 hartrees (-968548.31338941 kcal/mol)

Coordinates (from last standard orientation):

Center Number	Atomic Number	Coordinates (Angstroms) X	Y	Z

1	6	0.848752	-1.073365	0.175664
2	6	0.629354	-0.072574	-0.944440
3	6	-0.018211	1.226576	-0.380029
4	6	-0.261853	-0.701717	-2.052033
5	6	-1.457661	1.472807	-0.907241
6	6	0.349576	-1.984665	-2.619340
7	6	-2.248744	0.197633	-1.007774
8	6	-5.731797	0.286342	1.219969
9	6	-2.870955	0.952685	2.189668
10	6	-3.556203	-1.914519	1.407237
11	6	-1.698090	-0.875855	-1.585792
12	6	0.826260	2.447022	-0.726889
13	6	0.995348	4.788880	-0.447109
14	6	2.175449	4.952303	0.487724
15	6	-2.425326	-2.174617	-1.780392
16	1	1.587152	0.193032	-1.394678
17	1	-0.235063	0.048997	-2.859404
18	1	-1.960711	2.187615	-0.253503
19	1	-1.389341	1.935470	-1.902180
20	1	-0.077352	1.189104	0.714308
21	1	0.241365	-2.812175	-1.911890
22	1	-0.183272	-2.270125	-3.533991
23	1	-6.336114	-0.324203	0.540411
24	1	-6.078878	0.091043	2.241069
25	1	-5.928944	1.339826	0.994294
26	1	-1.801653	0.741888	2.065283
27	1	-3.033084	2.022825	2.021840
28	1	-3.122333	0.737449	3.235434
29	1	-4.186851	-2.583355	0.812258
30	1	-2.507606	-2.150639	1.192305
31	1	-3.739566	-2.133472	2.466072
32	1	0.239382	5.557049	-0.280168
33	1	1.301614	4.805773	-1.494959
34	1	2.647303	5.924217	0.315734
35	1	1.848731	4.908217	1.530594
36	1	2.922019	4.172516	0.312947
37	1	1.856755	-1.243631	-3.611062
38	1	-1.992655	-2.975618	-1.168417
39	1	-3.475780	-2.065616	-1.502680
40	1	-2.386975	-2.504514	-2.825085
41	8	0.039745	-1.853972	0.568759
42	8	-3.542365	0.242490	-0.557954
43	8	1.823779	2.435038	-1.415201
44	8	0.304713	3.545247	-0.186749
45	8	1.739570	-1.855119	-2.865002
46	14	-3.917481	-0.112129	1.054860

47	7	2.245401	-1.117944	0.870921
48	6	3.185797	-1.841371	-0.090722
49	1	3.152988	-1.288288	-1.030382
50	1	4.183631	-1.747190	0.334139
51	6	2.079069	-1.890370	2.168283
52	1	1.310829	-1.356562	2.731660
53	1	1.677169	-2.864413	1.899716
54	6	2.733621	0.300943	1.162640
55	1	2.320686	0.948607	0.395684
56	1	2.285598	0.570525	2.121609
57	6	4.239158	0.519272	1.160075
58	1	4.398626	1.547068	1.499674
59	1	4.789712	-0.142699	1.828964
60	1	4.654738	0.438660	0.153543
61	6	2.844460	-3.304117	-0.306384
62	1	1.796114	-3.471960	-0.563325
63	1	3.447737	-3.654918	-1.147451
64	1	3.093675	-3.913700	0.565824
65	6	3.348549	-2.029946	2.984335
66	1	3.685742	-1.077681	3.398259
67	1	3.113637	-2.689038	3.824920
68	1	4.165038	-2.492226	2.422631

DAL with acylammonium ion formed with Et_3N (exo):

Transition state 1 (TS1):

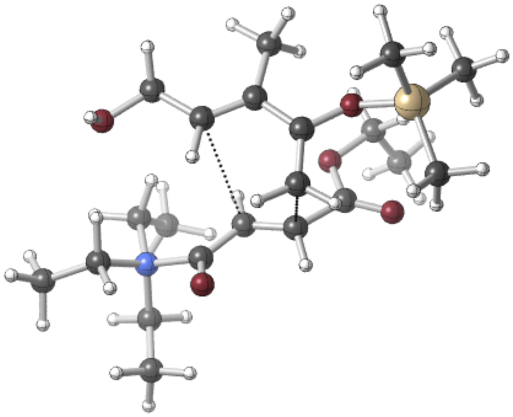

Charge = 1 Multiplicity = 1

HF = -1543.9494811 hartrees (-968843.738885061 kcal/mol)

Imaginary Frequencies: 1 (-370.8361 1/cm)

Zero-point correction = 0.598945 (Hartree/Particle)

Temperature 298.150 Kelvin. Pressure 1.00000 Atm.

Sum of electronic and thermal Free Energies =

-1543.414868 hartrees (-968508.26381868 kcal/mol)

Coordinates (from last standard orientation):

Center Number	Atomic Number	Coordinates (Angstroms) X	Y	Z
1	6	-2.231781	0.369460	-0.925773
2	6	0.044435	1.090981	-0.830686
3	6	-1.130313	0.803045	-0.140072
4	6	-0.380273	-2.092889	0.637917
5	6	1.043366	-0.631213	-1.457480
6	6	0.898358	-1.691170	0.812986
7	6	1.623186	-1.030553	-0.264595
8	6	-1.219628	-2.747592	1.703464
9	6	4.145717	0.330509	-2.473135
10	6	4.779038	-2.364299	-1.113951
11	6	5.469178	0.329774	0.294937
12	6	1.628743	-1.841011	2.121472
13	6	1.199875	1.727335	-0.122908
14	6	2.247038	2.048571	1.971831
15	6	2.151019	3.547643	2.168406
16	1	-0.022697	1.360629	-1.880889
17	1	-1.156379	0.855228	0.935746
18	1	-0.858352	-2.024124	-0.336793
19	1	1.678983	-0.191782	-2.217985
20	1	0.137944	-1.104173	-1.819577
21	1	-0.713395	-3.644857	2.087663
22	1	-1.352603	-2.071211	2.556384
23	1	5.155525	0.578487	-2.824467
24	1	3.668124	-0.270431	-3.253854
25	1	3.593212	1.268590	-2.357319
26	1	4.929015	-2.882894	-0.160828
27	1	3.993880	-2.892648	-1.667515
28	1	5.705714	-2.443783	-1.693528
29	1	6.501499	0.239670	-0.062631
30	1	5.225530	1.397303	0.332409
31	1	5.428667	-0.070992	1.313291
32	1	-2.441921	-3.819350	0.633598
33	1	1.961934	-0.864410	2.486680
34	1	1.003314	-2.302736	2.887299
35	1	2.526127	-2.455705	1.993493

36	1	3.175320	1.758357	1.473721
37	1	2.169839	1.520488	2.923953
38	1	1.198518	3.813212	2.636106
39	1	2.961174	3.878516	2.825195
40	1	2.241278	4.074558	1.215802
41	8	-2.203239	0.082646	-2.106105
42	8	2.852565	-0.631450	0.063943
43	8	-2.520200	-3.064778	1.240035
44	8	1.138086	1.545862	1.196961
45	8	2.103359	2.295863	-0.695923
46	14	4.310601	-0.576402	-0.847305
47	7	-3.630051	0.273378	-0.253724
48	6	-4.287654	-0.995989	-0.778212
49	1	-4.091683	-1.018093	-1.847855
50	1	-3.733345	-1.808552	-0.299825
51	6	-4.410782	1.514800	-0.652128
52	1	-5.340242	1.468240	-0.079062
53	1	-3.828415	2.363865	-0.289595
54	6	-3.554407	0.168033	1.260586
55	1	-2.719381	-0.502551	1.463975
56	1	-4.462909	-0.348003	1.570025
57	6	-5.777343	-1.107786	-0.504996
58	1	-6.097519	-2.079665	-0.891879
59	1	-6.031924	-1.083027	0.557410
60	1	-6.358943	-0.341843	-1.023704
61	6	-4.686401	1.665778	-2.137264
62	1	-5.338017	2.537207	-2.250854
63	1	-3.775124	1.845824	-2.708625
64	1	-5.204126	0.804759	-2.566197
65	6	-3.445825	1.474993	2.031947
66	1	-4.380935	2.038211	2.008021
67	1	-3.245744	1.213006	3.075073
68	1	-2.637847	2.125002	1.690111

Intermediate (INT):

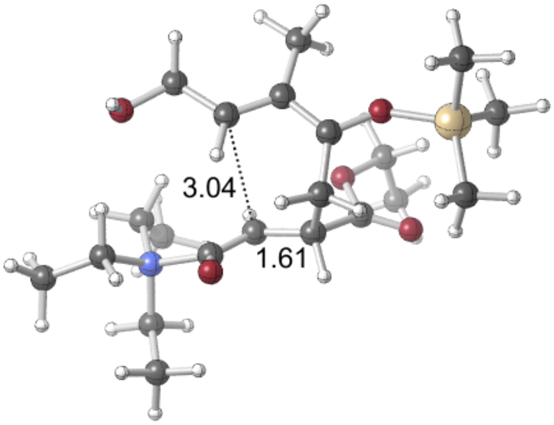

Charge = 1 Multiplicity = 1

HF = -1543.9613145 hartrees (-968851.164461895 kcal/mol)

Imaginary Frequencies: none found

Zero-point correction = 0.600705 (Hartree/Particle)

Temperature 298.150 Kelvin. Pressure 1.00000 Atm.

Sum of electronic and thermal Free Energies =

-1543.426124 hartrees (-968515.32707124 kcal/mol)

Coordinates (from last standard orientation):

Center Number	Atomic Number	Coordinates (Angstroms) X	Y	Z
1	6	2.117550	-0.194429	-0.925447
2	6	-0.217651	-0.868286	-0.882312
3	6	1.093602	-0.741055	-0.193214
4	6	0.420854	2.089176	0.705162
5	6	-0.914159	0.514231	-1.324435
6	6	-0.891771	1.782167	0.890928
7	6	-1.589331	1.117186	-0.167782
8	6	1.278911	2.740892	1.748462
9	6	-3.993746	-0.152001	-2.566495
10	6	-5.175500	2.161271	-0.899234
11	6	-5.163292	-0.732052	0.226903
12	6	-1.655411	2.061775	2.157791
13	6	-1.263490	-1.646487	-0.106710
14	6	-2.063324	-2.203327	2.056261
15	6	-1.783866	-3.689512	2.139241
16	1	-0.104920	-1.367544	-1.850819

17	1	1.199515	-1.100019	0.815950
18	1	0.896942	1.940114	-0.260051
19	1	-1.634022	0.287301	-2.108234
20	1	-0.112181	1.125625	-1.739363
21	1	0.791431	3.661682	2.101398
22	1	1.364842	2.079211	2.619352
23	1	-4.972197	-0.421219	-2.986466
24	1	-3.542872	0.581950	-3.241411
25	1	-3.382473	-1.059358	-2.549367
26	1	-5.328596	2.557385	0.110072
27	1	-4.600778	2.896693	-1.472649
28	1	-6.157487	2.058934	-1.375192
29	1	-6.209664	-0.829603	-0.086221
30	1	-4.690144	-1.714248	0.134437
31	1	-5.156085	-0.427569	1.278605
32	1	2.556178	3.754751	0.688693
33	1	-2.058184	1.131512	2.571799
34	1	-1.028117	2.531939	2.916435
35	1	-2.505194	2.721857	1.956259
36	1	-3.064958	-1.998923	1.672355
37	1	-1.946364	-1.721733	3.028326
38	1	-0.765254	-3.868455	2.495458
39	1	-2.481894	-4.151150	2.843843
40	1	-1.910250	-4.166255	1.164139
41	8	2.068197	0.334464	-2.043711
42	8	-2.848382	0.905674	0.032854
43	8	2.591004	2.997753	1.296250
44	8	-1.097862	-1.539080	1.213137
45	8	-2.194097	-2.225301	-0.626139
46	14	-4.313063	0.512351	-0.857335
47	7	3.572627	-0.266168	-0.295460
48	6	4.325987	0.959777	-0.767751
49	1	4.079875	1.079783	-1.820125
50	1	3.879659	1.789609	-0.213827
51	6	4.213075	-1.535708	-0.810098
52	1	5.193531	-1.610263	-0.332307
53	1	3.590621	-2.354215	-0.446205
54	6	3.554641	-0.241858	1.225391
55	1	2.704063	0.384559	1.493792
56	1	4.457949	0.284659	1.534879
57	6	5.830721	0.903079	-0.566280
58	1	6.232547	1.882437	-0.841932
59	1	6.127102	0.708339	0.468286
60	1	6.308845	0.160713	-1.210061
61	6	4.337851	-1.613236	-2.321814
62	1	4.848081	-2.551465	-2.558742

63	1	3.362509	-1.616802	-2.810256
64	1	4.931178	-0.795400	-2.738578
65	6	3.510559	-1.583379	1.943091
66	1	4.435200	-2.148600	1.806469
67	1	3.412611	-1.368047	3.011450
68	1	2.669292	-2.215348	1.651651

Transition state 2 (TS2):

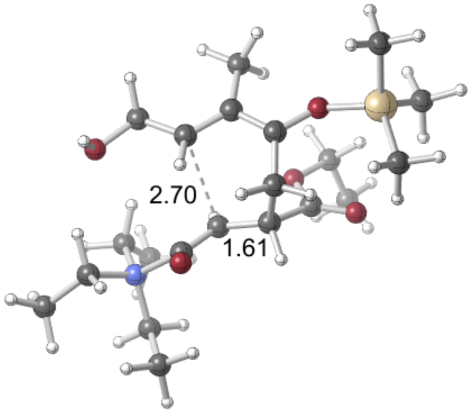

Charge = 1 Multiplicity = 1

HF = -1543.9607073 hartrees (-968850.783437823 kcal/mol)

Imaginary Frequencies: 1 (-98.0985 1/cm)

Zero-point correction = 0.600784 (Hartree/Particle)

Temperature 298.150 Kelvin. Pressure 1.00000 Atm.

Sum of electronic and thermal Free Energies =

-1543.424214 hartrees (-968514.12852714 kcal/mol)

Coordinates (from last standard orientation):

Center Number	Atomic Number	Coordinates (Angstroms) X	Y	Z
1	6	-2.155448	-0.066149	-0.853844
2	6	0.170589	0.665987	-0.900679
3	6	-1.095378	0.476278	-0.145072
4	6	-0.385880	-1.950788	0.802044
5	6	0.980934	-0.666371	-1.304406
6	6	0.945761	-1.673243	0.996071
7	6	1.667301	-1.149031	-0.098482
8	6	-1.289258	-2.417602	1.903567
9	6	4.076744	-0.123931	-2.581143

10	6	5.313324	-2.141912	-0.604683
11	6	5.179152	0.870206	0.116999
12	6	1.640324	-1.755737	2.329639
13	6	1.190986	1.555803	-0.215186
14	6	2.000992	2.343997	1.869122
15	6	1.709793	3.828647	1.799301
16	1	-0.028507	1.105887	-1.884101
17	1	-1.209884	0.952670	0.813698
18	1	-0.794052	-2.054226	-0.198914
19	1	1.689219	-0.394384	-2.083261
20	1	0.239985	-1.357142	-1.707842
21	1	-0.844679	-3.300533	2.386676
22	1	-1.370383	-1.647063	2.679396
23	1	5.058930	0.053283	-3.038769
24	1	3.598390	-0.932973	-3.142284
25	1	3.488695	0.792508	-2.692264
26	1	4.763031	-2.978302	-1.049216
27	1	6.283635	-2.074148	-1.109849
28	1	5.497377	-2.374496	0.449539
29	1	6.212264	0.988480	-0.230960
30	1	4.648725	1.808292	-0.074722
31	1	5.208358	0.692242	1.197138
32	1	-2.570840	-3.522805	0.944055
33	1	2.554197	-2.352068	2.248848
34	1	1.935574	-0.755614	2.667240
35	1	1.006974	-2.204997	3.096405
36	1	3.000607	2.106454	1.498899
37	1	1.896465	1.965358	2.887025
38	1	0.690201	4.034611	2.137961
39	1	2.404792	4.367125	2.450476
40	1	1.830048	4.203342	0.780035
41	8	-2.107588	-0.695991	-1.910179
42	8	2.920648	-0.852476	0.111343
43	8	-2.598156	-2.695760	1.452987
44	8	1.034840	1.587146	1.108826
45	8	2.103659	2.095099	-0.804027
46	14	4.377183	-0.541609	-0.789859
47	7	-3.610286	0.184838	-0.298514
48	6	-4.460171	-1.016110	-0.665190
49	1	-4.190629	-1.278109	-1.685794
50	1	-4.113130	-1.811299	-0.003253
51	6	-4.122314	1.438216	-0.978614
52	1	-5.106418	1.638306	-0.547211
53	1	-3.447513	2.241505	-0.680493
54	6	-3.643209	0.342091	1.215304
55	1	-2.868905	-0.324884	1.590770

56	1	-4.602582	-0.059576	1.542334
57	6	-5.958664	-0.799553	-0.539046
58	1	-6.440862	-1.766741	-0.708793
59	1	-6.267618	-0.451574	0.450663
60	1	-6.345237	-0.103980	-1.287831
61	6	-4.196277	1.356934	-2.493328
62	1	-4.630921	2.296510	-2.846772
63	1	-3.210033	1.242326	-2.945249
64	1	-4.834460	0.541094	-2.841854
65	6	-3.496730	1.749930	1.775041
66	1	-4.360937	2.375226	1.540396
67	1	-3.447981	1.651790	2.863779
68	1	-2.593106	2.266451	1.445622

Product:

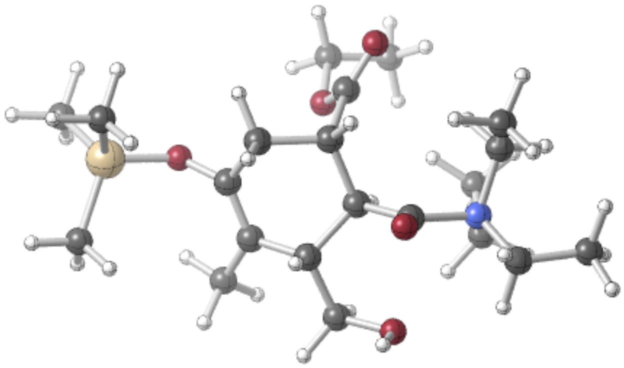

Charge = 1 Multiplicity = 1

HF = -1544.0255228 hartrees (-968891.455812228 kcal/mol)

Imaginary Frequencies: none found

Zero-point correction = 0.605041 (Hartree/Particle)

Temperature 298.150 Kelvin. Pressure 1.00000 Atm.

Sum of electronic and thermal Free Energies =

-1543.483430 hartrees (-968551.2871593 kcal/mol)

Coordinates (from last standard orientation):

Center Number	Atomic Number	Coordinates (Angstroms) X	Y	Z
1	6	2.041220	-0.935269	0.625334
2	6	0.067504	0.502104	1.030158

3	6	0.750948	-0.453060	0.000269
4	6	-0.135803	-1.685884	-0.349335
5	6	-1.414101	0.121820	1.277754
6	6	-1.510209	-1.212281	-0.787403
7	6	-2.105782	-0.322719	0.017787
8	6	0.569639	-2.523128	-1.415288
9	6	-4.641830	-0.296928	2.272065
10	6	-5.085073	-2.055385	-0.198882
11	6	-6.054649	0.887140	-0.210873
12	6	-2.133701	-1.704566	-2.063614
13	6	0.152519	1.951696	0.589435
14	6	-0.617986	3.528796	-1.003010
15	6	0.629305	4.048062	-1.686226
16	1	0.589331	0.458197	1.988707
17	1	0.920782	0.076483	-0.935992
18	1	-0.214961	-2.293812	0.565518
19	1	-1.944245	0.969677	1.721990
20	1	-1.421965	-0.689483	2.016700
21	1	-0.013019	-3.420424	-1.651183
22	1	0.685919	-1.943293	-2.337229
23	1	-5.584140	-0.647015	2.710565
24	1	-3.847622	-0.959006	2.635574
25	1	-4.450697	0.709875	2.659445
26	1	-4.252300	-2.722630	0.052260
27	1	-5.985831	-2.459311	0.278667
28	1	-5.237012	-2.092262	-1.282822
29	1	-7.051898	0.597400	0.138914
30	1	-5.854227	1.902137	0.148506
31	1	-6.078720	0.913661	-1.305732
32	1	1.822569	-3.530427	-0.297501
33	1	-3.066881	-1.170094	-2.252990
34	1	-1.476332	-1.548344	-2.926577
35	1	-2.359862	-2.777259	-2.021996
36	1	-0.878400	4.134708	-0.132392
37	1	-1.466319	3.503186	-1.688402
38	1	0.876771	3.443095	-2.563491
39	1	0.452892	5.075487	-2.019544
40	1	1.478972	4.051663	-0.998284
41	8	2.073637	-1.615105	1.606208
42	8	-3.312992	0.253133	-0.262110
43	8	1.889630	-2.875909	-1.014355
44	8	-0.470879	2.156233	-0.573545
45	8	0.688075	2.824976	1.235980
46	14	-4.764634	-0.311974	0.402831
47	7	3.391972	-0.414451	0.089949
48	6	4.439689	-1.488778	0.363629

49	1	4.242859	-1.866590	1.364411
50	1	4.216074	-2.279203	-0.356423
51	6	3.691192	0.867090	0.861015
52	1	4.608388	1.262265	0.420036
53	1	2.881463	1.562232	0.630275
54	6	3.375230	-0.141169	-1.415468
55	1	2.734462	-0.909475	-1.848354
56	1	4.391797	-0.339042	-1.753767
57	6	5.876554	-1.015701	0.248271
58	1	6.508535	-1.897070	0.389959
59	1	6.122938	-0.591586	-0.728088
60	1	6.142677	-0.294626	1.024610
61	6	3.827367	0.699920	2.362583
62	1	4.122747	1.674689	2.760956
63	1	2.887142	0.417441	2.840930
64	1	4.597595	-0.022736	2.642115
65	6	2.984604	1.259648	-1.861982
66	1	3.737482	2.000779	-1.585684
67	1	2.929714	1.233697	-2.954536
68	1	2.014802	1.600735	-1.493156

DAL with acylammonium ion formed with Hünig's base (exo):

Transition state 1 (TS1):

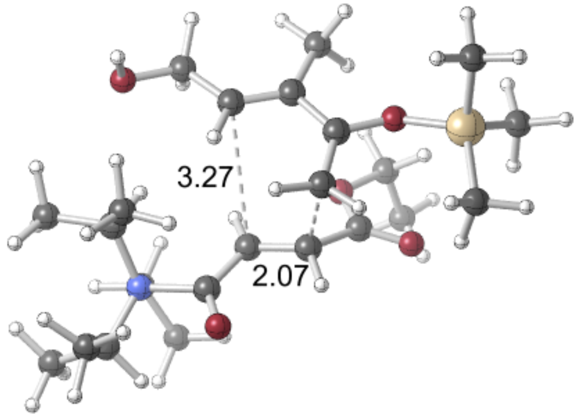

Charge = 1 Multiplicity = 1

HF = -1622.5294681 hartrees (-1018153.46652743 kcal/mol)

Imaginary Frequencies: 1 (-392.9102 1/cm)

Zero-point correction = 0.657089 (Hartree/Particle)

Temperature 298.150 Kelvin. Pressure 1.00000 Atm.

Sum of electronic and thermal Free Energies =

-1621.938567 hartrees (-1017782.67017817 kcal/mol)

Coordinates (from last standard orientation):

Center Number	Atomic Number	Coordinates (Angstroms) X	Y	Z
1	6	2.070925	-0.863572	-0.822509
2	6	-0.302515	-1.101660	-0.598512
3	6	0.913038	-0.765695	-0.000111
4	6	-0.131224	2.248769	0.712005
5	6	-1.231205	0.547180	-1.425105
6	6	-1.412735	1.830171	0.746993
7	6	-1.967854	1.036995	-0.351844
8	6	0.565913	2.978978	1.830156
9	6	-4.090080	-0.777268	-2.651333
10	6	-5.329062	1.829282	-1.578706
11	6	-5.598743	-0.823974	0.025652
12	6	-2.331193	2.081827	1.913745
13	6	-1.454184	-1.599801	0.217317
14	6	-2.456401	-1.635126	2.356712
15	6	-2.307871	-3.088129	2.758038
16	1	-0.256591	-1.576527	-1.574922
17	1	0.958353	-0.492733	1.044090
18	1	0.481056	2.067938	-0.167663
19	1	-1.767193	0.063369	-2.233420
20	1	-0.310636	1.042164	-1.710338
21	1	-0.074059	3.771467	2.239340
22	1	0.772844	2.282850	2.652947
23	1	-5.017403	-1.187905	-3.071490
24	1	-3.624786	-0.164163	-3.429817
25	1	-3.432827	-1.618550	-2.411098
26	1	-5.614388	2.397431	-0.686841
27	1	-4.640628	2.445102	-2.168830
28	1	-6.231207	1.670493	-2.180734
29	1	-6.585414	-1.003625	-0.416706
30	1	-5.127604	-1.795812	0.207279
31	1	-5.747689	-0.323290	0.988230
32	1	1.654288	4.282905	0.877587
33	1	-2.711514	1.137767	2.314511
34	1	-1.826277	2.616280	2.719267
35	1	-3.202180	2.667590	1.600874
36	1	-3.404456	-1.444526	1.847204
37	1	-2.375299	-0.976763	3.223141
38	1	-1.338814	-3.255545	3.237040

39	1	-3.095028	-3.348206	3.472041
40	1	-2.396338	-3.746059	1.890511
41	8	2.122014	-1.221117	-1.979443
42	8	-3.207702	0.625371	-0.130140
43	8	1.820872	3.505130	1.434562
44	8	-1.379298	-1.215556	1.491245
45	8	-2.359773	-2.258352	-0.246863
46	14	-4.549257	0.195853	-1.123850
47	7	3.434233	-0.459866	-0.158900
48	6	3.352136	1.073564	0.053590
49	1	2.557571	1.160505	0.799471
50	6	4.588280	-0.985585	-1.051686
51	1	4.224499	-1.969360	-1.350835
52	6	3.522702	-1.124418	1.205546
53	1	2.763798	-0.663112	1.835948
54	1	4.488517	-0.854859	1.626805
55	6	4.615292	1.688518	0.639477
56	1	4.345681	2.705555	0.939702
57	1	4.981946	1.171026	1.528531
58	1	5.418423	1.755633	-0.098680
59	6	4.826768	-0.175350	-2.322040
60	1	5.463090	-0.789655	-2.966136
61	1	3.911791	0.043092	-2.868652
62	1	5.370065	0.750912	-2.115550
63	6	3.344446	-2.632877	1.169973
64	1	4.105372	-3.136621	0.569340
65	1	3.433314	-3.000910	2.195947
66	1	2.357339	-2.917995	0.797148
67	6	5.915384	-1.162830	-0.313263
68	1	6.631768	-1.524668	-1.056720
69	1	6.306926	-0.226206	0.087844
70	1	5.884041	-1.906002	0.484858
71	6	2.900648	1.858298	-1.177582
72	1	2.075089	1.386993	-1.719047
73	1	2.547091	2.822225	-0.803711
74	1	3.713736	2.051314	-1.876231

Intermediate (INT):

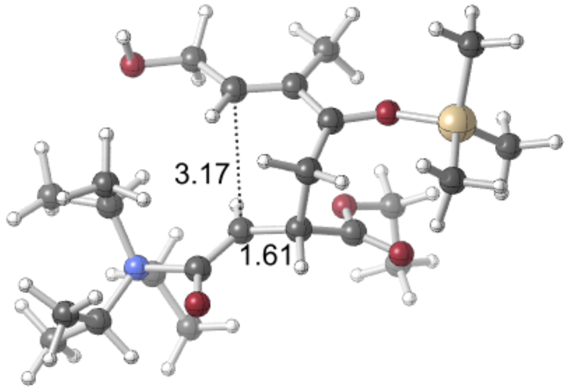

Charge = 1 Multiplicity = 1

HF = -1622.5400487 hartrees (-1018160.10595974 kcal/mol)

Imaginary Frequencies: none found

Zero-point correction = 0.658569 (Hartree/Particle)

Temperature 298.150 Kelvin. Pressure 1.00000 Atm.

Sum of electronic and thermal Free Energies =

-1621.948203 hartrees (-1017788.71686453 kcal/mol)

Coordinates (from last standard orientation):

Center Number	Atomic Number	Coordinates (Angstroms) X	Y	Z
1	6	1.991536	-0.803743	-0.893953
2	6	-0.445777	-0.818014	-0.818020
3	6	0.855891	-0.525111	-0.170386
4	6	-0.155781	2.375344	0.594836
5	6	-1.229865	0.470077	-1.389385
6	6	-1.420756	1.909467	0.744272
7	6	-1.995000	1.086408	-0.290224
8	6	0.571352	3.163500	1.645212
9	6	-4.216050	-0.627355	-2.613789
10	6	-5.613138	1.711441	-1.143091
11	6	-5.392070	-1.074183	0.210755
12	6	-2.267928	2.159299	1.963564
13	6	-1.435035	-1.572163	0.052404
14	6	-2.224433	-1.876115	2.267293
15	6	-1.779851	-3.289412	2.582627
16	1	-0.277394	-1.426657	-1.709745
17	1	0.875438	-0.181614	0.852356

18	1	0.402612	2.191104	-0.320022
19	1	-1.909115	0.138931	-2.173172
20	1	-0.480599	1.133505	-1.817690
21	1	-0.064485	3.981690	2.010763
22	1	0.764259	2.509285	2.506011
23	1	-5.151989	-1.032031	-3.020910
24	1	-3.829974	0.093081	-3.341700
25	1	-3.512182	-1.459238	-2.514320
26	1	-5.812841	2.158240	-0.163339
27	1	-5.080188	2.448522	-1.753468
28	1	-6.575917	1.509810	-1.626188
29	1	-6.418973	-1.291570	-0.106731
30	1	-4.834393	-2.015437	0.221496
31	1	-5.437218	-0.672098	1.228279
32	1	1.674007	4.414379	0.641638
33	1	-2.609732	1.209350	2.387624
34	1	-1.715890	2.699024	2.733784
35	1	-3.159139	2.740823	1.706075
36	1	-3.231388	-1.850839	1.844704
37	1	-2.192684	-1.241416	3.154323
38	1	-0.760242	-3.290143	2.978762
39	1	-2.444970	-3.721726	3.336021
40	1	-1.814410	-3.917602	1.689040
41	8	2.132407	-1.263465	-2.026632
42	8	-3.216213	0.734946	-0.097852
43	8	1.825486	3.641066	1.208967
44	8	-1.315693	-1.243878	1.342368
45	8	-2.287207	-2.320945	-0.375806
46	14	-4.633343	0.139114	-0.971896
47	7	3.375755	-0.441794	-0.137707
48	6	3.392718	1.090506	0.009804
49	1	2.567208	1.262510	0.703827
50	6	4.533075	-1.073798	-0.932619
51	1	4.119638	-2.039776	-1.224072
52	6	3.338811	-1.049922	1.248364
53	1	2.566815	-0.522466	1.807662
54	1	4.289587	-0.827225	1.730907
55	6	4.657337	1.654735	0.646051
56	1	4.447806	2.704582	0.874361
57	1	4.930325	1.162763	1.582320
58	1	5.509455	1.624000	-0.038053
59	6	4.893090	-0.332138	-2.216112
60	1	5.518274	-1.007724	-2.808331
61	1	4.017065	-0.081451	-2.810098
62	1	5.485125	0.565915	-2.016181
63	6	3.062689	-2.544842	1.270248

64	1	3.811544	-3.126821	0.727740
65	1	3.081364	-2.871686	2.314157
66	1	2.075765	-2.772781	0.861073
67	6	5.805827	-1.310809	-0.116910
68	1	6.541206	-1.737893	-0.805696
69	1	6.231219	-0.388849	0.284193
70	1	5.682891	-2.025065	0.698721
71	6	3.061826	1.857545	-1.272845
72	1	2.317015	1.357594	-1.896987
73	1	2.652791	2.821374	-0.958018
74	1	3.947785	2.054507	-1.877557

Transition state 2 (TS2):

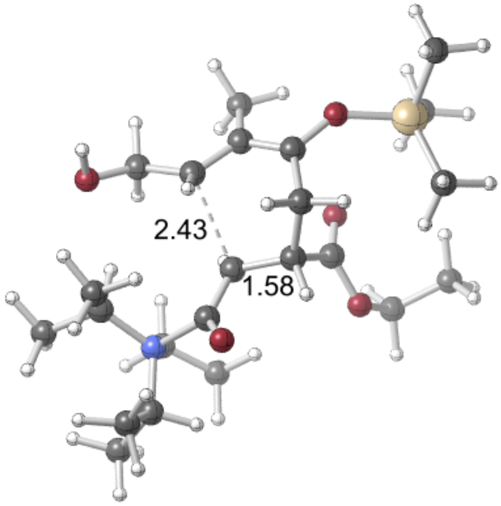

Charge = 1 Multiplicity = 1

HF = -1622.5348488 hartrees (-1018156.84297049 kcal/mol)

Imaginary Frequencies: 1 (-246.7758 1/cm)

Zero-point correction = 0.659204 (Hartree/Particle)

Temperature 298.150 Kelvin. Pressure 1.00000 Atm.

Sum of electronic and thermal Free Energies =

-1621.940719 hartrees (-1017784.02057969 kcal/mol)

Coordinates (from last standard orientation):

Center Number	Atomic Number	Coordinates (Angstroms) X	Y	Z
1	6	-1.919916	-0.428489	0.751683
2	6	0.502763	-0.521075	0.776582

3	6	-0.737982	-0.059199	0.054540
4	6	-0.147370	2.281231	-0.213429
5	6	1.367465	0.611615	1.459376
6	6	1.212569	2.139063	-0.491312
7	6	2.007142	1.452252	0.427120
8	6	-1.029674	2.962974	-1.229161
9	6	4.261709	-1.051880	1.621577
10	6	5.930828	1.482343	1.282919
11	6	5.152461	-0.124114	-1.225968
12	6	1.797051	2.532799	-1.824996
13	6	1.331559	-1.314412	-0.211903
14	6	1.806321	-3.479489	-1.042802
15	6	3.274278	-3.684865	-0.730947
16	1	0.205274	-1.182508	1.591207
17	1	2.109124	0.167324	2.122911
18	1	0.668835	1.189001	2.071308
19	1	-0.431973	3.702724	-1.779591
20	1	-1.384453	2.239935	-1.974351
21	1	5.121341	-1.733999	1.586212
22	1	4.156052	-0.721183	2.660936
23	1	3.377440	-1.637255	1.354740
24	1	6.151261	2.353688	0.657070
25	1	5.603765	1.841762	2.264477
26	1	6.865977	0.927758	1.422995
27	1	4.322504	-0.592111	-1.762375
28	1	5.490747	0.745646	-1.800330
29	1	5.983547	-0.837871	-1.180841
30	1	-1.875188	4.313920	-0.117817
31	1	2.791533	2.100779	-1.946735
32	1	1.168491	2.164656	-2.642798
33	1	1.880302	3.620242	-1.935074
34	1	1.663140	-3.038593	-2.032212
35	1	1.252058	-4.417406	-0.983544
36	1	3.403364	-4.090941	0.276736
37	1	3.697734	-4.397310	-1.445495
38	1	3.827399	-2.745195	-0.811859
39	8	-2.002425	-0.739881	1.926169
40	8	3.309134	1.456781	0.272294
41	8	-2.172338	3.574265	-0.672500
42	8	1.167672	-2.629333	-0.063237
43	8	2.017258	-0.812580	-1.079189
44	14	4.643694	0.390954	0.491736
45	7	-3.292192	-0.424790	-0.027706
46	6	-3.742437	1.054167	-0.071896
47	1	-2.984616	1.498510	-0.717461
48	6	-4.252876	-1.426708	0.671035

49	1	-3.590539	-2.261574	0.907136
50	6	-3.054532	-0.890974	-1.452148
51	1	-2.435664	-0.131241	-1.930211
52	1	-4.016761	-0.876334	-1.960145
53	6	-5.104041	1.276551	-0.718705
54	1	-5.198009	2.355665	-0.876055
55	1	-5.215194	0.796287	-1.693094
56	1	-5.924676	0.966006	-0.067193
57	6	-4.871986	-0.934620	1.977574
58	1	-5.304227	-1.815543	2.462679
59	1	-4.145385	-0.499430	2.658102
60	1	-5.690485	-0.231411	1.799567
61	6	-2.411349	-2.265462	-1.563875
62	1	-3.048495	-3.063438	-1.176966
63	1	-2.240023	-2.464248	-2.625977
64	1	-1.447410	-2.317625	-1.051833
65	6	-5.378648	-1.936118	-0.232857
66	1	-5.971168	-2.625221	0.376173
67	1	-6.046922	-1.137752	-0.560729
68	1	-5.040177	-2.495038	-1.105422
69	6	-3.672637	1.787934	1.265720
70	1	-2.747624	1.603036	1.815539
71	1	-3.705530	2.851810	1.024411
72	1	-4.519118	1.560836	1.912707
73	1	-0.719956	-0.069810	-1.026080
74	1	-0.501252	2.308472	0.811861

Product:

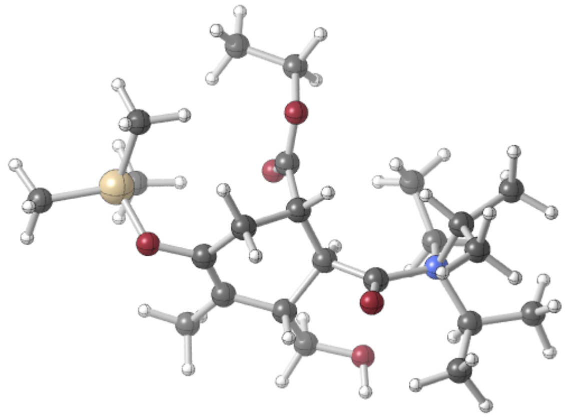

Charge = 1 Multiplicity = 1

HF = -1622.5949757 hartrees (-1018194.57320151 kcal/mol)

Imaginary Frequencies: none found

Zero-point correction = 0.665393 (Hartree/Particle)

Temperature 298.150 Kelvin. Pressure 1.00000 Atm.

Sum of electronic and thermal Free Energies =

-1621.991535 hartrees (-1017815.90812785 kcal/mol)

Coordinates (from last standard orientation):

Center Number	Atomic Number	Coordinates (Angstroms) X	Y	Z
1	6	1.984044	-0.561117	0.544298
2	6	-0.274609	0.453504	0.683287
3	6	0.622400	-0.528017	-0.129525
4	6	0.069710	-1.986779	-0.189379
5	6	-1.302746	-0.305616	1.549604
6	6	-1.444441	-2.064275	-0.171052
7	6	-2.079700	-1.292355	0.718172
8	6	0.702931	-2.651258	-1.407337
9	6	-4.488082	1.281647	1.248826
10	6	-6.216100	-1.229734	0.885678
11	6	-4.465435	-0.227750	-1.459378
12	6	-2.152438	-3.072911	-1.030903
13	6	-0.968099	1.400030	-0.287273
14	6	-1.534891	3.663802	-0.683197
15	6	-3.042022	3.708701	-0.814862
16	1	0.327423	1.074980	1.352671
17	1	-1.976783	0.402834	2.038963
18	1	-0.747820	-0.823275	2.343102
19	1	0.427101	-3.707537	-1.478689
20	1	0.376643	-2.149157	-2.324989
21	1	-5.267048	1.964548	0.888800
22	1	-4.618312	1.169457	2.331645
23	1	-3.520923	1.763826	1.074463
24	1	-6.314143	-2.202418	0.391398
25	1	-6.257584	-1.395218	1.967881
26	1	-7.084858	-0.622623	0.606564
27	1	-3.463253	0.109250	-1.746729
28	1	-4.655798	-1.188781	-1.950973
29	1	-5.191366	0.494615	-1.851656
30	1	2.478292	-3.271934	-0.859593
31	1	-3.223596	-3.069114	-0.818947
32	1	-2.013825	-2.861480	-2.098429
33	1	-1.777492	-4.088231	-0.851839
34	1	-1.056317	3.499123	-1.652202
35	1	-1.147825	4.585484	-0.245971

36	1	-3.513376	3.911331	0.150325
37	1	-3.312082	4.514684	-1.504386
38	1	-3.430365	2.767628	-1.214584
39	8	2.128934	-1.046821	1.624950
40	8	-3.416097	-1.408713	0.976923
41	8	2.121204	-2.509306	-1.343325
42	8	-1.083581	2.629407	0.223170
43	8	-1.334059	1.096005	-1.400996
44	14	-4.627851	-0.381365	0.396998
45	7	3.224408	0.149876	-0.065870
46	6	4.391139	-0.883688	-0.283357
47	1	4.080047	-1.366189	-1.210413
48	6	3.529305	1.331211	0.922255
49	1	2.529911	1.728532	1.119192
50	6	2.908389	0.672866	-1.464459
51	1	2.539033	-0.199466	-2.005221
52	1	3.868541	0.932218	-1.903420
53	6	5.739690	-0.209157	-0.524655
54	1	6.396288	-0.983234	-0.932341
55	1	5.713179	0.602040	-1.254157
56	1	6.196919	0.153438	0.398126
57	6	4.138437	0.919710	2.258692
58	1	4.170520	1.830806	2.864418
59	1	3.553982	0.178739	2.798909
60	1	5.166590	0.568507	2.152330
61	6	1.942243	1.860772	-1.572353
62	1	2.450327	2.730817	-1.992542
63	1	1.113307	1.609555	-2.237805
64	1	1.514652	2.175995	-0.616133
65	6	4.375902	2.454003	0.319051
66	1	4.183962	3.340401	0.931297
67	1	5.442973	2.236623	0.383097
68	1	4.131561	2.716866	-0.708889
69	6	4.540182	-1.973803	0.773942
70	1	3.667706	-2.620515	0.852387
71	1	5.381912	-2.590663	0.443528
72	1	4.778570	-1.587042	1.765023
73	1	0.672911	-0.177418	-1.156355
74	1	0.443419	-2.511341	0.703035

Energies of transition state structures (TSSs) and intermediates (INTs) along the Diels–Alder pathway for chiral and achiral dienophiles:

Calculations of the Diels-Alder step between chiral (BTM-derived) and achiral (Brønsted base-derived) dienophiles showed that only the BTM-bound acylammonium dienophile is likely to undergo cycloaddition (**Table S3**). While several barriers with achiral dienophiles look low enough to appear viable (**Table S3*b***), only the Diels-Alder reaction with chiral (BTM) dienophile is not preceded by a significant endergonic step (overall barriers shown in **Table S3*a***).

All achiral dienophiles may undergo a step-wise Diels-Alder reaction, assuming that the final step is exergonic. If the last step is not exergonic, however, the cycloaddition would likely be reversible. This would mean that formation of the acylammonium ion would control which species (chiral or achiral dienophile) would undergo the Diels-Alder step. As discussed above, all formation of acylammonium salts derived from Brønsted bases are endergonic and have barriers too high to compete with BTM. We conclude then that the Bronsted base does not effectively compete with BTM in the acylammonium formation step nor the Diels-Alder step of this organocascade.

Table 1. Comparison of energy barriers (ΔG) for the DA cycloaddition between BTM-derived and achiral Lewis base-derived, α,β-unsaturated acylammonium salt and siloxy diene.[a]

a. Energies relative to separated reactants.

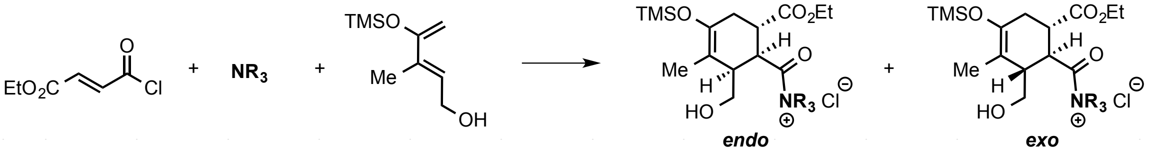

	BTM		Pyridine		2,6–Lutidine		DTBP		Triethylamine		Hünig's base	
	endo	*exo*	*endo*	*exo*	*endo*	*exo*	*endo*	*exo*	*endo*	*exo*	*endo*	*exo*
**TS**	10.7	12.0	24.9, 17.2	25.3	31.0, 25.7	31.1, –	53.9, 44.0	50.8, 44.6	25.1, 24.0	28.1, 22.3	–	34.6, 33.2
**MIN**	–30.7	–35.7	15.6, –16.4	–0.34	38.0, –9.6	23.4, –12.0	40.9, 14.8	41.7, 7.6	21.8, –11.9	21.1, –14.9	–	28.5, –

b. Energies relative to preformed acylammonium salt.

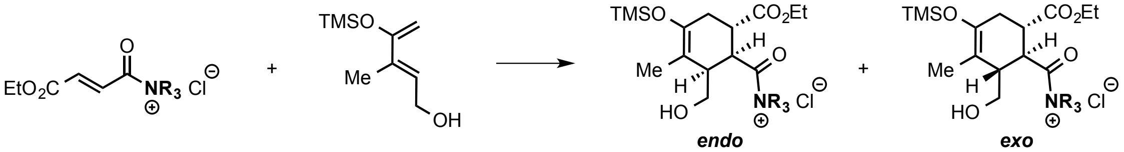

	BTM		Pyridine		2,6–Lutidine		DTBP		Triethylamine		Hünig's base	
	endo	*exo*	*endo*	*exo*	*endo*	*exo*	*endo*	*exo*	*endo*	*exo*	*endo*	*exo*
**TS**	16.6	17.8	12.5, 4.8	13.2	18.4, 13.2	18.6, –	21.8, 12.0	18.7, 12.5	17.6, 16.5	20.6, 14.7	–	17.0, 15.7
**MIN**	–24.9	–29.9	3.2, –28.8	–12.7	25.5, –22.2	10.9, –24.5	8.8, –17.2	9.7, –24.4	14.3, –19.4	13.5, –22.4	–	11.0, –

[a]Energies computed with SMD(DCM)-M06-2X/6-31G(d) and shown in kcal/mol relative to separated reactants.

b. Diels-Alder step of the DAL organocascade

***ii.* Modeling explicit base**

Once we confirmed that Brønsted bases were not competing with the BTM catalyst in acylammonium formation or the Diels-Alder step, we sought another explanation for its effect on yield and selectivity of the DAL organocascade. We postulated that the base could be involved in non-covalent interactions in the Diels-Alder TSS, helping to stabilize one TSS over another. Manual conformational searching of the *endo* and *exo* TSSs with an explicit 2,6-lutidine Brønsted base was carried out to consider this role of the base. We reasoned that the base could hydrogen bond with the terminal alcohol of the diene and exhibit π-stacking interactions with the BTM catalyst. Multiple orientations of the base (displaced, stacked, T-shaped) were considered during the manual conformational search. We chose the lowest energy conformations and orientations of the *endo* and *exo* TSSs found to compare and present here. As expected, the base did indeed exhibit non-covalent interactions with the adduct. We found that it stablilized the *exo* TSS further than the *endo* TSS, leading to a lowering of the energy barrier and a ΔΔG of nearly zero. The computational results supported our hypothesis that the base indeed played a role in the TSS of the Diels-Alder step of the DAL organocascade. Though these results were promising and exciting, it led us to question the origin of diastereoselectivity, discussed in the following section.

Diene:

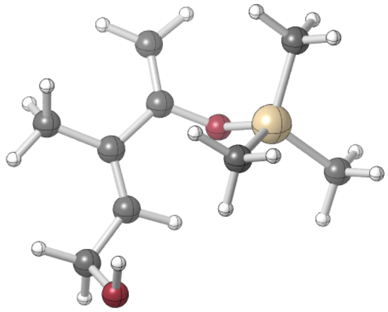

Charge = 0 Multiplicity = 1

HF = -793.4977666 hartrees (-497927.783519166 kcal/mol)

Imaginary Frequencies: none found

Zero-point correction = 0.255956 (Hartree/Particle)

Temperature 298.150 Kelvin. Pressure 1.00000 Atm.

Sum of electronic and thermal Free Energies =

-793.286374 hartrees (-497795.13254874 kcal/mol)

Coordinates (from last standard orientation):

Center Number	Atomic Number	Coordinates (Angstroms) X	Y	Z
1	6	-1.789481	-0.288014	-0.572437
2	6	0.380319	2.656051	-0.177700
3	6	-3.090830	-0.997507	-0.350989
4	6	-0.048458	1.411338	-0.423352
5	6	0.791570	-1.302206	1.391004
6	6	3.117543	0.624855	0.841751
7	6	2.564231	-1.710651	-1.107445
8	6	-1.445877	0.955064	-0.200382
9	6	-2.378693	1.946485	0.443736
10	1	-1.034051	-0.907485	-1.051103
11	1	1.413073	2.925175	-0.374643
12	1	-0.283560	3.425212	0.196776
13	1	-3.831018	-0.372046	0.161086
14	1	-3.524191	-1.299905	-1.310550
15	1	0.046637	-1.982972	0.964440
16	1	1.448027	-1.888470	2.045693
17	1	0.266990	-0.572411	2.018642
18	1	3.697007	1.161466	0.082416
19	1	2.678684	1.363738	1.521178
20	1	3.816673	0.014203	1.425967
21	1	3.238273	-2.390030	-0.573475
22	1	1.786506	-2.316488	-1.585546
23	1	3.140843	-1.217469	-1.897471
24	1	-2.480754	-1.965170	1.218249
25	1	-1.969850	2.302424	1.395567
26	1	-2.512158	2.823853	-0.199200
27	1	-3.365079	1.520991	0.631188
28	8	0.810376	0.473233	-0.934987
29	8	-2.871154	-2.210857	0.364149
30	14	1.810874	-0.464008	0.059455

Ethyl fumaroyl chloride (**5**):

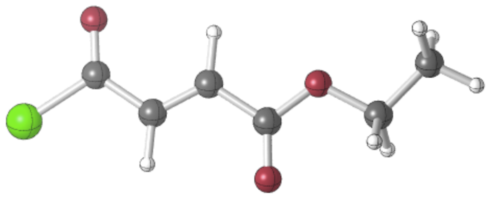

Charge = 0 Multiplicity = 1

HF = -918.5039922 hartrees (-576370.440145422 kcal/mol)

Imaginary Frequencies: none found

Zero-point correction = 0.126267 (Hartree/Particle)

Temperature 298.150 Kelvin. Pressure 1.00000 Atm.

Sum of electronic and thermal Free Energies =

-918.416338 hartrees (-576315.43625838 kcal/mol)

Coordinates (from last standard orientation):

Center Number	Atomic Number	Coordinates (Angstroms) X	Y	Z
1	6	-2.404785	0.535097	-0.020979
2	6	0.008958	0.346013	-0.031898
3	6	-1.169862	-0.277188	-0.025157
4	6	1.268965	-0.453229	-0.032192
5	6	3.616705	-0.317867	-0.008249
6	6	4.675205	0.755901	0.080148
7	1	0.088461	1.428999	-0.034584
8	1	-1.250380	-1.358672	-0.017684
9	1	3.699592	-0.904604	-0.927911
10	1	3.655164	-1.005150	0.841606
11	1	4.570953	1.326879	1.006880
12	1	5.665622	0.292605	0.068215
13	1	4.604344	1.443500	-0.767095
14	8	-2.491100	1.719758	-0.059931
15	8	2.334266	0.343047	-0.005009
16	8	1.311464	-1.661878	-0.054280
17	17	-3.896583	-0.468432	0.059665

(S)-(–)-BTM catalyst:

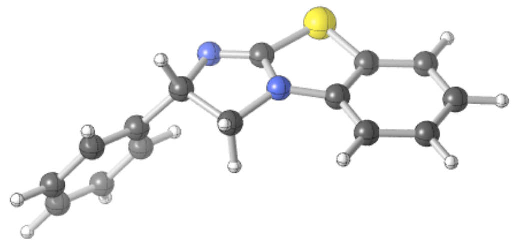

Charge = 0 Multiplicity = 1

HF = -1086.2217193 hartrees (-681614.991077943 kcal/mol)

Imaginary Frequencies: none found

Zero-point correction = 0.238948 (Hartree/Particle)

Temperature   298.150 Kelvin. Pressure   1.00000 Atm.

Sum of electronic and thermal Free Energies =

-1086.024409 hartrees (-681491.17689159 kcal/mol)

Coordinates (from last standard orientation):

Center Number	Atomic Number	Coordinates (Angstroms) X	Y	Z
1	6	-2.658926	-1.872440	0.032577
2	6	-2.123816	-0.600345	-0.141211
3	6	-2.895105	0.543447	0.132450
4	6	-4.207606	0.432725	0.561787
5	6	-4.746737	-0.845244	0.732328
6	6	-3.978352	-1.979555	0.473061
7	1	-2.058599	-2.752062	-0.178240
8	1	-4.800047	1.317910	0.772337
9	1	-5.771715	-0.949580	1.072628
10	1	-4.410212	-2.965542	0.613459
11	6	-0.539268	1.070167	-0.533267
12	16	-1.966889	2.036351	-0.147291
13	7	-0.860156	-0.271737	-0.595974
14	6	0.395774	-1.012047	-0.630557
15	6	1.379397	0.139158	-1.027978
16	7	0.675758	1.406318	-0.721608
17	6	2.713946	0.013122	-0.330895
18	6	3.702254	-0.807909	-0.878940
19	6	2.957188	0.651293	0.886801
20	6	4.914045	-0.995064	-0.218440
21	1	3.520707	-1.300931	-1.831631

22	6	4.171891	0.470832	1.545054
23	1	2.193991	1.298559	1.309570
24	6	5.151970	-0.354415	0.996466
25	1	5.674328	-1.635977	-0.655503
26	1	4.353595	0.977589	2.488602
27	1	6.098468	-0.494531	1.510257
28	1	0.621577	-1.422838	0.361589
29	1	0.374413	-1.817883	-1.366373
30	1	1.544570	0.099252	-2.112384

2,6-lutidine/2,6-dimethylpyridine (DMP):

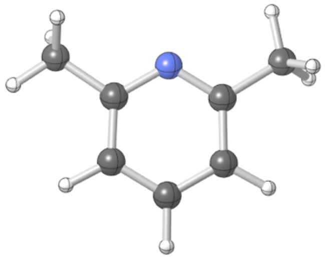

Charge = 0 Multiplicity = 1

HF = -326.7824947 hartrees (-205059.283249197 kcal/mol)

Imaginary Frequencies: none found

Zero-point correction = 0.145307 (Hartree/Particle)

Temperature 298.150 Kelvin. Pressure 1.00000 Atm.

Sum of electronic and thermal Free Energies =

-326.669833 hartrees (-204988.58690583 kcal/mol)

Coordinates (from last standard orientation):

Center Number	Atomic Number	Coordinates (Angstroms) X	Y	Z
1	6	-1.160473	-0.261946	-0.004035
2	6	-1.195166	1.131299	-0.003236
3	6	0.008859	1.831739	0.000842
4	6	1.199720	1.121255	0.003073
5	6	1.149738	-0.278042	0.001039
6	7	-0.006143	-0.949731	-0.002114
7	1	0.013558	2.918072	0.001370
8	1	-2.147267	1.652127	-0.005575
9	1	2.159615	1.628875	0.005136

10	6	2.418847	-1.088539	-0.000521
11	1	3.018921	-0.873332	-0.891088
12	1	3.035787	-0.852185	0.872816
13	1	2.182057	-2.154074	0.013626
14	6	-2.421617	-1.082545	0.002318
15	1	-2.405224	-1.812259	-0.812977
16	1	-2.505201	-1.644612	0.938663
17	1	-3.308700	-0.453813	-0.104057

Chloride anion (Cl^{-}):

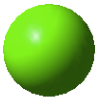

Charge = -1 Multiplicity = 1

HF = -460.31703 hartrees (-288853.5394953 kcal/mol)

Imaginary Frequencies: none found

Zero-point correction = 0.000000 (Hartree/Particle)

Temperature 298.150 Kelvin. Pressure 1.00000 Atm.

Sum of electronic and thermal Free Energies =

-460.332053 hartrees (-288862.96657803 kcal/mol)

Coordinates (from last standard orientation):
atom 1 is isolated, type=Cl

Center Number	Atomic Number	Coordinates (Angstroms) X	Y	Z
1	17	0.000000	0.000000	0.000000

Diels-Alder TSS with explicit DMP (endo)

Reactant:

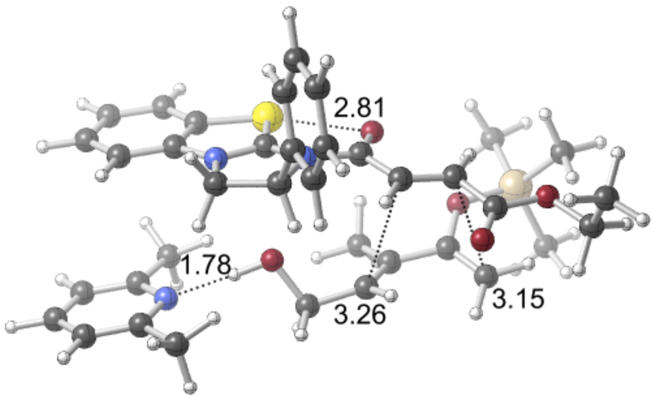

Charge = 1 Multiplicity = 1

HF = -2664.7483368 hartrees (-1672156.22882537 kcal/mol)

Imaginary Frequencies: none found

Zero-point correction = 0.774735 (Hartree/Particle)

Temperature 298.150 Kelvin. Pressure 1.00000 Atm.

Sum of electronic and thermal Free Energies =

-2664.060647 hartrees (-1671724.69659897 kcal/mol)

Coordinates (from last standard orientation):

Center Number	Atomic Number	Coordinates (Angstroms) X	Y	Z
1	6	-4.947260	0.060299	-1.422320
2	6	-3.586680	-0.164400	-1.605819
3	6	-3.109991	-1.243207	-2.361758
4	6	-3.992806	-2.123804	-2.977426
5	6	-5.355397	-1.897877	-2.807393
6	6	-5.824306	-0.825721	-2.036172
7	6	-1.330564	0.129195	-1.421847
8	6	-2.457314	1.794633	-0.284553
9	6	-0.919825	2.028908	-0.150059
10	6	-0.465132	3.361917	-0.696935
11	6	0.039119	4.331329	0.168459
12	6	-0.571394	3.637241	-2.063182
13	6	0.432878	5.573618	-0.326324
14	6	-0.175036	4.875541	-2.556565
15	6	0.326637	5.845723	-1.687482

16	6	0.993747	0.476582	-1.003812
17	6	1.957214	1.243614	-0.191690
18	6	3.249659	0.915440	-0.216379
19	6	0.886025	-0.835053	2.084584
20	6	3.829491	-1.314144	1.935374
21	6	-0.575120	-0.799816	2.452895
22	6	2.914736	-1.821636	1.093150
23	6	5.333687	-4.185581	0.860215
24	6	5.900460	-1.851002	-1.110445
25	6	4.164716	-4.234075	-1.988652
26	6	1.449685	-1.809173	1.351893
27	6	4.192514	1.602023	0.710872
28	6	6.462074	1.801613	1.334876
29	6	6.831350	3.207764	0.910240
30	6	0.666844	-2.924729	0.714975
31	1	-5.296954	0.894473	-0.821595
32	1	-3.632040	-2.958268	-3.570012
33	1	-6.065753	-2.569009	-3.278697
34	1	-6.892900	-0.681209	-1.914353
35	1	-2.953617	2.615626	-0.806061
36	1	-2.936882	1.612419	0.677744
37	1	0.127580	4.111425	1.229950
38	1	-0.959557	2.877791	-2.739351
39	1	0.827453	6.323219	0.352475
40	1	-0.254599	5.084811	-3.618912
41	1	0.639298	6.810862	-2.074220
42	1	-0.620457	1.903942	0.892447
43	1	1.603692	2.028525	0.468943
44	1	1.511533	-0.019402	2.444135
45	1	4.888507	-1.307827	1.696544
46	1	3.527926	-0.905887	2.892577
47	1	3.634792	0.134460	-0.866123
48	1	-0.885414	-1.776225	2.853259
49	1	-0.726673	-0.062125	3.253164
50	1	5.811000	-3.592502	1.645908
51	1	4.528214	-4.768965	1.321300
52	1	6.077888	-4.895259	0.479504
53	1	5.533928	-1.326966	-2.000922
54	1	6.096484	-1.100158	-0.337915
55	1	6.855909	-2.320108	-1.375469
56	1	3.454270	-5.003082	-1.665674
57	1	3.685403	-3.641853	-2.775929
58	1	5.031509	-4.739338	-2.429494
59	1	7.307544	1.118584	1.238578
60	1	6.091533	1.772982	2.362592
61	1	7.641329	3.578984	1.545230

62	1	7.174323	3.216906	-0.128087
63	1	5.977036	3.882124	1.009498
64	1	0.764994	-2.859174	-0.373775
65	1	1.062790	-3.902839	1.012719
66	1	-0.393333	-2.873093	0.968451
67	8	1.268887	-0.483574	-1.697451
68	8	3.223460	-2.410573	-0.090604
69	8	3.850370	2.372254	1.580765
70	8	5.453613	1.244604	0.466189
71	8	-1.362533	-0.469295	1.315466
72	7	-2.527208	0.581881	-1.101003
73	7	-0.342812	0.891461	-0.928501
74	16	-1.338429	-1.287837	-2.387702
75	14	4.675305	-3.151565	-0.555452
76	1	-2.310963	-0.525702	1.602683
77	6	-4.582226	0.388883	2.821385
78	6	-5.961605	0.458491	3.009611
79	6	-6.768708	-0.508718	2.418785
80	6	-6.181049	-1.516053	1.663611
81	6	-4.793243	-1.521733	1.512288
82	7	-4.025035	-0.578174	2.074900
83	1	-7.846614	-0.476149	2.547797
84	1	-6.386813	1.256498	3.610069
85	1	-6.778218	-2.287997	1.188080
86	6	-4.091604	-2.592533	0.724723
87	1	-4.006291	-3.508077	1.321898
88	1	-4.646370	-2.841597	-0.184814
89	1	-3.081968	-2.268113	0.456957
90	6	-3.645213	1.367974	3.474078
91	1	-3.399154	1.032516	4.488393
92	1	-2.707642	1.438494	2.914811
93	1	-4.095293	2.360887	3.553371

Transition state (TS):

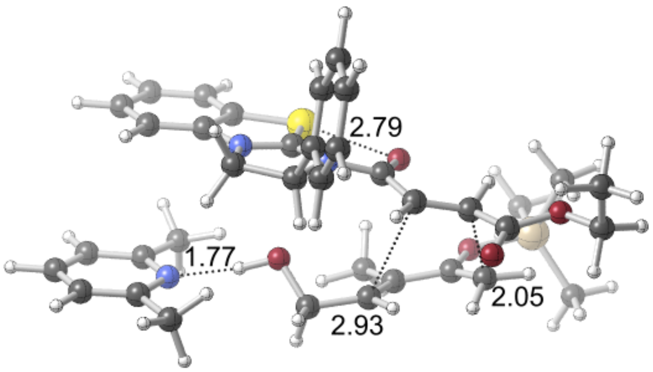

Charge = 1 Multiplicity = 1

HF = -2664.734806 hartrees (-1672147.73811306 kcal/mol)

Imaginary Frequencies: 1 (-410.8986 1/cm)

Zero-point correction = 0.774236 (Hartree/Particle)

Temperature 298.150 Kelvin. Pressure 1.00000 Atm.

Sum of electronic and thermal Free Energies =

-2664.045805 hartrees (-1671715.38309555 kcal/mol)

Coordinates (from last standard orientation):

Center Number	Atomic Number	Coordinates (Angstroms) X	Y	Z
1	6	-4.869586	0.027183	-1.659229
2	6	-3.505442	-0.237164	-1.715511
3	6	-2.999718	-1.372379	-2.362774
4	6	-3.854842	-2.272920	-2.986756
5	6	-5.221603	-2.009018	-2.943108
6	6	-5.720106	-0.877701	-2.285620
7	6	-1.257608	0.018786	-1.366903
8	6	-2.424031	1.772212	-0.426370
9	6	-0.896895	1.950078	-0.154273
10	6	-0.352711	3.270818	-0.649238
11	6	0.007470	4.254099	0.271177
12	6	-0.241695	3.529584	-2.017681
13	6	0.472995	5.491628	-0.170036
14	6	0.227698	4.763255	-2.457617
15	6	0.584552	5.746226	-1.534361
16	6	1.038910	0.306155	-0.743522
17	6	1.941587	1.063925	0.056785

18	6	3.266144	0.640090	0.076760
19	6	0.856570	-0.666469	2.154661
20	6	3.648504	-0.832300	1.458033
21	6	-0.590356	-0.454483	2.521205
22	6	2.733223	-1.834053	1.145134
23	6	5.677173	-3.816544	1.213359
24	6	5.293506	-2.089919	-1.349610
25	6	4.103383	-4.923315	-1.199217
26	6	1.330107	-1.763198	1.511979
27	6	4.268225	1.588087	0.658391
28	6	6.545359	2.226328	0.636024
29	6	6.528388	3.563506	-0.075508
30	6	0.452684	-2.895928	1.055523
31	1	-5.246299	0.903315	-1.139774
32	1	-3.469372	-3.153656	-3.490533
33	1	-5.909725	-2.695439	-3.425299
34	1	-6.790455	-0.700499	-2.261188
35	1	-2.826830	2.576091	-1.046766
36	1	-3.002057	1.680060	0.494500
37	1	-0.072355	4.046286	1.335989
38	1	-0.515935	2.762953	-2.739315
39	1	0.755012	6.251581	0.552339
40	1	0.317188	4.958247	-3.522039
41	1	0.952029	6.707471	-1.880412
42	1	-0.694803	1.827368	0.912944
43	1	1.624945	1.931500	0.621235
44	1	1.547177	0.086603	2.517941
45	1	4.700233	-1.008739	1.253429
46	1	3.456915	-0.218032	2.329307
47	1	3.616817	0.035201	-0.753627
48	1	-0.893266	-1.226562	3.247632
49	1	-0.684522	0.514665	3.031179
50	1	5.929614	-2.944451	1.824736
51	1	5.202209	-4.560262	1.862731
52	1	6.616188	-4.247414	0.846292
53	1	4.563599	-1.776446	-2.104719
54	1	5.642158	-1.198426	-0.816945
55	1	6.154378	-2.513729	-1.881722
56	1	3.621866	-5.668452	-0.556973
57	1	3.412965	-4.678795	-2.013600
58	1	4.993143	-5.385833	-1.641118
59	1	7.459389	1.670463	0.421403
60	1	6.438554	2.341373	1.717194
61	1	7.384343	4.162475	0.249689
62	1	6.597926	3.421855	-1.157743
63	1	5.612875	4.114975	0.153066

64	1	0.408133	-2.911198	-0.038674
65	1	0.870071	-3.855513	1.376592
66	1	-0.560969	-2.793498	1.442603
67	8	1.307289	-0.738031	-1.340995
68	8	3.052966	-2.877249	0.382296
69	8	4.011450	2.426682	1.492902
70	8	5.486515	1.372210	0.153685
71	8	-1.414244	-0.502654	1.371313
72	7	-2.470634	0.510487	-1.164843
73	7	-0.295839	0.787699	-0.860506
74	16	-1.231627	-1.460433	-2.240823
75	14	4.566668	-3.400433	-0.231368
76	1	-2.360343	-0.497664	1.674035
77	6	-4.630738	0.619561	2.684206
78	6	-6.007478	0.819913	2.735468
79	6	-6.842172	-0.090171	2.090473
80	6	-6.282543	-1.169318	1.420635
81	6	-4.891387	-1.304652	1.403542
82	7	-4.097483	-0.417885	2.016345
83	1	-7.919616	0.044425	2.113070
84	1	-6.412493	1.671794	3.271978
85	1	-6.901590	-1.899851	0.908860
86	6	-4.222546	-2.459549	0.712372
87	1	-4.261235	-3.353457	1.346025
88	1	-4.724299	-2.698490	-0.230199
89	1	-3.171004	-2.230953	0.516110
90	6	-3.661216	1.523557	3.393929
91	1	-3.283011	1.029037	4.296290
92	1	-2.797172	1.745976	2.758251
93	1	-4.134648	2.461410	3.693022

Product:

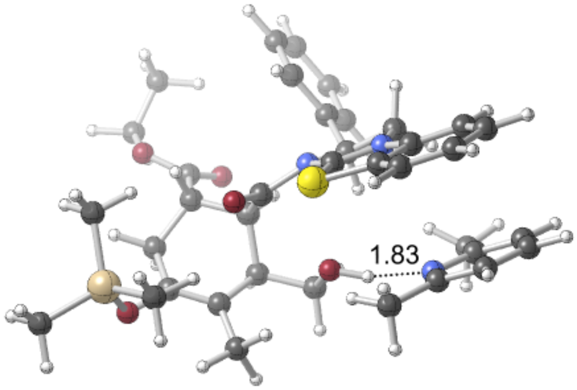

Charge = 1 Multiplicity = 1

HF = -2664.809906 hartrees (-1672194.86411406 kcal/mol)

Imaginary Frequencies: none found

Zero-point correction = 0.780567 (Hartree/Particle)

Temperature 298.150 Kelvin. Pressure 1.00000 Atm.

Sum of electronic and thermal Free Energies =

-2664.111084 hartrees (-1671756.34632084 kcal/mol)

Coordinates (from last standard orientation):

Center Number	Atomic Number	Coordinates (Angstroms) X	Y	Z
1	6	-4.938792	1.071080	-1.593191
2	6	-3.692276	0.457631	-1.537143
3	6	-3.485936	-0.849542	-1.996054
4	6	-4.540707	-1.590824	-2.519318
5	6	-5.791953	-0.984061	-2.574618
6	6	-5.986322	0.327392	-2.121599
7	6	-1.455038	0.196681	-1.145664
8	6	-2.138304	2.301550	-0.507312
9	6	-0.698058	2.034633	0.018842
10	6	0.257400	3.160664	-0.292648
11	6	0.479950	4.139152	0.675821
12	6	0.880098	3.259021	-1.539003
13	6	1.304739	5.225598	0.394510
14	6	1.716261	4.338210	-1.812532
15	6	1.921491	5.327269	-0.850358
16	6	0.769127	-0.084762	-0.350457
17	6	1.744441	0.380581	0.705418
18	6	3.179378	0.276102	0.146509
19	6	1.576300	-0.461575	2.013317
20	6	4.006040	-0.874957	0.786851
21	6	0.180756	-0.293571	2.604386
22	6	3.160629	-2.069209	1.144011
23	6	4.627135	-5.466190	-0.686802
24	6	3.835867	-2.776733	-2.007917
25	6	1.671254	-4.566159	-0.818414
26	6	1.991704	-1.903168	1.773883
27	6	3.942430	1.565030	0.386129
28	6	5.957642	2.698008	-0.124498
29	6	5.491172	3.928581	-0.873540
30	6	1.137734	-3.044217	2.245640
31	1	-5.073356	2.087217	-1.236257
32	1	-4.391462	-2.603789	-2.879486

33	1	-6.630531	-1.538230	-2.983323
34	1	-6.973974	0.772455	-2.183521
35	1	-2.151978	3.040841	-1.313021
36	1	-2.824245	2.596694	0.288546
37	1	0.007801	4.047188	1.651476
38	1	0.712894	2.493621	-2.292953
39	1	1.470466	5.986544	1.150680
40	1	2.205368	4.410224	-2.779374
41	1	2.568926	6.171049	-1.069849
42	1	-0.731944	1.844134	1.096420
43	1	1.552268	1.423909	0.962002
44	1	2.272072	0.006580	2.726819
45	1	4.811216	-1.165991	0.109470
46	1	4.480293	-0.494527	1.703363
47	1	3.150428	0.101945	-0.933249
48	1	0.095540	-0.890505	3.522529
49	1	0.044307	0.762880	2.887539
50	1	5.668649	-5.136132	-0.608843
51	1	4.427312	-6.155123	0.141293
52	1	4.522055	-6.027432	-1.622428
53	1	3.140403	-1.930646	-1.957687
54	1	4.861064	-2.395025	-1.951534
55	1	3.709080	-3.246587	-2.990978
56	1	1.410451	-5.341530	-0.090219
57	1	0.992025	-3.716890	-0.682380
58	1	1.498783	-4.975976	-1.821342
59	1	6.921265	2.344530	-0.494841
60	1	6.033355	2.886449	0.949267
61	1	6.237185	4.722821	-0.771863
62	1	5.366250	3.706646	-1.937735
63	1	4.541186	4.288969	-0.471508
64	1	0.142532	-3.012188	1.789425
65	1	1.610333	-3.999700	2.009448
66	1	0.992502	-3.003382	3.332719
67	8	0.870250	-1.114703	-0.981030
68	8	3.702218	-3.297751	0.858497
69	8	3.626894	2.428955	1.172949
70	8	5.065156	1.585154	-0.337653
71	8	-0.770396	-0.692796	1.639684
72	7	-2.506090	0.991903	-1.046969
73	7	-0.337188	0.751296	-0.645742
74	16	-1.790305	-1.339842	-1.826722
75	14	3.452428	-4.014062	-0.655270
76	1	-1.699093	-0.568364	1.958335
77	6	-4.130169	0.733707	2.245565
78	6	-5.480990	0.947430	1.979829

79	6	-6.209295	-0.061579	1.354291
80	6	-5.572577	-1.247641	1.018067
81	6	-4.213789	-1.391566	1.311620
82	7	-3.516632	-0.411352	1.903408
83	1	-7.262951	0.080112	1.130701
84	1	-5.948321	1.884600	2.264788
85	1	-6.106161	-2.056767	0.529411
86	6	-3.486915	-2.675104	1.019418
87	1	-3.672118	-3.398880	1.821859
88	1	-3.837704	-3.123183	0.084791
89	1	-2.408886	-2.505466	0.963765
90	6	-3.287949	1.749700	2.965445
91	1	-3.072850	1.405325	3.983373
92	1	-2.326183	1.882304	2.459527
93	1	-3.791821	2.716839	3.030803

Diels-Alder TSS with explicit DMP (exo)

Reactant:

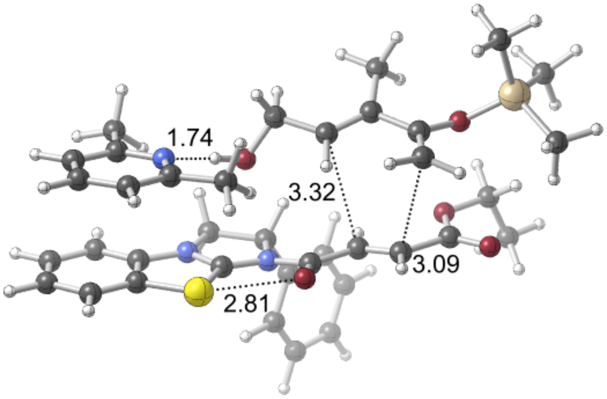

Charge = 1 Multiplicity = 1

HF = -2664.7516116 hartrees (-1672158.28379512 kcal/mol)

Imaginary Frequencies: none found

Zero-point correction = 0.774875 (Hartree/Particle)

Temperature 298.150 Kelvin. Pressure 1.00000 Atm.

Sum of electronic and thermal Free Energies =

-2664.063546 hartrees (-1671726.51575046 kcal/mol)

Coordinates (from last standard orientation):

Center Number	Atomic Number	Coordinates (Angstroms) X	Y	Z
1	6	-5.509026	1.094107	1.104228
2	6	-4.475748	0.889089	0.195094
3	6	-4.699682	0.303659	-1.058389
4	6	-5.975568	-0.101853	-1.436190
5	6	-7.012875	0.105484	-0.533406
6	6	-6.782084	0.693683	0.717507
7	6	-2.371022	0.911450	-0.688010
8	6	-2.360491	1.829329	1.434508
9	6	-0.933595	1.951829	0.817184
10	6	-0.445686	3.374479	0.680101
11	6	0.685160	3.785630	1.384508
12	6	-1.127676	4.286043	-0.130500
13	6	1.135294	5.100916	1.281121
14	6	-0.676094	5.597211	-0.236506
15	6	0.456170	6.005795	0.470042
16	6	-0.091757	0.821086	-1.374067
17	6	2.300071	0.750713	-1.781574
18	6	1.297105	1.115437	-0.978563
19	6	1.080831	-1.726950	0.726279
20	6	3.236496	-2.152552	-1.276773
21	6	2.381004	-1.793905	1.057763
22	6	3.425471	-1.773689	-0.002508
23	6	-0.106331	-1.821692	1.641450
24	6	6.778574	-1.808145	-1.513616
25	6	5.904419	-3.909348	0.572487
26	6	7.232540	-1.273580	1.485709
27	6	2.906558	-1.890633	2.466968
28	6	3.727565	0.999000	-1.433000
29	6	5.214408	1.705613	0.258160
30	6	5.591564	3.132093	-0.085956
31	1	-5.318349	1.549062	2.070920
32	1	-6.153589	-0.562910	-2.402439
33	1	-8.018984	-0.196935	-0.804522
34	1	-7.611997	0.840978	1.400757
35	1	-2.794563	2.802816	1.671220
36	1	-2.365654	1.179076	2.309131
37	1	1.215603	3.071814	2.011125
38	1	-2.013201	3.968174	-0.678582
39	1	2.016802	5.413657	1.832390
40	1	-1.206067	6.301853	-0.870052
41	1	0.807645	7.029571	0.385673

42	1	-0.237296	1.350512	1.403253
43	1	2.123783	0.277643	-2.742840
44	1	1.492745	1.606466	-0.033452
45	1	0.816186	-1.627598	-0.327198
46	1	4.029194	-2.049385	-2.011253
47	1	2.291606	-2.570375	-1.603215
48	1	-0.517034	-2.843168	1.594407
49	1	0.174080	-1.640316	2.685233
50	1	7.865505	-1.954813	-1.529769
51	1	6.341189	-2.506587	-2.235299
52	1	6.565682	-0.789622	-1.857641
53	1	5.589219	-4.083682	1.607334
54	1	5.158620	-4.359659	-0.091423
55	1	6.850470	-4.441703	0.418853
56	1	8.190545	-1.803100	1.547769
57	1	7.448110	-0.228763	1.237591
58	1	6.772476	-1.298828	2.479973
59	1	-1.924638	-1.413788	1.045456
60	1	3.529919	-1.021310	2.699824
61	1	2.107608	-1.957020	3.206257
62	1	3.544920	-2.775221	2.578454
63	1	5.891868	0.986174	-0.205622
64	1	5.195343	1.535585	1.336362
65	1	4.878365	3.836683	0.351867
66	1	6.587645	3.355089	0.307908
67	1	5.608695	3.275442	-1.170111
68	8	-0.417281	0.199628	-2.369949
69	8	4.629702	-1.312953	0.443444
70	8	-1.105728	-0.891957	1.253373
71	8	3.872163	1.424211	-0.178912
72	8	4.632703	0.839318	-2.221598
73	7	-3.128324	1.199350	0.354125
74	7	-1.092713	1.289484	-0.516046
75	16	-3.203452	0.178212	-1.994753
76	14	6.123519	-2.085057	0.218960
77	6	-3.308308	-2.961300	-0.620854
78	6	-4.476289	-3.500986	-1.162191
79	6	-5.654671	-3.416888	-0.428913
80	6	-5.640309	-2.806386	0.821034
81	6	-4.439461	-2.284114	1.297931
82	7	-3.310485	-2.356421	0.575389
83	1	-6.579282	-3.822945	-0.829097
84	1	-4.451033	-3.976385	-2.137512
85	1	-6.542368	-2.727488	1.419198
86	6	-1.983807	-3.042230	-1.327920
87	1	-1.423881	-3.917860	-0.977481

88	1	-2.111370	-3.134470	-2.409496
89	1	-1.383656	-2.152852	-1.112987
90	6	-4.323051	-1.642020	2.651738
91	1	-3.881978	-2.349681	3.363430
92	1	-3.663789	-0.769460	2.606249
93	1	-5.299155	-1.336405	3.035577

Transition state (TS):

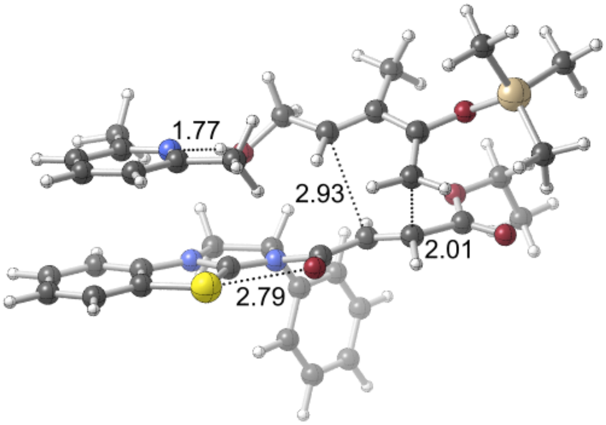

Charge = 1 Multiplicity = 1

HF = -2664.7374882 hartrees (-1672149.42122038 kcal/mol)

Imaginary Frequencies: 1 (-390.1744 1/cm)

Zero-point correction = 0.775625 (Hartree/Particle)

Temperature 298.150 Kelvin. Pressure 1.00000 Atm.

Sum of electronic and thermal Free Energies =

-2664.045355 hartrees (-1671715.10071605 kcal/mol)

Coordinates (from last standard orientation):

Center Number	Atomic Number	Coordinates (Angstroms) X	Y	Z
1	6	-5.620449	0.987076	0.714275
2	6	-4.481942	0.743694	-0.047364
3	6	-4.533815	-0.018074	-1.222726
4	6	-5.735926	-0.550159	-1.674352
5	6	-6.879615	-0.301195	-0.922361
6	6	-6.819991	0.452296	0.256758
7	6	-2.288832	0.776037	-0.697596
8	6	-2.570701	2.027569	1.224979
9	6	-1.088032	2.127154	0.746548

10	6	-0.647427	3.531273	0.399095
11	6	0.390224	4.137811	1.105776
12	6	-1.286740	4.233525	-0.626791
13	6	0.786887	5.437313	0.791774
14	6	-0.889543	5.528114	-0.942080
15	6	0.148546	6.132299	-0.232240
16	6	0.067421	0.740633	-1.145462
17	6	2.464998	0.636315	-1.342327
18	6	1.347397	1.083789	-0.633731
19	6	1.036308	-1.215287	1.158975
20	6	2.775081	-1.341412	-1.154206
21	6	2.378451	-1.344718	1.316387
22	6	3.232954	-1.465687	0.155744
23	6	0.044315	-1.026843	2.278228
24	6	6.051928	-1.989456	-2.099584
25	6	5.241441	-4.196176	-0.086880
26	6	7.273052	-1.976850	0.723820
27	6	3.061191	-1.286219	2.658303
28	6	3.827232	1.101327	-0.936288
29	6	5.161906	1.814150	0.876273
30	6	5.468183	3.263374	0.559168
31	1	-5.564455	1.573517	1.625701
32	1	-5.777433	-1.140786	-2.584034
33	1	-7.831509	-0.700766	-1.256041
34	1	-7.727819	0.628792	0.824505
35	1	-3.068256	2.999690	1.226529
36	1	-2.647522	1.553141	2.203350
37	1	0.891674	3.590742	1.901153
38	1	-2.095508	3.765077	-1.184573
39	1	1.596986	5.903736	1.344211
40	1	-1.388524	6.065665	-1.742539
41	1	0.458406	7.142918	-0.480358
42	1	-0.432731	1.685502	1.499130
43	1	2.359498	0.492267	-2.414484
44	1	1.455348	1.620436	0.298052
45	1	0.583788	-1.361548	0.182425
46	1	3.490996	-1.494373	-1.956008
47	1	1.750756	-1.605761	-1.397658
48	1	-0.074473	-1.982129	2.816431
49	1	0.424848	-0.303382	3.008013
50	1	7.060815	-2.306061	-2.391919
51	1	5.344476	-2.492584	-2.767163
52	1	5.976737	-0.909140	-2.265081
53	1	5.115018	-4.445413	0.972507
54	1	4.286495	-4.378232	-0.593545
55	1	5.979330	-4.886153	-0.511426

56	1	8.126641	-2.610454	0.457622
57	1	7.569905	-0.935386	0.557837
58	1	7.074500	-2.109943	1.792563
59	1	-1.726910	-1.303455	1.447102
60	1	3.723106	-0.415478	2.717130
61	1	2.343320	-1.237202	3.478591
62	1	3.686259	-2.172615	2.807991
63	1	5.915171	1.138006	0.464913
64	1	5.081247	1.648196	1.951944
65	1	4.682497	3.915799	0.951724
66	1	6.417681	3.548569	1.022046
67	1	5.549028	3.415474	-0.520172
68	8	-0.159877	-0.003926	-2.105881
69	8	4.544752	-1.471374	0.418411
70	8	-1.198101	-0.549116	1.817385
71	8	3.872031	1.437226	0.355999
72	8	4.783744	1.118602	-1.679639
73	7	-3.179465	1.166427	0.203992
74	7	-1.066360	1.244028	-0.452363
75	16	-2.936563	-0.178038	-1.971355
76	14	5.782143	-2.421745	-0.301624
77	6	-2.828405	-3.153820	-0.286096
78	6	-3.924695	-3.782551	-0.877378
79	6	-5.177668	-3.638678	-0.290716
80	6	-5.304525	-2.880078	0.867155
81	6	-4.167403	-2.273856	1.400016
82	7	-2.963997	-2.407224	0.819626
83	1	-6.048082	-4.114669	-0.733503
84	1	-3.787692	-4.374470	-1.776550
85	1	-6.266258	-2.748322	1.352080
86	6	-1.440299	-3.267911	-0.851143
87	1	-0.753826	-3.660381	-0.092985
88	1	-1.418993	-3.931331	-1.718904
89	1	-1.068654	-2.284089	-1.162768
90	6	-4.214795	-1.464549	2.665922
91	1	-3.693813	-1.996719	3.469967
92	1	-3.704456	-0.506599	2.530661
93	1	-5.244538	-1.282335	2.981704

Product:

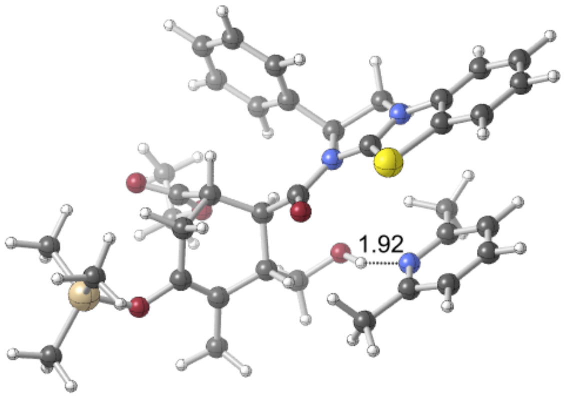

Charge = 1 Multiplicity = 1

HF = -2664.811776 hartrees (-1672196.03755776 kcal/mol)

Imaginary Frequencies: none found

Zero-point correction = 0.782027 (Hartree/Particle)

Temperature 298.150 Kelvin. Pressure 1.00000 Atm.

Sum of electronic and thermal Free Energies =

-2664.110817 hartrees (-1671756.17877567 kcal/mol)

Coordinates (from last standard orientation):

Center Number	Atomic Number	Coordinates (Angstroms) X	Y	Z
1	6	-5.952387	1.703789	-0.506832
2	6	-4.778584	1.001377	-0.763180
3	6	-4.764631	-0.153504	-1.556587
4	6	-5.940041	-0.642708	-2.117735
5	6	-7.116455	0.053804	-1.863871
6	6	-7.121685	1.209410	-1.070275
7	6	-2.576529	0.411206	-0.661810
8	6	-2.946826	2.351224	0.548739
9	6	-1.432100	1.982244	0.622779
10	6	-0.511792	3.004033	-0.003838
11	6	0.518835	3.569355	0.744860
12	6	-0.662996	3.350231	-1.350749
13	6	1.396954	4.475610	0.150071
14	6	0.218162	4.247479	-1.944539
15	6	1.251498	4.809256	-1.193583
16	6	-0.273451	-0.159741	-0.333301
17	6	2.087893	0.673917	-0.449735

18	6	0.975504	0.130140	0.459688
19	6	1.370276	-1.167672	1.225781
20	6	2.836560	-0.466998	-1.155490
21	6	2.809597	-1.611423	1.051785
22	6	3.452312	-1.356650	-0.099179
23	6	0.912028	-0.996555	2.678717
24	6	6.468945	0.006804	-1.688200
25	6	4.940731	-2.217333	-3.222887
26	6	7.113851	-2.948220	-1.182667
27	6	3.402539	-2.482885	2.124673
28	6	3.144522	1.452494	0.330921
29	6	3.987567	2.027422	2.463934
30	6	3.991753	3.540655	2.501737
31	1	-5.942745	2.596168	0.110328
32	1	-5.939378	-1.539499	-2.728650
33	1	-8.047314	-0.307836	-2.287498
34	1	-8.056939	1.729055	-0.890652
35	1	-3.111666	3.314751	0.062154
36	1	-3.425156	2.340490	1.529358
37	1	0.641716	3.291307	1.789360
38	1	-1.467573	2.912470	-1.940467
39	1	2.196110	4.917201	0.736644
40	1	0.100661	4.507533	-2.991991
41	1	1.941708	5.507976	-1.656182
42	1	-1.139959	1.773793	1.655205
43	1	1.677977	1.369666	-1.192355
44	1	0.741726	0.895892	1.197184
45	1	0.752834	-1.980873	0.818473
46	1	3.608750	-0.024778	-1.789010
47	1	2.149084	-1.024147	-1.803541
48	1	1.040033	-1.932630	3.238124
49	1	1.508259	-0.220587	3.172500
50	1	7.262994	0.051207	-2.443801
51	1	5.721196	0.766027	-1.936310
52	1	6.911181	0.284336	-0.724517
53	1	4.404755	-3.166666	-3.108080
54	1	4.230493	-1.470178	-3.591315
55	1	5.700518	-2.361217	-4.000748
56	1	7.905027	-2.938766	-1.941029
57	1	7.572056	-2.705662	-0.217660
58	1	6.720514	-3.968768	-1.121014
59	1	-1.014420	-1.270731	2.342913
60	1	3.481101	-1.946711	3.078753
61	1	2.778739	-3.368265	2.306705
62	1	4.400038	-2.822031	1.840805
63	1	4.932307	1.633558	2.081301

64	1	3.798801	1.604191	3.452270
65	1	3.032514	3.922451	2.865121
66	1	4.775286	3.883103	3.184872
67	1	4.193083	3.952478	1.509971
68	8	-0.413016	-1.094687	-1.098024
69	8	4.687810	-1.884669	-0.325957
70	8	-0.437648	-0.566638	2.721362
71	8	2.916725	1.510672	1.646350
72	8	4.124251	1.922000	-0.202312
73	7	-3.500683	1.279253	-0.287363
74	7	-1.370328	0.693059	-0.137784
75	16	-3.141023	-0.847168	-1.676758
76	14	5.768540	-1.727667	-1.613900
77	6	-2.447897	-3.322497	0.791171
78	6	-3.608315	-3.864771	0.232743
79	6	-4.821520	-3.220372	0.438282
80	6	-4.846345	-2.058589	1.201754
81	6	-3.650638	-1.580079	1.739014
82	7	-2.476218	-2.198320	1.523675
83	1	-5.737827	-3.619013	0.012066
84	1	-3.548658	-4.778050	-0.350419
85	1	-5.774717	-1.527341	1.389813
86	6	-1.109085	-3.967949	0.571301
87	1	-0.497418	-3.904454	1.476089
88	1	-1.221043	-5.018966	0.293442
89	1	-0.573871	-3.457226	-0.237593
90	6	-3.633959	-0.363379	2.623245
91	1	-3.717152	-0.662817	3.674862
92	1	-2.691483	0.181342	2.519857
93	1	-4.475440	0.298122	2.395949

c. **Diels-Alder step of the DAL organocascade**

iii. **Entropy-controlled diastereoselection**

Upon realizing that the base was selectively stabilizing the *exo* TSS of the Diels-Alder step bringing the ΔΔG to nearly zero from 1.3 kcal/mol (without explicit base), we began to question the origin of diastereoselectivity. A closer examination of energies led us to find that the difference in enthalpy (ΔΔH) between the two TSSs was zero. This led us to believe that the diastereoselectivity was controlled by entropy. Many model chemistries and basis sets were used (**Table S4**) to ensure that this was not an artifact of the level of theory initially used. Though the magnitudes and directions slightly change, enthalpy does not seem to control the diastereoselectivity of the asymmetric reaction. Furthermore, experimental results provided evidence for this rare case of entropy-controlled selectivity, as the diastereoselectivity of the reaction was temperature independent.

Table S2. Comparison of free energies (ΔΔG) and enthalpies (ΔΔH) of *endo* and *exo* TSSs from the background and enantioselective Diels-Alder reactions.

a. Comparing functionals **– SMD(DCM) and 6-31G(d) basis set. Energies shown in kcal/mol relative to separated reactants.**

	Free Energy, *ΔΔG* (kcal/mol)			Enthaply, *ΔΔH* (kcal/mol)		
Model	M06–2X	B3LYP–D3[14]	ωB97xD[15]	M06–2X	B3LYP–D3[14]	ωB97xD[15]
Background	1.8	1.8	2.9	1.5	1.8	2.2
$MAD_{background}$	2.2			1.8		
Enantioselective	1.3	–1.1	–0.3	0.0	0.2	0.4
$MAD_{enantioselective}$	0.9			0.2		

b. Comparing basis sets **– SMD(DCM) and M06-2X functional. Energies shown in kcal/mol relative to separted reactants.**

	Free Energy, *ΔΔG* (kcal/mol)		Enthaply, *ΔΔH* (kcal/mol)	
Model	6–31G(d)	6–31+G(d,p)[16]	6–31G(d)	6–31+G(d,p)[16]
Background	1.8	1.2	1.5	1.7
$MAD_{background}$	1.5		1.6	
Enantioselective	1.3	–1.0	0.0	–0.5
$MAD_{enantioselective}$	1.2		0.3	

c. Computed with SMD(DCM). Energies shown in kcal/mol relative to separted reactants.

	Free Energy, *ΔΔG* (kcal/mol)		Enthaply, *ΔΔH* (kcal/mol)	
Model	B3LYP–D3/6–31+G(d,p)	ωB97xD/6–31+G(d,p)	B3LYP–D3/6–31+G(d,p)	ωB97xD/6–31+G(d,p)
Background	1.0	3.2	2.4	2.9
$MAD_{background}$	2.1		2.7	
Enantioselective	0.2	0.8	0.5	0.8
$MAD_{enantioselective}$	0.5		0.7	

c. Lactonization step of DAL organocascade

Initial work towards modeling the lactonization step of the DAL organocascade involved searching for an S_N2-like mechanism where the nucleophilic attack of the terminal alcohol to the carbonyl would regenerate the BTM catalyst and result in a lactone with protonated oxygen. A scan of this S_N2-like mechanism showed that the energy only continues to increase (up to 35 kcal/mol) as the alcohol approaches the carbonyl (**Figure S3**). Unsurprisingly, attempts to optimize the lactone intermediate from such a mechanism were unsuccessful, as the lactone ring would open back up.

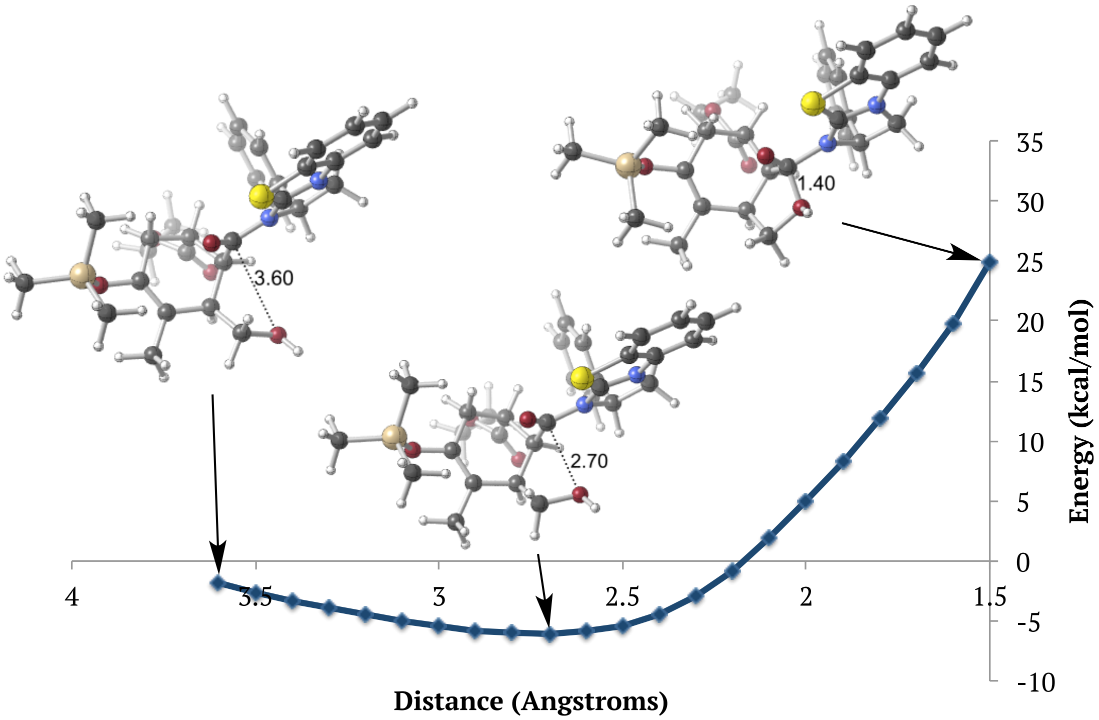

Figure S4. Scan of the O–C bond for γ-lactone formation. Select distances shown in Å.

We then considered an intermediate with a deprotonated alcohol, but protonated carbonyl oxygen to carry out the S_N2-like mechanism; these changes bringing both a better nucleophile and electrophile and because of base and shuttle base in the solution; we reasoned such an intermediate could exist (though likely not for long or without coordination w/ molecules in solution). A scan of this structure (**Figure S4**) showed promise, but every attempt to locate a TSS failed. We were, however, able to find a tetrahedral intermediate (BTM catalyst not kicked off as a leaving group) –4.8 kcal/mol relative to the Diels-Alder product. Based on this result, we chose to deprotonate the alcohol, but leave the carbonyl untouched and ran another scan. Based on the scan, we located another tetrahedral intermediate with an energy of –28.7 kcal/mol relative to the Diels–Alder product and found that there was almost no barrier to reach this intermediate or the final lactone product, making this a very exergonic process if deprotonation of the terminal alcohol occurs. However, based on the pKa's of the base and alcohol (~6 and ~16.0, respectively), deprotonation by base does not seem like a likely path. Therefore, paths *a* and *b* shown in **Scheme S1** were proposed as alternate routes. Though the energy of the intermediates for both pathways relative to the Diels-Alder product seem reasonable, the pathways would lead to scrambling of stereochemistry, which is not experimentally observed. Finally, we reasoned that deprotonation of the terminal alcohol likely occurs in a coordinated fashion with a proton-relay (path *c*).

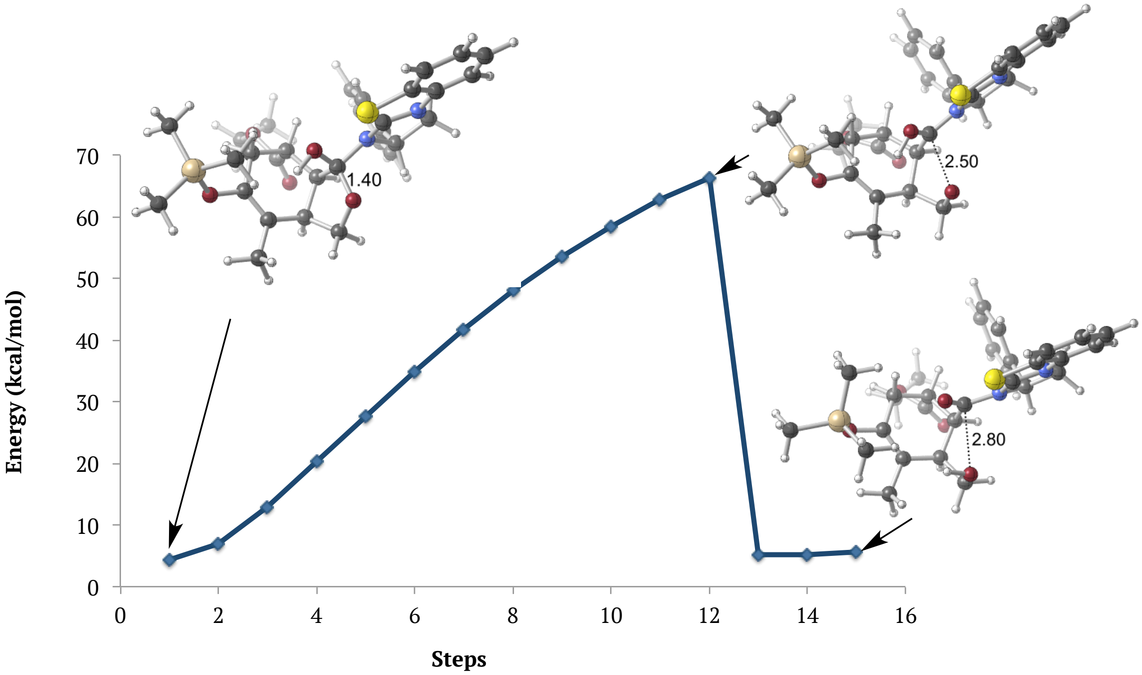

Figure S5. Scan of the O–C bond for γ-lactone formation with a protonated carbonyl (better electrophile) and deprotonated alcohol (better nucleophile). A proton-transfer also occurred during lactonization. Select distances shown in Å.

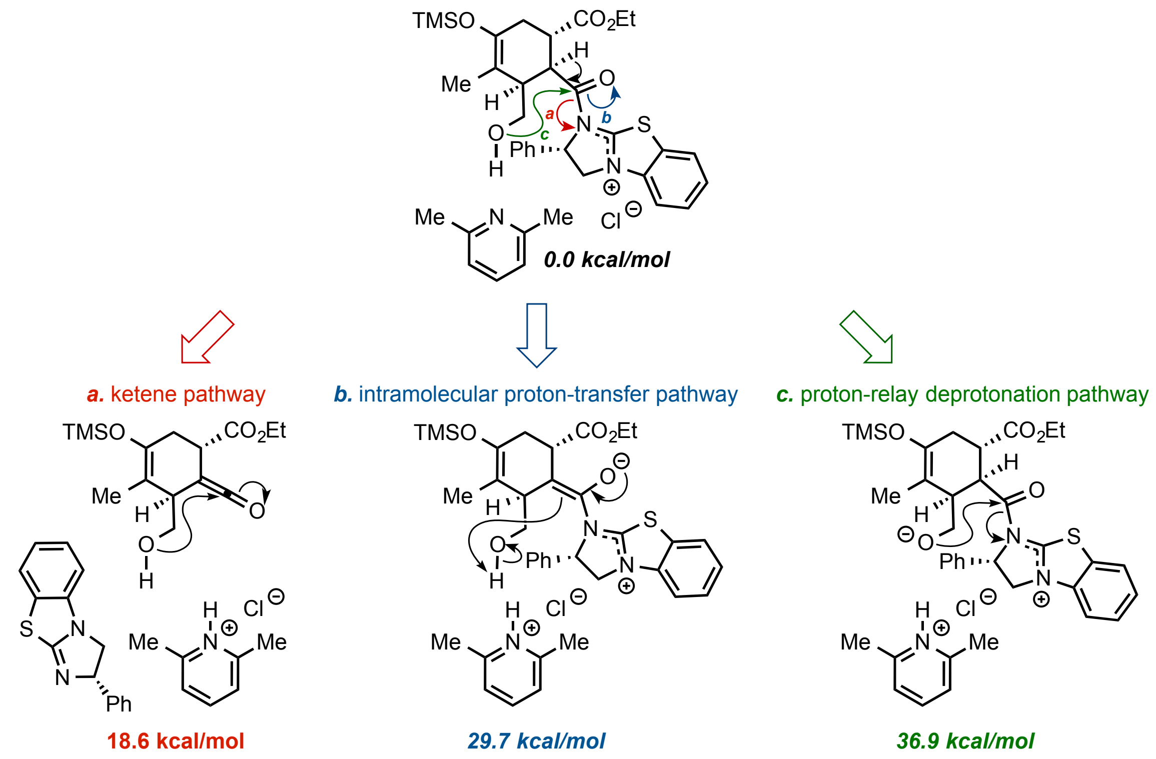

Scheme S1. Possible mechanistic routes for lactonization following the Diels-Alder step.

γ-lactone derived from protonated carbonyl and deprotonated alcohol:

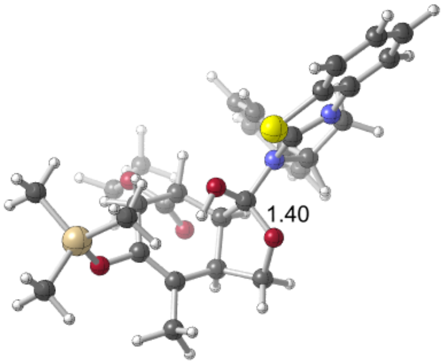

Charge = 1 Multiplicity = 1

HF = -2338.0134158 hartrees (-1467126.79854866 kcal/mol)

Imaginary Frequencies: none found

Zero-point correction = 0.634619 (Hartree/Particle)

Temperature 298.150 Kelvin. Pressure 1.00000 Atm.

Sum of electronic and thermal Free Energies =

-2337.449978 hartrees (-1466773.23569478 kcal/mol)

Coordinates (from last standard orientation):

Center Number	Atomic Number	Coordinates (Angstroms) X	Y	Z
1	6	-6.183318	-0.346167	-0.281287
2	6	-4.845419	-0.720615	-0.230150
3	6	-4.426108	-2.010849	-0.571396
4	6	-5.345558	-2.972949	-0.972796
5	6	-6.686442	-2.604653	-1.028059
6	6	-7.098129	-1.310420	-0.687661
7	6	-2.576563	-0.534104	0.076619
8	6	-3.629518	1.465416	0.540540
9	6	-2.119641	1.562907	0.907052
10	6	-1.469197	2.791786	0.314709
11	6	-1.162718	3.873940	1.139596
12	6	-1.201060	2.864236	-1.054219
13	6	-0.592954	5.025444	0.599212
14	6	-0.617860	4.008614	-1.590528
15	6	-0.317791	5.092593	-0.764454
16	6	-0.306399	-0.282446	0.913840
17	6	0.859519	0.716218	0.963042

18	6	1.619705	0.888446	-0.347654
19	6	1.686815	0.177894	2.134479
20	6	2.480580	-0.335990	-0.723300
21	6	0.561598	-0.253542	3.079448
22	6	2.981616	-1.127225	0.455977
23	6	3.997795	-3.028887	-2.633485
24	6	1.853431	-3.979432	-0.632850
25	6	4.830126	-4.716350	-0.194840
26	6	2.626891	-0.941341	1.737799
27	6	2.477015	2.143251	-0.251552
28	6	3.979673	3.531098	-1.442335
29	6	3.223736	4.834793	-1.590684
30	6	3.216617	-1.768575	2.848321
31	1	-6.488245	0.660574	-0.014304
32	1	-5.028153	-3.975835	-1.239126
33	1	-7.423041	-3.335648	-1.344487
34	1	-8.150595	-1.052435	-0.741150
35	1	-3.884393	2.099834	-0.311917
36	1	-4.280269	1.691566	1.385827
37	1	-1.364936	3.811361	2.206224
38	1	-1.440626	2.020251	-1.697391
39	1	-0.356163	5.865224	1.245224
40	1	-0.401835	4.056502	-2.653691
41	1	0.132541	5.986751	-1.184972
42	1	-1.995243	1.557174	1.995817
43	1	0.462285	1.687061	1.268957
44	1	2.262797	0.985552	2.599589
45	1	1.895560	-1.000633	-1.369926
46	1	3.347619	-0.019696	-1.313366
47	1	0.908074	1.070390	-1.162654
48	1	0.823627	-1.078061	3.745399
49	1	0.208656	0.592997	3.678149
50	1	3.278003	-2.315365	-3.049049
51	1	5.000483	-2.601480	-2.746015
52	1	3.954154	-3.938308	-3.244858
53	1	1.565645	-4.017816	0.425494
54	1	1.154899	-3.325164	-1.167794
55	1	1.720053	-4.988666	-1.040157
56	1	4.774035	-5.643380	-0.776246
57	1	5.861976	-4.351583	-0.246137
58	1	4.611668	-4.959526	0.850920
59	1	4.623211	3.340625	-2.302758
60	1	4.585064	3.520263	-0.532869
61	1	3.936848	5.658224	-1.695998
62	1	2.589019	4.812447	-2.481627
63	1	2.601902	5.023403	-0.712271

64	1	3.404789	-1.146630	3.731426
65	1	2.539247	-2.573580	3.160886
66	1	4.158216	-2.226822	2.539049
67	8	-0.092106	-1.387055	0.086199
68	8	3.907912	-2.079837	0.130366
69	8	2.607516	2.821404	0.742874
70	8	3.082435	2.401566	-1.413501
71	8	-0.515275	-0.698511	2.229875
72	7	-3.758500	0.060785	0.152465
73	7	-1.556255	0.281438	0.383299
74	16	-2.668280	-2.183639	-0.423015
75	14	3.636711	-3.436920	-0.842962
76	1	0.572432	-1.949114	0.528374

c. Lactonization step of DAL organocascade

i. Proton-relay deprotonation pathway

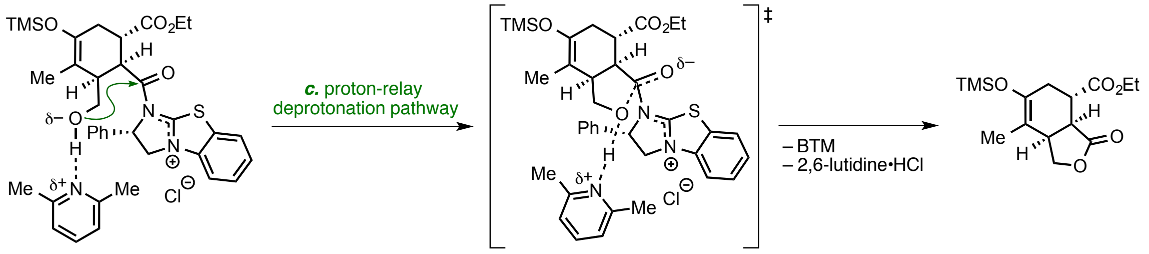

Scheme S2. Proposed lactonization pathway involving a proton-relay deprotonation of the substrate with various molecules (base, shuttle base, solvent) in solution.

Lactonization step after deprotonation of the alcohol moiety (endo):

Reactant:

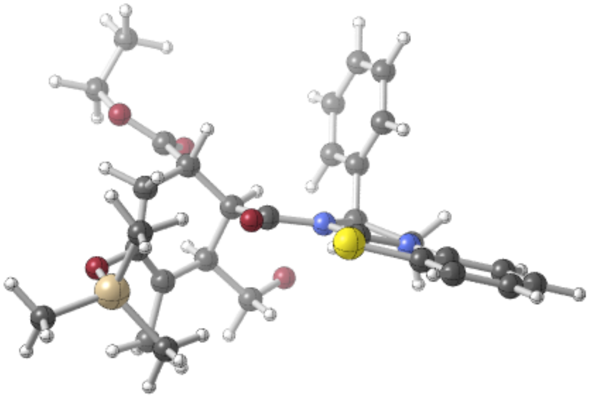

Charge = 0 Multiplicity = 1

HF = -2337.4920644 hartrees (-1466799.64533164 kcal/mol)

Imaginary Frequencies: none found

Zero-point correction = 0.619174 (Hartree/Particle)

Temperature 298.150 Kelvin. Pressure 1.00000 Atm.

Sum of electronic and thermal Free Energies =

-2336.943603 hartrees (-1466455.48031853 kcal/mol)

Coordinates (from last standard orientation):

Center Number	Atomic Number	Coordinates (Angstroms) X	Y	Z
1	6	-6.005652	-0.175905	0.426782
2	6	-4.697223	-0.468682	0.054137
3	6	-4.387497	-1.576384	-0.745330
4	6	-5.390661	-2.426335	-1.199370
5	6	-6.700594	-2.137882	-0.831523
6	6	-7.002277	-1.028821	-0.030128
7	6	-2.423722	-0.276910	-0.098665
8	6	-3.276139	1.369290	1.281353
9	6	-1.763442	1.637634	1.054017
10	6	-1.477981	2.907191	0.284998
11	6	-0.552334	3.811125	0.811991
12	6	-2.114696	3.195053	-0.925623
13	6	-0.265577	4.993705	0.131044
14	6	-1.828615	4.375990	-1.603986
15	6	-0.903627	5.277178	-1.074436

16	6	-0.048213	-0.037485	-0.061928
17	6	1.137983	0.751171	0.437185
18	6	2.212278	0.786505	-0.661075
19	6	1.715957	0.177106	1.754953
20	6	2.856472	-0.591959	-0.897022
21	6	0.632398	0.206070	2.872540
22	6	2.865559	-1.482665	0.314968
23	6	3.870711	-5.412768	-0.508057
24	6	1.861829	-3.749423	-2.148222
25	6	1.217874	-4.437897	0.749893
26	6	2.399140	-1.156208	1.528411
27	6	3.210862	1.893039	-0.345498
28	6	5.271944	2.868999	-0.968425
29	6	4.849445	4.113460	-1.722518
30	6	2.583803	-2.066283	2.714327
31	1	-6.227746	0.685165	1.048649
32	1	-5.156838	-3.287149	-1.817422
33	1	-7.501357	-2.785589	-1.172535
34	1	-8.033937	-0.830089	0.240603
35	1	-3.899020	2.220624	1.001409
36	1	-3.498761	1.066378	2.307544
37	1	-0.046851	3.549318	1.740979
38	1	-2.838499	2.495785	-1.342282
39	1	0.459278	5.690688	0.541073
40	1	-2.326450	4.594501	-2.544150
41	1	-0.680291	6.197890	-1.605526
42	1	-1.157393	1.615915	2.002637
43	1	0.817616	1.765298	0.669691
44	1	2.456406	0.923594	2.079645
45	1	2.321634	-1.106411	-1.700258
46	1	3.889315	-0.472369	-1.240668
47	1	1.755598	1.113167	-1.606180
48	1	-0.130649	-0.578801	2.582923
49	1	1.115605	-0.231514	3.779228
50	1	4.677316	-5.177298	-1.211103
51	1	4.321351	-5.573112	0.477730
52	1	3.414634	-6.357288	-0.826061
53	1	1.074455	-2.988408	-2.113066
54	1	2.617940	-3.432586	-2.875124
55	1	1.411206	-4.678312	-2.519773
56	1	1.591460	-4.787852	1.717713
57	1	0.585619	-3.558775	0.921620
58	1	0.581004	-5.226826	0.331078
59	1	6.193696	2.449133	-1.374722
60	1	5.409603	3.071458	0.096224
61	1	5.631448	4.874799	-1.642936

62	1	4.694576	3.886942	-2.781374
63	1	3.924444	4.522439	-1.307928
64	1	1.626051	-2.422784	3.111963
65	1	3.192802	-2.934670	2.453499
66	1	3.084016	-1.531038	3.531062
67	8	0.014608	-1.042492	-0.748311
68	8	3.477414	-2.695739	0.064649
69	8	3.030762	2.779285	0.459255
70	8	4.299563	1.816388	-1.120595
71	8	0.129803	1.442135	3.076848
72	7	-3.544143	0.233400	0.387335
73	7	-1.339360	0.422569	0.264840
74	16	-2.643150	-1.698914	-1.039993
75	14	2.601458	-4.042532	-0.450215

Transition state 1 (TS1):

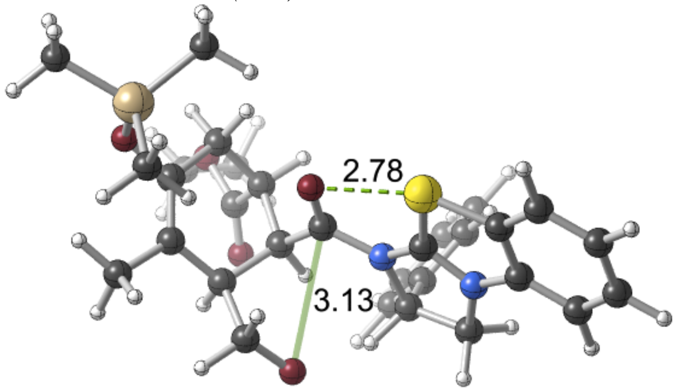

Charge = 0 Multiplicity = 1

HF = -2337.4913425 hartrees (-1466799.19233217 kcal/mol)

Imaginary Frequencies: 1 (-35.2145 1/cm)

Zero-point correction = 0.618310 (Hartree/Particle)

Temperature 298.150 Kelvin. Pressure 1.00000 Atm.

Sum of electronic and thermal Free Energies =

-2336.944226 hartrees (-1466455.87125726 kcal/mol)

Coordinates (from last standard orientation):

Center Number	Atomic Number	Coordinates (Angstroms) X	Y	Z
1	6	-6.146219	-0.404688	0.323547
2	6	-4.815842	-0.705446	0.048141

3	6	-4.448286	-1.872356	-0.633785
4	6	-5.414148	-2.776074	-1.063866
5	6	-6.746214	-2.479069	-0.794526
6	6	-7.105445	-1.311485	-0.108751
7	6	-2.541898	-0.479128	-0.013883
8	6	-3.494051	1.288719	1.132836
9	6	-1.963505	1.538497	0.994851
10	6	-1.621100	2.767572	0.184867
11	6	-0.794078	3.743952	0.741364
12	6	-2.111918	2.940244	-1.112958
13	6	-0.459416	4.882530	0.009806
14	6	-1.779178	4.076454	-1.843998
15	6	-0.951260	5.049585	-1.282383
16	6	-0.171891	-0.181717	0.136614
17	6	0.945132	0.708914	0.619580
18	6	1.918654	0.983492	-0.536323
19	6	1.657393	0.096491	1.843617
20	6	2.681907	-0.279803	-0.954084
21	6	0.569623	-0.294226	2.896976
22	6	3.044623	-1.164751	0.206809
23	6	4.900354	-4.680752	-0.950783
24	6	2.364197	-3.512917	-2.259362
25	6	2.245141	-4.452585	0.632622
26	6	2.626415	-1.001335	1.470058
27	6	2.817628	2.152146	-0.151896
28	6	4.721989	3.408544	-0.768494
29	6	4.116866	4.664935	-1.362369
30	6	3.133406	-1.872255	2.588971
31	1	-6.411443	0.501488	0.858031
32	1	-5.137582	-3.680806	-1.595397
33	1	-7.518393	-3.166727	-1.122881
34	1	-8.152598	-1.108671	0.089742
35	1	-4.090151	2.090698	0.693254
36	1	-3.794925	1.120034	2.168965
37	1	-0.398604	3.595243	1.743728
38	1	-2.757908	2.183520	-1.555548
39	1	0.192072	5.632160	0.448977
40	1	-2.163993	4.203087	-2.851491
41	1	-0.689655	5.934808	-1.854380
42	1	-1.443853	1.507223	1.970441
43	1	0.521943	1.646898	0.963119
44	1	2.212142	0.929324	2.301461
45	1	2.068824	-0.851080	-1.659010
46	1	3.600073	-0.009218	-1.485106
47	1	1.361538	1.357480	-1.408614
48	1	0.110468	-1.254535	2.503640

49	1	1.138005	-0.639185	3.800242
50	1	5.528962	-4.226299	-1.724559
51	1	5.494602	-4.750736	-0.033048
52	1	4.657866	-5.700497	-1.270913
53	1	1.404163	-3.013840	-2.085420
54	1	2.910135	-2.953287	-3.026979
55	1	2.148807	-4.509272	-2.665496
56	1	2.795479	-4.722108	1.539806
57	1	1.437287	-3.765448	0.910975
58	1	1.782670	-5.365115	0.236692
59	1	5.654789	3.141560	-1.268174
60	1	4.906447	3.517923	0.302710
61	1	4.815958	5.498852	-1.247437
62	1	3.916435	4.526488	-2.428774
63	1	3.183523	4.923032	-0.855353
64	1	2.334459	-2.488344	3.018791
65	1	3.931555	-2.534884	2.246218
66	1	3.526111	-1.252551	3.405424
67	8	-0.035630	-1.258640	-0.415075
68	8	3.912677	-2.178567	-0.153172
69	8	2.593691	2.927692	0.750299
70	8	3.863194	2.271063	-0.978923
71	8	-0.296734	0.701158	3.140112
72	7	-3.692653	0.047423	0.371805
73	7	-1.491121	0.283290	0.313694
74	16	-2.688493	-1.988285	-0.823806
75	14	3.346874	-3.680159	-0.670453

Intermediate (INT):

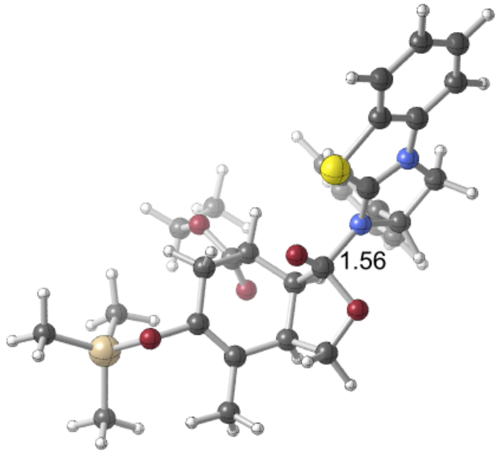

Charge = 0 Multiplicity = 1

HF = -2337.538113 hartrees (-1466828.54128863 kcal/mol)

Imaginary Frequencies: none found

Zero-point correction = 0.620198 (Hartree/Particle)

Temperature 298.150 Kelvin. Pressure 1.00000 Atm.

Sum of electronic and thermal Free Energies =

-2336.989327 hartrees (-1466484.17258577 kcal/mol)

Coordinates (from last standard orientation):

Center Number	Atomic Number	Coordinates (Angstroms) X	Y	Z
1	6	-6.479053	-0.363877	-0.504460
2	6	-5.155782	-0.778067	-0.408817
3	6	-4.698509	-1.941439	-1.043627
4	6	-5.570834	-2.716916	-1.795994
5	6	-6.899702	-2.307360	-1.901403
6	6	-7.346140	-1.148062	-1.261783
7	6	-2.937311	-0.786963	0.215012
8	6	-4.050914	0.975362	1.201955
9	6	-2.514667	1.047156	1.503908
10	6	-1.885110	2.308496	0.949769
11	6	-1.518130	3.341058	1.811291
12	6	-1.700893	2.462306	-0.427418
13	6	-0.973046	4.519978	1.303642
14	6	-1.152954	3.636245	-0.934039
15	6	-0.790376	4.669256	-0.068411
16	6	-0.633981	-0.920126	1.116109
17	6	0.553654	0.069796	1.004597
18	6	1.091710	0.209269	-0.412931
19	6	1.547013	-0.477619	2.049392
20	6	1.915057	-1.013961	-0.854230
21	6	0.634962	-1.383942	2.935791
22	6	2.892026	-1.458681	0.200969
23	6	2.735100	-1.235748	1.510897
24	6	1.943051	1.459395	-0.534469
25	6	3.035161	2.895830	-2.072149
26	6	2.303923	4.215397	-1.930182
27	6	3.695494	-1.741576	2.551950
28	1	-6.814943	0.538861	-0.003994
29	1	-5.227037	-3.620966	-2.289661
30	1	-7.593790	-2.900858	-2.487429
31	1	-8.385570	-0.849807	-1.353918
32	1	-4.415804	1.884795	0.720531

33	1	-4.645111	0.763957	2.094335
34	1	-1.652300	3.217714	2.883322
35	1	-1.979498	1.657332	-1.104277
36	1	-0.685627	5.317724	1.981964
37	1	-1.010904	3.746791	-2.005204
38	1	-0.363286	5.585618	-0.465290
39	1	-2.324538	0.976466	2.577628
40	1	0.224812	1.052670	1.350196
41	1	1.928140	0.350987	2.658113
42	1	1.218135	-1.830609	-1.062762
43	1	2.458953	-0.787245	-1.777924
44	1	0.253649	0.323816	-1.112710
45	1	0.863978	-2.442701	2.750932
46	1	0.739582	-1.181381	4.005529
47	1	3.387012	2.744612	-3.094872
48	1	3.889186	2.843849	-1.392346
49	1	2.975726	5.036586	-2.199453
50	1	1.434826	4.249478	-2.594039
51	1	1.970899	4.362017	-0.899842
52	1	3.159967	-2.291408	3.337057
53	1	4.446063	-2.410666	2.126535
54	1	4.212013	-0.909883	3.049617
55	8	-0.609508	-1.981463	0.402790
56	8	3.933628	-2.215923	-0.295667
57	8	2.400163	2.091377	0.392847
58	8	2.165992	1.776964	-1.820528
59	8	-0.700308	-1.105608	2.560112
60	7	-4.120560	-0.159600	0.281393
61	7	-1.979677	-0.176955	0.861909
62	16	-2.970543	-2.247248	-0.715359
63	14	5.399262	-1.544740	-0.789709
64	6	6.628013	-1.532991	0.625009
65	1	6.763798	-2.528977	1.060660
66	1	7.605925	-1.193792	0.260928
67	1	6.318841	-0.850316	1.424239
68	6	5.115949	0.220927	-1.350680
69	1	4.517656	0.284346	-2.265738
70	1	4.600288	0.781085	-0.560319
71	1	6.074513	0.720258	-1.536511
72	6	6.009495	-2.651106	-2.167326
73	1	6.138989	-3.680141	-1.813513
74	1	5.300847	-2.670493	-3.002341
75	1	6.976311	-2.306504	-2.551558

Transition state 2 (TS2):

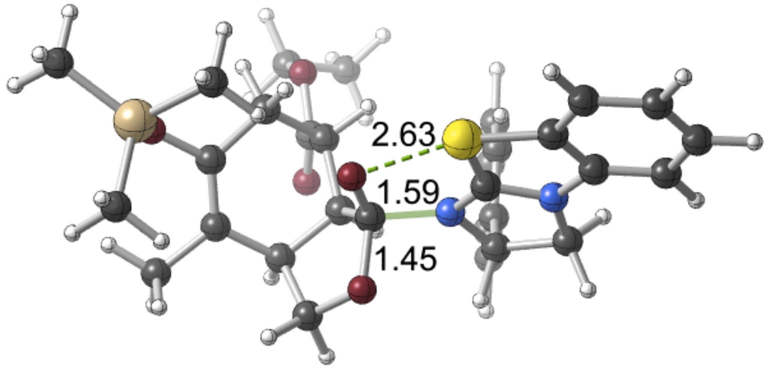

Charge = 0 Multiplicity = 1

HF = -2337.5363814 hartrees (-1466827.45469231 kcal/mol)

Imaginary Frequencies: 1 (-68.3179 1/cm)

Zero-point correction = 0.620500 (Hartree/Particle)

Temperature 298.150 Kelvin. Pressure 1.00000 Atm.

Sum of electronic and thermal Free Energies =

-2336.985617 hartrees (-1466481.84452367 kcal/mol)

Coordinates (from last standard orientation):

Center Number	Atomic Number	Coordinates (Angstroms) X	Y	Z
1	6	-6.047029	-1.154876	-0.169147
2	6	-4.664551	-1.298872	-0.160507
3	6	-4.034156	-2.400960	-0.755623
4	6	-4.788129	-3.386558	-1.378351
5	6	-6.176158	-3.249557	-1.394509
6	6	-6.794740	-2.148760	-0.796334
7	6	-2.445760	-0.823978	0.258523
8	6	-3.816653	0.772848	1.206275
9	6	-2.305361	1.134665	1.409855
10	6	-1.943923	2.463320	0.777634
11	6	-1.661628	3.568087	1.580012
12	6	-1.896421	2.592934	-0.613349
13	6	-1.334279	4.793227	1.000903
14	6	-1.559468	3.812627	-1.192430
15	6	-1.281642	4.916828	-0.385119
16	6	-0.077056	-0.473867	0.983728
17	6	0.838518	0.767416	0.846122

18	6	1.317930	1.009205	-0.574689
19	6	1.942702	0.495820	1.868102
20	6	2.281467	-0.089498	-1.054758
21	6	1.134737	-0.208413	2.986423
22	6	3.209842	-0.581415	0.021440
23	6	5.581987	-3.551170	-1.597362
24	6	2.483277	-3.478864	-1.380241
25	6	4.163145	-3.805451	1.123897
26	6	3.099196	-0.316087	1.330080
27	6	1.951894	2.385272	-0.682049
28	6	2.931866	3.932020	-2.183814
29	6	1.955304	5.090583	-2.186372
30	6	4.155350	-0.711750	2.326865
31	1	-6.518319	-0.295862	0.297584
32	1	-4.309270	-4.243573	-1.842231
33	1	-6.780022	-4.009974	-1.878928
34	1	-7.876345	-2.062252	-0.819058
35	1	-4.353548	1.555947	0.666737
36	1	-4.328142	0.556010	2.147176
37	1	-1.686077	3.464296	2.662162
38	1	-2.113114	1.733364	-1.244472
39	1	-1.110455	5.647151	1.633235
40	1	-1.515843	3.903375	-2.273904
41	1	-1.019389	5.868419	-0.838439
42	1	-2.050045	1.148302	2.473287
43	1	0.296590	1.650582	1.193154
44	1	2.342163	1.439226	2.258554
45	1	1.686916	-0.930858	-1.416253
46	1	2.892265	0.274110	-1.889007
47	1	0.453847	1.022609	-1.252936
48	1	1.691348	-1.029403	3.453220
49	1	0.869231	0.510298	3.773103
50	1	5.534995	-3.132366	-2.608537
51	1	6.521778	-3.222794	-1.139859
52	1	5.617722	-4.642829	-1.688041
53	1	1.642891	-3.063283	-0.808616
54	1	2.412693	-3.115867	-2.411678
55	1	2.372892	-4.570175	-1.402087
56	1	5.075104	-3.555959	1.675854
57	1	3.299764	-3.509060	1.729883
58	1	4.127088	-4.896788	1.014278
59	1	3.408986	3.812010	-3.158279
60	1	3.704544	4.065903	-1.422683
61	1	2.483623	6.013751	-2.444931
62	1	1.165685	4.927405	-2.926251
63	1	1.502293	5.212859	-1.199907

64	1	3.826689	-1.512524	2.999743
65	1	5.067287	-1.047766	1.827966
66	1	4.406245	0.149441	2.960547
67	8	0.099686	-1.489337	0.231172
68	8	4.251818	-1.336722	-0.481117
69	8	2.161737	3.140665	0.241484
70	8	2.265701	2.677567	-1.953596
71	8	-0.062964	-0.716199	2.412766
72	7	-3.725361	-0.439934	0.394853
73	7	-1.582917	0.004001	0.779663
74	16	-2.259514	-2.334048	-0.572537
75	14	4.108480	-3.018133	-0.575594

Product (complex with (S)-(–)-BTM catalyst):

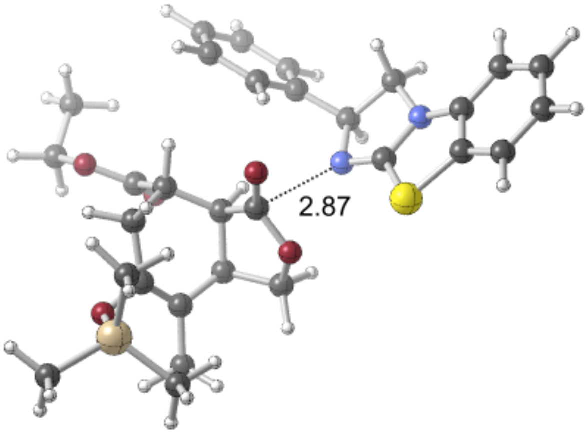

Charge = 0 Multiplicity = 1

HF = -2337.5729067 hartrees (-1466850.37468332 kcal/mol)

Imaginary Frequencies: none found

Zero-point correction = 0.619397 (Hartree/Particle)

Temperature 298.150 Kelvin. Pressure 1.00000 Atm.

Sum of electronic and thermal Free Energies =

-2337.028591 hartrees (-1466508.81113841 kcal/mol)

Coordinates (from last standard orientation):

Center Number	Atomic Number	Coordinates (Angstroms) X	Y	Z
1	6	-6.609039	-0.841569	-0.556118

2	6	-5.303998	-1.043766	-0.119902
3	6	-4.738211	-2.330733	-0.118905
4	6	-5.473271	-3.429588	-0.534392
5	6	-6.786947	-3.229780	-0.966310
6	6	-7.341907	-1.950454	-0.979910
7	6	-3.139739	-0.551608	0.611211
8	6	-4.353835	1.350324	0.239710
9	6	-2.967425	1.616676	0.910741
10	6	-2.196520	2.730496	0.239301
11	6	-2.342236	4.043621	0.691005
12	6	-1.366492	2.475315	-0.856596
13	6	-1.673935	5.090358	0.058635
14	6	-0.686818	3.519996	-1.481200
15	6	-0.843592	4.830315	-1.029540
16	6	0.206694	-0.746820	-0.146330
17	6	0.941705	0.414657	0.496266
18	6	1.901306	1.072330	-0.477515
19	6	1.578622	-0.232437	1.727856
20	6	2.990537	0.084979	-0.909989
21	6	0.501711	-1.284797	2.048016
22	6	3.502137	-0.753725	0.233114
23	6	6.545275	-3.290192	-0.953121
24	6	3.794985	-2.976352	-2.332894
25	6	3.930796	-4.161706	0.465700
26	6	2.921648	-0.864575	1.438224
27	6	2.457696	2.345716	0.131666
28	6	3.875440	4.198055	-0.256431
29	6	2.898419	5.320265	-0.543862
30	6	3.567663	-1.606085	2.576551
31	1	-7.037019	0.156110	-0.561084
32	1	-5.034177	-4.422713	-0.533912
33	1	-7.374564	-4.080185	-1.296303
34	1	-8.361456	-1.809760	-1.325333
35	1	-4.359001	1.634330	-0.820182
36	1	-5.174619	1.847864	0.758857
37	1	-2.979427	4.247152	1.548911
38	1	-1.238057	1.451478	-1.203511
39	1	-1.796032	6.106780	0.421393
40	1	-0.036531	3.310837	-2.326866
41	1	-0.318111	5.643808	-1.521329
42	1	-3.133418	1.892747	1.960366
43	1	0.174155	1.124842	0.820833
44	1	1.665081	0.473813	2.559943
45	1	2.587835	-0.565218	-1.695805
46	1	3.840730	0.615572	-1.350355
47	1	1.344622	1.384249	-1.370518

48	1	0.869189	-2.170616	2.567666
49	1	-0.328799	-0.841752	2.605906
50	1	6.992742	-2.577661	-1.654524
51	1	7.092002	-3.220736	-0.006453
52	1	6.697151	-4.297889	-1.356052
53	1	2.718329	-2.829311	-2.185122
54	1	4.152049	-2.214275	-3.034138
55	1	3.925666	-3.955196	-2.810652
56	1	4.480041	-4.253426	1.408362
57	1	2.897912	-3.872779	0.694457
58	1	3.897481	-5.153044	-0.002753
59	1	4.798717	4.312677	-0.827157
60	1	4.116577	4.137863	0.807397
61	1	3.346836	6.280243	-0.270318
62	1	2.648167	5.345158	-1.608843
63	1	1.979536	5.188918	0.033908
64	1	3.067127	-2.556330	2.797003
65	1	4.616479	-1.822900	2.362186
66	1	3.518323	-1.000943	3.490693
67	8	-0.133479	-0.865919	-1.297642
68	8	4.692722	-1.380032	-0.051781
69	8	2.130719	2.802286	1.204072
70	8	3.339215	2.932249	-0.686927
71	8	0.000479	-1.714757	0.767022
72	7	-4.422303	-0.100148	0.371295
73	7	-2.249027	0.319502	0.891026
74	16	-3.045992	-2.305189	0.430205
75	14	4.728457	-2.939020	-0.707431

Product:

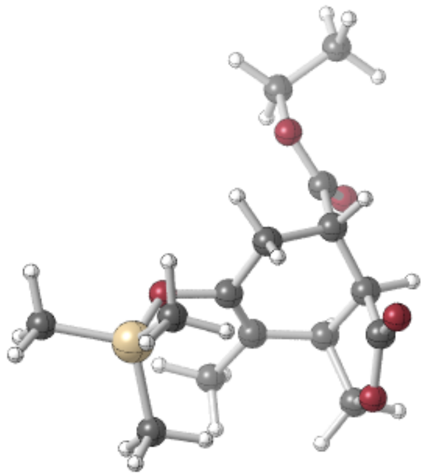

Charge = 0 Multiplicity = 1

HF = -1251.3380755 hartrees (-785227.155757005 kcal/mol)

Imaginary Frequencies: none found

Zero-point correction = 0.379846 (Hartree/Particle)

Temperature   298.150 Kelvin. Pressure   1.00000 Atm.

Sum of electronic and thermal Free Energies =

-1251.011171 hartrees (-785022.01991421 kcal/mol)

Coordinates (from last standard orientation):

Center Number	Atomic Number	Coordinates (Angstroms) X	Y	Z
1	6	-0.234363	2.455680	1.143848
2	6	-1.243317	1.718590	0.277881
3	6	-1.572626	0.347160	0.847760
4	6	-0.553424	1.699640	-1.088502
5	6	-0.297427	-0.494236	0.949640
6	6	0.210481	3.033536	-1.018954
7	6	0.534505	-0.403906	-0.303875
8	6	3.827389	-2.906264	-0.226399
9	6	2.943914	-1.022383	2.049040
10	6	3.890031	0.170564	-0.599543
11	6	0.388856	0.527719	-1.259914
12	6	-2.636004	-0.319091	-0.009290
13	6	-3.888147	-2.299621	-0.328823
14	6	-5.265164	-1.928031	0.181878
15	6	1.149071	0.473524	-2.557191
16	1	-2.144696	2.341722	0.225236
17	1	-1.274922	1.727207	-1.910573
18	1	0.282357	-0.159895	1.818217
19	1	-0.543473	-1.546155	1.121897
20	1	-2.002979	0.458929	1.850309
21	1	1.126691	3.065323	-1.609481
22	1	-0.431029	3.876221	-1.295336
23	1	3.307430	-3.742963	0.252749
24	1	3.829233	-3.083785	-1.307318
25	1	4.867707	-2.917390	0.117705
26	1	2.558360	-0.031386	2.314998
27	1	2.320107	-1.776785	2.541271
28	1	3.954153	-1.102251	2.469413
29	1	4.035622	0.025416	-1.674995
30	1	3.331867	1.102450	-0.448337
31	1	4.878251	0.302406	-0.142245
32	1	-3.661510	-3.350029	-0.139009

33	1	-3.785755	-2.097126	-1.397322
34	1	-6.021671	-2.532901	-0.327184
35	1	-5.338933	-2.115488	1.256911
36	1	-5.478752	-0.873686	-0.011507
37	1	1.947642	1.223171	-2.608621
38	1	1.601344	-0.509118	-2.708579
39	1	0.469642	0.673844	-3.395129
40	8	-0.128124	2.428935	2.343752
41	8	1.445039	-1.424365	-0.429723
42	8	-3.229579	0.220223	-0.916923
43	8	-2.862374	-1.576961	0.381022
44	8	0.585097	3.185256	0.365511
45	14	3.012156	-1.281409	0.193749

Lactonization step after deprotonation of the alcohol moiety (exo):

Reactant:

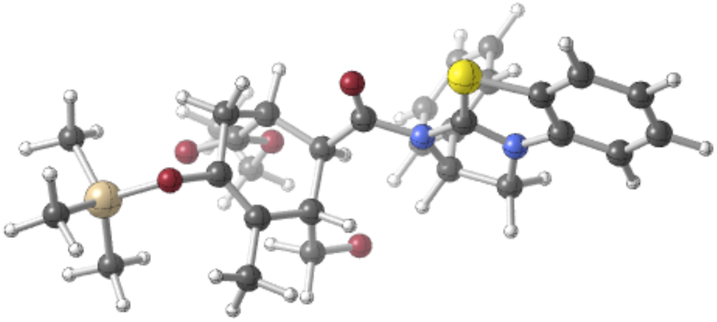

Charge = 0 Multiplicity = 1

HF = -2337.5006474 hartrees (-1466805.03124997 kcal/mol)

Imaginary Frequencies: none found

Zero-point correction = 0.619244 (Hartree/Particle)

Temperature   298.150 Kelvin. Pressure   1.00000 Atm.

Sum of electronic and thermal Free Energies =

-2336.952449 hartrees (-1466461.03127199 kcal/mol)

Coordinates (from last standard orientation):

Center Number	Atomic Number	Coordinates (Angstroms) X	Y	Z
1	6	-6.217158	-1.195309	1.117640
2	6	-5.022971	-1.289583	0.410748

3	6	-4.849840	-2.216115	-0.625916
4	6	-5.878938	-3.080307	-0.983962
5	6	-7.075688	-2.990030	-0.280350
6	6	-7.241183	-2.060803	0.754624
7	6	-2.871293	-0.847482	-0.220847
8	6	-3.449092	0.467361	1.585607
9	6	-2.108278	0.979470	0.994426
10	6	-2.227786	2.303536	0.264979
11	6	-1.300384	3.312774	0.535667
12	6	-3.241507	2.521869	-0.674646
13	6	-1.394050	4.533737	-0.133751
14	6	-3.329362	3.740842	-1.338762
15	6	-2.403665	4.749540	-1.068287
16	6	-0.562460	-0.353395	-0.675737
17	6	1.746914	0.533061	-1.129340
18	6	0.617886	0.360282	-0.102551
19	6	1.069479	-0.366586	1.191701
20	6	2.464492	-0.772482	-1.444379
21	6	2.155409	-1.392476	0.979482
22	6	2.784725	-1.547056	-0.194562
23	6	1.406224	0.732031	2.270720
24	6	6.031598	-1.152944	-1.617121
25	6	6.178086	-3.908136	-0.256277
26	6	5.727309	-1.343770	1.420013
27	6	2.502682	-2.238952	2.176944
28	6	2.736505	1.567823	-0.606056
29	6	2.908235	3.705851	0.389045
30	6	3.580971	4.598378	-0.633702
31	1	-6.331666	-0.470862	1.917207
32	1	-5.752979	-3.802798	-1.783710
33	1	-7.894357	-3.652364	-0.540838
34	1	-8.186261	-2.014279	1.285400
35	1	-4.196132	1.258260	1.665215
36	1	-3.322462	-0.031937	2.549338
37	1	-0.505072	3.099897	1.254265
38	1	-3.969384	1.740995	-0.891718
39	1	-0.671588	5.318337	0.073596
40	1	-4.117265	3.903984	-2.067959
41	1	-2.472402	5.701006	-1.587734
42	1	-1.295156	1.046047	1.744303
43	1	1.319907	0.960920	-2.045658
44	1	0.288032	1.345523	0.241357
45	1	0.195115	-0.889556	1.612453
46	1	3.394958	-0.556297	-1.977746
47	1	1.845570	-1.387029	-2.106540
48	1	1.663611	0.145729	3.189863

49	1	2.392333	1.170307	1.950808
50	1	7.126140	-1.090218	-1.572934
51	1	5.761909	-1.572065	-2.593095
52	1	5.633139	-0.134822	-1.552311
53	1	5.764754	-4.557192	0.523683
54	1	6.009651	-4.395141	-1.223207
55	1	7.261127	-3.844718	-0.100095
56	1	6.780903	-1.044170	1.475696
57	1	5.119190	-0.435183	1.502375
58	1	5.512704	-1.983859	2.281999
59	1	3.099900	-1.681978	2.908836
60	1	1.590310	-2.561946	2.694160
61	1	3.067993	-3.128264	1.886579
62	1	3.638902	3.217063	1.038453
63	1	2.200086	4.261087	1.007163
64	1	2.837192	5.053268	-1.294777
65	1	4.127230	5.398777	-0.125349
66	1	4.288359	4.025238	-1.239111
67	8	-0.581652	-1.142100	-1.600585
68	8	3.745639	-2.517946	-0.375652
69	8	0.415131	1.620505	2.439210
70	8	2.113168	2.695301	-0.253093
71	8	3.936158	1.412712	-0.532177
72	7	-3.869435	-0.532571	0.589955
73	7	-1.775899	-0.116094	0.011528
74	16	-3.230274	-2.098385	-1.342318
75	14	5.396337	-2.210821	-0.206213

Transition state 1 (TS1):

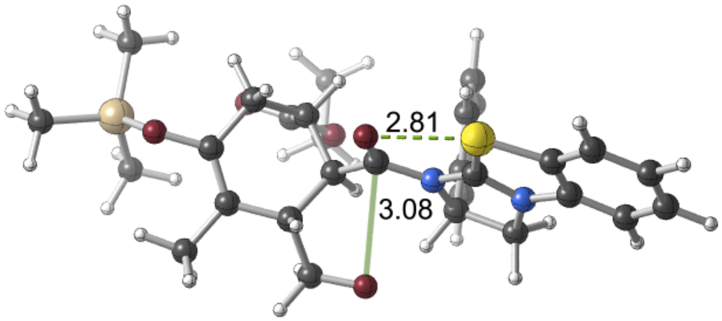

Charge = 0 Multiplicity = 1

HF = -2337.488541 hartrees (-1466797.43436291 kcal/mol)

Imaginary Frequencies: 1 (-29.8405 1/cm)

Zero-point correction = 0.618136 (Hartree/Particle)

Temperature 298.150 Kelvin. Pressure 1.00000 Atm.

Sum of electronic and thermal Free Energies =

-2336.939655 hartrees (-1466453.00290905 kcal/mol)

Coordinates (from last standard orientation):

Center Number	Atomic Number	Coordinates (Angstroms) X	Y	Z
1	6	-6.635519	-0.204335	0.538661
2	6	-5.403450	-0.659264	0.078131
3	6	-5.304287	-1.702411	-0.851899
4	6	-6.445561	-2.317307	-1.355153
5	6	-7.679647	-1.862727	-0.902045
6	6	-7.771610	-0.822732	0.031990
7	6	-3.137028	-0.829928	-0.156373
8	6	-3.649492	0.755318	1.440928
9	6	-2.127186	0.816958	1.123413
10	6	-1.723613	2.105360	0.443416
11	6	-1.368661	3.189507	1.249916
12	6	-1.726064	2.255741	-0.944258
13	6	-1.037326	4.413959	0.676872
14	6	-1.381828	3.477681	-1.518489
15	6	-1.042521	4.560379	-0.709993
16	6	-0.743915	-0.974428	-0.172949
17	6	1.447203	-0.023779	-0.863839
18	6	0.544843	-0.395972	0.338328
19	6	1.178898	-1.399732	1.353857
20	6	2.322721	-1.188154	-1.314880
21	6	2.635407	-1.731482	1.095350
22	6	3.133875	-1.686830	-0.147670
23	6	0.817266	-0.849579	2.764158
24	6	5.956169	-0.336528	-2.147451
25	6	7.159725	-2.547146	-0.439350
26	6	5.902556	-0.111531	0.965000
27	6	3.436309	-2.269812	2.250701
28	6	2.328517	1.172862	-0.525703
29	6	2.367087	3.380936	0.311177
30	6	2.659646	4.207064	-0.924627
31	1	-6.694009	0.600917	1.263725
32	1	-6.373864	-3.125165	-2.076288
33	1	-8.585650	-2.323804	-1.280832
34	1	-8.748273	-0.491946	0.369143
35	1	-4.140155	1.721842	1.319495
36	1	-3.855175	0.345845	2.432977
37	1	-1.350711	3.067579	2.330485

38	1	-1.989063	1.417664	-1.585296
39	1	-0.766014	5.252388	1.312021
40	1	-1.383783	3.584683	-2.599314
41	1	-0.780503	5.514246	-1.158780
42	1	-1.507110	0.584490	2.014953
43	1	0.811007	0.320541	-1.691383
44	1	0.318534	0.516644	0.888529
45	1	0.622214	-2.347538	1.278035
46	1	2.996935	-0.864779	-2.113176
47	1	1.683340	-1.983551	-1.716332
48	1	1.244177	-1.569228	3.507368
49	1	1.455981	0.076189	2.886262
50	1	6.955565	0.106829	-2.240407
51	1	5.822402	-1.023670	-2.991331
52	1	5.218666	0.465872	-2.229788
53	1	7.114423	-3.114222	0.497260
54	1	7.079374	-3.260391	-1.267398
55	1	8.149857	-2.079990	-0.499934
56	1	6.622020	0.696838	0.788630
57	1	4.928549	0.347389	1.162549
58	1	6.220076	-0.654714	1.862077
59	1	3.594899	-1.507431	3.023120
60	1	2.912150	-3.103731	2.734740
61	1	4.412698	-2.630449	1.917905
62	1	3.286999	3.092004	0.826300
63	1	1.719224	3.916051	1.006915
64	1	1.732248	4.448751	-1.452037
65	1	3.146857	5.142801	-0.633632
66	1	3.324426	3.667516	-1.603795
67	8	-0.825081	-1.926113	-0.928619
68	8	4.386053	-2.156961	-0.458897
69	8	-0.509371	-0.661282	2.885615
70	8	1.627190	2.189410	-0.017821
71	8	3.522505	1.224613	-0.727962
72	7	-4.139910	-0.196297	0.431227
73	7	-1.943299	-0.356969	0.222377
74	16	-3.613942	-2.079621	-1.233093
75	14	5.808699	-1.253538	-0.519310

Transition state 2 (TS2):

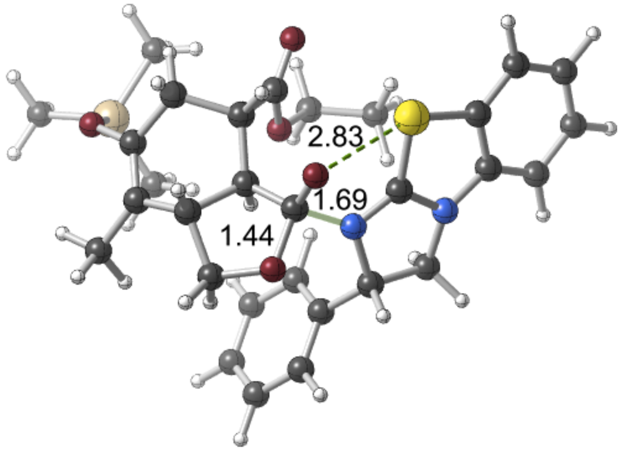

Charge = 0 Multiplicity = 1

HF = -2337.5310651 hartrees (-1466824.1186609 kcal/mol)

Imaginary Frequencies: 1 (-59.0535 1/cm)

Zero-point correction = 0.619255 (Hartree/Particle)

Temperature 298.150 Kelvin. Pressure 1.00000 Atm.

Sum of electronic and thermal Free Energies =

-2336.979383 hartrees (-1466477.93262633 kcal/mol)

Coordinates (from last standard orientation):

Center Number	Atomic Number	Coordinates (Angstroms) X	Y	Z
1	6	-5.355866	-0.187356	-1.496003
2	6	-4.399229	-0.141008	-0.488817
3	6	-4.311952	-1.154943	0.479300
4	6	-5.193284	-2.225801	0.464069
5	6	-6.154552	-2.278088	-0.545913
6	6	-6.231598	-1.272279	-1.510983
7	6	-2.545465	0.538721	0.705755
8	6	-2.873855	1.901999	-1.108336
9	6	-1.726078	2.432508	-0.193246
10	6	-0.482483	2.835334	-0.946680
11	6	-0.013906	4.146109	-0.866305
12	6	0.205400	1.908893	-1.735131
13	6	1.129670	4.531169	-1.564757
14	6	1.347721	2.290650	-2.433566
15	6	1.812252	3.603982	-2.349100

16	6	-0.414440	1.131034	2.028229
17	6	0.803632	-1.140488	1.656957
18	6	0.773509	0.360856	1.426122
19	6	1.957320	1.080910	2.076103
20	6	2.266726	-1.619959	1.943547
21	6	3.225300	0.602178	1.411711
22	6	3.318265	-0.732483	1.313587
23	6	1.489188	2.523140	1.897662
24	6	3.248915	-3.466437	-0.997311
25	6	6.033006	-2.228750	-1.401692
26	6	3.479949	-0.541087	-1.918714
27	6	4.220255	1.568641	0.840262
28	6	0.237035	-1.963982	0.517487
29	6	-0.169386	-2.071614	-1.819030
30	6	-1.670760	-1.888414	-1.920832
31	1	-5.411812	0.598624	-2.242644
32	1	-5.126531	-3.010780	1.211358
33	1	-6.846593	-3.113163	-0.579067
34	1	-6.984725	-1.333489	-2.290285
35	1	-2.492423	1.483005	-2.046938
36	1	-3.620156	2.667685	-1.322126
37	1	-0.541904	4.865209	-0.244953
38	1	-0.146472	0.879209	-1.783188
39	1	1.487364	5.554022	-1.493562
40	1	1.875857	1.563861	-3.043761
41	1	2.702677	3.900704	-2.895441
42	1	-2.083226	3.289715	0.387573
43	1	0.187948	-1.377574	2.530005
44	1	0.845702	0.583881	0.359203
45	1	1.962163	0.848927	3.153030
46	1	2.408074	-2.657962	1.628859
47	1	2.400438	-1.605193	3.032163
48	1	1.930735	3.226218	2.610514
49	1	1.698941	2.875447	0.877072
50	1	3.175959	-3.799422	-2.040193
51	1	3.714336	-4.275713	-0.422775
52	1	2.228384	-3.338506	-0.620073
53	1	6.497353	-2.973459	-0.745697
54	1	6.080964	-2.610442	-2.427867
55	1	6.634965	-1.314606	-1.353432
56	1	3.315434	-0.882647	-2.948084
57	1	2.510321	-0.259558	-1.491166
58	1	4.111339	0.354016	-1.955148
59	1	3.792255	2.132219	0.000588
60	1	4.531407	2.304767	1.592022
61	1	5.111881	1.047970	0.481046

62	1	0.101889	-3.127165	-1.738767
63	1	0.350020	-1.632387	-2.674297
64	1	-1.923903	-0.822171	-1.958865
65	1	-2.047858	-2.362814	-2.831977
66	1	-2.171337	-2.344583	-1.061563
67	8	-1.041880	0.658889	3.007417
68	8	4.377891	-1.366659	0.709413
69	8	0.077374	2.485874	2.126820
70	8	0.344042	-1.360325	-0.679487
71	8	-0.212494	-3.080705	0.651195
72	7	-3.428282	0.830867	-0.277852
73	7	-1.512766	1.315245	0.759470
74	16	-2.988187	-0.862007	1.635491
75	14	4.267691	-1.894408	-0.892848

Product (complex with (S)-(–)-BTM catalyst):

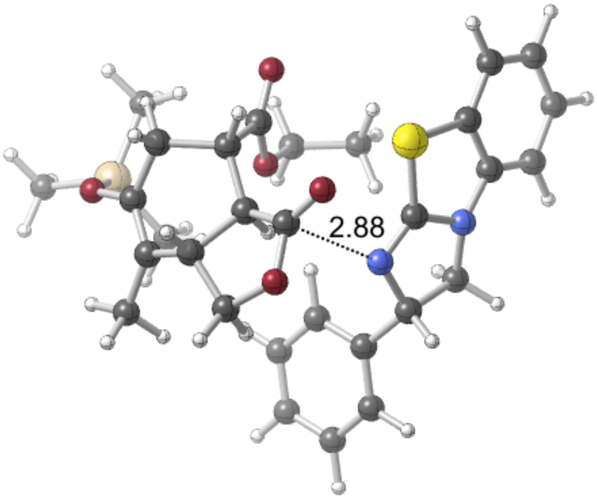

Charge = 0 Multiplicity = 1

HF = -2337.5589342 hartrees (-1466841.60679984 kcal/mol)

Imaginary Frequencies: none found

Zero-point correction = 0.619298 (Hartree/Particle)

Temperature 298.150 Kelvin. Pressure 1.00000 Atm.

Sum of electronic and thermal Free Energies =

-2337.010983 hartrees (-1466497.76194233 kcal/mol)

Coordinates (from last standard orientation):

Center Number	Atomic Number	Coordinates (Angstroms) X	Y	Z
1	6	-5.290997	-0.475136	-1.599859
2	6	-4.371365	-0.365070	-0.562492
3	6	-4.101028	-1.459438	0.278038
4	6	-4.758145	-2.667053	0.103606
5	6	-5.686765	-2.778837	-0.934847
6	6	-5.943770	-1.696267	-1.775323
7	6	-2.652572	0.518808	0.748896
8	6	-3.236038	1.932728	-0.953710
9	6	-2.166444	2.531280	0.017607
10	6	-0.996009	3.141962	-0.716659
11	6	-0.945979	4.520785	-0.926080
12	6	0.017188	2.335371	-1.245798
13	6	0.093874	5.090665	-1.659026
14	6	1.055443	2.903531	-1.981068
15	6	1.096309	4.282507	-2.188904
16	6	-0.041396	0.434110	3.005355
17	6	1.143503	-1.548336	1.747075
18	6	0.930818	-0.051874	1.958380
19	6	2.193409	0.704190	2.389462
20	6	2.677840	-1.854183	1.698209
21	6	3.277831	0.528448	1.363182
22	6	3.487547	-0.756535	1.037187
23	6	1.559938	2.053851	2.697758
24	6	3.336726	-3.114000	-1.648182
25	6	5.958611	-1.615574	-2.164059
26	6	3.290671	-0.048101	-2.191475
27	6	4.014404	1.699810	0.787290
28	6	0.524952	-2.088274	0.468211
29	6	0.097562	-1.660639	-1.832769
30	6	-1.404552	-1.483932	-1.902664
31	1	-5.491371	0.372415	-2.248179
32	1	-4.543807	-3.512342	0.750753
33	1	-6.205092	-3.719830	-1.087462
34	1	-6.664477	-1.799940	-2.580513
35	1	-2.801358	1.644350	-1.920629
36	1	-4.076391	2.609193	-1.119520
37	1	-1.727765	5.152406	-0.510386
38	1	-0.010550	1.259477	-1.075724
39	1	0.120865	6.165478	-1.812857
40	1	1.833439	2.268460	-2.394983
41	1	1.907659	4.723473	-2.760578

42	1	-2.640779	3.310100	0.627565
43	1	0.697186	-2.128674	2.559362
44	1	0.595569	0.417426	1.024831
45	1	2.525705	0.286971	3.354518
46	1	2.861096	-2.810656	1.200147
47	1	3.014226	-1.971945	2.736320
48	1	2.126436	2.688347	3.380424
49	1	1.303307	2.613216	1.790033
50	1	3.111656	-3.303644	-2.704840
51	1	3.966733	-3.937752	-1.292027
52	1	2.393831	-3.160582	-1.093377
53	1	6.527070	-2.414403	-1.675123
54	1	5.922100	-1.839748	-3.236315
55	1	6.511874	-0.678863	-2.035814
56	1	2.947176	-0.275428	-3.207560
57	1	2.415962	0.183493	-1.573139
58	1	3.923041	0.845667	-2.242143
59	1	3.353070	2.317647	0.165147
60	1	4.406827	2.349306	1.579331
61	1	4.852896	1.369596	0.168570
62	1	0.382885	-2.702595	-1.996382
63	1	0.611733	-1.028661	-2.560385
64	1	-1.677627	-0.446829	-1.677469
65	1	-1.759315	-1.721767	-2.910389
66	1	-1.905142	-2.148367	-1.193954
67	8	-0.979496	-0.126273	3.513555
68	8	4.486670	-1.155201	0.189136
69	8	0.335252	1.679251	3.375445
70	8	0.609217	-1.224345	-0.557144
71	8	0.087963	-3.210634	0.354977
72	7	-3.629687	0.746703	-0.203762
73	7	-1.770363	1.425427	0.920639
74	16	-2.851980	-1.076277	1.484364
75	14	4.238365	-1.482190	-1.450502

Product:

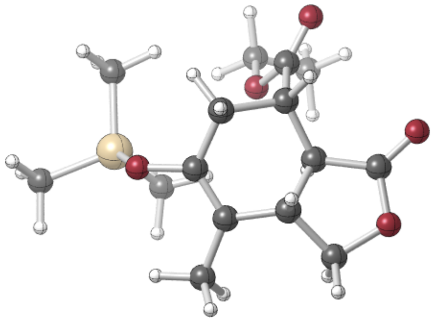

Charge = 0 Multiplicity = 1

HF = -1251.322997 hartrees (-785217.69384747 kcal/mol)

Imaginary Frequencies: none found

Zero-point correction = 0.378570 (Hartree/Particle)

Temperature 298.150 Kelvin. Pressure 1.00000 Atm.

Sum of electronic and thermal Free Energies =

-1250.998262 hartrees (-785013.91938762 kcal/mol)

Coordinates (from last standard orientation):

Center Number	Atomic Number	Coordinates (Angstroms) X	Y	Z
1	6	-3.397637	-0.358907	0.071827
2	6	-1.295353	0.468737	-1.253636
3	6	-1.901247	-0.356114	-0.119625
4	6	-1.601519	-1.850364	-0.233421
5	6	-0.023104	-0.253429	-1.801382
6	6	-0.117932	-2.064382	-0.090841
7	6	0.622114	-1.228921	-0.835023
8	6	-2.578021	-2.381778	0.810545
9	6	3.198843	1.371047	-0.834593
10	6	4.637091	-1.175436	0.072133
11	6	2.296179	-0.078821	1.759212
12	6	0.432969	-3.110576	0.831132
13	6	-0.942976	1.859298	-0.769807
14	6	0.404621	3.055377	0.778626
15	6	-0.584666	3.499520	1.836006
16	1	-2.023051	0.596053	-2.058266
17	1	-1.476249	-0.026531	0.840293
18	1	-1.954121	-2.195942	-1.218521

19	1	0.721426	0.477957	-2.129438
20	1	-0.324650	-0.808493	-2.698350
21	1	-2.899833	-3.412155	0.656661
22	1	-2.201838	-2.259307	1.832339
23	1	3.863784	2.026085	-0.258244
24	1	3.646027	1.233523	-1.825901
25	1	2.244848	1.891422	-0.963286
26	1	4.540204	-2.153866	0.555599
27	1	5.024642	-1.335441	-0.940140
28	1	5.386336	-0.599457	0.627572
29	1	2.877463	0.657864	2.326666
30	1	1.254969	0.259543	1.730848
31	1	2.335773	-1.024835	2.311102
32	1	0.213484	-2.878726	1.881143
33	1	-0.012233	-4.091264	0.622180
34	1	1.516838	-3.197725	0.720450
35	1	0.511165	3.799655	-0.014147
36	1	1.387174	2.846894	1.208376
37	1	-0.688729	2.733475	2.610352
38	1	-0.232177	4.421937	2.306958
39	1	-1.565387	3.690815	1.392165
40	8	-4.206441	0.491226	-0.191554
41	8	1.988910	-1.290685	-0.868032
42	8	-3.743685	-1.536892	0.643667
43	8	-0.005398	1.805212	0.191918
44	8	-1.412313	2.888719	-1.192522
45	14	2.999241	-0.277402	0.034919

c. Lactonization step of DAL organocascade

ii. Ketene pathway

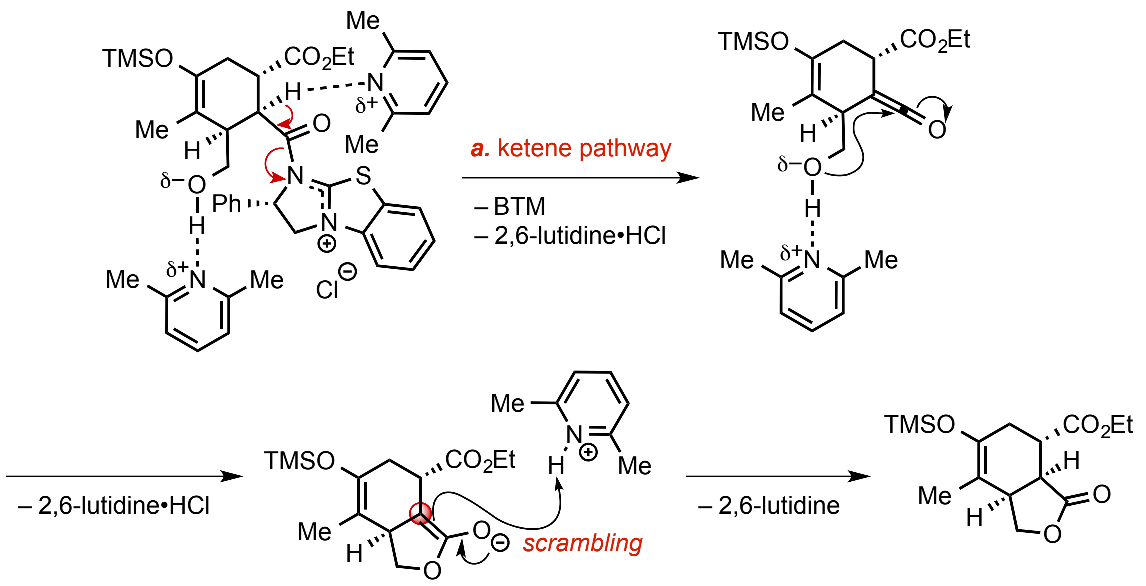

Scheme S3. A ketene pathway was investigated for lactonization; however, this pathway would potentially lead to stereochemical scrambling, which is not experimentally observed.

Diels-Alder product (endo):

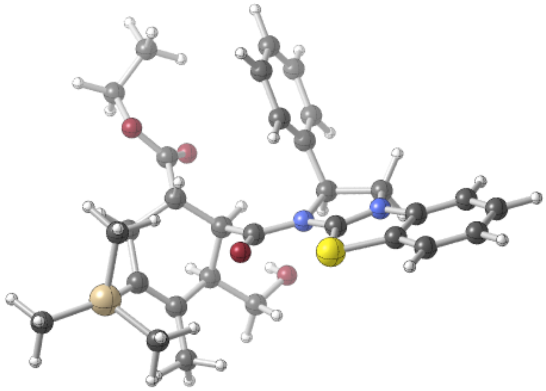

Charge = 1 Multiplicity = 1

HF = -2338.0058055 hartrees (-1467122.0230093 kcal/mol)

Imaginary Frequencies: none found

Zero-point correction = 0.633959 (Hartree/Particle)

Temperature 298.150 Kelvin. Pressure 1.00000 Atm.

Sum of electronic and thermal Free Energies =

-2337.442357 hartrees (-1466768.45344107 kcal/mol)

Coordinates (from last standard orientation):

Center Number	Atomic Number	Coordinates (Angstroms) X	Y	Z
1	6	-6.028443	-0.351319	-0.080054
2	6	-4.690019	-0.723403	-0.157688
3	6	-4.288515	-1.921822	-0.761050
4	6	-5.226731	-2.788241	-1.313068
5	6	-6.565766	-2.422412	-1.236920
6	6	-6.959804	-1.222396	-0.629220
7	6	-2.425956	-0.625474	0.121761
8	6	-3.429889	1.213020	1.094257
9	6	-1.882555	1.407563	1.092278
10	6	-1.424117	2.527680	0.182443
11	6	-1.435982	3.826881	0.693744
12	6	-1.030764	2.307182	-1.138233
13	6	-1.080916	4.901352	-0.115480
14	6	-0.660789	3.384321	-1.943236
15	6	-0.695461	4.681438	-1.437213
16	6	-0.064188	-0.349901	0.432696
17	6	1.030634	0.497998	1.028591

18	6	2.096054	0.814298	-0.040044
19	6	1.637913	-0.251262	2.253611
20	6	3.436451	0.059632	0.187534
21	6	0.564762	-0.548277	3.302699
22	6	3.239903	-1.310369	0.779558
23	6	5.014138	-4.159729	-1.619768
24	6	2.881116	-2.059195	-2.420930
25	6	2.109434	-4.360309	-0.574532
26	6	2.404383	-1.485010	1.810480
27	6	2.422808	2.298080	-0.050745
28	6	3.769418	3.914312	-1.142624
29	6	2.788152	4.972579	-1.598358
30	6	2.214542	-2.806891	2.499354
31	1	-6.319913	0.580264	0.393770
32	1	-4.923165	-3.717345	-1.784164
33	1	-7.317853	-3.082011	-1.656557
34	1	-8.013222	-0.966725	-0.586014
35	1	-3.950065	2.034689	0.600918
36	1	-3.830181	1.055062	2.097785
37	1	-1.724684	3.994879	1.728677
38	1	-1.010108	1.302026	-1.553162
39	1	-1.096117	5.909059	0.288300
40	1	-0.353297	3.206353	-2.969441
41	1	-0.412114	5.519379	-2.066962
42	1	-1.522654	1.561009	2.110883
43	1	0.614498	1.437153	1.393606
44	1	2.335159	0.477454	2.699376
45	1	3.982795	-0.006162	-0.755071
46	1	4.057026	0.651383	0.876117
47	1	1.726294	0.546877	-1.034932
48	1	-0.154449	-1.287120	2.930642
49	1	1.030030	-0.970942	4.201197
50	1	5.832012	-3.481537	-1.886159
51	1	5.371861	-4.822824	-0.824431
52	1	4.791916	-4.779467	-2.495951
53	1	1.998490	-1.506515	-2.077925
54	1	3.642734	-1.340536	-2.742409
55	1	2.580315	-2.640742	-3.300945
56	1	2.421812	-5.077173	0.192118
57	1	1.257329	-3.787692	-0.191272
58	1	1.762966	-4.930953	-1.445163
59	1	4.599336	3.813112	-1.844034
60	1	4.166262	4.137976	-0.148802
61	1	3.311966	5.926733	-1.713130
62	1	2.351706	4.701040	-2.564440
63	1	1.987679	5.099019	-0.866510

64	1	0.405028	1.251836	4.033577
65	1	1.193137	-3.190472	2.382963
66	1	2.903446	-3.547444	2.088112
67	1	2.409402	-2.728755	3.575831
68	8	0.109793	-1.390329	-0.170740
69	8	4.017715	-2.310585	0.257228
70	8	2.130780	3.085246	0.820760
71	8	3.172643	2.600597	-1.114210
72	8	-0.196944	0.603787	3.630219
73	7	-3.584223	-0.024649	0.319241
74	7	-1.390714	0.079285	0.623290
75	16	-2.528362	-2.123607	-0.704677
76	14	3.498730	-3.212665	-1.079601

2,6-lutidine/2,6-dimethylpyridine (DMP):

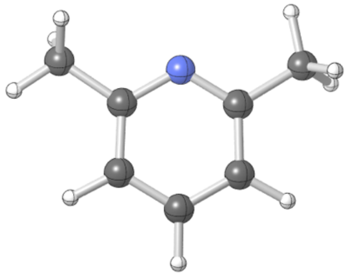

Charge = 0 Multiplicity = 1

HF = -326.7824947 hartrees (-205059.283249197 kcal/mol)

Imaginary Frequencies: none found

Zero-point correction = 0.145307 (Hartree/Particle)

Temperature 298.150 Kelvin. Pressure 1.00000 Atm.

Sum of electronic and thermal Free Energies =

-326.669833 hartrees (-204988.58690583 kcal/mol)

Coordinates (from last standard orientation):

Center Number	Atomic Number	Coordinates (Angstroms) X	Y	Z
1	6	-1.160473	-0.261946	-0.004035
2	6	-1.195166	1.131299	-0.003236
3	6	0.008859	1.831739	0.000842
4	6	1.199720	1.121255	0.003073
5	6	1.149738	-0.278042	0.001039
6	7	-0.006143	-0.949731	-0.002114

7	1	0.013558	2.918072	0.001370
8	1	-2.147267	1.652127	-0.005575
9	1	2.159615	1.628875	0.005136
10	6	2.418847	-1.088539	-0.000521
11	1	3.018921	-0.873332	-0.891088
12	1	3.035787	-0.852185	0.872816
13	1	2.182057	-2.154074	0.013626
14	6	-2.421617	-1.082545	0.002318
15	1	-2.405224	-1.812259	-0.812977
16	1	-2.505201	-1.644612	0.938663
17	1	-3.308700	-0.453813	-0.104057

Chloride anion (Cl^-):

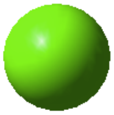

Charge = -1 Multiplicity = 1

HF = -460.31703 hartrees (-288853.5394953 kcal/mol)

Imaginary Frequencies: none found

Zero-point correction = 0.000000 (Hartree/Particle)

Temperature 298.150 Kelvin. Pressure 1.00000 Atm.

Sum of electronic and thermal Free Energies =

-460.332053 hartrees (-288862.96657803 kcal/mol)

Coordinates (from last standard orientation):
atom 1 is isolated, type=Cl

Center Number	Atomic Number	Coordinates (Angstroms) X	Y	Z
1	17	0.000000	0.000000	0.000000

Protonated 2,6-lutidine:

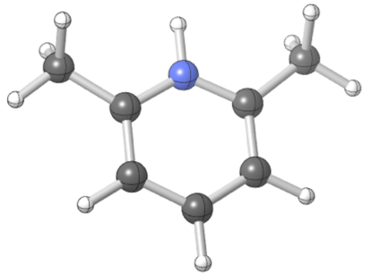

Charge = 1 Multiplicity = 1

HF = -327.2368823 hartrees (-205344.416012073 kcal/mol)

Imaginary Frequencies: none found

Zero-point correction = 0.159452 (Hartree/Particle)

Temperature 298.150 Kelvin. Pressure 1.00000 Atm.

Sum of electronic and thermal Free Energies =

-327.109815 hartrees (-205264.68001065 kcal/mol)

Coordinates (from last standard orientation):

Center Number	Atomic Number	Coordinates (Angstroms) X	Y	Z
1	6	1.201400	-0.224554	-0.000015
2	6	1.208756	1.160921	-0.000092
3	6	0.000000	1.851219	-0.000080
4	6	-1.208755	1.160921	-0.000012
5	6	-1.201400	-0.224553	0.000024
6	7	0.000000	-0.844275	0.000028
7	1	0.000001	2.936645	-0.000116
8	1	2.157473	1.683848	-0.000142
9	1	-2.157473	1.683848	-0.000001
10	1	0.000000	-1.866334	0.000068
11	6	-2.420240	-1.085936	-0.000100
12	1	-2.434712	-1.727254	-0.886837
13	1	-2.434389	-1.728149	0.885984
14	1	-3.316122	-0.464886	0.000346
15	6	2.420240	-1.085936	0.000205
16	1	2.434744	-1.727083	0.887066
17	1	2.434356	-1.728321	-0.885754
18	1	3.316122	-0.464886	-0.000389

(S)-(–)-BTM catalyst:

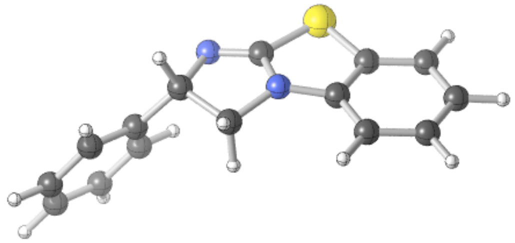

Charge = 0 Multiplicity = 1

HF = -1086.2217193 hartrees (-681614.991077943 kcal/mol)

Imaginary Frequencies: none found

Zero-point correction = 0.238948 (Hartree/Particle)

Temperature 298.150 Kelvin. Pressure 1.00000 Atm.

Sum of electronic and thermal Free Energies =

-1086.024409 hartrees (-681491.17689159 kcal/mol)

Coordinates (from last standard orientation):

Center Number	Atomic Number	Coordinates (Angstroms) X	Y	Z
1	6	-2.658926	-1.872440	0.032577
2	6	-2.123816	-0.600345	-0.141211
3	6	-2.895105	0.543447	0.132450
4	6	-4.207606	0.432725	0.561787
5	6	-4.746737	-0.845244	0.732328
6	6	-3.978352	-1.979555	0.473061
7	1	-2.058599	-2.752062	-0.178240
8	1	-4.800047	1.317910	0.772337
9	1	-5.771715	-0.949580	1.072628
10	1	-4.410212	-2.965542	0.613459
11	6	-0.539268	1.070167	-0.533267
12	16	-1.966889	2.036351	-0.147291
13	7	-0.860156	-0.271737	-0.595974
14	6	0.395774	-1.012047	-0.630557
15	6	1.379397	0.139158	-1.027978
16	7	0.675758	1.406318	-0.721608
17	6	2.713946	0.013122	-0.330895
18	6	3.702254	-0.807909	-0.878940
19	6	2.957188	0.651293	0.886801
20	6	4.914045	-0.995064	-0.218440
21	1	3.520707	-1.300931	-1.831631

22	6	4.171891	0.470832	1.545054
23	1	2.193991	1.298559	1.309570
24	6	5.151970	-0.354415	0.996466
25	1	5.674328	-1.635977	-0.655503
26	1	4.353595	0.977589	2.488602
27	1	6.098468	-0.494531	1.510257
28	1	0.621577	-1.422838	0.361589
29	1	0.374413	-1.817883	-1.366373
30	1	1.544570	0.099252	-2.112384

Ketene intermediate (INT):

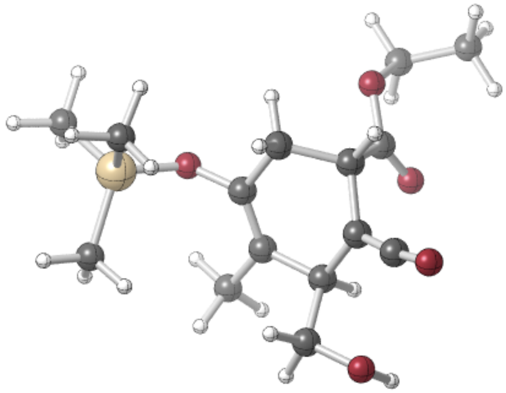

Charge = 0 Multiplicity = 1

HF = -1251.2691394 hartrees (-785183.897664894 kcal/mol)

Imaginary Frequencies: none found

Zero-point correction = 0.375508 (Hartree/Particle)

Temperature 298.150 Kelvin. Pressure 1.00000 Atm.

Sum of electronic and thermal Free Energies =

-1250.949835 hartrees (-784983.53096085 kcal/mol)

Coordinates (from last standard orientation):

Center Number	Atomic Number	Coordinates (Angstroms) X	Y	Z
1	6	-2.152541	2.153866	1.238162
2	6	-1.480118	1.298835	0.495865
3	6	-1.467636	-0.145911	0.940932
4	6	-0.732124	1.650035	-0.772577
5	6	-0.009930	-0.607475	1.078228

6	6	-0.257704	3.105743	-0.724193
7	6	0.785995	-0.239762	-0.148516
8	6	4.433354	-2.198937	-0.163620
9	6	3.260085	-0.525171	2.152339
10	6	4.082892	0.865049	-0.443207
11	6	0.446490	0.726208	-1.015832
12	6	-2.221130	-1.017773	-0.062498
13	6	-2.828381	-3.244423	-0.582256
14	6	-4.307082	-3.295111	-0.256882
15	6	1.211121	0.960507	-2.291367
16	1	-1.414400	1.549733	-1.632779
17	1	0.424618	-0.153917	1.976725
18	1	0.038668	-1.693211	1.206951
19	1	-1.976517	-0.271521	1.903216
20	1	0.498933	3.217882	0.059556
21	1	0.190610	3.394555	-1.683021
22	1	4.022793	-3.115479	0.273986
23	1	4.475111	-2.334482	-1.249850
24	1	5.460628	-2.084650	0.200563
25	1	2.695176	0.371477	2.431277
26	1	2.777834	-1.392264	2.616900
27	1	4.261289	-0.427590	2.589681
28	1	4.230237	0.783615	-1.525256
29	1	3.416069	1.713746	-0.250860
30	1	5.053506	1.099022	0.011123
31	1	-2.343002	-4.202270	-0.387298
32	1	-2.655408	-2.961067	-1.623038
33	1	-4.793540	-4.053936	-0.877299
34	1	-4.461394	-3.556915	0.793853
35	1	-4.781086	-2.330381	-0.455336
36	1	1.871331	1.834384	-2.229404
37	1	1.826097	0.093723	-2.543955
38	1	0.514129	1.144051	-3.118524
39	8	-2.782245	2.800964	1.982772
40	8	1.877078	-1.047786	-0.364791
41	8	-2.824565	-0.612079	-1.028209
42	8	-2.140313	-2.311082	0.273470
43	8	-1.318246	3.981449	-0.381199
44	14	3.396618	-0.714234	0.292981
45	1	-1.957754	3.968968	-1.112753

c. **Lactonization step of DAL organocascade**

***iii.* Intramolecular proton-transfer pathway**

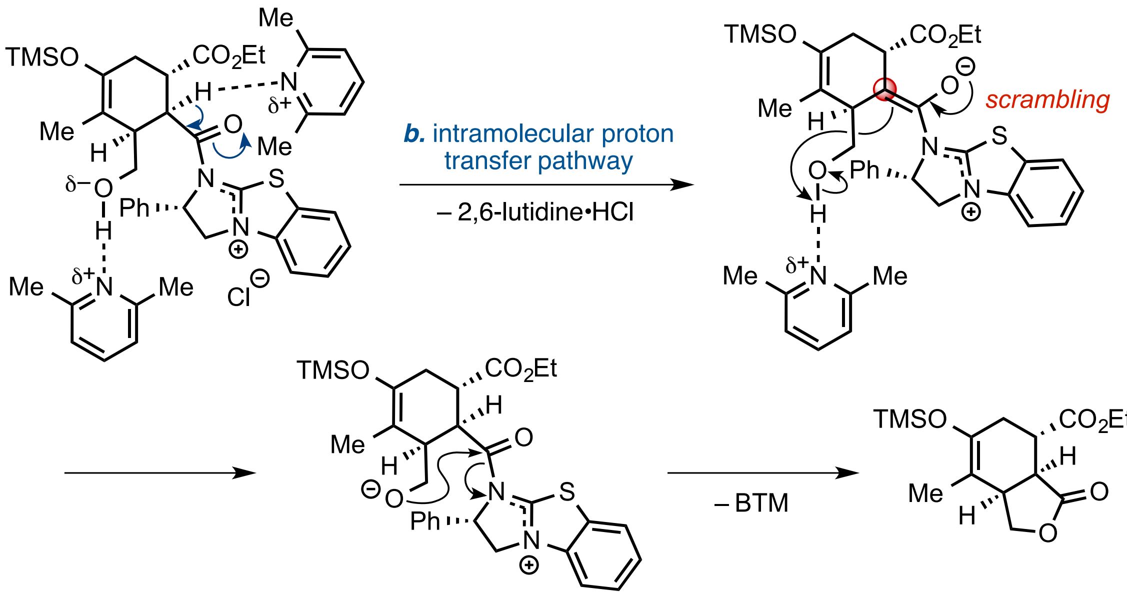

Scheme S4. An intramolecular proton-relay pathway was investigated for lactonization; however, this pathway would potentially lead to stereochemical scrambling, which is not experimentally observed.

Diels-Alder product (endo):

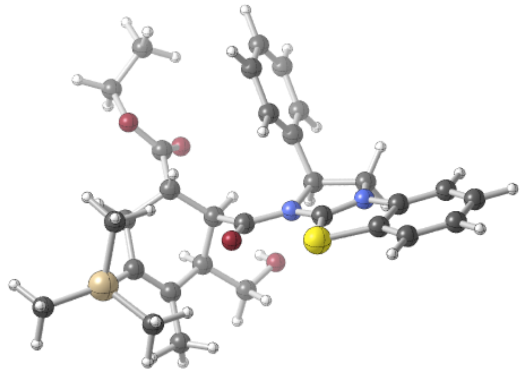

Charge = 1 Multiplicity = 1

HF = -2338.0058055 hartrees (-1467122.0230093 kcal/mol)

Imaginary Frequencies: none found

Zero-point correction = 0.633959 (Hartree/Particle)

Temperature 298.150 Kelvin. Pressure 1.00000 Atm.

Sum of electronic and thermal Free Energies =

-2337.442357 hartrees (-1466768.45344107 kcal/mol)

Coordinates (from last standard orientation):

Center Number	Atomic Number	Coordinates (Angstroms) X	Y	Z
1	6	-6.028443	-0.351319	-0.080054
2	6	-4.690019	-0.723403	-0.157688
3	6	-4.288515	-1.921822	-0.761050
4	6	-5.226731	-2.788241	-1.313068
5	6	-6.565766	-2.422412	-1.236920
6	6	-6.959804	-1.222396	-0.629220
7	6	-2.425956	-0.625474	0.121761
8	6	-3.429889	1.213020	1.094257
9	6	-1.882555	1.407563	1.092278
10	6	-1.424117	2.527680	0.182443
11	6	-1.435982	3.826881	0.693744
12	6	-1.030764	2.307182	-1.138233
13	6	-1.080916	4.901352	-0.115480
14	6	-0.660789	3.384321	-1.943236
15	6	-0.695461	4.681438	-1.437213
16	6	-0.064188	-0.349901	0.432696
17	6	1.030634	0.497998	1.028591

18	6	2.096054	0.814298	-0.040044
19	6	1.637913	-0.251262	2.253611
20	6	3.436451	0.059632	0.187534
21	6	0.564762	-0.548277	3.302699
22	6	3.239903	-1.310369	0.779558
23	6	5.014138	-4.159729	-1.619768
24	6	2.881116	-2.059195	-2.420930
25	6	2.109434	-4.360309	-0.574532
26	6	2.404383	-1.485010	1.810480
27	6	2.422808	2.298080	-0.050745
28	6	3.769418	3.914312	-1.142624
29	6	2.788152	4.972579	-1.598358
30	6	2.214542	-2.806891	2.499354
31	1	-6.319913	0.580264	0.393770
32	1	-4.923165	-3.717345	-1.784164
33	1	-7.317853	-3.082011	-1.656557
34	1	-8.013222	-0.966725	-0.586014
35	1	-3.950065	2.034689	0.600918
36	1	-3.830181	1.055062	2.097785
37	1	-1.724684	3.994879	1.728677
38	1	-1.010108	1.302026	-1.553162
39	1	-1.096117	5.909059	0.288300
40	1	-0.353297	3.206353	-2.969441
41	1	-0.412114	5.519379	-2.066962
42	1	-1.522654	1.561009	2.110883
43	1	0.614498	1.437153	1.393606
44	1	2.335159	0.477454	2.699376
45	1	3.982795	-0.006162	-0.755071
46	1	4.057026	0.651383	0.876117
47	1	1.726294	0.546877	-1.034932
48	1	-0.154449	-1.287120	2.930642
49	1	1.030030	-0.970942	4.201197
50	1	5.832012	-3.481537	-1.886159
51	1	5.371861	-4.822824	-0.824431
52	1	4.791916	-4.779467	-2.495951
53	1	1.998490	-1.506515	-2.077925
54	1	3.642734	-1.340536	-2.742409
55	1	2.580315	-2.640742	-3.300945
56	1	2.421812	-5.077173	0.192118
57	1	1.257329	-3.787692	-0.191272
58	1	1.762966	-4.930953	-1.445163
59	1	4.599336	3.813112	-1.844034
60	1	4.166262	4.137976	-0.148802
61	1	3.311966	5.926733	-1.713130
62	1	2.351706	4.701040	-2.564440
63	1	1.987679	5.099019	-0.866510

64	1	0.405028	1.251836	4.033577
65	1	1.193137	-3.190472	2.382963
66	1	2.903446	-3.547444	2.088112
67	1	2.409402	-2.728755	3.575831
68	8	0.109793	-1.390329	-0.170740
69	8	4.017715	-2.310585	0.257228
70	8	2.130780	3.085246	0.820760
71	8	3.172643	2.600597	-1.114210
72	8	-0.196944	0.603787	3.630219
73	7	-3.584223	-0.024649	0.319241
74	7	-1.390714	0.079285	0.623290
75	16	-2.528362	-2.123607	-0.704677
76	14	3.498730	-3.212665	-1.079601

2,6-lutidine/2,6-dimethylpyridine (DMP):

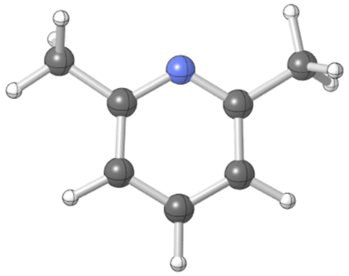

Charge = 0 Multiplicity = 1

HF = -326.7824947 hartrees (-205059.283249197 kcal/mol)

Imaginary Frequencies: none found

Zero-point correction = 0.145307 (Hartree/Particle)

Temperature 298.150 Kelvin. Pressure 1.00000 Atm.

Sum of electronic and thermal Free Energies =

-326.669833 hartrees (-204988.58690583 kcal/mol)

Coordinates (from last standard orientation):

Center Number	Atomic Number	Coordinates (Angstroms) X	Y	Z
1	6	-1.160473	-0.261946	-0.004035
2	6	-1.195166	1.131299	-0.003236
3	6	0.008859	1.831739	0.000842
4	6	1.199720	1.121255	0.003073
5	6	1.149738	-0.278042	0.001039
6	7	-0.006143	-0.949731	-0.002114

7	1	0.013558	2.918072	0.001370
8	1	-2.147267	1.652127	-0.005575
9	1	2.159615	1.628875	0.005136
10	6	2.418847	-1.088539	-0.000521
11	1	3.018921	-0.873332	-0.891088
12	1	3.035787	-0.852185	0.872816
13	1	2.182057	-2.154074	0.013626
14	6	-2.421617	-1.082545	0.002318
15	1	-2.405224	-1.812259	-0.812977
16	1	-2.505201	-1.644612	0.938663
17	1	-3.308700	-0.453813	-0.104057

Chloride anion (Cl^-):

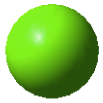

Charge = -1 Multiplicity = 1

HF = -460.31703 hartrees (-288853.5394953 kcal/mol)

Imaginary Frequencies: none found

Zero-point correction = 0.000000 (Hartree/Particle)

Temperature 298.150 Kelvin. Pressure 1.00000 Atm.

Sum of electronic and thermal Free Energies =

-460.332053 hartrees (-288862.96657803 kcal/mol)

Coordinates (from last standard orientation):
atom 1 is isolated, type=Cl

Center Number	Atomic Number	Coordinates (Angstroms) X	Y	Z
1	17	0.000000	0.000000	0.000000

Protonated 2,6-lutidine:

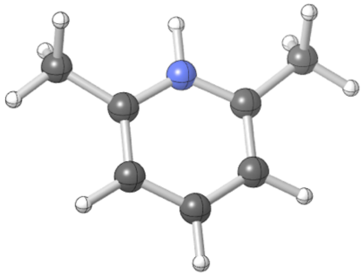

Charge = 1 Multiplicity = 1

HF = -327.2368823 hartrees (-205344.416012073 kcal/mol)

Imaginary Frequencies: none found

Zero-point correction = 0.159452 (Hartree/Particle)

Temperature 298.150 Kelvin. Pressure 1.00000 Atm.

Sum of electronic and thermal Free Energies =

-327.109815 hartrees (-205264.68001065 kcal/mol)

Coordinates (from last standard orientation):

Center Number	Atomic Number	Coordinates (Angstroms) X	Y	Z
1	6	1.201400	-0.224554	-0.000015
2	6	1.208756	1.160921	-0.000092
3	6	0.000000	1.851219	-0.000080
4	6	-1.208755	1.160921	-0.000012
5	6	-1.201400	-0.224553	0.000024
6	7	0.000000	-0.844275	0.000028
7	1	0.000001	2.936645	-0.000116
8	1	2.157473	1.683848	-0.000142
9	1	-2.157473	1.683848	-0.000001
10	1	0.000000	-1.866334	0.000068
11	6	-2.420240	-1.085936	-0.000100
12	1	-2.434712	-1.727254	-0.886837
13	1	-2.434389	-1.728149	0.885984
14	1	-3.316122	-0.464886	0.000346
15	6	2.420240	-1.085936	0.000205
16	1	2.434744	-1.727083	0.887066
17	1	2.434356	-1.728321	-0.885754
18	1	3.316122	-0.464886	-0.000389

Enolate intermediate (INT):

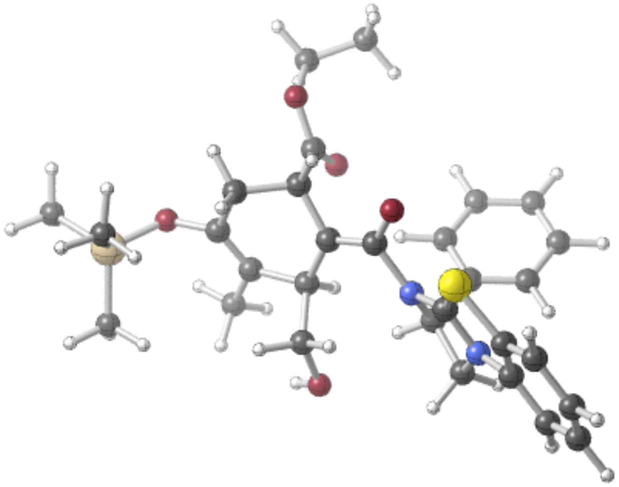

Charge = 0 Multiplicity = 1

HF = -2337.5007733 hartrees (-1466805.11025348 kcal/mol)

Imaginary Frequencies: none found

Zero-point correction = 0.619013 (Hartree/Particle)

Temperature 298.150 Kelvin. Pressure 1.00000 Atm.

Sum of electronic and thermal Free Energies =

-2336.955023 hartrees (-1466462.64648273 kcal/mol)

Coordinates (from last standard orientation):

Center Number	Atomic Number	Coordinates (Angstroms) X	Y	Z
1	6	-6.042990	-1.841234	0.704217
2	6	-4.930834	-1.449894	-0.033047
3	6	-4.886078	-1.588323	-1.427973
4	6	-5.970089	-2.115606	-2.118042
5	6	-7.087767	-2.511486	-1.385510
6	6	-7.120878	-2.376951	0.005776
7	6	-2.822350	-0.654539	-0.494133
8	6	-3.158546	-0.710344	1.771056
9	6	-1.937728	0.166447	1.427690
10	6	-2.172841	1.665752	1.478073
11	6	-1.058673	2.510897	1.458580
12	6	-3.452610	2.227464	1.489578
13	6	-1.222097	3.892921	1.470001
14	6	-3.614001	3.611939	1.501800
15	6	-2.500203	4.447965	1.496820

16	6	-0.556775	0.128110	-0.904758
17	6	0.724008	0.086505	-0.409126
18	6	1.769383	0.681221	-1.312115
19	6	1.172417	-0.421523	0.949001
20	6	3.041883	-0.179583	-1.360952
21	6	0.577264	-1.818080	1.307505
22	6	3.507918	-0.483509	0.037380
23	6	7.455654	-1.745918	-0.016473
24	6	5.130649	-2.853746	-1.715407
25	6	5.143529	-3.319453	1.301767
26	6	2.684833	-0.523976	1.092299
27	6	2.164515	2.089011	-0.874219
28	6	3.322775	4.044957	-1.541943
29	6	2.211647	5.045484	-1.791956
30	6	3.195372	-0.769532	2.486696
31	1	-6.059302	-1.731672	1.783765
32	1	-5.945798	-2.221457	-3.198134
33	1	-7.942318	-2.932139	-1.905199
34	1	-8.001966	-2.693192	0.554720
35	1	-3.853463	-0.236480	2.464203
36	1	-2.848468	-1.685747	2.159922
37	1	-0.057030	2.093539	1.403395
38	1	-4.337602	1.596951	1.488132
39	1	-0.345955	4.534532	1.459525
40	1	-4.614108	4.034825	1.515321
41	1	-2.628562	5.526226	1.510175
42	1	-1.094820	-0.116526	2.056034
43	1	0.851010	0.253740	1.762389
44	1	2.827132	-1.102287	-1.914678
45	1	3.847753	0.338047	-1.892391
46	1	1.361439	0.776736	-2.322504
47	1	-0.321931	-2.019446	0.723684
48	1	1.307982	-2.599989	1.050840
49	1	7.733020	-1.062914	-0.826872
50	1	7.745659	-1.279021	0.931038
51	1	8.045491	-2.662271	-0.133280
52	1	4.075121	-3.148418	-1.727480
53	1	5.297014	-2.149113	-2.537723
54	1	5.724046	-3.752808	-1.922487
55	1	5.446537	-2.958937	2.290482
56	1	4.060237	-3.487368	1.314931
57	1	5.624345	-4.291817	1.138364
58	1	4.164886	4.211820	-2.216911
59	1	3.677961	4.090640	-0.509581
60	1	2.581589	6.061059	-1.619760
61	1	1.858985	4.976316	-2.825121

62	1	1.369462	4.862693	-1.119746
63	1	3.012545	-1.801828	2.819465
64	1	4.272678	-0.599357	2.550662
65	1	2.698597	-0.100734	3.201092
66	8	-0.970444	0.397742	-2.065345
67	8	4.871940	-0.639241	0.172409
68	8	1.901384	2.612165	0.186486
69	8	2.885651	2.705829	-1.828162
70	8	0.174949	-1.932306	2.666465
71	7	-3.753116	-0.884653	0.441817
72	7	-1.674083	-0.244295	0.005698
73	16	-3.349752	-0.988784	-2.101944
74	14	5.627857	-2.127535	-0.060273
75	1	0.952096	-1.778292	3.225708
